# Supplementary material for: Non-viral precision T cell receptor replacement for personalized cell therapy
Source: Nature. 2022 Nov 10;615(7953):687–96. doi: 10.1038/s41586-022-05531-1 (PMC9768791; doi:10.1038/s41586-022-05531-1)
Supplement: Supplementary file 1 — Supplementary Figs. 1–8, Supplementary Tables 1 and 3 and the clinical trial protocol. [file 41586_2022_5531_MOESM1_ESM.pdf]

nature portfolio

https://doi.org/10.1038/s41586-022-05531-1

Supplementary information

Non-viral precision T cell receptor replacement for personalized cell therapy

In the format provided by the authors and unedited

Supplemental Information Guide for:

Non-viral precision T cell receptor replacement for personalized cell therapy

Susan P. Foy[1,13], Kyle Jacoby[1,13], Daniela A. Bota[2,13], Theresa Hunter[1], Zheng Pan[1], Eric Stawiski[1], Yan Ma[1], William Lu[1], Songming Peng[1], Clifford L. Wang[1], Benjamin Yuen[1], Olivier Dalmas[1], Katharine Heeringa[1], Barbara Sennino[1], Andy Conroy[1], Michael T. Bethune[1], Ines Mende[1], William White[1], Monica Kukreja[1], Swetha Gunturu[1], Emily Humphrey[1], Adeel Hussaini[1], Duo An[1], Adam J. Litterman[1], Boi Bryant Quach[1], Alphonsus H. C. Ng[3], Yue Lu[3], Chad Smith[1], Katie M. Campbell[4], Daniel Anaya[1], Lindsey Skrdlant[1], Eva Yi-Hsuan Huang[1], Ventura Mendoza[1], Jyoti Mathur[1], Luke Dengler[1], Bhamini Purandare[1], Robert Moot[1], Michael C. Yi1, Roel Funke[1], Alison Sibley[1], Todd Stallings-Schmitt[1], David Y. Oh[5], Bartosz Chmielowski[4,6], Mehrdad Abedi[7], Yuan Yuan[8], Jeffrey A. Sosman[9], Sylvia M. Lee[101], Adam J. Schoenfeld[11], David Baltimore[12], James R. Heath[3], Alex Franzusoff[1], Antoni Ribas[4,6,14], Arati V. Rao[1,14], Stefanie J. Mandl[1,14]

Affiliations:

1. PACT Pharma, South San Francisco, CA, USA.
2. Department of Neurology and Chao Family Comprehensive Cancer Center, University of California, Irvine, CA, USA.
3. Institute for Systems Biology, Seattle, WA, USA.
4. Department of Medicine, Division of Hematology-Oncology, University of California, Los Angeles (UCLA), Los Angeles, CA, USA.
5. Division of Hematology/Oncology, Department of Medicine, University of California, San Francisco, CA, USA.
6. Jonsson Comprehensive Cancer Center at the University of California Los Angeles, Los Angeles, CA, USA.
7. Division of Hematology/Oncology, Department of Internal Medicine, University of California Davis Comprehensive Cancer Center, Sacramento, CA, USA.
8. Department of Medical Oncology and Therapeutics Research, City of Hope National Medical Center, Duarte, CA, USA.
9. Department of Medicine and Robert H. Lurie Cancer Center, Northwestern University, Evanston, IL, USA.
10. Clinical Research Division, Fred Hutchinson Cancer Research Center, Seattle, WA, USA.
11. Thoracic Oncology Service, Division of Solid Tumor Oncology, Department of Medicine, Memorial Sloan Kettering Cancer Center, Weill Cornell Medical College, New York, NY, USA.
12. Division of Biology and Biological Engineering, California Institute of Technology, Pasadena, CA, USA.
13. These authors contributed equally: Susan P. Foy, Kyle Jacoby, and Daniela A. Bota.
14. These authors contributed equally: Antoni Ribas, Arati V. Rao, and Stefanie J. Mandl.

This PDF file includes:

- Supplemental Figures 1-8
- Supplemental Tables 1 and 3
- Clinical Trial Protocol

Table of Contents

Supplementary Information Figures 1

Supplementary Information Tables.. 9

Supplementary Information: Clinical Trial Protocol 12

List of Figures

Supplementary Information Figure 1. NeoTCR T Cell isolation gating strategy.......................... 1

Supplementary Information Figure 2. Proliferation gating strategy... 2

Supplementary Information Figure 3. Intracellular cytokine staining gating strategy. 3

Supplementary Information Figure 4. NeoTCR pharmacokinetics gating strategy. 4

Supplementary Information Figure 5. NeoTCR identification for lot release............................... 5

Supplementary Information Figure 6. Final cell product phenotype characterization gating strategy... 6

Supplementary Information Figure 7. Post- infusion phenotype characterization gating strategy. 7

Supplementary Information Figure 8. Post-infusion activation characterization gating strategy. . 8

List of Tables

Supplementary Information Table 1: Specific alleles including in the PACT-64 HLA panel and their prevalence in The Cancer Genome Atlas (TCGA) dataset.. 9

Supplementary Information Table 2. (separate file). Full length TCR sequences for the 37 TCRs infused into patients. 11

Supplementary Information Table 3. Clinical Cell Manufacturing Process Versions................. 11

Supplementary Information Table 4. (separate file). List of flow cytometry reagents................ 11

Supplementary Information Figures

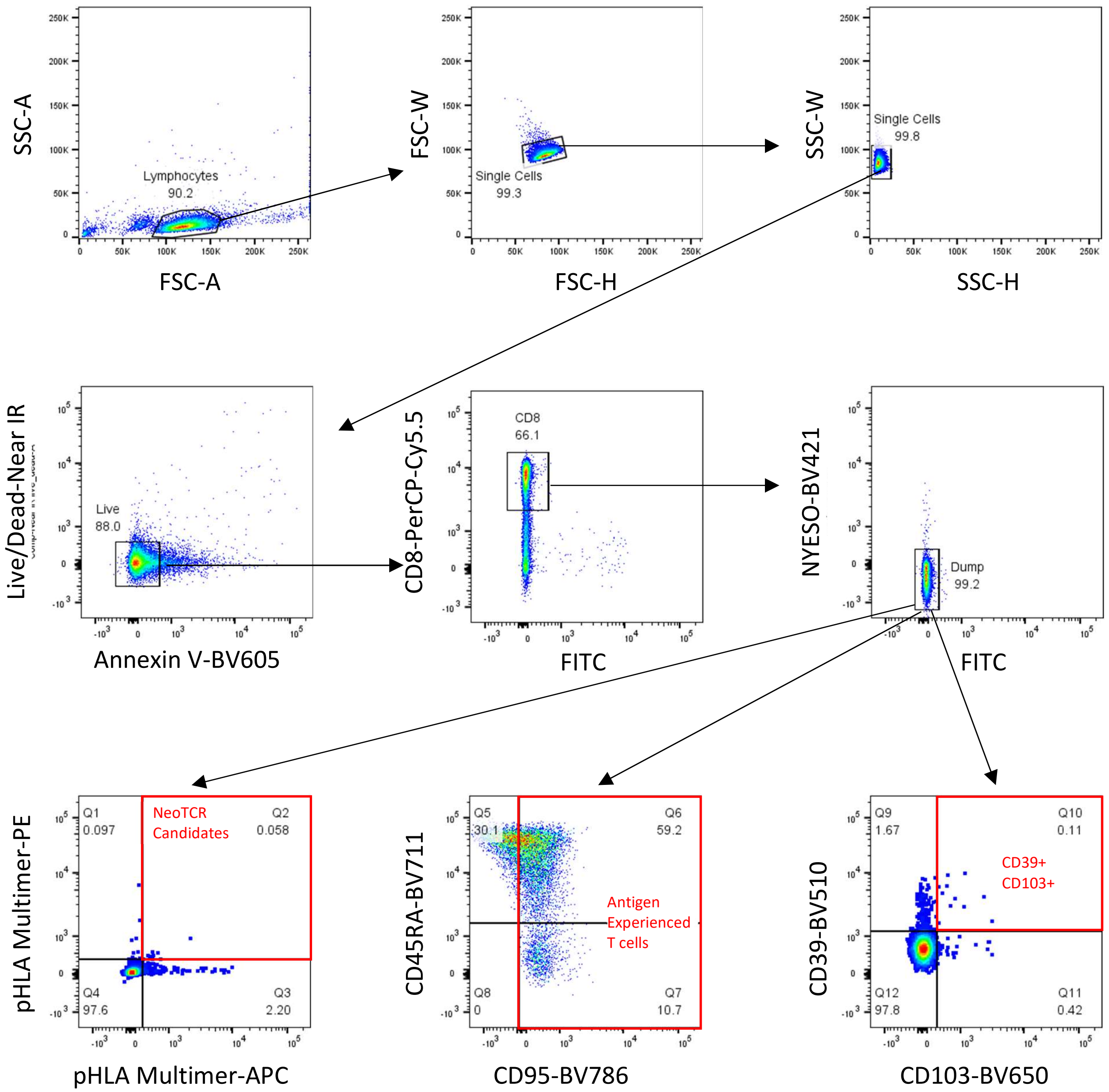

Supplementary Information Figure 1. NeoTCR T Cell isolation gating strategy.

Cells are gated for lymphocytes, single cells (i.e., exclusion of doublets), and live cells. Cells are then gated for CD8+ T cells with cells falling into a lineage negative dump gate excluded. These CD8+ T cells are then gated for neoantigen-HLA multimer+ cells using two colors, APC and PE. These neoTCR+ candidates are then single cell sorted for further analysis and determination of TCR sequences. Staining of CD39, CD103, CD45RA, and CD95 enabled determination of T cell phenotypes. Used for Figure 1b, 1c.

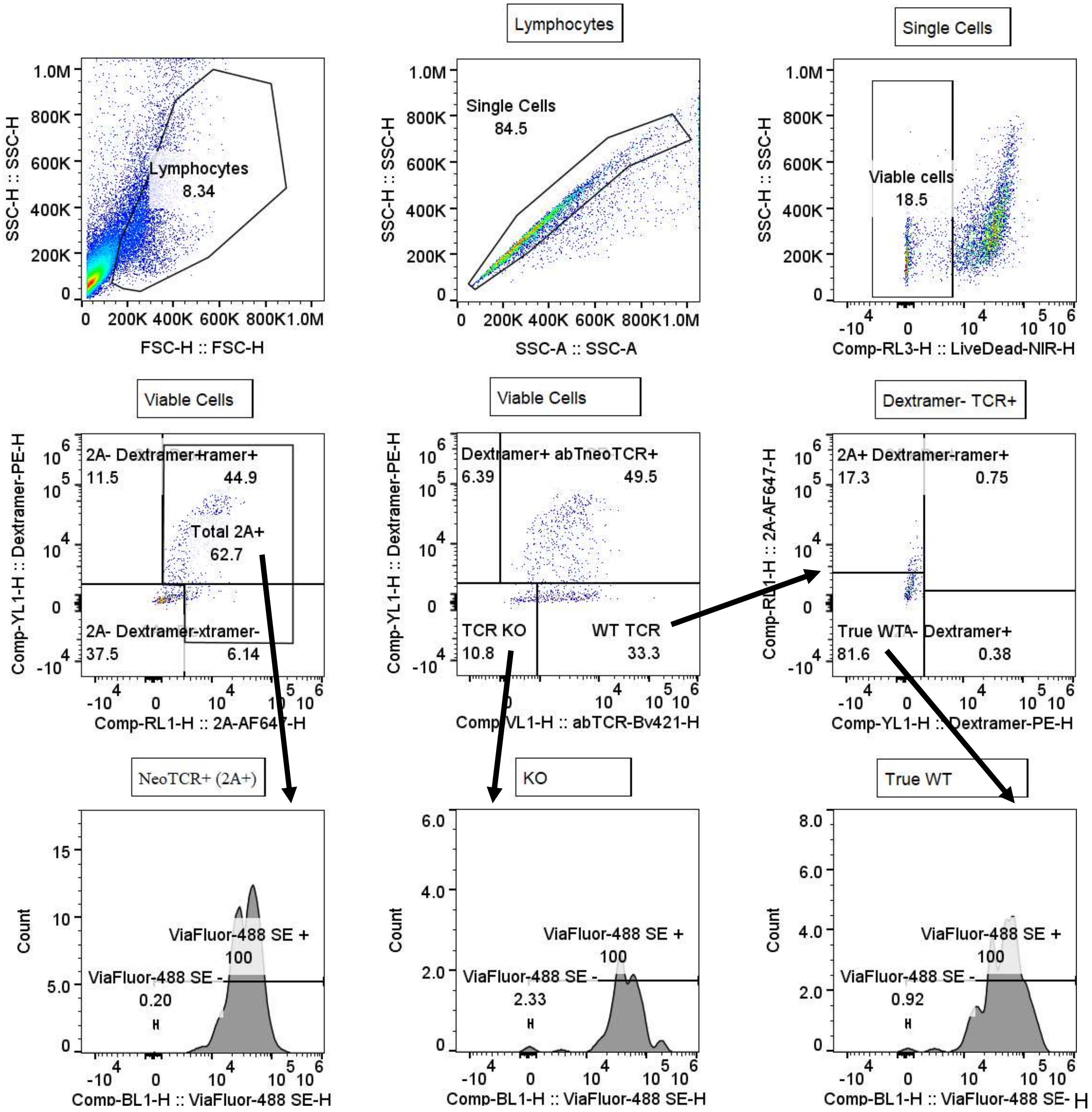

Supplementary Information Figure 2. Proliferation gating strategy.

Cells are gated for size, singlets, and viability. Cells are then gated on expression of the 2A peptide, indicating successful integration of the neoTCR transgene. KO T cells are identified by cells that are TCR negative. True WT cells are identified by TCR+ cells that are also 2A negative. Used for Figure 2d, Extended Data Figure 2b-c.

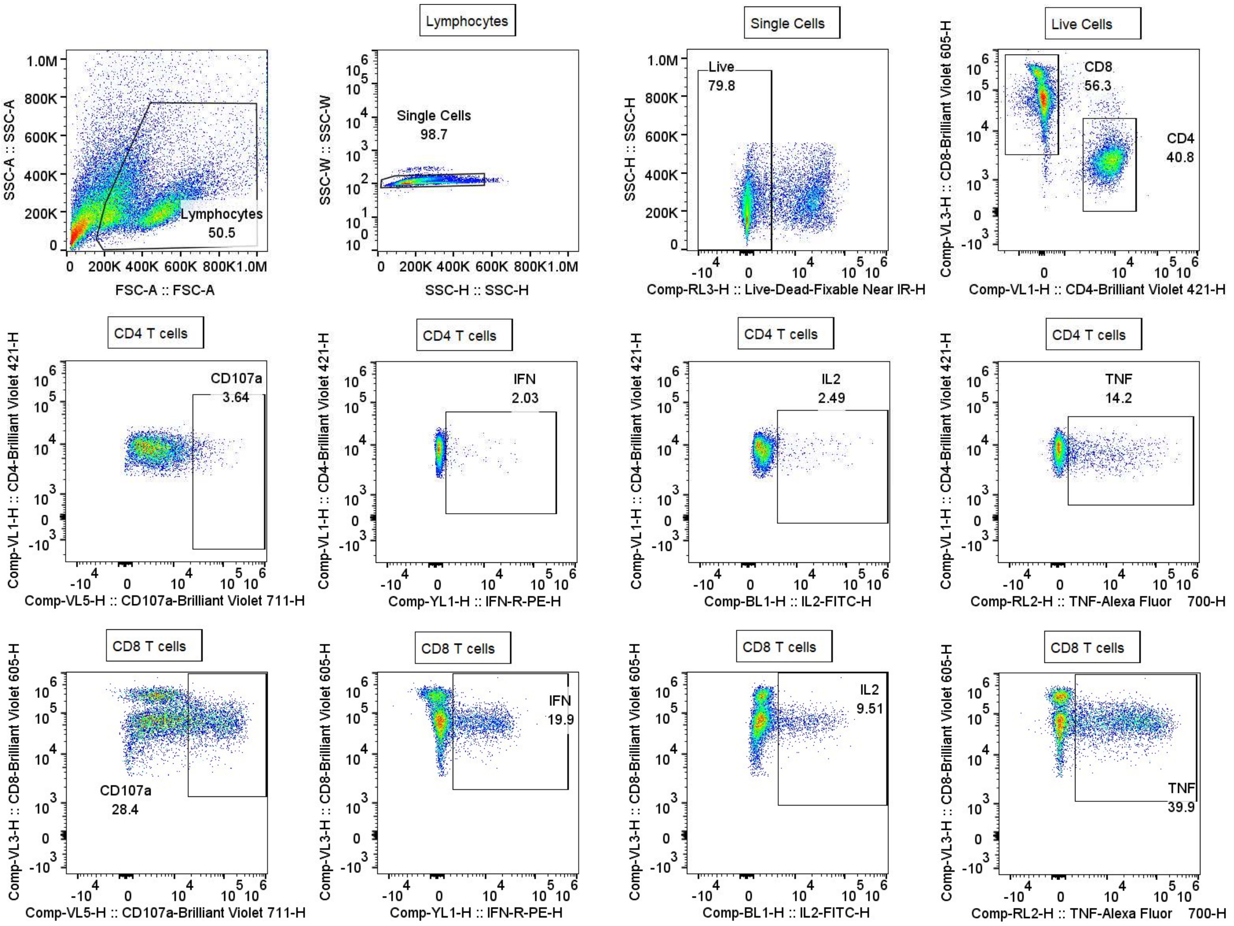

Supplementary Information Figure 3. Intracellular cytokine staining gating strategy.

Cells are gated for size, singlets, and viability. CD4 and CD8 T cells are then gated separately for expression of CD107a, IFN-gamma, IL-2, and TNFα. Used for Extended Data Figure 2a.

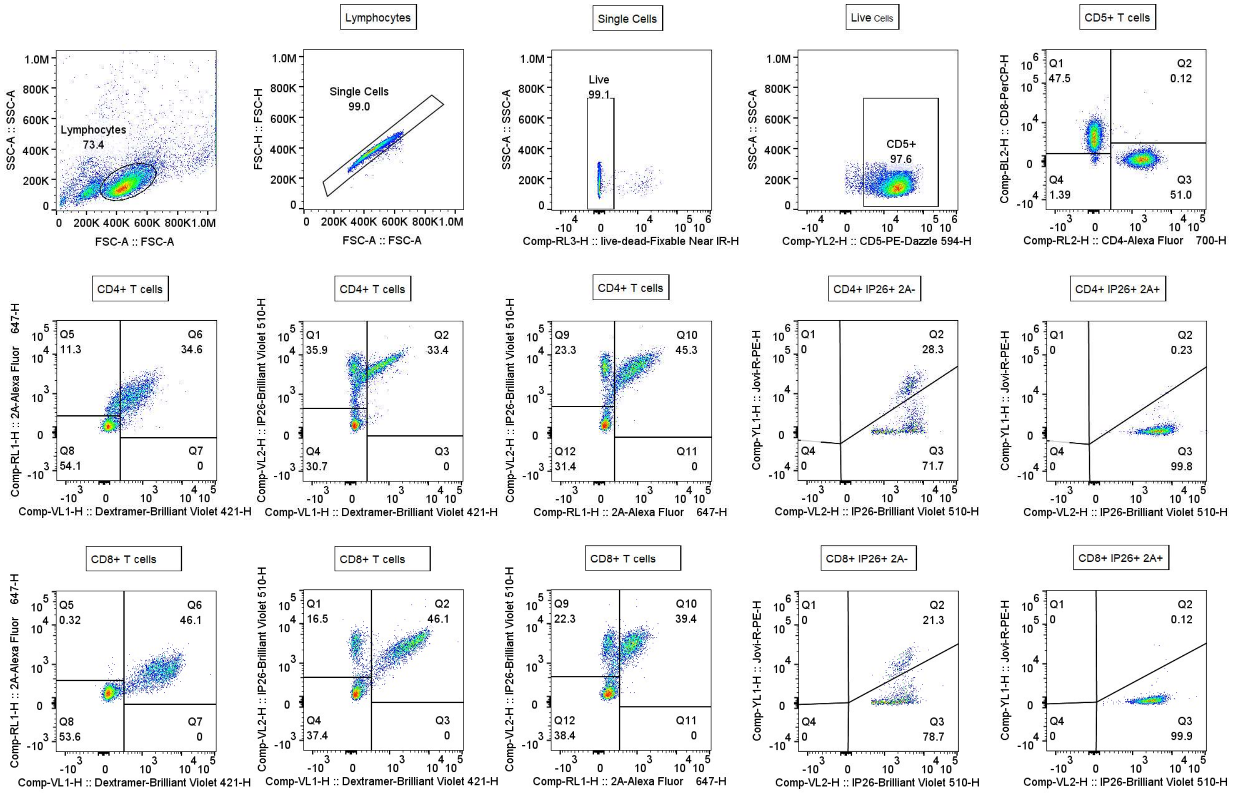

Supplementary Information Figure 4. NeoTCR pharmacokinetics gating strategy.

Cells are gated for size, singlets, and viability. T cells are identified as CD5+. CD4 and CD8 T cells are separated and gated three ways: dextramer and 2A, dextramer and TCRαβ-IP26 (binds all TCRs), 2A and TCRαβ. WT (2A- TCRαβ-IP26+) and neoTCR+ (2A+ TCRαβ-IP26+) cells are further gated for TCRαβ-JOVI.1 and TCRαβ-IP26. TCRαβ-JOVI.1 binds only TRBC1+ T cells, while neoTCR cells express only *TRBC2*. Used for Figure 2b, 3b, Extended Data Figure 1d, Extended Data Figure 4c-d.

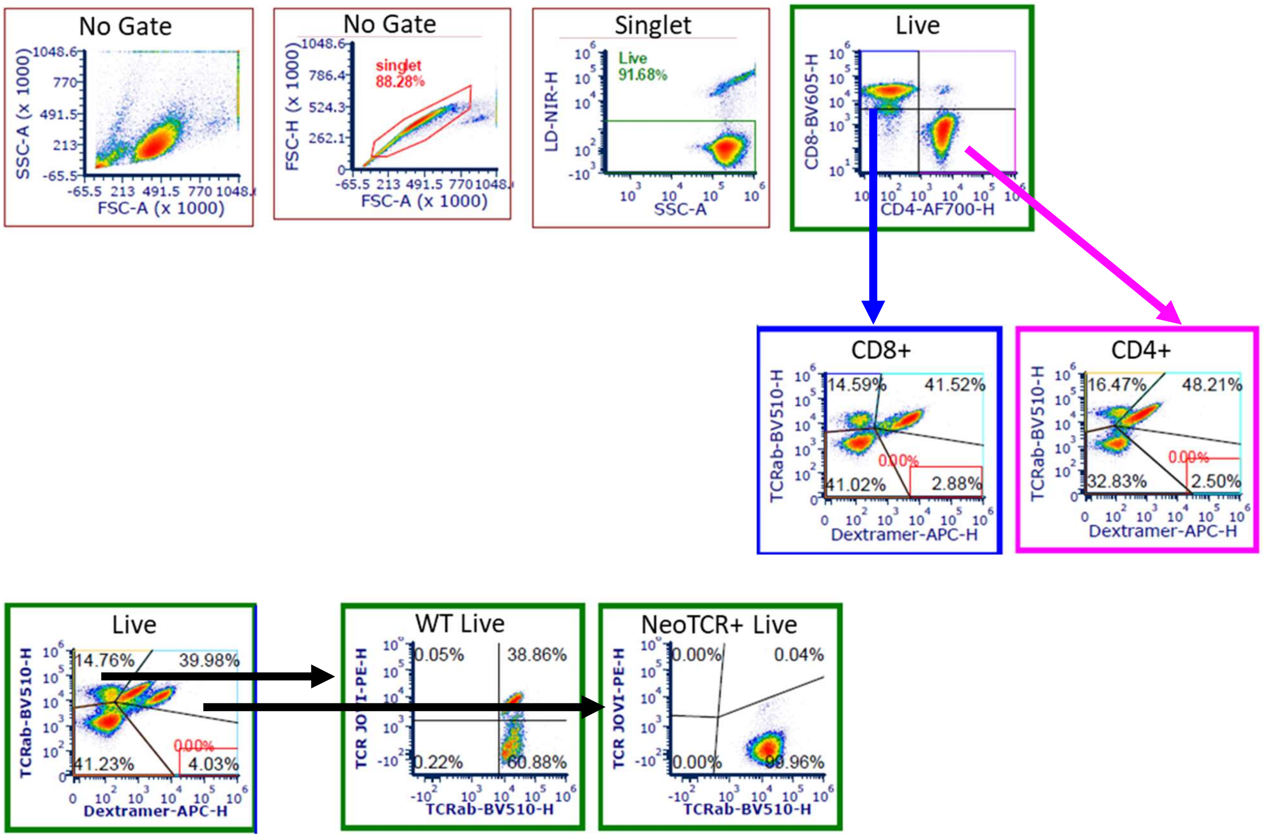

Supplementary Information Figure 5. NeoTCR identification for lot release.

Cells are gated for singlets and viability. % NeoTCR+ (dextramer+) is determined on total live cells, CD4 cells alone, and CD8 cells alone. WT (dextramer- TCRαβ-IP26+) and neoTCR+ (dextramer+ TCRαβ-IP26+) cells are further gated for TCRαβ-JOVI.1 and TCRαβ-IP26. TCRαβ-JOVI.1 binds only TRBC1+ T cells, while neoTCR cells express only TRBC2. Used for Extended Data Figure 3c.

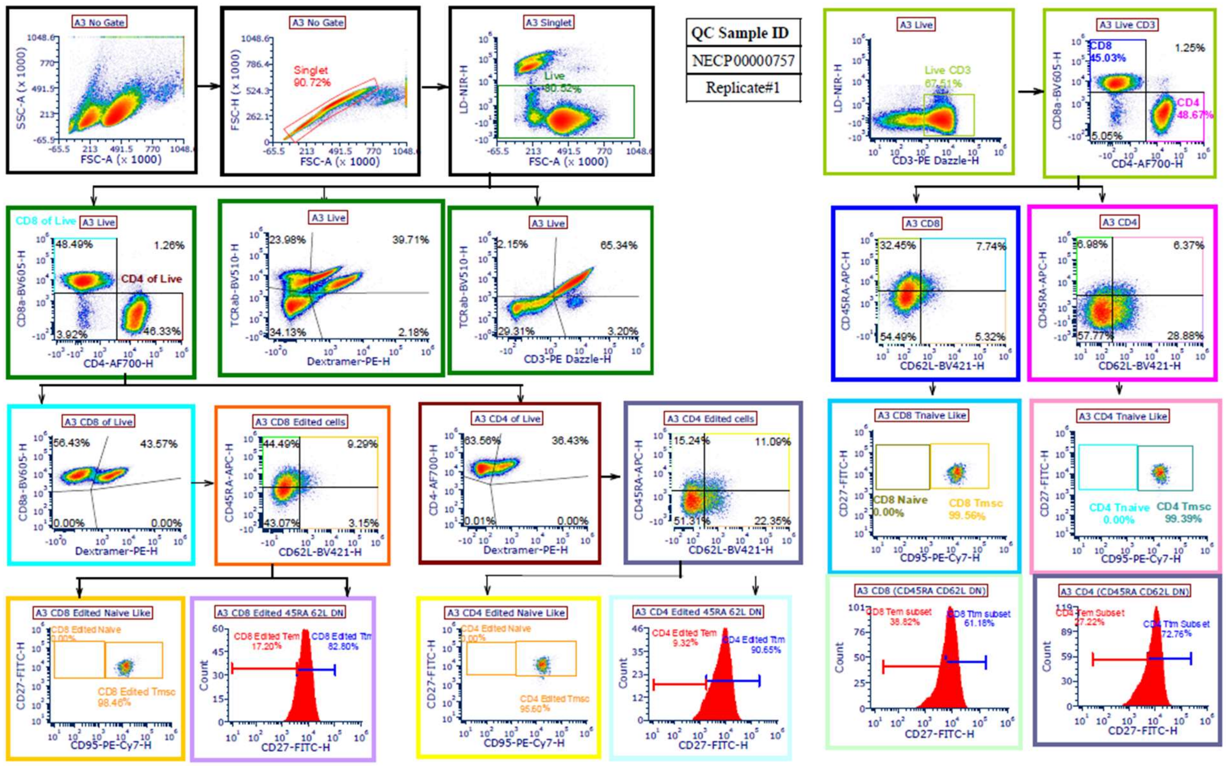

Supplementary Information Figure 6. Final cell product phenotype characterization gating strategy.

Cells are gated on singlets and viability. They are then gated on all CD3 cells (right side). CD3 cells are then gated on CD4 and CD8, and further gated on CD62L and CD45RA. CD62L+ CD45RA+ cells are designated "naïve-like" and are then gated on CD95+CD27+ (Tmsc, memory stem cells) and CD95-CD27+ (Tn, naïve). CD62L- CD45RA+ cells are designated Teff (effector). CD62L+ CD45RA- cells are designated Tcm (central memory). CD45RA- CD62L- cells (DN) are further gated as CD27+ (Ttm, transitional memory) and CD27- (Tem, effector memory). Total live cells are also directly gated for CD4 and CD8 cells, then gated for binding of the peptide-HLA multimer (dextramer-PE, left side) to assess phenotype of neoTCR cells specifically. After identifying dextramer+ CD4 and CD8 cells, they are gated in the same manner as described above. Used for Extended Data Figure 3a, 5b.

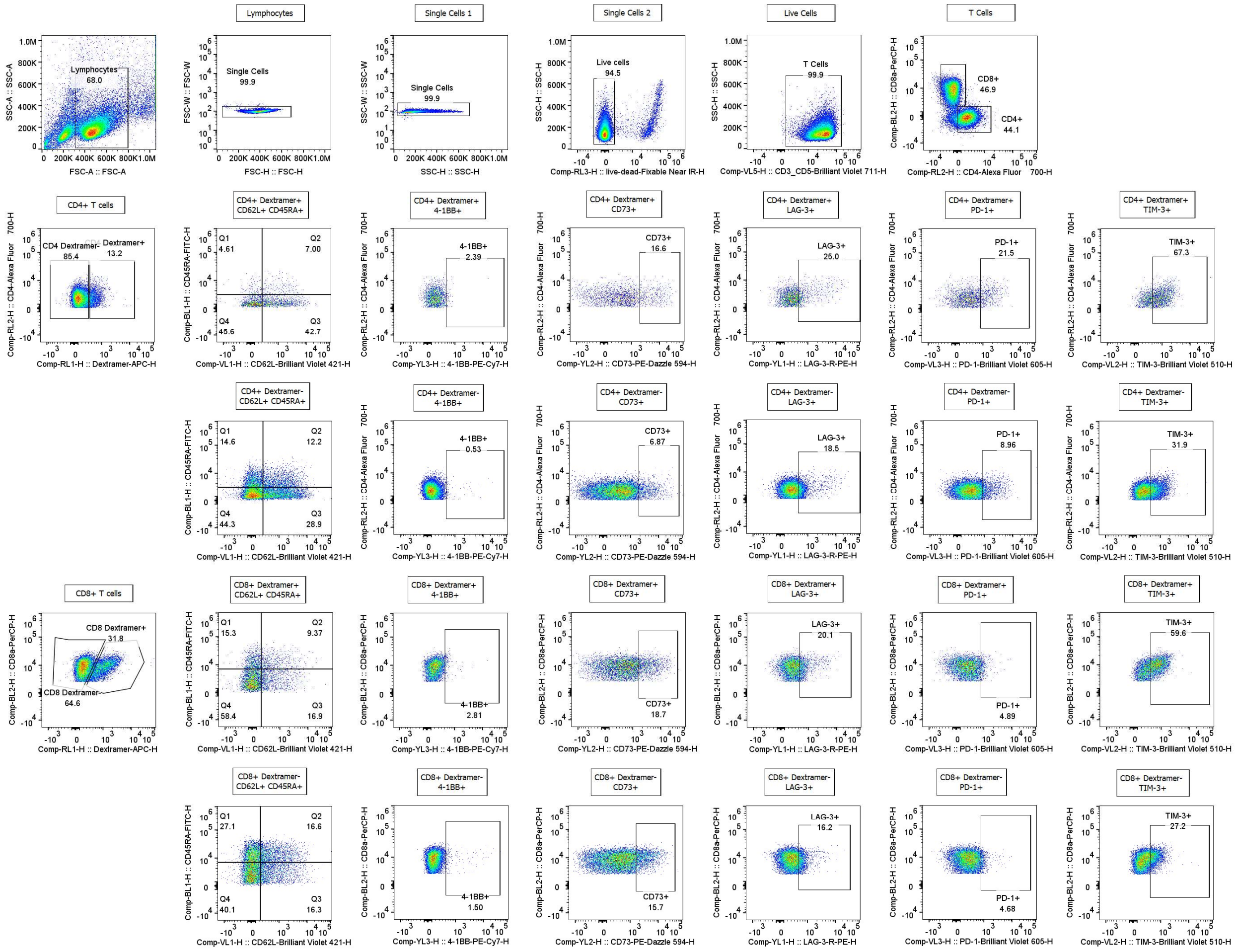

Supplementary Information Figure 7. Post- infusion phenotype characterization gating strategy.

Cells are gated for size, singlets, and viability. T cells are identified by staining for CD5 and/or CD3. T cells are separated into CD4 T cells (rows 2 and 3) and CD8 T cells (rows 4 and 5). Cells are then gated for expression of peptide-HLA multimer (dextramer-APC), with dextramer+ cells shown on the top rows and dextramer- cells shown on the bottom rows. Cells are further gated on CD62L and CD45RA. CD62L+ CD45RA+ cells are designated "naïve-like" and are then gated on CD95+CD27+ (Tmsc, memory stem cells) and CD95-CD27+ (Tn, naïve), and CD95+CD27- (other). CD62L- CD45RA+ cells are designated Teff (effector). CD62L+ CD45RA- cells are designated Tcm (central memory). CD45RA- CD62L- cells (DN) are further gated as CD27+ (Ttm, transitional memory) and CD27- (Tem, effector memory). Additionally, CD4 and CD8 dextramer+ and dextramer- cells are gated on CD39 and CD103 (far right). Used for Extended Data Figure 4b, Extended Data Figure 5c.

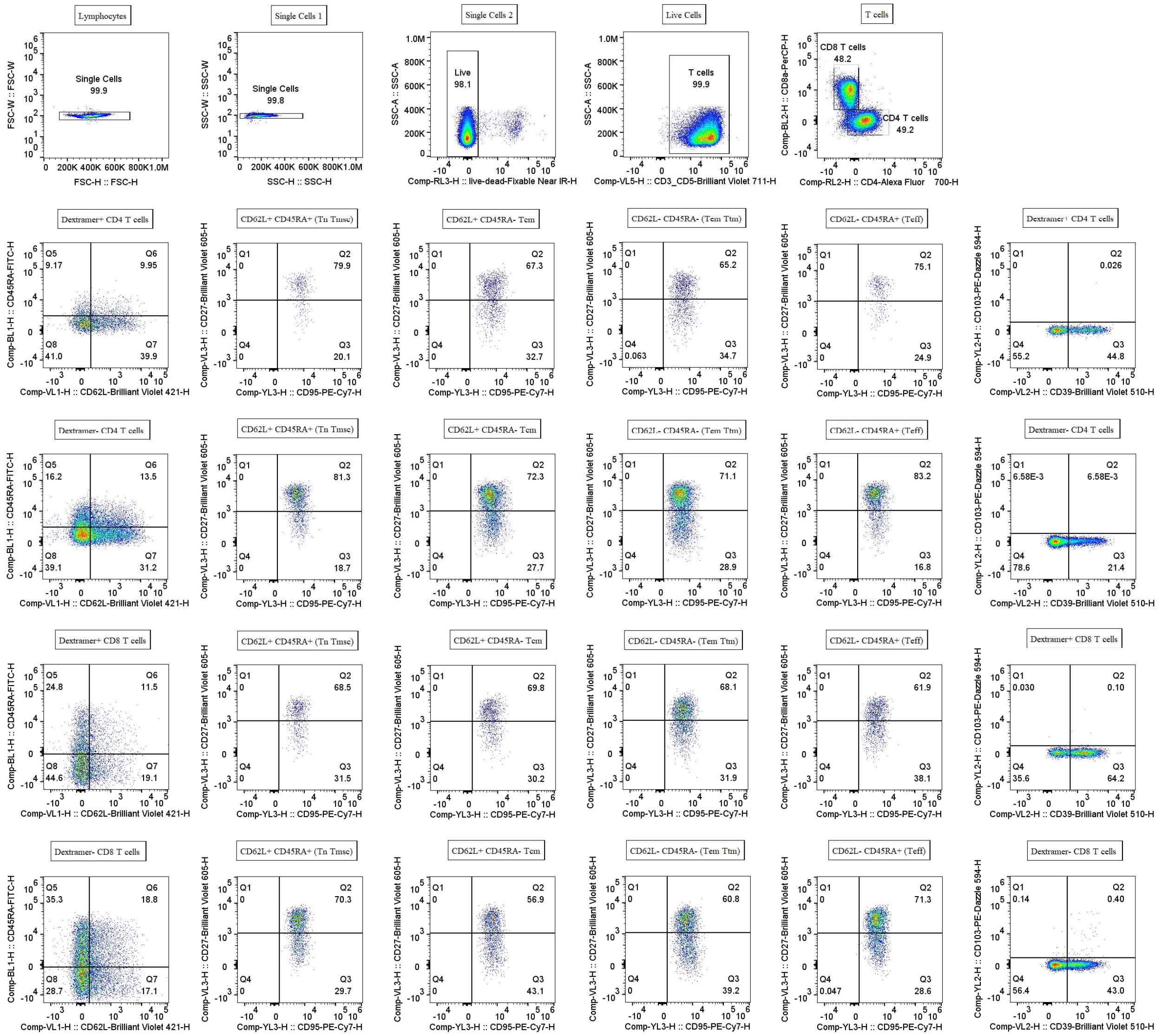

Supplementary Information Figure 8. Post-infusion activation characterization gating strategy.

Cells are gated for size, singlets, and viability. T cells are identified by staining for CD5 and/or CD3. T cells are separated into CD4 T cells (rows 2 and 3) and CD8 T cells (rows 4 and 5). Cells are then gated for expression of peptide-HLA multimer (dextramer-APC), with dextramer+ cells shown on the top rows and dextramer- cells shown on the bottom rows. Cells are further gated for expression of CD62L and CD45RA, 4-1BB, CD73, LAG-3, PD-1, and TIM-3. Used for Extended Data Figure 3b, Extended Data Figure 5b.

Supplementary Information Tables

Supplementary Information Table 1: Specific alleles including in the PACT-64 HLA panel and their prevalence in The Cancer Genome Atlas (TCGA) dataset.

HLA Allele	TCGA Prevalence
HLA-A02:01	41.40%
HLA-A01:01	23.65%
HLA-A03:01	22.71%
HLA-A24:02	16.85%
HLA-A11:01	12.11%
HLA-A32:01	6.20%
HLA-A26:01	6.06%
HLA-A68:01	5.90%
HLA-A23:01	5.56%
HLA-A29:02	5.56%
HLA-A31:01	5.09%
HLA-A25:01	4.14%
HLA-A30:01	4.12%
HLA-A30:02	2.96%
HLA-A68:02	2.68%
HLA-A33:01	1.93%
HLA-B07:02	20.36%
HLA-B08:01	16.24%
HLA-B35:01	11.26%
HLA-B51:01	9.93%
HLA-B15:01	9.41%
HLA-B18:01	8.96%
HLA-B44:03	8.84%
HLA-B40:01	8.43%
HLA-B27:05	5.68%
HLA-B57:01	5.26%
HLA-B14:02	5.06%
HLA-B13:02	4.81%
HLA-B38:01	4.34%
HLA-B35:03	3.50%
HLA-B49:01	3.50%
HLA-B52:01	3.11%
HLA-B58:01	2.78%
HLA-B53:01	2.76%
HLA-B55:01	2.76%

HLA Allele	TCGA Prevalence
HLA-B40:02	2.65%
HLA-B37:01	2.42%
HLA-B39:01	2.39%
HLA-B50:01	2.07%
HLA-B15:03	1.56%
HLA-B46:01	1.17%
HLA-B42:01	1.08%
HLA-B27:02	0.94%
HLA-B41:02	0.86%
HLA-B44:05	0.53%
HLA-B15:07	0.09%
HLA-C07:01	25.91%
HLA-C07:02	23.65%
HLA-C04:01	22.67%
HLA-C06:02	16.33%
HLA-C05:01	12.95%
HLA-C03:04	12.85%
HLA-C12:03	10.34%
HLA-C03:03	8.59%
HLA-C01:02	8.01%
HLA-C02:02	7.48%
HLA-C16:01	6.77%
HLA-C08:02	6.72%
HLA-C15:02	4.43%
HLA-C14:02	3.25%
HLA-C07:04	3.14%
HLA-C17:01	3.13%
HLA-C12:02	2.77%
HLA-C08:01	1.59%

Supplementary Information Table 2. **(separate file).** Full length TCR sequences for the 37 TCRs infused into patients.

Supplementary Information Table 3. Clinical Cell Manufacturing Process Versions.

Process Version	Cell Seeding Density (x10^6) per TCR	Electroporation Device	Media	Media Exchange (Day)	Changes from Previous Version
2.0	715	Lonza Nucleofector	TexMACS	6, 8	Cell Culture in G-rex
2.1	715	Lonza Nucleofector	Prime-XV	6, 8	Switch Media
3.0	715	CTS Xenon Electroporation System	Prime-XV	6, 8	Change Electroporation Device

Supplementary Information Table 4. (separate file). List of flow cytometry reagents.

Supplementary Information: Clinical Trial Protocol

Protocol Title: A PHASE 1A/1B, OPEN-LABEL FIRST-IN-HUMAN STUDY OF THE SAFETY, TOLERABILITY, AND FEASIBILITY OF GENE-EDITED AUTOLOGOUS NeoTCR T CELLS (NeoTCR-P1) ADMINISTERED AS A SINGLE AGENT OR IN COMBINATION WITH ANTI-PD-1 TO PATIENTS WITH LOCALLY ADVANCED OR METASTATIC SOLID TUMORS

Protocol Number: PACT-0101

Title Page

Protocol Title: A PHASE 1A/1B, OPEN-LABEL FIRST-IN-HUMAN STUDY OF THE SAFETY, TOLERABILITY, AND FEASIBILITY OF GENE-EDITED AUTOLOGOUS NeoTCR T CELLS (NeoTCR-P1) ADMINISTERED AS A SINGLE AGENT OR IN COMBINATION WITH ANTI-PD-1 TO PATIENTS WITH LOCALLY ADVANCED OR METASTATIC SOLID TUMORS

Protocol Number: PACT-0101

Amendment Number: Version 6

Compound: NeoTCR-P1

Study Phase: Phase 1

Short Title: A Phase 1 study of gene-edited autologous neoantigen-targeted TCR-T cells with or without anti–PD-1 in patients with solid tumors

Sponsor Name: PACT Pharma, Inc.
Legal Registered Address: 2 Corporate Drive, 2nd Floor, South San Francisco, CA 94080

Regulatory Agency Identifier Number(s)
IND: 18801

Approval Date: 22 December 2020

[redacted]

[redacted]

[redacted]

[redacted]

Amendment Rationale (Version 6)

Study PACT-0101 protocol was amended to increase the intensity of the conditioning chemotherapy regimen. Whereas previously the regimen consisted of 3 daily doses of fludarabine 30 mg/m^2 (total dose 162 mg assuming 1.8 m^2 BSA) and cyclophosphamide 300 mg/m^2 (total dose ~1620 mg), the updated regimen will consist of 4 daily doses of fludarabine 30 mg/m^2 (total dose ~216 mg) and 3 daily doses of cyclophosphamide 600 mg/m^2 (total dose ~3240 mg). This change was made following a review of data from the first 6 treated participants which suggested that adequate lymphodepletion was not achieved with the original regimen. The increased regimen is less intense than the standard NCI solid tumor preparative regimen (total dose of fludarabine ~225 mg and cyclophosphamide ~9600 mg) but is within the range of those used in other cell therapy studies where clinical activity was observed.

This amendment also adds the option to increase the adjunctive IL-2 regimen following review of the safety and pharmacokinetics of the low-dose regimen that will be administered to the first 6 participants in the IL-2 expansion cohort. Despite the rationale for the low-dose regimen, data from this study and emerging data external to this study may suggest that a conventional high-dose regimen is superior in supporting the persistence and expansion of NeoTCR T cells after infusion. The ability to switch to a high-dose regimen following a predetermined interim analysis improves the efficiency of testing a potentially more effective alternative regimen in subsequent participants.

The following changes were made to the protocol:

- Throughout the text, “low-dose” in reference to IL-2 was removed.
- Throughout the text, an additional visit (i.e., Day -6) was added to the start of the Conditioning Chemotherapy Period.
- Throughout the text, the conditioning chemotherapy regimen was extended to 4 days instead of 3 days and the cyclophosphamide dose revised to 600 mg/m^2/day instead of 300 mg/m^2/day.
- A Day -6 column was added to the Schedule of Activities and the biopsy timing was changed from Day -5 to Day -6 (Section 1.4.2).
- Table 11 was updated with additional information from Robbins et al. (2011) and Doran et al. (2019) (Section 2.10).
- The review of the IL-2 basket expansion cohort was clarified to include the safety and pharmaceutical data; in addition, it was clarified that the SRT may recommend evaluation of a more intensive IL-2 regimen based upon this review (Section 2.10).
- Parameters for allowing the SRT to recommend the evaluation of a more intense regimen of IL-2 was added in Section 4.1 under “Phase 1a Expansion (u3TCR).”
- The rationale for the conditioning chemotherapy was revised (Section 4.2.10).
- In the inclusion criteria, it was clarified that patients with a creatinine clearance <60 mL/min may not receive IL-2 as part of their experimental treatment, and such patients will be assigned to receive NeoTCR-P1 ± nivolumab (Sections 5.1 and 5.3).

- The cohort dosing for IL-2 was changed to "…will receive IL-2 at the prescribed dose per protocol" from "…will receive IL-2 at 500,000 IU/m^2 SC twice daily (BID)" Section 6.1.3.6.2).
- The administration of nivolumab was clarified (Section 6.1.4.2).
- A table listing clinical management recommendations for high-dose IL-2 therapy was added (Table 18, Section 6.7.3).
- The windows for the conditioning chemotherapy were added for clarification. In addition, it was clarified that cell product should be available before the start of the conditioning chemotherapy (Section 8.5.6).
- Text concerning the administration of NeoTCR-P1 without conditioning chemotherapy was removed (Section 8.5.7).

Additional minor clarifications and administrative changes were made to improve the organization and clarity of the protocol. Revisions are shown in *Book Antiqua* italics. This amendment represents cumulative changes to the original protocol.

Amendment Rationale (Version 5)

(Note: Since Version 5 was submitted only to the FDA, the Amendment Rationale and revisions shown in Book Antiqua for Version 5 are also included in Version 6 of the protocol for the convenience of IRBs and sites.)

Study PACT-0101 was amended to include a more rigorous eligibility assessment prior to the start of the T-cell receptor (TCR) identification process. The rationale is to minimize participant drop-out at leukapheresis due to irreversible disqualifying laboratory abnormalities or medical conditions that can be identified earlier.

The independent data monitoring committee (IDMC) was removed, and IDMC responsibilities were subsumed under those of the Safety Review Team (SRT). This conforms with Food and Drug Administration (FDA) guidance and is in alignment with the general practice of not convening an IDMC until more advanced testing begins (e.g., Phase 2 and beyond). The SRT, in collaboration with investigators, will be responsible for safety monitoring during the dose-escalation phase and for making decisions to open expansion cohorts and initiate combination testing.

Enrollment into the Phase 1b portion may begin after preliminary safety and optimal dosing of single-agent NeoTCR-P1 is reviewed by the SRT after clearance of at least 1 dose level and at least 12 participants in the Initial Phase have had the opportunity to be followed until their first tumor assessment. Previously, opening of the Phase 1b portion was gated by a minimum of 10 participants in the basket expansion cohorts who had the opportunity to be followed until the first tumor assessment. This change was made to enable potentially earlier evaluation of the combination with nivolumab, if warranted by data from the Initial Phase and/or supportive data external to the study.

The first dose of nivolumab in the Phase 1b portion was moved from Day 0 (i.e., same day as NeoTCR-P1) to Day 1 (i.e., 1 day after administration of NeoTCR-P1) in order to minimize the possibility of confounded attribution of adverse events related to product infusion.

In addition, the Phase 1b portion may include testing of NeoTCR-P1 with interleukin (IL)-2 plus nivolumab, if warranted by data from the Initial Phase and/or supportive data external to the study.

The INR/aPTT assessment was removed as an eligibility criterion with the rationale that a history of symptomatic deep venous thrombosis or pulmonary embolism requiring anticoagulation within 6 months of enrollment is already exclusionary, and that adequate coagulation function is also ensured by surrogate measures, such as liver function tests.

The term “patient” was changed to “participant” in sections referring to procedures that involve enrollees in the study for internal consistency and consistency with other protocols.

The following changes were made to the protocol:

- The option to perform small-volume leukapheresis was added for participants in whom PBMCs from whole blood are not adequate for imPACT analysis (Sections 1.4.1 and 8.5.1.1).
- References to the “IDMC” were replaced with “SRT” or deleted, as appropriate (Sections 1, 2.1, 4.1, 4.2, 6.1, and 8.16). Section 8.17 titled “Independent Data Monitoring Committee” was removed. The role of the SRT was clarified (Section 4.1.4).

- The Expansion Phase was revised to remove NeoTCR-P1 dose escalation in the Phase 1b portion (Sections 1 and 4.1). Instead, combination dosing with nivolumab may commence at the highest NeoTCR-P1 cell dose cleared in the Phase 1a portion. The rationale for this change is accumulating evidence that tumor infiltrating lymphocytes and engineered T cells can be safely administered with PD-1 inhibitors, decreasing the risk that unexpected toxicities will be encountered in this study (e.g., Zhou et al., 2020; Heczey et al., 2017; Jacobson et al., 2020). Continued enrollment of the Phase 1b portion will be gated by an SRT review of safety data from the first 6 participants treated with NeoTCR-P1 + nivolumab.
- The Phase 1b portion may include testing of NeoTCR-P1with IL-2 plus nivolumab, if warranted by data from the Initial Phase and/or supportive data external to the study (Section 4.1). The addition of IL-2 will be gated by the establishment of a safe and tolerable NeoTCR-P1 + nivolumab regimen (see schema, Section 1.3), as well as SRT review of safety data from a minimum of 6 participants treated in the NeoTCR-P1 + IL-2 basket expansion cohort in the Initial Phase. Full enrollment of a 10–20 participant NeoTCR-P1 and IL-2 plus nivolumab basket cohort will be contingent on SRT review of safety data from the first 6 participants. The risk of synergistic toxicity is expected to be low, and participants will be closely monitored for expected adverse events for each agent described in the protocol. A recent study of pembrolizumab + high-dose IL-2 (~150 million units per day) in patients with metastatic clear cell renal cell carcinoma (n=18; Chatzkel et al., 2019) concluded that the combination was feasible, with no accrual stop for safety.
- As a result of the addition of the optional nivolumab + IL-2 cohort and other changes to the Phase 1b portion of the study, the maximum possible number of participants in the study was increased to 188 (Sections 1.1 and 4.1, Table 14).
- A column was added to the Schedule of Activities (SOA) to represent the waiting period for the TCR identification and product selection. Since this addition expanded the SOA beyond the marginal limits of the page, the SOA was divided into 2 tables: “Screening Part 1 Activities” and “Screening Part 2 and Main Study Activities” (Section 1.4).
- Minor edits were made to clarify the collection times listed in Table 1 (Section 1.4).
- The assessment “Coagulation (PT/PTT)” was removed (Table 3, Sections 1.4 and 8.4.7.1).
- Figure 2 was updated to more clearly represent the NeoTCR-P1 process (Section 2.5).
- Table 12 (Risk Assessment) was revised to add information to the mitigation strategy for the risk “Neurotoxicity” (Section 2.11).
- The number of possible sites participating in this study was increased from 6 to 12 (Section 4.1.1).
- The timing of tumor biopsies was clarified (Sections 4.2.8 and 8.5.8).
- The ability to use specimens, including but not limited to fluid from ascites and pleural effusions and left-over tissue from standard-of-care or palliative procedures, for exploratory analyses with participant consent was added (Section 4.2.9).
- The screening procedures were divided into two parts, including a preliminary eligibility assessment and submission of tumor tissue and whole blood during Screening Part 1, and a re-evaluation prior to leukapheresis if TCR selection is successful (Screening Part 2; Sections

5, 8.5.1, and 8.5.3). Select criteria pertaining to irreversible disqualifying conditions (e.g., pericardial effusion and cardiac involvement of primary malignancy) were moved up to Screening Part 1. Criteria pertaining to washout from prior therapy and defining required prior therapies per indication were retained in Screening Part 2, because they do not apply until actionable TCRs have been identified and the participant is to be scheduled for leukapheresis and NeoTCR-P1 dosing.

- Revisions were made to the following criteria for Screening Part 2: timing of inclusions and exclusions changed from prior to "enrollment" to prior to "leukapheresis"; parameters for absolute neutrophil count (ANC), platelet count, creatinine clearance criterion were relaxed and/or clarified to accommodate potential changes in clinical status during the TCR identification and product selection waiting period (Sections 5.3 and 5.4).
- The packaging for NeoTCR-P1 was revised from "CryoMacs Freezing Bag 250 with 35 mL of product" to "single-use bags" (Section 6.1.2).
- The Section 6.1.3 title was revised to clarify that the section presents baseline procedures. In addition, text was added to clarify the timing of procedures.
- Examples of immunosuppressive drugs were added for clarity (Section 6.6).
- Clarification was added that MRIs are to be conducted with contrast whenever possible or without contrast in case of contraindication (Section 8.4.3).
- Minimum tumor sample requirements were revised for clarity (Section 8.5.1.1.1).
- Text was added to clarify the timing of imaging prior to conditioning chemotherapy (Section 8.5.5).
- A diary was added for patients to record their daily temperature after infusion of NeoTCR-P1 (Section 8.5.7).
- The IDMC interim safety analysis was removed (Section 8.16.2), consistent with the removal of the IDMC and transfer of IDMC responsibilities to the Safety Review Team (SRT)
- The patients excluded and potentially eligible based upon their history of preexisting autoimmune disease was clarified (Section 9.2)
- The list of preexisting autoimmune diseases in Appendix 2 was updated.

Additional minor clarifications and administrative changes were made to improve the organization and clarity of the protocol. Revisions are shown in *Book Antiqua* italics. This amendment represents cumulative changes to the original protocol.

PRINCIPAL INVESTIGATOR AGREEMENT AND SIGNATURE PAGE

Printed Name of Investigator:
Printed Institution Name & Address:
I have reviewed this protocol (including Appendices) and agree:

- To assume responsibility for the proper conduct of the study at this site;
- To conduct the study in compliance with this protocol, with any future amendments, and with any other study conduct procedures provided by PACT Pharma or designee. I also agree to comply with current good clinical practices (cGCPs) and all regulatory requirements;
- Not to implement any changes to the protocol without agreement from PACT Pharma or designee and prior review and written approval from the Institutional Review Board (IRB) except where it is necessary to eliminate an immediate hazard to the subjects or for administrative aspects of the study (where permitted by all applicable regulatory requirements);
- That I am thoroughly familiar with the appropriate use of the investigational and approved product(s), as described in this protocol, and with any other relevant information (e.g., the Investigator's Brochure);
- To ensure that all persons assisting me with the conduct of this study are adequately informed about the investigational product(s) and about their study-related duties and functions as described in this protocol;
- That I am aware that certain regulatory authorities require Investigators to disclose all information about ownership interests and financial ties related to the Sponsor and/or the investigational product(s). Consequently, I agree to supply all such information to PACT Pharma, and to promptly update this information if any relevant changes occur during the course of the study and for 1 year following completion of the study. I also agree that PACT Pharma may disclose any information it has regarding ownership interests and financial ties of Investigators to regulatory authorities.

________________________________	____________________
Signature of Investigator	Date

Table of Contents

1. Protocol Summary 16
1.1. Synopsis 16
1.2. Participant-Level Study Flow in PACT-0101 20
1.3. Schema 21
1.4. Schedule of Activities 22
1.4.1. *Screening Part 1 Activities* 22
1.4.2. *Screening Part 2 and Main Study Activities* 23

2. Introduction 28
2.1. Study Rationale 28
2.2. Background 30
2.2.1. Melanoma 31
2.2.2. Urothelial Carcinoma 31
2.2.3. Head and Neck Squamous Cell Cancer 32
2.2.4. Non-Small Cell Lung Cancer 33
2.2.5. Colorectal Cancer 34
2.2.6. Ovarian Cancer 35
2.2.7. Hormone-Receptor Positive Breast Cancer 36
2.2.8. Triple Negative Breast Cancer 37
2.2.9. Prostate Cancer 37
2.3. Engineered TCR-T Cells for Advanced Malignancies 38
2.4. Neoantigens As Targets for T Cells 41
2.5. NeoTCR-P1 T-Cell Product 42
2.6. Summary of Nonclinical Studies Supporting NeoTCR-P1 47
2.7. Nivolumab 51
2.8. Adoptive T-Cell Therapy (ACT) in Combination with Checkpoint Blockade 52
2.9. Interleukin-2 (Aldesleukin Recombinant Interleukin 2) 53
2.10. Interleukin-2 as Adjunctive Therapy with ACT 53
2.11. Benefit/Risk Assessment 55
2.11.1. Potential for Overlapping Toxicities 63
2.11.2. Benefit Assessment 63
2.11.3. Overall Benefit/Risk Conclusion 63

3. Objectives and Endpoints 64

4. Study Design 65
4.1. Overall Design 65
4.1.1. Participating Sites 75
4.1.2. Number of Participants 75
4.1.3. Replacement of Participants 76
4.1.4. Safety Review Team 76
4.1.5. Definition of Dose-Limiting Toxicity and DLT Rules 76
4.2. Scientific Rationale for Study Design 79
4.2.1. Rationale for Patient Population 79
4.2.2. Rationale for Study Design and Endpoints 80
4.2.3. Rationale for Evaluating Polyclonal TCR Products 80

4.2.4. Rationale for Evaluating NeoTCR-P1 in Combination with Nivolumab....81
4.2.5. Rationale for Dose of Nivolumab....82
4.2.6. Rationale for IL-2 Regimen....82
4.2.7. Rationale for the DLT Criteria and Assessment Window....83
4.2.8. Rationale for Collection of Baseline and On-Treatment Biopsy....83
4.2.9. Rationale for Collecting Tumor Specimens at the Time of Progression....84
4.2.10. Rationale for Conditioning Chemotherapy....84
4.2.11. Justification for Starting Dose of NeoTCR-P1....85
4.3. Rationale for Flat-Fixed Dosing Regimen....87
4.4. End of Study and Length of Study....88

5. Study Population....88
5.1. *Screening Part 1* Inclusion Criteria....89
5.2. Screening Part 1 Exclusion Criteria....90
5.3. *Screening Part 2 Inclusion Criteria*....92
5.4. *Screening Part 2* Exclusion Criteria....97
5.5. Screen Failures....98

6. Study Intervention Terminology....98
6.1. Study Intervention(s) Administered....99
6.1.1. Conditioning Chemotherapy....99
6.1.2. NeoTCR-P1....99
6.1.3. *Baseline* Evaluation *Prior To* Conditioning Chemotherapy and NeoTCR-P1 T Cell *Infusion*....100
6.1.4. Nivolumab....102
6.2. Treatment Assignment....102
6.3. IP Accountability and Study Intervention Compliance....103
6.4. Concomitant Therapy....103
6.5. Bridging Therapy....104
6.6. Excluded Medications....104
6.7. Dose Modification....105
6.7.1. NeoTCR-P1....105
6.7.2. Nivolumab....105
6.7.3. IL-2....106
6.7.4. Rescue Medications....111
6.7.5. Retreatment Criteria....111
6.8. Subsequent Therapy....111
6.9. Intervention After the End of the Study....111
6.10. Study Treatment Schedule....111
6.10.1. Leukapheresis....111
6.10.2. Study Treatment....112
6.11. Toxicity Management....112
6.11.1. Acute Infusion Reaction....112
6.11.2. Febrile Reaction....112
6.11.3. Cytokine Release Syndrome....112

6.11.4. Hypotension and Renal Toxicity 117
6.11.5. Cardiac Toxicity....................117
6.11.6. Hemophagocytic Lymphohistiocytosis....................118
6.11.7. Neurotoxicity 119
6.11.8. Tumor Lysis Syndrome....................124
6.11.9. Uncontrolled T-cell Proliferation....................124
6.11.10. Graft-Versus-Host Disease/Autoimmunity....................124
6.11.11. Capillary Leak Syndrome 125
6.11.12. Fever and Neutropenia....................125
6.11.13. Infection Prophylaxis....................125
6.11.14. Blood Product Support....................126

7. Discontinuation of Study Intervention and Participant Discontinuation/Withdrawal....................126
7.1. Discontinuation of Study Intervention....................126
7.2. Participant Discontinuation/Withdrawal from the Study....................127
7.3. Post-Study Access....................128
7.4. Lost to Follow Up 128

8. Study Assessments and Procedures....................128
8.1. Informed Consent....................129
8.2. Screening Assessments 129
8.2.1. Medical and Treatment History, Concomitant Medications, and Demographic Information....................129
8.2.2. Physical Examination....................129
8.2.3. Vital Signs....................130
8.3. Efficacy Assessments....................130
8.3.1. Tumor and Response Evaluations....................130
8.3.2. *Participants* with Prostate Cancer 131
8.4. Safety Assessments....................132
8.4.1. Cardiac Function....................132
8.4.2. Neurological Assessment....................132
8.4.3. MRI/CT Brain....................132
8.4.4. Electrocardiograms 132
8.4.5. Bone Marrow Biopsy....................133
8.4.6. Lumbar Puncture....................133
8.4.7. Clinical Laboratory Assessments....................133
8.4.8. Leukapheresis 134
8.5. Description of Study Periods 135
8.5.1. Screening....................135
8.5.2. Rescreening....................137
8.5.3. Enrollment/Leukapheresis 137
8.5.4. Baseline Biopsy 138
8.5.5. Re-evaluation Prior to Conditioning Chemotherapy 139
8.5.6. Conditioning Chemotherapy....................139
8.5.7. Inpatient IP Administration....................140
8.5.8. Post Treatment Assessment Period....................141
8.5.9. Long-Term Follow-Up Period....................142

8.5.10. Cross-Over 143
8.5.11. Retreatment 143
8.6. Adverse Events and Serious Adverse Events 144
8.6.1. Time Period and Frequency for Collecting AE and SAE Information 144
8.6.2. Method of Detecting AEs and SAEs 145
8.6.3. Follow-up of AEs and SAEs 145
8.6.4. Regulatory Reporting Requirements for SAEs 146
8.6.5. Pregnancy 147
8.6.6. Death Events 147
8.6.7. Adverse Events of Special Interest 148
8.7. Pharmacokinetics 148
8.8. Pharmacodynamics 148
8.9. Genetics 148
8.10. Biomarkers 149
8.11. Immunogenicity Assessments 150
8.12. Statistical Considerations and Hypotheses 150
8.13. Sample Size Determination 150
8.14. Populations for Analyses 152
8.15. Statistical Analyses 153
8.15.1. General Considerations 153
8.15.2. Primary Endpoint(s) 153
8.15.3. Secondary Endpoint(s) 153
8.15.4. Exploratory Endpoint(s) 154
8.16. Interim Analyses 155
8.16.1. Interim Analysis and Early Stopping Rules 155
8.16.2. Efficacy Interim Analyses 155
8.17. Criteria to Pause Enrollment 155
8.18. Study Sample Storage and Retention 156

9. Supporting Documentation and Operational Considerations 157
9.1. Appendix 1. Regulatory, Ethical, and Study Oversight Considerations 157
9.1.1. Regulatory and Ethical Considerations 157
9.1.2. Financial Disclosure 157
9.1.3. Informed Consent Process 157
9.1.4. Data Protection 158
9.1.5. Administrative Structure 158
9.1.6. Dissemination of Clinical Study Data 159
9.1.7. Data Quality Assurance 159
9.1.8. Source Documents 160
9.1.9. Study and Site Start and Closure 161
9.2. Appendix 2. Preexisting Autoimmune Diseases 161
9.3. Appendix 3. Adverse Events. Definitions and Procedures for Recording, Evaluating, Follow-up, and Reporting 164
9.3.1. Definition of *Adverse Event* (AE) 164
9.3.2. Definition of SAE 165

9.3.3. Recording and Follow-Up of AE and/or SAE 166
9.3.4. Reporting of SAEs and Adverse Events of Special Interest (AESI)s 168
9.4. Appendix 4. Contraceptive Guidance and Collection of Pregnancy Information 168
9.5. Appendix 5. Genetics 170
9.6. Appendix 6. Response Evaluation Criteria in Solid Tumors. Modified Excerpt from Original Publication 170
9.7. Appendix 7. ECOG Performance Status 179
9.8. Appendix 8. Abbreviations 180
10. References 184

Table of Tables

Table 1 Collection Times for Fludarabine Pharmacokinetic and Pharmacogenetic Samples 26
Table 2 Windows for Re-evaluation Prior to Conditioning Chemotherapy 26
Table 3 Frequency of Assessments during Inpatient Hospitalization for IP Administration Period 27
Table 4 Comparison of CARs, TCRs, and TILs 30
Table 5 Summary of PD-1/PD-L1 Activity in Patients with Metastatic or Locally Advanced Urothelial Carcinoma 32
Table 6 Summary of Outcomes for Second-Line Treatment of Metastatic Colorectal Cancer 35
Table 7 Outcomes for Relapsed Epithelial Ovarian Cancer in Platinum-Resistant Disease 36
Table 8 Outcomes for Second and Subsequent-Line Treatment of Metastatic Breast Cancer 37
Table 9 Outcomes for Second/Third-Line Therapy in Patients with CRPC 38
Table 10 Clinical Studies with Gene-Edited TCRs 39
Table 11 *Cell* Therapy with IL-2 54
Table 12 Risk Assessment 58
Table 13 Cell Dose as a Function of the Number of Administered NeoTCRs 69
Table 14 Gates for Opening Phase 1b Cohorts (Expansion Phase) 72
Table 15 Minimum and Maximum Numbers of Participants 75
Table 16 TCR-Target and Engineering 86
Table 17 Conditioning Chemotherapies 99

Table 18 Clinical Management Recommendations for High Dose IL-2 Therapy 107

Table 19 Clinical Signs and Symptoms Associated with Cytokine Release Syndrome 113

Table 20 CRS Reported in Recent Clinical Trials of CD19 CAR-T 113

Table 21 Cytokine Release Syndrome Consensus Grading Scale (ASTCT)....114

Table 22 Cytokine Release Syndrome Management Guidelines....115

Table 23 Neurotoxicity/ICANS Consensus Grading Scale (ASTCT)....120

Table 24 Encephalopathy Assessment Tool for Grading Neurotoxicity/ICANS 121

Table 25 Neurotoxicity Management Guidelines 122

Table 26 Cerebral Edema Management Guidelines 123

Table 27 Vital Sign Measurement at Day 0....130

Table 28 Tumor Assessments per Indication....131

Table 29 Summary of AE Collection Periods....145

Table 30 Probability of Observing DLTs for Different Underlying DLT Rates 151

Table 31 Definitions of Populations for Analysis....152

Table of Figures

Figure 1 Neoantigens: Optimal Targets for T Cell-Tumor Immunity....41

Figure 2 NeoTCR-P1 Process 43

Figure 3 NeoTCR-P1 Product Selection Process....45

Figure 4 Summary of Isolated Neoepitope-Specific T cell and Cognate neoTCRs....46

Figure 5 Specific Killing of Antigen-Expressing Surrogate Tumor Target Cells and Antigen-Specific Proliferation of NeoTCR-P1 T Cells....48

Figure 6 Antigen-Specific Activity of Precision Genome-Engineered Human NeoTCR T Cells – Comparable Functional Activity of T Cells derived from Patients with Cancer or Healthy Donors....49

Figure 7 Single Cell Analysis of NeoTCR-P1 T Cells Reveals Polyfunctional Phenotype....50

Figure 8 NeoTCR-P1 T Cells are Mainly of the "Younger" Tscm and Tcm Phenotype....51

Figure 9 Phase 1a/Phase 1b Design....66

Figure 10 Phase 1a Initial Phase....68

Figure 11 Schema for Opening Dose Expansion Cohorts....70

Figure 12 Study Design....74

Figure 13 Phase 1 DLT Evaluation Schema (Alternative).....78
Figure 14 Tumor Mutational Load Across Landscape of Cancer Indications.....79
Figure 15 Screening Part 1.....136
Figure 16 Cessation of Therapies Prior to Leukapheresis.....138

1. Protocol Summary

1.1. Synopsis

Protocol Title: A Phase 1a/1b, Open-Label First-in-Human Study of The Safety, Tolerability, and Feasibility of Gene-Edited Autologous NeoTCR T Cells (NeoTCR-P1) Administered as a Single Agent or in Combination with Anti–PD-1 to Patients with Locally Advanced or Metastatic Solid Tumors

Short Title: A Phase 1 study of gene-edited autologous neoantigen-targeted TCR-T cells with or without anti–PD-1 in patients with solid tumors

Rationale: Adoptive transfer of gene-edited neoepitope-specific T-cell receptor (neoTCR) T cells targeting neoantigens is an immunotherapy modality designed to unleash the immune system's ability to specifically recognize tumor-exclusive mutational targets in each participant. Since all cancers are driven by underlying founder or truncal mutations, adoptive neoTCR T-cell therapy targeting neoepitopes holds the potential for treatment of any patient with cancer in whom a neoTCR can be identified. NeoTCR-P1 adoptive cell therapy involves engineering an individual's own CD8 and CD4 T cells to express naturally occurring neoTCRs that already recognize tumor-specific antigens. These neoTCRs of native sequence are identified and authenticated from a small sample of blood derived from each participant from pre-existing mutation-targeted CD8 T cells. In a subsequent step, freshly derived CD4 and CD8 T cells of the same patient are genetically engineered to express neoTCRs in a manner that reconstitutes fully "native" autologous T-cell function. The potential for significant clinical benefit to participants with solid tumors, thus, stems from delivering a single dose of *ex vivo* engineered tumor mutation-targeted autologous NeoTCR-P1 cells. Novel therapies for patients with relapsed or refractory metastatic solid tumors are urgently needed. The administration of NeoTCR-P1 cells combines the selection of authenticated personalized tumor-exclusive targets for each participant (i.e., neoantigen) with the potency of T-cell therapies, thus providing the potential to trigger rapid and durable responses in patients who have no curative treatment options. This first-in-human clinical trial will assess the safety and feasibility of NeoTCR-P1 as a single agent, with adjunctive interleukin-2 (IL-2), *and* in combination with nivolumab in patients with relapsed/refractory solid tumors as measured by the adverse event rate and percentage of screened patients for whom product can be manufactured. If antitumor activity is observed and the administration of NeoTCR-P1 is tolerable and feasible in the Initial Phase of the study, then the study may proceed to an Expansion Phase.

Objectives and Endpoints:

Objectives	Endpoints
**Primary**	
Safety	
• To evaluate the safety and tolerability of a single dose of NeoTCR-P1 administered as a single agent without or with IL-2 (Phase 1a), *and* in combination with nivolumab *without or with IL-2* (Phase 1b) in *participants* with locally advanced or metastatic tumors<br>• To determine the maximum tolerated dose (MTD) of NeoTCR-P1<br>• To identify a recommended Phase 2 dose (RP2D) of NeoTCR-P1	• Incidence and nature of DLTs<br>• Incidence, nature, and severity of adverse events graded according to NCI CTCAE v5.0 (ASTCT consensus grading criteria will be used for CRS and neurotoxicity)<br>• Incidence, nature and severity of adverse events of special interest (AESI) according to NCI CTCAE v5.0
**Feasibility**	
• To evaluate the feasibility of manufacturing NeoTCR-P1	• Percentage of screened *participants* in whom product selection is successful<br>• Percentage of screened *participants* who enroll on study<br>• Percentage of enrolled *participants* who receive NeoTCR-P1<br>• Percentage of manufactured products that meet release criteria for neoTCR-positive editing efficiency, T-cell viability, sterility, T-cell number<br>• Median time from leukapheresis to product delivery<br>• Median time from signing informed consent to infusion
**Secondary**	
**Pharmacokinetic/Pharmacodynamic**	
• To evaluate the pharmacokinetics of NeoTCR-P1 after administration as a single agent without or with IL-2 *and* in combination with nivolumab *without or with IL-2*	• Proliferation, engraftment, persistence, and trafficking of neoTCR T cells when administered as a single agent without or with IL-2 *and* in combination with nivolumab *without or with IL-2*<br>• Median values of C_{max} and AUC_{0-28d} of neoTCR-P1 cells administered as a single agent without or with IL-2 *and* in combination with nivolumab *without or with IL-2*<br>• Evaluation of neoTCR T-cell infiltration in biopsy specimens post-dosing
• To evaluate the pharmacodynamics of NeoTCR-P1 when administered as a single agent without or with IL-2 *and* in combination with nivolumab *without or with IL-2*	• Changes in blood cytokine levels following administration of NeoTCR-P1 as a single agent without or with IL-2 *and* in combination with nivolumab *without or with IL-2*
• To characterize the pharmacokinetics of fludarabine when administered as part of the conditioning chemotherapy regimen	• Fludarabine plasma and intracellular triphosphate concentrations

Objectives	Endpoints
**Immunogenicity (Phase 1b only)**	
• To evaluate the effect of NeoTCR-P1 administration on the immunogenicity of nivolumab	• Incidence of ADAs to nivolumab
**Activity**	
• Preliminary assessment of the anti-tumor activity of NeoTCR-P1 administered as a single agent, without or with IL-2 *and* in combination with nivolumab *without or with IL-2* in *participants* with locally advanced or metastatic solid tumors	• Objective response rate, defined as CR or PR per RECIST v1.1 as determined by the investigator<br>• Duration of response, defined as time from the first occurrence of a documented objective response to the time of relapse or death from any cause<br>• Progression-free survival (PFS), defined as the time from the first study treatment to the first occurrence of progression or death, whichever occurs first<br>• Overall survival (OS), defined as the time from the first study treatment to death
**Exploratory**	
• Preliminary assessment of biomarkers that might act as pharmacodynamic indicators of anti-tumor activity of NeoTCR-P1 when administered as a single agent without or with IL-2 *and* in combination with nivolumab *without or with IL-2*<br>• Preliminary assessment of biomarkers that might act as predictors of toxicity or anti-tumor activity of NeoTCR-P1 when administered as a single agent without or with IL-2 *and* in combination *with nivolumab* without or with *IL-2*<br>• Preliminary assessment of the relationship between fludarabine exposure and the pharmacokinetics and anti-tumor activity of NeoTCR-P1	• T-cell markers (e.g., CD39) of activation and homing to tumor<br>• Changes in T-cell functional markers and relative T-cell subsets (e.g., T_{scm}, T_{cm}, T_{em}, T_{eff}) of neoTCR-P1 T cells when administered as a single agent without or with IL-2 *and* in combination with nivolumab *without or with IL-2*<br>• Incidence of adverse events and ORR as a function of maximum value of neoTCR-P1 T-cell expansion post-infusion, CD4/CD8 ratio of the product, baseline T-cell phenotypes, transfection ratio of the product, and tumor expression of the targeted neoepitope

ADA = anti-drug antibody; AESI = adverse event of special interest; ASTCT = American Society for Transplantation and Cellular Therapy; $AUC_{0–28d}$ = area under the concentration-time curve from 0 to 28 days; C_{max} = maximum concentration; CR = complete response; CRS = cytokine release syndrome; DLT = dose-limiting toxicity; MTD = maximum tolerated dose; NCI CTACAE = National Cancer Institute Common Terminology of Adverse Events; IL-2 = interleukin 2; NeoTCR = neoepitope-specific T-cell receptor; OS = overall survival; PFS = progression-free survival; PR = partial response; RECIST = Response Evaluation Criteria in Solid Tumors; RP2D = recommended Phase 2 dose; T_{cm} = T central memory; T_{eff} = T effector; T_{em} = T effector memory; T_{scm} = T stem cell memory.

Overall Design: This is a first-in-human, single-arm, open-label, Phase 1a/1b study to determine the safety, feasibility, and efficacy of a single dose of NeoTCR-P1 T cells in participants with solid tumors. *Data from the ongoing* study will be reviewed by *the Safety Review Team (SRT)*. The study will be separated into 2 distinct phases: The Initial Phase and the Expansion Phase. Enrollment into the Expansion Phase will only commence following *SRT* review.

Initial Phase: The Initial Phase consists of a single-agent, dose-escalation portion and 2 dose-expansion basket cohorts. The primary objectives of this phase are to evaluate the safety

and tolerability, determine the maximum tolerated dose (MTD), and evaluate manufacturing feasibility of NeoTCR-P1. The dose-escalation portion will evaluate the safety and feasibility of cell products containing gene-edited T-cell receptors (TCR) with up to 3 distinct neoTCR clonal populations (u3TCR).

Three dose levels, ranging from 4×10^8 to 4×10^9 neoTCR-positive T cells, may be evaluated in the Phase 1a dose-escalation portion to determine the safety and activity of NeoTCR-P1. *If enabled by manufacturing, dose escalation may continue in half-log increments beyond Dose Level 3.* The total gene-edited T-cell dose at a given dose level will be the same, independent of the number of neoTCR species in the product (1, 2, or 3). All participants will be evaluated for DLTs; however, only participants who received a product containing 3 neoTCR populations will contribute to clearing a given dose level.

After preliminary safety of NeoTCR-P1 has been evaluated, additional participants may be enrolled in 2 separate dose-expansion basket cohorts (up to 20 participants each) without or with IL-2 within the Initial Phase of the study (see schema below).

In this phase of the study, participants with melanoma, urothelial carcinoma, non-small cell lung cancer (NSCLC), head and neck squamous cell carcinoma (HNSCC), colorectal cancer (CRC), ovarian cancer, human epidermal growth factor receptor 2 (HER2)-negative breast cancer (i.e., hormone receptor-positive [HR+] or triple negative), or prostate cancer will be enrolled.

If acceptable safety and antitumor activity is observed in the Initial Phase of the study, then enrollment into the Expansion Phase may proceed following review by *the SRT*.

Expansion Phase: The Expansion Phase of the study will comprise 2 portions. The Phase 1a portion will include indication-specific dose-expansion cohorts to provide a preliminary estimation of single-agent NeoTCR-P1 activity in 3 different solid tumor indications. NeoTCR-P1 may be administered with IL-2 in the indication-specific expansion cohorts, depending on safety and antitumor activity observed during the Initial Phase. The Phase 1b portion is designed to evaluate the safety, tolerability, and efficacy of NeoTCR-P1 T cells when administered in combination with nivolumab, an anti–*programmed death 1 (*PD-1*)* monoclonal antibody*, without or with* IL-2.

Phase 1a Indication-specific Expansion Cohorts. *Participants in the dose-expansion cohorts will be enrolled at or below the MTD or maximum administered dose (MAD) that has been cleared in the initial Phase 1a dose-escalation portion of the study.* The 3 indication-specific dose-expansion cohorts will enroll participants with tumor types that will be selected by the Sponsor based upon potential efficacy signals observed in the initial dose-*escalation portion of the study, the participants treated in the two* basket cohorts *and/*or other supportive data external to the study. The purpose of the indication-specific expansion cohorts is to more accurately estimate the efficacy in specific tumor types and to determine the breadth of activity across different tumor types with different mutational burdens and immune contextures.

Phase 1b Portion. Enrollment into the Phase 1b portion *may begin after preliminary* safety and *optimal dosing of single-agent NeoTCR-P1 is reviewed* by the *SRT*. The objective of the Phase 1b *portion* is to evaluate a single infusion of NeoTCR-P1 in combination with

nivolumab administered following NeoTCR-P1 on Day 1 and then every 4 weeks for up to *7* cycles. Phase 1b study treatment *may* include IL-2.

Disclosure Statement:
This is a single-group treatment study with no masking.

Number of Participants: Potentially 9–*188* participants will be enrolled as follows:

- Initial Phase: potentially 9–76 participants
- Expansion Phase: potentially *0–92* participants

Intervention Groups and Duration:
Following the initial infusion period, participants will return to the clinic weekly until Week 4, monthly until Month 6, and then every 3 months until Month 24. Following this evaluation, participants will enter a long-term follow up (LTFU) study for annual follow up.

Participants who discontinue from Post-Treatment Follow-Up before Month 24 (e.g., for unequivocal disease progression; see Section 7.1) will enter the Long-Term Follow-Up period at that time.

The expected course of participants in the study is depicted in Section 1.2.

1.2. Participant-Level Study Flow in PACT-0101

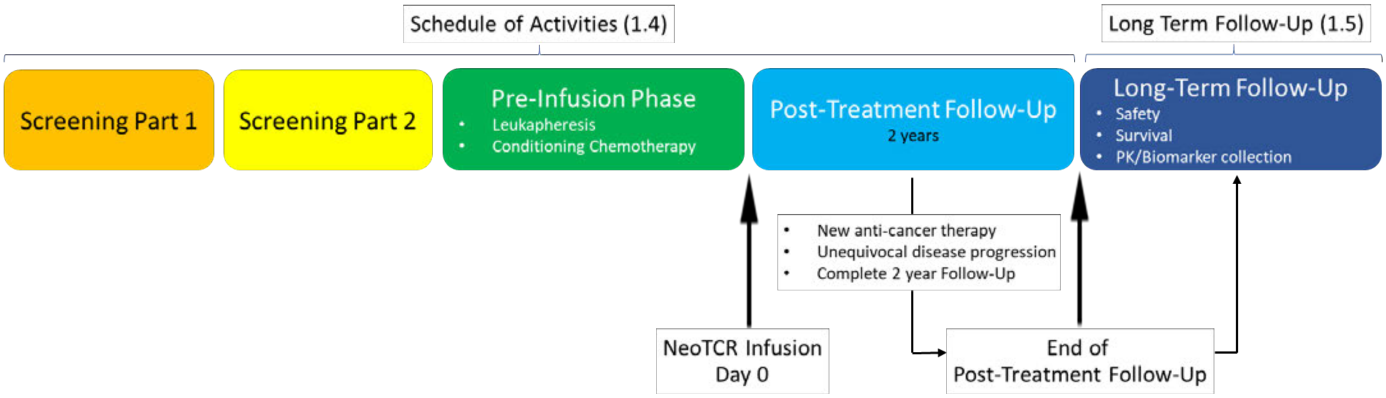

Data Monitoring Committee: *No*

Safety Review Team: Yes

1.3. Schema

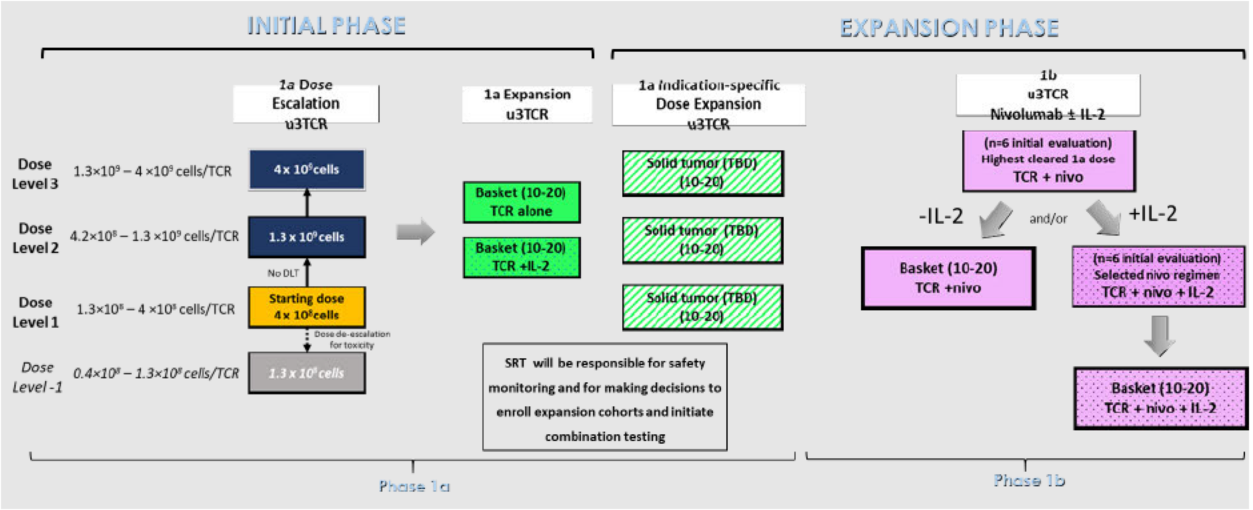

MTD/MAD = maximum tolerated dose/maximum administered dose; neoTCR = neoepitope-specific T-cell receptor; TBD = to be determined; TCR = T-cell receptor; u3TCR = up to 3 TCR clonal populations per product.
Dose based upon neoTCR-positive T cells (includes CD4 and CD8 cells). The total gene-edited cell dose at each dose level is independent of the number of neoTCRs in the product. The number of neoTCR+ T cells/TCR at each dose level is approximately inversely proportional to the number of neoTCRs in the product.
Dose escalation may continue in half-log increments beyond upper dose level if enabled by manufacturing.
Basket and expansion cohorts may be enrolled at any dose at or below the MTD/MAD.
Solid tumor (TBD) expansion cohorts to be determined by Sponsor based upon data from Phase 1a dose escalation and basket expansion cohorts or data external to the study.

1.4. Schedule of Activities

1.4.1. *Screening Part 1 Activities*

Procedures *Timepoint*	*Screening Within 28 Days after Consent*	*Within 28 days of Screening Approval*
Informed consent	*x*	
Assessment of eligibility criteria (Part 1)	*x*	
Oncology history & prior cancer treatment	*x*	
Smoking history	*x*	
Medical history	*x*	
Physical examination [a]	*x*	
ECOG Performance status	*x*	
Vital signs and pulse oximetry	*x*	
Urinalysis	*x*	
Chemistry panel	*x*	
CBC w/differential & T cell subsets	*x*	
HBV/HCV/HIV testing	*x*	
Blood for plasma biomarker analyses	*x*	
Adverse events/ Concomitant medication [b]	*x*	
Tumor tissue		*x* [c]
Blood for PBMCs		*x* [c]

CBC = complete blood count; ECOG = Eastern Cooperative Oncology Group; HBV = hepatitis B virus; HCV = hepatitis C virus; PBMC = peripheral blood mononuclear cell; TCR = T-cell receptor.

[a] *A complete physical examination is required during Screening Part 1. A limited physical examination may be conducted at subsequent timepoints. See Section 8.2.2 for further details.*

[b] *During the Screening Part 1 activities, the collection of concomitant medications will be limited to steroids and anti-infectives. In addition, the time period for collecting adverse events and serious adverse events is described in Section 8.6.1.*

[c] *Tumor samples and whole blood samples for PBMCs are to be collected during Part 1 of screening after eligibility criteria have been met and following approval by the Medical Monitor. If whole blood samples are determined not to be optimal for imPACT analysis, participants may be requested to undergo a small-volume apheresis to increase the number of cells available for processing. Participants who have TCRs identified in a separate screening protocol will provide informed consent and enter this study at Screening Part 2.*

1.4.2. *Screening Part 2 and Main Study Activities*

Procedures	Screening Part 2	Enroll-ment	Conditioning Chemotherapy Period						IP Admin. Period		Post-Treatment Follow-Up (each visit calculated from Day 0)								End of Post-Treatment Follow-Up Visit [p]
			Days						*Days*		*Days*			*Months (M)*					
**Timepoint**	*After TCR ID*		-6 [q]	-5	-4	-3	-2	-1	0	~1-7	14	21	28	End of M2	End of M3	End of M4	End of M5	End of M6 & Q3*M* Until M24	
**Informed consent**	*x* [a]																		
Assessment of eligibility criteria (Part 2)	*x*																		
**Oncology history & prior cancer treatment**	x																		
**Physical examination** [b]	x		*x* [q]	x	x	x			x	x	x	x	x	x	x	x	x	x	x
**ECOG Performance status**	x		*x* [q]								x	x	x	x	x	x	x	x	x
**Neurological assessment (ICE Score)** [c]	x								x	q12h	x	x	x	x				x	
**ECG**	x								x										
**PFT** *(spirometry only)*	x																		
**LVEF assessment**	x		*x* [q]																
**Brain MRI**	x		*x* [q]																
**CMV/EBV**	x																		
**CD3+ T-cell count**	x																		
**Leukapheresis**		x [d]											(x) [e]						
**Baseline & on-treatment biopsy**			*x* [f, g]							x [g] (D+5-7 *and/or* D+28-42)									*x* [g]
**Fludarabine/Cyclophosphamide**			*x*	x	x	x													
**Blood for fludarabine PK**			See Table 1 for collection times																
**Inpatient admission** [h]									x	x									
**NeoTCR-P1 infusion**									x										
**IL-2 (IL-2 participants only)** [i]										bid									
**Nivolumab (Phase 1b only)**										*x* *(Day 1)*			x	x	x	x	x	x (M6 only)	

Procedures	Screening Part 2	Enroll-ment	Conditioning Chemotherapy Period						IP Admin. Period		Post-Treatment Follow-Up (each visit calculated from Day 0)								End of Post-Treatment Follow-Up Visit [p]
			Days						*Days*		*Days*			*Months (M)*					
Timepoint	*After TCR ID*		-6 [q]	-5	-4	-3	-2	-1	0	~1-7	14	21	28	End of M2	End of M3	End of M4	End of M5	End of M6 & Q3*M* Until M24	
**Amylase/Lipase (Phase 1b only)**			*x*						x					x		x		x	X
**Thyroid function (Phase 1b only)**			*x*												x			x	X
**Tumor assessment**	x [j]		*(x)* [k, q]										x	x		x		x	X [p]
**Vital signs and pulse oximetry**	x		*x*	*x*	x	x			x	q4-6h	x	x	x	x	x	x	x	x	X
**Height**	x																		
**Weight**	x		*x*						x	qd	x	x	x	x	x	x	x	x	X
**Pregnancy testing** [l]	x		*x* [q]										(x)					(x)	X
**Urinalysis**	x		*x* [q]																
**Chemistry panel**	x		*x* [q]	*x*	x	x	x	x	x	qd	x	x	x	x	x	x	x	x	X
**CBC w/differential**	x [q]		*x*	*x*	x	x	x	x	x	qd	x	x	x	x [r]	x	x	x	x	X [r]
**C-reactive protein**			*x*						x	qd			x	x		x		x	X
**Serum & Plasma PK/PD** [m]			*x*						x	D 1, 2, & 7	x	x	x	x [r]	x	x	x	x	X [r]
**Blood for PBMC PK** [n]									x	D 1, 2, & 7	x	x	(x) [n]	x [r]	x	x	x	x	X [r]
**Blood for PBMC biomarker analysis**			*x*										(x) [n]	x [r]				x (M6 & 12)	X [r]
**Blood for plasma biomarker analysis**			*x*						x				x	x [r]		x		x	X [r]
**Serum immunogenicity (ADA to nivolumab) (Phase 1b Only)** [o]									x				x	x		x		x (M8)	X
**Adverse events/concomitant medication** [s]	x		------------------> [s]																
**Subsequent therapy for cancer**				As applicable following study treatment discontinuation															

ADA = anti-drug antibodies; Admin. = Administration; bid = twice a day; CBC = complete blood count; CMV = cytomegalovirus; D = Day(s); EBV = Epstein-Barr virus; ECG = electrocardiogram; ECOG = Eastern Cooperative Oncology Group; eCRF = electronic Case Report Form; F/U = Follow-Up; HBV = hepatitis B virus; HCV = hepatitis C virus; ICE = immune effector cell-associated encephalopathy; ID = identification; IP = investigational product; LVEF = left ventricular ejection fraction; M = Month(s); MRI = magnetic resonance imaging; NeoTCR-P1 = personalized adoptive T-cell receptor;

PBMC = peripheral blood mononuclear cell; PFT = pulmonary function test; PD=Pharmacodynamics; PK=Pharmacokinetics; Q3M = every 3 months; q4-6h = every 4-6 hours; q12h = every 12 hours; qd = every day; SC = subcutaneous; TCR = T-cell receptor; TX = Treatment; W = Week(s).

( ) = conditional assessment; see footnotes below for additional details.

In Post-Treatment Follow-Up the duration of a month will be defined as 28 days. Windows for each visit are as follows: weekly visits ±1-day, monthly visits ±4 days, every 3 monthly visits ±7 days.

All blood collections should be performed prior to the administration of study drugs.

[a] Participants who have *TCRs* identified in a separate *screening protocol will provide informed consent and enter this* study *at Screening Part 2.*

[b] A complete physical examination is required during Screening Part *1*. A limited physical examination may be conducted at subsequent timepoints. See Section 8.2.2 for further details.

[c] The neurological assessment should be performed using the ICE score. See Table 24 for additional details.

[d] Leukapheresis for manufacturing should be performed within 5 days of the completion of *Screening Part 2* eligibility assessments. Leukapheresis may be performed prior to the completion of all eligibility criteria in select participants following Medical Monitor approval.

[e] Single blood volume leukapheresis for immune monitoring performed between Days +28–35 is required for participants in the dose-escalation cohort and optional for participants in other cohorts.

[f] *The biopsy on Day -6 is to be collected prior to the conditioning chemotherapy.*

[g] A baseline biopsy is required following enrollment and prior to conditioning chemotherapy, if clinically feasible, in participants who have received systemic therapy between the biopsy material used for manufacturing and the start of conditioning chemotherapy. A baseline biopsy is optional in participants who have not received intervening therapy since providing the biopsy used for manufacturing. If clinically feasible, a post-infusion biopsy should be obtained between Days +5–7 *and/or Days 28–42, and at the time of progression (if applicable).*

[h] See Table 3 for additional collection timepoints during the IP administration period.

[i] *The first* participants will receive IL-2 at 500,000 IU/m^2 SC bid for 7 days. *Following review of safety and PK data from the first 6 participants in the IL-2 basket cohort, the SRT may recommend an alternative IL-2 regimen.*

[j] Baseline tumor assessment must be performed within 28 days of the start of conditioning chemotherapy. See Table 28 regarding additional details related to indication-specific tumor assessments.

[k] Participants who receive bridging therapy after Screening Part 2 and/or whose scans are out of window should repeat tumor assessments to establish a new baseline prior to the start of conditioning chemotherapy. Repeating baseline scans is not required for participants whose screening scans were obtained within the window and who did not receive bridging therapy.

[l] Pregnancy testing to be conducted during Screening Part 2, prior to administration of conditioning chemotherapy and following treatment (Week 4 in the Phase 1a and Month 9 in the Phase 1b). Female participants should also undergo pregnancy testing in the case of delayed menstrual period (over 1 month between menstruations).

[m] In participants who are hospitalized for symptoms of CRS or neurotoxicity plasma/serum (cytokine levels), blood for PBMCs should be collected at the time of admission, 24 hours after admission, then weekly and on the day of discharge. See Table 3 for additional collection timepoints during IP administration period.

[n] Blood for PBMC PK and biomarker collection not required for dose-escalation participants or other participants undergoing single volume leukapheresis at Week 4 timepoint.

[o] Serum samples for nivolumab ADAs will be collected prior to infusion.

[p] A separate End of Post-Treatment Follow-Up visit is not required if the most recent Post-Treatment Follow-Up visit took place <28 days prior to discontinuation. Tumor assessments are not required at the End of Post-Treatment Follow-Up visit if the most recent scans were obtained ≤56 days prior to

discontinuation. If disease progression is the reason for Post-Treatment Follow-Up discontinuation, a tumor assessment documenting radiographic progression must be reported in the eCRF.

[q] See Re-evaluation Prior to Conditioning Chemotherapy (Table 2) for additional details regarding windows for re-evaluations to be performed prior to conditioning chemotherapy.

[r] If clinically feasible every effort should be made to collect M2 CBC, PK/PD, PBMC, and plasma biomarker samples from participants who discontinue from Post-Treatment Follow-Up at the Day 28 visit (e.g., for radiographic progression).

[s] *The time period for collecting AEs and SAEs is described in Section 8.6.1.*

Table 1 Collection Times for Fludarabine Pharmacokinetic and Pharmacogenetic Samples

1st Collection Interval [a]	2nd Collection Interval [a]	3rd Collection Interval
0-2 hours post end of *fludarabine* infusion	4-24 hours post end of *fludarabine* infusion	Immediately prior to neoTCR infusion on Day 0

[a] Samples must be collected after 1 of the doses of conditioning chemotherapy only (i.e., during 2 intervals following the dose on either Day -6, -5, -4, or -3). It is preferred not to collect samples immediately after infusion and at trough (i.e., at 0 hours and 24 hours).

Table 2 Windows for Re-evaluation Prior to Conditioning Chemotherapy

Procedures	Timepoint (in reference to the start of conditioning chemotherapy)
**LVEF assessment**	Within 90 days
**Brain MRI**	Within 90 days
**Tumor assessments**	Within 28 days
**Chemistry panel**	Within 14 days
**Thyroid panel**	Within 14 days
**CBC w/differential**	Within 14 days
**Urinalysis (and cultures if indicated)**	Within 14 days
**Pregnancy testing**	Within 7 days
**Physical examination**	Within 7 days
**ECOG Performance Status**	Within 7 days

CBC = complete blood count; ECOG = Eastern Cooperative Oncology Group; LVEF = left ventricular ejection fraction; MRI = magnetic resonance imaging.

Table 3 Frequency of Assessments during Inpatient Hospitalization for IP Administration Period

Procedures	Frequency
**Vital signs**	Every 4-6 hours
**Physical examination**	Daily
**Weight**	Daily
**Neurological assessment (ICE Score)**	Every 12 hours
**C-reactive protein**	Daily
**CBC w/differential**	Daily
**Chemistries (including liver function tests)**	Daily
**C-reactive protein**	Daily
**Ferritin**	Daily
**Serum and Plasma PK/PD**	Collect on Day 1, 2 and 7
**Blood for PBMC PK**	Collect on Day 1, 2 and 7

CBC = complete blood count; ICE = immune effector cell-associated encephalopathy; NeoTCR-P1 = personalized adoptive T-cell receptor; PBMC = peripheral blood mononuclear cell; PD = pharmacodynamics; PK = pharmacokinetic.

1.5 Schedule of Activities for Long-Term Follow-Up

Procedures	Long-Term Follow-Up Period (Each Visit Calculated from Day 0)
	Yearly [a] (±1 month)
**Survival status**	x
**CBC w/differential**	x
**Plasma biomarker**	x
**Blood for PBMC PK analysis**	x
**Blood for PBMC biomarker analysis**	x
**Subsequent therapy for cancer**	x

CBC = complete blood count; PBMC = peripheral blood mononuclear cell; PK = pharmacokinetic.

[a] If the most recent Post-Treatment Follow-Up visit or the End of Post-Treatment Follow-Up visit took place <3 months before Year 1 or Year 2, that Long-Term Follow-Up visit is not required.

2. Introduction

NeoTCR-Product 1 (P1), the first product candidate from PACT Pharma, is a novel autologous precision genome-engineered neoepitope-specific T-cell receptor (neoTCR) T-cell product. It is being developed as a single-dose administration as a single agent without or with interleukin-2 (IL-2), or in combination with the anti-programmed cell death 1 (PD-1) antibody, nivolumab, for the treatment of patients with solid tumors.

2.1. Study Rationale

The cellular immune response makes a major contribution to the control and eradication of human cancers. Clinical benefit from unleashing endogenous T-cell immune responses following treatment with checkpoint inhibitors (e.g., anti–PD-1, anti–PD-L1) is routinely observed as rapid and durable tumor shrinkage following initiation of therapy and has resulted in improvements in overall survival (OS) in patients with advanced malignancies in a wide spectrum of tumor types (Brahmer et al., 2012; Topalian et al., 2012; Hamid et al., 2013; Wolchok et al., 2013; Herbst et al., 2014; Robert et al., 2015). Nonetheless, despite the success of therapies that block inhibitory immune checkpoint signals to T cells, cancer progression is unchanged or recurs in a significant proportion of patients.

Evidence to date suggests that checkpoint-responsive solid tumors are more likely to exhibit higher somatic mutation burden, higher CD8 T-cell infiltration and/or pre-existing programmed death ligand 1 (PD-L1) expression (Schumacher and Schreiber 2015) – all of which would be associated with a higher potential for intrinsic immunogenicity of these tumors (Lawrence et al., 2013; Tumeh et al., 2014; Roh et al., 2017). The T cells that specifically target the intrinsic tumor mutations are proposed to be the main mediators of effective cancer immunotherapies to trigger clinical benefit (Schumacher and Schreiber 2015, Tran et al., 2017).

Several approaches have recently been developed to harness the potency of T cells. Some strategies have involved increasing the number of endogenous tumor-targeted T cells while others have attempted to redirect T cells to target tumor associated antigens in patients with cancer. Treatment with chimeric antigen receptor (CAR)-engineered autologous T cells targeting CD19 have yielded remarkable complete response (CR) rates in patients with chemotherapy refractory lymphomas and leukemias (Maude et al., 2018; Neelapu et al., 2017; Schuster et al., 2017; Park et al., 2018). The administration of autologous T cells expanded from tumor-infiltrating T cells (TILs) has also been shown to induce clinical responses in patients with melanoma, Merkel-cell carcinoma, and other tumor types that are not traditionally responsive to checkpoint inhibitor drug therapy (e.g., cholangiocarcinoma, microsatellite-stable [MSS], colorectal cancer [CRC], and hormone receptor positive [HR+] breast cancer) (Tran et al., 2014; Tran et al., 2016; Paulson et al., 2018; Zacharkis et al., 2018). More recently, the administration of engineered autologous T-cell receptor (TCR)-T cells directed against shared tumor antigens (e.g., NY-ESO-1) and viral antigens (e.g., *human papillomavirus* [HPV] 16 E7) shows evidence of antitumor activity (D'Angelo et al., 2018; Norberg et al., 2018; Doran et al., 2019). These data support the premise that solely augmenting the number of tumor-targeted T cells by adoptive T-cell transfer holds potential to provide clinical benefit for patients with solid tumors. A comparison of CARs, TCRs, and TILs is presented in Table 4.

Personalized cancer mutation-targeted therapies such as RNA or peptide-based vaccines targeting cancer neoantigens are a promising new therapeutic modality with potential to increase the magnitude of pre-existing mutation-targeted T-cell responses as well as generate *de novo* immune responses against tumor neoantigens (Ott et al., 2017; Sahin and Türeci, 2018). Neoantigen cancer vaccines may be particularly beneficial in low mutational burden tumor types, such as prostate, MSS CRC, or other translocation-driven tumors where insufficient numbers of T cells are primed intrinsically to tumor antigens. Nonetheless, the potential benefit of neoantigen cancer vaccines may be limited by an inability to validate predicted antigens prior to vaccine administration, by the variable response to T-cell vaccines in the human population and the challenge of eradicating large or fast-growing tumors with a more gradually expanding anti-tumor immune response.

Adoptive transfer of gene-edited neoTCR T cells targeting neoantigens is an immunotherapy modality designed to unleash the immune system's ability to specifically recognize tumor-exclusive mutational targets in each participant. Since all cancers are driven by underlying founder or truncal mutations, adoptive neoTCR T-cell therapy targeting tumor-exclusive neoepitopes holds the potential for treatment of any patient with cancer in whom a neoTCR can be identified. NeoTCR-P1 adoptive cell therapy involves engineering an individual's own CD8 and CD4 T cells to express naturally occurring neoTCRs that already recognize tumor-specific antigens. These neoTCRs are identified and authenticated from pre-existing mutation-targeted CD8 T cells captured from a small sample of blood derived from each participant. In a subsequent step, freshly derived CD4 and CD8 T cells of the same patient are genetically engineered to express 1 neoTCR in a manner that reconstitutes fully "native" autologous T-cell function. The potential for significant clinical benefit to participants with solid tumors thus stems from delivering a single dose of ex vivo engineered, tumor mutation-targeted autologous NeoTCR-P1 cells.

Table 4 Comparison of CARs, TCRs, and TILs

	CAR	TCR	TIL
**Example**	Yescarta, Kymriah	**NeoTCR-P1**	Lifileucel
**Target Location**	Surface targets only	**Surface and intracellularly expressed target proteins**	Surface and intracellularly expressed target proteins
**Target types**	Shared antigens only	**Shared and neoantigens**	Shared and neoantigens
**Target density for triggering T-cell activity** [a]	High (100s)	**Low (1-5)**	Low (1-5)
**HLA-dependent**	No (MHC independent. engagement & induction of signal)	**Yes**	Yes
**Receptor on T cell**	Artificial receptor (scFv)	**Natural TCRs**	Natural TCR
**Signaling domain**	Artificial (e.g., 4-1BB, CD28)	**Natural**	Natural
**Kinetics of response**	Immediate effector function + memory	**Immediate effector function + memory**	Immediate effector function, high potential for T-cell exhaustion
**Shared TCRs**	Mfg. requires large batch using viral production	**Yes**	Yes
**Neoepitope-specific TCRs**	N/A	**Can be isolated from PBMCs and small amount of tumor tissue**	Mfg. difficult; Low number of cells and exhausted phenotype.

CAR = chimeric antigen receptor; HLA = human leukocyte antigen; Mfg. = manufacturing; MHC = major histocompatibility complex; N/A = not applicable; PBMC = peripheral blood mononuclear cell; scFv = single-chain variable fragment; TCR = T-cell receptor; TIL = tumor-infiltrating T cells.

[a] Source: Stone et al. 2012; Watanabe et al., 2018.

In summary, novel therapies for patients with relapsed or refractory metastatic solid tumors are urgently needed. The administration of NeoTCR-P1 cells combines the selection of an authenticated personalized tumor-exclusive target for each participant (i.e., neoantigen) with the potency of T-cell therapies, thus providing the potential to trigger rapid and durable responses in patients who have no curative treatment options. This first-in-human clinical trial will assess the safety and feasibility of NeoTCR-P1 either as a single agent without or with IL-2, or in combination with nivolumab in patients with relapsed/refractory solid tumors as measured by the adverse event rate and percentage of screened patients for whom product can be manufactured. If anti-tumor activity is observed and the administration of NeoTCR-P1 is tolerable and feasible, then the study may proceed to an Expansion Phase following review by *the SRT*.

2.2. Background

Despite the development and approval of multiple checkpoint inhibitors (e.g., pembrolizumab, atezolizumab, nivolumab), novel targeted agents (e.g., cetuximab) and other new classes of therapy (e.g., poly ADP ribose polymerase [PARP] inhibitors, selective estrogen receptor modulators/degraders [SERM/SERD]) over the last few years, survival remains limited for patients with solid tumors who fail to respond, or progress, after first-line therapy. In general, the

goals of therapy in second-line are palliative and involve balancing improvements in progression-free survival and quality of life with toxicity. The following sections address 8 tumor types reported to harbor high, intermediate, and low mutation burden wherein patients with these cancers will be enrolled in the NeoTCR-P1 first-in-human trial.

2.2.1. Melanoma

Melanoma is a form of skin cancer and the sixth most common cancer in men and women (Siegel et al., 2018). While most cases are diagnosed at an early stage and cured with surgical resection alone, some patients will present with *de novo* metastatic disease or recur following initial resection. In 2019, it is estimated that 96,480 new cases of melanoma will be diagnosed and approximately 7,230 people are expected to die of melanoma in the United States. In patients with advanced melanoma (i.e., Stage IV), the 5-year survival rate is approximately 15−25% (Schadendorf et al., 2015).

For patients with advanced disease, current treatments include targeted therapy (BRAF/MEK inhibitors) for patients with *BRAF* V600-mutant tumors and immune therapies (e.g., IL-2, PD-1, cytotoxic T-lymphocyte-associated [CTLA]-4). Cytotoxic chemotherapy is also a treatment option but has not been shown to improve survival and its use has generally been limited to those patients who are not candidates for further treatment with targeted agents or immunotherapy and who have no available clinical trial options. In the Checkmate-037 study in which patients were randomized to either nivolumab or investigator's choice of chemotherapy (ICC; dacarbazine alone or paclitaxel combined with carboplatin) in the second line following prior receipt of ipilimumab (anti–CTLA-4) or ipilimumab and a BRAF inhibitor, if they were $BRAF^{v600}$ mutation-positive, chemotherapy was associated with a response rate of 10% and a progression-free survival of 3.7 months (Weber et al., 2015). While the OS of the ICC group was approximately 14 months, there was significant crossover to subsequent therapy with anti–PD-1 agents in the control group (Larkin et al., 2018). In the BRIM-3 study that compared vemurafenib against dacarbazine, the median OS with dacarbazine was 9.6 months (95% confidence intervals [CI] 7.9–11.8) and the 12-month OS rate was 43% (Chapman et al., 2012). In earlier Phase 3 studies of dacarbazine, the response rate ranged from 7% to 12% and the median OS was 5.6 to 7.8 months.

While checkpoint inhibitor antibody drugs such as pembrolizumab, nivolumab (with or without ipilimumab) are approved first-line options, there are no standard second-line options for BRAF wild-type patients or third-line options for BRAF-mutant patients following targeted therapy. Furthermore, as the administration of anti–PD-1 antibodies has moved to earlier lines with their approval for patients with high-risk disease as adjuvant therapy, there are also limited treatment options for patients who rapidly relapse after receiving adjuvant immunotherapy.

2.2.2. Urothelial Carcinoma

Urothelial carcinoma or transitional cell carcinoma is the most common type of bladder cancer and can also involve the renal pelvis, ureter, and urethra. In the United States, there were an estimated 80,470 new cases and 17,670 deaths from bladder cancer in 2019. For patients with advanced or metastatic disease the 5-year survival has been estimated at 4.8% based upon an analysis of the Surveillance, Epidemiology, and End Results (SEER) database (SEER 18 2008-2014).

Cisplatin-based chemotherapy (e.g., methotrexate, vinblastine, doxorubicin, and cisplatin [MVAC], gemcitabine and cisplatin [GC]) is considered the standard initial therapy for medically fit patients with advanced disease and associated with OS benefits. For patients who are considered cisplatin-ineligible (i.e., World Health Organization [WHO] Performance Status of 2 or greater, creatinine clearance less than 60 mL/min, hearing loss of 25 dB at 2 contiguous frequencies, Grade ≥2 peripheral neuropathy, or New York Heart Association class III or greater heart failure) carboplatin-based regimens, non-platinum regimens, atezolizumab and pembrolizumab, are considered standard of care options. For patients whose disease progressed following treatment with platinum-based regimens, multiple anti–PD-L1 or anti–PD-1 antibodies (e.g., atezolizumab, durvalumab, avelumab, pembrolizumab, nivolumab) have been approved by the Food and Drug Administration (FDA). While these agents represent a substantial improvement with respect to tolerability and durability over chemotherapy (e.g., docetaxel, paclitaxel, vinflunine) which had been the prior standard of care, the agents are all associated with modest response rates and improvements in OS.

Table 5 Summary of PD-1/PD-L1 Activity in Patients with Metastatic or Locally Advanced Urothelial Carcinoma

Study	Phase	Population	Number of Patients	ORR	PFS (months)	OS (months)
**Pembrolizumab (anti–PD-1)**						
Keynote 045	III	2L after failure of platinum-containing regimen	542	21.1%	2.1	10.3
Keynote 052	II	1L cis-ineligible	374	28.9%	NR	11.5
**Nivolumab (anti–PD-1)**						
CheckMate 275	II	2L after failure of platinum-containing regimen	270	19.6%	2.0	8.7
**Atezolizumab (anti–PD-L1)**						
IMVigor210	II	2L after failure of platinum-containing regimen	310	15%	2.1	11.4
IMVigor210	II	1L cis-ineligible	119	23%	2.7	15.9
IMVigor211	III	2L after failure of platinum-containing regimen	931	13.4%	2.1	8.3

1L = first-line therapy; 2L = second-line therapy; NR = not recorded; ORR = objective response rate; OS = overall survival; PD-1 = programmed cell death 1; PD-L1 = programmed cell death ligand 1; PFS = progression-free survival.

2.2.3. Head and Neck Squamous Cell Cancer

HNSCC is responsible for 3% of all cancers and approximately 1.5% of all cancer deaths (10,030) in the United States (Bray et al. 2018). While the global incidence of HNSCC is slowly declining because of decreased tobacco use, cases of sexually transmitted human papillomavirus (HPV)-associated oropharyngeal cancer are rising. While HPV-positive disease tends to affect younger, fitter patients and has a better prognosis, treatment algorithms are generally the same regardless of HPV status (Chow, 2020).

Mutation rates do not differ by HPV status (Cancer Genome Atlas Network, 2015), and HPV+ and HPV- tumors are expected to be equally amenable to tumor mutation-targeted autologous T-cell therapy. Among nasopharyngeal cancers, which are often associated with Epstein-Barr virus (EBV), EBV infection is marginally correlated with high tumor mutational burden (Ali et al. 2017).

Stage I or II disease (30%-40% of patients) can often be treated successfully with surgery or radiotherapy, with long-term survival rates as high as 90%. However, more than 60% of patients present with Stage III or IV disease which has a poor prognosis, with a median survival of 15 months or less depending on disease- and patient-specific factors. Definitive locoregional therapy with concurrent chemotherapy (generally with a cisplatin-containing regimen) is the standard of care for patients with locally advanced disease and good performance status.

Management of patients with metastatic or recurrent disease depends on prior treatment for local HNSCC, performance status and related prognostic factors, and PD-L1 combined positive score (CPS). Pembrolizumab was recently established as a standard of care in the first-line setting. The KEYNOTE-48 study compared pembrolizumab alone to pembrolizumab with chemotherapy (platinum + 5-fluorouracil) and cetuximab with chemotherapy (Burtness et al. 2019). Single-agent pembrolizumab significantly improved overall survival in patients with CPS ≥20 and CPS ≥1 when compared to the standard cetuximab plus chemotherapy regimen (14.9 months vs 10.7 months and 12.3 vs 10.3, respectively). At the second interim analysis, pembrolizumab with chemotherapy improved overall survival versus cetuximab with chemotherapy in the total study population (13·0 months vs 10·7 months).

For patients who relapsed after prior systemic therapy for metastatic or recurrent disease, the choice of subsequent therapy is based on previous treatments, with active agents including cetuximab and various single agent or combination chemotherapy regimens.

Given the poor outcomes and relative weakness of recommendations to guide the selection of regimens in the second-line therapy and beyond, clinical trials are an acceptable option in this setting.

2.2.4. Non-Small Cell Lung Cancer

Lung cancer is the leading cause of cancer deaths worldwide, with 1.8 million deaths in 2018 (18.4% of all sites; Bray et al., 2018). More than 80% of patients are diagnosed with non-small cell disease, and the majority of patients (approximately 65%) present with metastatic disease (Aupérin et al., 2010). The 5-year survival rate for patients with metastatic non-small cell lung cancer (NSCLC) is 5%, although the prognosis is improving with the introduction of effective new therapies and smoking cessation programs (cancer.net 2020).

In the metastatic setting, the goal of treatment is to prolong survival while palliating disease symptoms. The choice of treatment for advanced NSCLC is determined by the following factors: PD-L1 expression, the presence of a driver mutation (e.g., EGFR, ALK, ROS1, BRAF), the extent and symptoms of disease, and histology (squamous versus non-squamous).

For patients without driver mutations, the addition of checkpoint inhibitors to standard platinum doublet regimens has led to significant improvements in survival. In the IMpower 150 study, atezolizumab plus carboplatin/paclitaxel/bevacizumab (ACBP) demonstrated longer overall survival compared to the BCP alone (19.2 months vs 14.7 months). Progression-free survival was also improved in the ABCP and BCP arms: 8.3 months versus 6.8 months, respectively (Socinski et al., 2018). Similarly, KEYNOTE-189 demonstrated the benefit of pembrolizumab when added to pemetrexed and a platinum drug as a first-line therapy for nonsquamous NSCLC (Gandhi et al., 2018). In this study, the pembrolizumab and placebo combination groups demonstrated 12-month overall survival rates of 69.2% and 49.4%, respectively. Progression-free survival was 8.8 versus 4.9 months. Benefit in the KEYNOTE-189 study was seen across all PD-L1 expression categories. The KEYNOTE-24 study had previously demonstrated that single-agent pembrolizumab resulted in superior PFS, 6-month OS rates, *objective response rate* (ORR)*, and duration of response* (DOR) compared to doublet chemotherapy in patients with high (≥50%) tumor PD-L1 expression (Reck et al., 2016). In addition, treatment-related adverse events of all grades were less frequent with pembrolizumab. The clinical benefit seen in these studies has established checkpoint inhibitors as a standard of care for the initial treatment of advanced NSCLC.

For patients with EGFR or ALK driver mutations, treatment with a TKI is superior to standard chemotherapy as initial treatment for advanced disease (Rosell et al., 2012; Leon et al., 2014; Solomon et al., 2018). Next generation TKIs have continued to extend overall survival in these populations and it now exceeds what is typically achieved in patients with wild-type tumors (Ou et al., 2020; Ramalingam et al., 2020). After progression on targeted agents, patients with driver mutations are generally offered chemotherapy plus immunotherapy (IMpower150 or KEYNOTE-189 regimens) although the efficacy of chemotherapy in such patients is modest, and more data are needed to clarify the role of immunotherapy.

In subsequent lines, few effective treatment options exist and the efficacy of standard-of-care second- and third-line chemotherapies, such as docetaxel is offset by substantial toxicities. Clinical trials are an acceptable alternative at this point in the course of disease.

2.2.5. Colorectal Cancer

CRC is a significant public health problem in the United States and the third leading cause of cancer deaths for men and women. In 2019, it is estimated that 101,420 new cases of colon cancer and 44,180 new cases of rectal cancer will occur and that approximately 51,020 deaths will result from CRC (American Cancer Society 2019). The last 10 to 15 years have seen the approval of multiple new agents in different classes that have resulted in improved survival outcomes for patients with CRC. Nonetheless, the prognosis is limited for patients whose metastatic disease progresses after first-line therapy for which the goals of treatment remain palliative rather than curative. While immunotherapy (i.e., pembrolizumab, nivolumab) has demonstrated durable responses in patients with CRC these profound benefits have been limited to a small subset of patients with high microsatellite instability (MSI-H) or mismatch repair deficiency (dMMR) phenotypes (Lee V et al., 2016).

The initial treatment approach for patients with non-operable CRC is based upon patient fitness, *BRAF*/*RAS* mutation status, and the location of the primary tumor and includes a cytotoxic

chemotherapy backbone that is either oxaliplatin-based or irinotecan-based. Subsequent-line treatment is variable and generally directed by the first-line treatment (e.g., switch from oxaliplatin-containing regimen to irinotecan plus leucovorin) (Lee J et al., 2016).

Table 6 Summary of Outcomes for Second-Line Treatment of Metastatic Colorectal Cancer

Second-line regimen (Reference)	First-line regimen	Median PFS (months)	Median OS (months)
Bevacizumab + FOLFOX4 versus FOLFOX4 alone (Giantonio et al., 2007)	Irinotecan-based therapy alone	7.3 vs. 4.7	12.9 vs. 10.8
Bevacizumab monotherapy	Irinotecan-based therapy alone	2.7	10.2
FOLFIRI + Aflibercept versus FOLFIRI + placebo (Van Custem et al., 2012)	Oxaliplatin-based therapy alone	6.9 vs. 5.4	13.9 vs. 12.4
FOLFIRI + Aflibercept versus FOLFIRI + Placebo (Van Custem et al., 2012)	Bevacizumab + Oxaliplatin-based therapy	6.2 vs. 3.9	12.5 vs. 11.7
Bevacizumab + Oxaliplatin-based therapy or Bevacizumab + irinotecan-based therapy versus oxaliplatin based therapy alone or irinotecan-based therapy alone (Bennouna et al., 2013)	Bevacizumab + irinotecan-based therapy or Bevacizumab + oxaliplatin-based therapy	5.7 vs. 4.9	11.2 vs 9.8

OS = objective survival; PFS = progression-free survival.
Source: Smaglo et al., 2013.

2.2.6. Ovarian Cancer

Ovarian cancer is the second most common cancer of the female reproductive system and the fifth most common cause of cancer-related death in women. In 2019, it is estimated that 22,530 women will be diagnosed with ovarian cancer and that 13,980 women will die from the disease (American Cancer Society 2019). The majority of ovarian malignancies arise from the epithelium. High-grade serous epithelial ovarian carcinoma, fallopian tube, and peritoneal carcinomas are considered a single clinical entity due to their shared clinical behavior and treatment.

Platinum-based chemotherapy combined with a taxane is the first-line standard of care therapy. In women who respond to first-line chemotherapy and have a germline BRCA mutation, maintenance therapy with olaparib, a PARP inhibitor, is now considered standard of care. Most women with advanced disease will recur following an initial response to therapy. Those that have a platinum free interval >6 months are considered to be “platinum sensitive” while those who have a platinum free interval <6 months are considered to be “platinum resistant.” While women with platinum-sensitive disease have multiple treatment options that include platinum retreatment and PARP inhibitor, women with platinum-resistant disease have more limited options. Single-agent treatment with topotecan, paclitaxel, or pegylated liposomal doxorubicin is considered standard of care resulting in similar survival outcomes, but each is associated with a different toxicity profile.

Table 7 Outcomes for Relapsed Epithelial Ovarian Cancer in Platinum-Resistant Disease

Treatment regimen (Ref)	Number of patients	ORR	Median PFS (months)	Median OS (months)
Paclitaxel + Bevacizumab vs. Paclitaxel alone (Pujade-Lauraine et al., 2014)	115	53% vs. 30%	10 vs. 4	16.6 vs. 13.3
Topotecan + Bevacizumab vs. Topotecan alone (Pujade-Lauraine et al., 2014)	120	17% vs. 0%	6 vs. 2	
Liposomal doxorubicin (PLD) + Bevacizumab vs. PDL alone (Pujade-Lauraine et al., 2014)	126	14% vs. 8%	5 vs. 4	
PLD vs. Topotecan (Gordon et al., 2001)	474	20% vs. 17%	TTP. 22 weeks vs. 20 weeks	66 vs. 56 weeks
Gemcitabine vs. PLD (Mutch et al., 2007)	195	9% vs. 11%	4 vs. 3	13 vs. 14

ORR = objective response rate; OS = overall survival; PFS = progression-free survival.

2.2.7. Hormone-Receptor Positive Breast Cancer

Breast cancer is the most common nonskin cancer in US women. It is estimated that there were 252,710 new cases of invasive breast cancer in the US and an estimated 40,610 deaths with African-American women having the highest rates of death. There are 4 main molecular subtypes of breast cancer, which include Luminal A (HR+/HER2-negative), Triple negative (hormone receptor negative [HR-]/HER2-negative), Luminal B (HR+/HER2+), and HER2-enriched (HR-/HER2+), that account for 71%, 12%, 12%, and 5% of invasive breast cancer, respectively.

For postmenopausal women with hormone receptor-positive (i.e., estrogen receptor-positive [ER+]/progesterone receptor-positive [PR+]) metastatic breast cancer (MBC), endocrine therapy with an aromatase inhibitor (AI) plus a CDK4/6 inhibitor (e.g., palbociclib, ribociclib) is the preferred first-line therapy. Alternatives include selective estrogen receptor degraders such as fulvestrant or AI monotherapy. At progression, women with HR+ breast cancer are generally switched to an alternative endocrine therapy. For women who progress after 2 or more lines of endocrine therapy and who are symptomatic, combination chemotherapy or single-agent chemotherapy with a taxane, anthracycline, capecitabine, or another agent is a reasonable treatment option. For women receiving second and subsequent lines of chemotherapy for MBC the outcomes are limited. In an observational study of women receiving second-line chemotherapy (n = 209), the progression-free survival was 5.1 months (Park et al., 2015).

Table 8 Outcomes for Second and Subsequent-Line Treatment of Metastatic Breast Cancer

Treatment Regimen (Reference)	Number of Patients	ORR	Median PFS (months)	Median OS (months)
Capecitabine + docetaxel vs. Docetaxel (O'Shaughnessy et al., 2002)	255 (capecitabine + docetaxel) 256 docetaxel	42% vs. 30%	TTP 6.1 vs. 4.2	14.5 vs. 11.5
Capecitabine (Fumoleau et al., 2004)	126	28%	TTP 5	15
Ixabepilone + capecitabine vs. Capecitabine (Thomas et al., 2008)	369 (ixabepilone + capecitabine) 368 capecitabine	42% vs. 23%	5.3 vs. 3.8	NS
Nab-paclitaxel (Blum et al., 2007)	106 (100 mg/m^2) 75 (125 mg/m^2)	14% and 16%	3 and 3.5	9.2 and 9.1
Eribulin vs. Capecitabine (Kaufman et al., 2015)	554 vs. 548	11.0% vs. 11.5%	4.1 vs. 4.2	15.9 vs. 14.5

TTP = time to progression; ORR = objective response rate; OS = overall survival; PFS = progression-free survival; Source: Roché et al., 2011.

2.2.8. Triple Negative Breast Cancer

Triple-negative breast cancer (TNBC) is defined by the absence of immunostaining for estrogen receptor (ER), progesterone receptor (PR), and HER2. Overall, approximately 15%-20% of breast cancers are classified as TNBC. Patients with metastatic TNBC exhibit poor clinical outcomes, generally with rapid progression and a median overall survival of 18 months or less (Rodler et al., 2010; Miles et al., 2013; Gobbini et al., 2018; Yardley et al., 2018).

Chemotherapy is the mainstay of systemic treatment, with first-line systemic regimens typically consisting of a taxane or anthracycline combination, with response rates of approximately 20%-40% and progression-free survival ranging from 6-8 months. Palliation is the main goal of treatment for metastatic disease, and outcomes decline progressively in second and later lines.

The PD-L1 inhibitor atezolizumab was recently approved for the treatment of advanced TNBC based on the results of the Impassion130 study (Schmid et al., 2018). In this study, first-line patients were randomized to receive nab-paclitaxel + atezolizumab or nab-paclitaxel + placebo. Clinical benefit was limited to patients whose tumors expressed PD-L1;hazard ratio = 0.62, 95% CI 0.49–0.78; P <0.0001). Among patients with PD-L1-positive tumors, Kaplan–Meier analyses showed a median overall survival of 25.0 months in the nab-paclitaxel + atezolizumab group and 15.5 months in the nab-paclitaxel + placebo group, although this result could not be tested formally.

2.2.9. Prostate Cancer

Prostate cancer is the most common nonskin cancer in US men. It is estimated that approximately 174,650 new cases will be diagnosed, and an estimated 31,620 deaths will result from prostate cancer in 2019. The disease develops mainly in older men and occurs with a higher frequency in African-American men.

For men with symptomatic metastatic castration-resistant prostate cancer (CRPC) systemic therapy options include agents that interfere with androgenic stimulation, taxane chemotherapy, and bone-targeted radiopharmaceuticals. Androgen pathway inhibitors such as abiraterone or enzalutamide are first-line therapy in patients with metastatic CRPC and generally associated with high overall and prostate-specific antigen (PSA)-response rates. Chemotherapy is generally reserved for patients with relatively rapid progressive symptomatic disease with docetaxel being the preferred agent for initial treatment. While docetaxel has been associated with an OS benefit, OS benefits have not been demonstrated for cabazitaxel or mitoxantrone. Recent studies (i.e., STAMPEDE, CHAARTED, GETUG-AFU 15) have demonstrated the benefit of moving these agents (e.g., abiraterone plus androgen-derivation therapy [ADT], docetaxel plus ADT) to high-risk patients in earlier lines of therapy. There is limited information to inform later line treatment options in patients who receive these agents within the castrate-sensitive disease setting.

Table 9 Outcomes for Second/Third-Line Therapy in Patients with CRPC

Second-Line Treatment (Ref)	Prior Treatment	ORR	Median PFS (months)	Median OS (months)
Cabazitaxel (TROPIC) (de Bono et al., 2010)	Docetaxel	14.4%	2.8	15.1
Abiraterone (COU-AA-301) (de Bono et al., 2011)	Docetaxel	14.8%	5.6	15.8
Enzalutamide (AFFIRM) (Scher et al., 2012)	Docetaxel	29%	8.3	18.4
Docetaxel (Mezynski et al., 2012)	Abiraterone	11%	TTP (PSA) 4.6	12.4
Abiraterone (Noonan et al., 2013)	Enzalutamide	0%	TTP 15.4 weeks	50.1 weeks
Abiraterone (Loriot et al., 2013)	Enzalutamide	8%	2.7	7.2
Enzalutamide (Bianchini et al., 2014)	Docetaxel and abiraterone	4.3%	2.8	NR
Enzalutamide (Schrader et al., 2014)	Docetaxel and abiraterone	2.9%	4.0	7.1

ORR =objective response rate; OS = overall survival; PFS = progression-free survival.

2.3. Engineered TCR-T Cells for Advanced Malignancies

There are several ongoing or completed TCR gene therapy trials in patients with solid tumors and hematologic malignancies. These cell therapy products have targeted tumor associated antigens, cancer testis antigens, and viral antigens (see Table 10). While impressive response rates were observed in many patients, thus validating the approach, some were transient. In addition, “on-target”/“off-tumor” toxicities (e.g., rash, uveitis, diarrhea, neural/cardiac toxicity, were noted) (Cameron et al., 2013; Linette et al., 2013).

Table 10 Clinical Studies with Gene-Edited TCRs

Indication (Ref)	TCR-Target & Engineering	No. of Subjects Treated (Evaluable)	Efficacy	Safety
Multiple Myeloma (HLA-A2 restricted) (Rapoport et al., 2015)	NY-ESO-1 / LAGE-1 (NY-ESO^{c259})	20	ORR 16/20 mPFS 19.1 months	No treatment-related fatalities. Skin rash with lymphocytosis occurred in 3/20 and 3/20 developed aGVHD (GI). SAEs included hypoxia, neutropenia, hyponatremia, hypotension, pancytopenia, dehydration. CRS and neurotoxicity were not reported.
Synovial Sarcoma (HLA-A2 restricted) (D'Angelo et al., 2018)	NY-ESO-1 /LAGE-1a (NY-ESO^{c259})	12 (10)	ORR 6/12 (1 CR) Median DoR 30.9 weeks	No fatal AEs. 11/12 patients reported TRAEs Grade ≥3. Most common AEs were lymphopenia (100%), leukopenia (92%), neutropenia (83%), anemia (83%), hypophosphatemia (67%), and thrombocytopenia (67%). FN 17%. 2/12 patients experienced sCRS. No neurotoxicity was reported.
Synovial Sarcoma or Metastatic Melanoma (HLA-A2 restricted) (Robbins et al., 2011)	NY-ESO-1	38 (18 Sarcoma and 20 Melanoma)	ORR *4/6* (Sarcoma) *5/11* (Melanoma 4 CRs)	All patients experienced transient neutropenia and thrombocytopenia. There was 1 death from septic shock in a patient with neutropenia.
Metastatic Melanoma (HLA-A2 restricted) (Johnson et al., 2009)	DMF5 MART-1 and gp100	36 (20 MART-1 and 16 gp100)	ORR 6/20 (DMF5 MART-1) 3/16 (gp100)	Anterior uveitis, rash, hearing loss
Metastatic Melanoma (HLA-A2 restricted) (Morgan et al., 2006)	MART-1	17	ORR 2/17	Not reported
Metastatic Melanoma (HLA-A2 restricted) (Chodon et al., 2014)	MART-1	14 (13)	11/14 showed evidence of transient tumor response	Two patients had SAEs of acute respiratory distress requiring intubation associated with patchy pulmonary infiltrates within 1 week of cell infusion, resulting in the discontinuation of this cohort due to increased toxicities
Hodgkins Lymphoma (Bollard et al., 2018)	EBV-derived tumor antigens (LMP-1 and LMP-2) T cells expressed DNRII	8 (7)	ORR 4/7 (4 CRs)	No DLT occurred. No AI, GVHD, or sCRS observed. Most common TRAEs Grade ≥3 were anemia, lymphopenia, leukopenia, and neutropenia each of which occurred in all 7 evaluable patients

Indication (Ref)	TCR-Target & Engineering	No. of Subjects Treated (Evaluable)	Efficacy	Safety
HPV-related malignancies (HNSCC, cervical, anal) (Norberg et al., 2018)	HPV-16 E7	12 (12)	ORR 6/12	TRAEs Grade ≥3. Most common AEs were lymphopenia (100%), leukopenia (100%), neutropenia (100%), anemia (100%), thrombocytopenia (67%), FN (67%), hypophosphatemia (50%) pulmonary edema (25%), hypoxia (25%) and AST increased 25%. CRS and neurotoxicity not reported.
Sarcoma or Metastatic Melanoma (HLA-A2 restricted) (Nowicki et al., 2018)	NY-ESO-1<br>TCR + DC vaccine ± anti-CTLA-4	10	ORR 2/10<br>Evidence of initial antitumor response in 4 of 6 patients treated with ESO and 2 *of* 4 patients with ipi + ESO; initial responses were incomplete and transient	No Grade 5 or other serious toxicities beyond known toxicities attributed to conditioning chemotherapy, systemic IL-2, and ipilimumab therapy. Serious toxicities were generally reversible. AEs Grade ≥3 (regardless of attribution) occurring in 2 or more patients included FN (5/10), pancytopenia (5/10), pneumonia (2/10), cytokine storm (2/10), bacteremia (1/10).

AEs = adverse events; DLT = dose-limiting toxicity; FN = febrile neutropenia; GI = gastrointestinal; GVHD = graft-versus-host disease; HLA = human leukocyte antigen; HNSCC = head and neck squamous-cell carcinoma; HPV = human papillomavirus; ORR = objective response rate; OS = overall survival; PFS = progression-free survival; SAE = serious adverse event; sCRS = severe cytokine release syndrome; TCR = T-cell receptor; TRAE = treatment-related AE.

2.4. Neoantigens As Targets for T Cells

T cells are the primary mediators of adaptive immunity. Directed by the specificity of each T cell's unique TCR, T cells regulate autoimmunity, help activate B cells and innate effectors, and directly kill infected and cancerous cells in a precisely targeted manner. Each TCR recognizes a foreign or altered self (mutated) peptide ligand presented by a major histocompatibility complex (MHC) molecule on target cells. Identification of relevant peptide-MHC ligands is the key to understanding immune responses to tumors and pathogens as well as inappropriate responses to self and dietary antigens (Schumacher and Schreiber, 2015; Sollid et al., 2015). This understanding enables clinically beneficial immunotherapies (e.g., TCR gene transfer and vaccines) that initiate, amplify, or attenuate immune responses to target antigens (Kontos et al., 2015; Rosenberg and Restifo, 2015).

Figure 1 Neoantigens: Optimal Targets for T Cell-Tumor Immunity

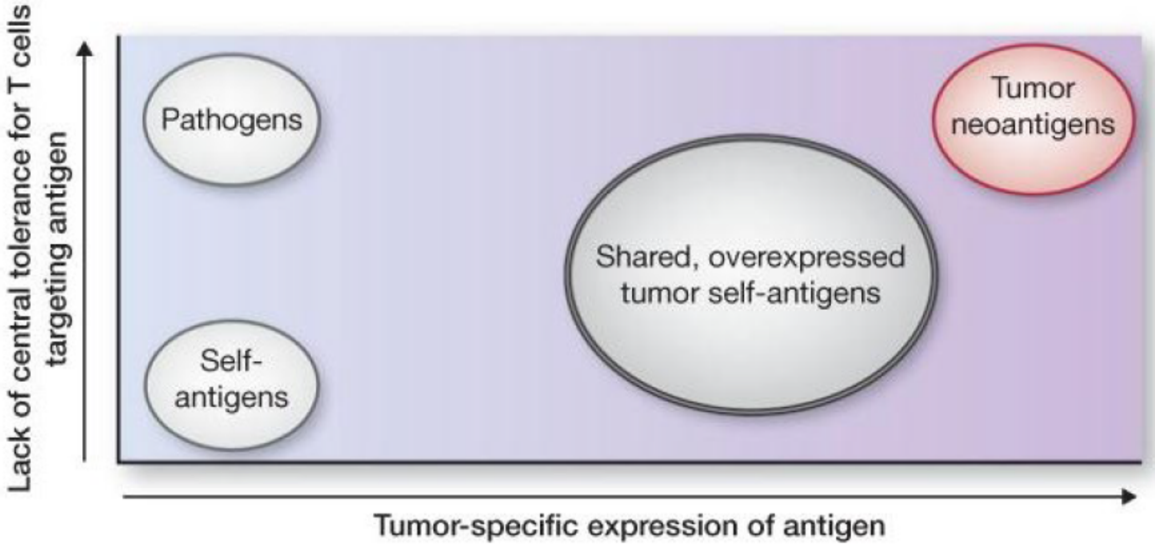

From: Hacohen et al., 2013.

Mutated "neoepitopes" are important targets of endogenous and engineered immune responses to cancer (Tran et al., 2015). Neoepitope-reactive T cells are present at the tumor site or in the peripheral blood (Robbins et al., 2013; Gros et al., 2016) and regress tumors upon adoptive transfer (Rosenberg and Dudley, 2009; Tran et al., 2014; Tran et al., 2016). Likewise, tumor mutational burden predicts the clinical effectiveness of CTLA-4 (Snyder et al., 2014; Van Allen et al., 2015) or PD-1 (Rizvi et al., 2015; Le et al., 2015; McGranhan et al., 2016) blockade, suggesting these checkpoint inhibition strategies affect tumor regression by unleashing neoepitope-reactive T cells. Because neoepitopes result from somatic mutation in tumor cells, they are not presented by thymic epithelial cells to induce central tolerance. Thus, T-cell responses directed at these neoepitopes are tumor-specific, likely high-affinity, and patient-specific (i.e., private). From a clinical standpoint, this presents an opportunity as neoepitopes are ideal targets for immunotherapy and once identified, enable personalized therapeutic application across an inclusive patient population (Figure 1).

2.5. NeoTCR-P1 T-Cell Product

The investigational agent in this protocol is NeoTCR-P1, an autologous adoptive T-cell therapy (ACT) for patients with solid cancers. NeoTCR-P1 is composed of apheresis-derived CD8 and CD4 T cells that are precision genome engineered to express autologous TCRs of native sequence that target neoepitopes presented by human leukocyte antigen (HLA) receptors exclusively on the surface of that patient's tumor cells and not on other cells in the body.

The goal for NeoTCR-P1 is to manufacture 3 distinct clonal populations of T cells for each patient (i.e., 3 TCR) whenever possible. Each T-cell clonal population will express a neoTCR that is unique to each specific patient. In participants in whom only 2 actionable neoTCRs are identified, a 2TCR product will be manufactured, and in cases where only 1 actionable neoTCR is identified, a 1TCR product will be manufactured. At a given dose level, the total number of neoTCR+ gene-edited cells administered to participants for a 1TCR, 2TCR, or 3TCR product will be the same. The term "up-to-3TCR" (abbreviated u3TCR) is used to acknowledge that the product selection process during *participant* screening may yield less than 3 actionable neoTCR candidates for product development in some trial participants.

To control the total cell dose in the u3TCR product, the number of T cells expressing each neoTCR is adjusted according to the number of TCRs in the product manufactured for that patient (see Table 13). Ideally, u3TCR products will comprise proportionate numbers of engineered cells according to the number of distinct TCRs in the product, however different manufacturing and cell expansion efficiencies for each TCR may result in non-idealized ratios (i.e., not 1:1 or 1:1:1 for 2TCR and 3TCR products, respectively).

Unlike many other genetically engineered ACT products, NeoTCR-P1 cells are generated using DNA-mediated (non-viral) precision genome engineering during Good Manufacturing Practice (GMP) manufacturing. During this single-step process, the private neoTCR is stably integrated into the patient's CD8 and CD4 T cells at a defined genomic site that results in natural neoTCR expression and regulation. Subsequently, the NeoTCR-P1 cells are expanded for several days under conditions that favor the generation of "younger" T-cell phenotypes that are most likely to engraft following reinfusion, to target and kill tumor cells, as well as to persist for ongoing cancer surveillance and control.

NeoTCR-P1 cell product will be administered by infusion after a non-myeloablative conditioning regimen into the same patient with solid tumors. The goal is to provide a complete, personalized, tumor-targeted T-cell immune response in a single dose for rapid and durable clinical benefit to patients with cancer.

The generation of NeoTCR-P1 product for each patient comprises 3 main activities: Product Selection, Plasmid Production, and Cell Manufacturing. Product Selection begins with the bioinformatic prediction of the patient's neoepitopes from sequencing data of tumor biopsies (i.e., compared to healthy peripheral blood mononuclear cells [PBMCs]) sequence information). Using PACT's proprietary imPACT isolation technology™, intrinsic neoepitope-specific CD8 T cells already present in the patient's peripheral blood are captured. The neoTCR sequences targeting the predicted tumor-specific mutations are cloned and functionally characterized for NeoTCR-P1 product selection. The personalized DNA plasmid reagent, containing the selected

neoTCR sequence, is then manufactured as the genetic engineering vector used for subsequent GMP T-cell manufacturing. NeoTCR-P1 product manufacturing begins with apheresis of the patient's blood followed by enrichment of autologous CD8 and CD4 T cells. T cells are then ex vivo precision genome engineered to express the personalized, tumor mutation-targeted neoTCR. Resulting cells are expanded for several days and cryopreserved until the patient is ready for reinfusion.

A schematic of this process is depicted in Figure 2.

Figure 2 NeoTCR-P1 Process

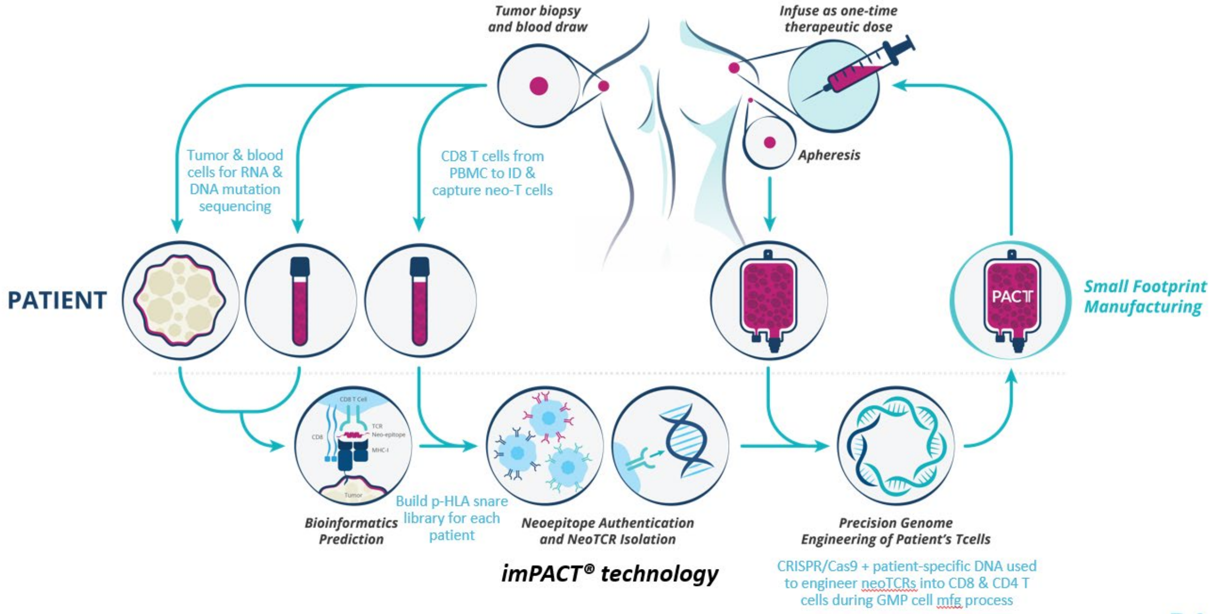

ID = identify; *GMP = Good Manufacturing Practice; mfg = manufacturing*; NeoTCR = neoepitope-specific T-cell receptor; NeoTCR-P1 = gene-edited autologous NeoTCR-T cells (i.e., compound under study)*; PBMC = peripheral blood mononuclear cells.*

A more detailed description of the neoTCR selection process is highlighted in Figure 3. Briefly, NeoTCR-P1 product selection starts with whole exome DNA sequencing (WES) and RNA transcriptome sequencing of tumor biopsies followed by the bioinformatic prediction of tumor-displayed neoepitopes presented by the patient's own HLA receptors (bioinformatics). A library of the predicted private neoepitope-HLA fusion protein candidates is generated (comPACT™) and used as "bar-coded snares" to interrogate a sample of the patient's PBMCs for neoepitope-specific CD8 T cells (imPACT Isolation Technology®). To date, the comPACT protein platform comprises 66 HLA alleles that collectively allow PACT to interrogate T cells from patients across all ethnicities possessing 4/6 HLA alleles from the catalog in >95% of the population or possessing 6/6 alleles in >80% of the population. Therefore, this catalog enables the assembly of bar-coded snare libraries to interrogate pre-existing antitumor T cells from blood and potential implementation of NeoTCR-P1 therapy in most patients with cancer. Capture of pre-existing, antigen-experienced CD8 T cells that bind to the patient's private neoepitope-HLA candidate snares confirms that the respective neoepitope is presented to the immune system and therefore likely to be expressed on the surface of the tumor cells. From each of the neoepitope-

specific CD8 T cells captured from blood, the corresponding TCR alpha and beta chain genes are cloned and sequenced (Figure 3, mutation-targeted T cells isolated from blood). From the isolated TCR sequences, only neoTCR candidates derived from previously activated, antigen-experienced T cells are processed further. This ensures that the cognate neoepitope has in fact been presented to the immune system, and this interaction resulted in epitope and HLA-specific T-cell activation, yet in the absence of autoreactivity. Antigen-experienced neoTCRs are then engineered into fresh T cells from a healthy donor source for confirmatory sequencing and functional characterization (identity, specificity, and induction of interferon-γ upon antigen specific trigger, Figure 3 box 4 and 5). The top (up to 3) neoTCR candidates are selected for product development. The corresponding neoTCR plasmids are manufactured in a GMP facility and subsequently used for GMP manufacturing of NeoTCR-P1 product.

The NeoTCR-P1 cells will be manufactured by a contract manufacturing organization or directly by the Sponsor. At the end of the cell manufacturing process, the cells are cryopreserved in infusible cryomedia and stored at the appropriate temperature until reinfusion.

Figure 3 NeoTCR-P1 Product Selection Process

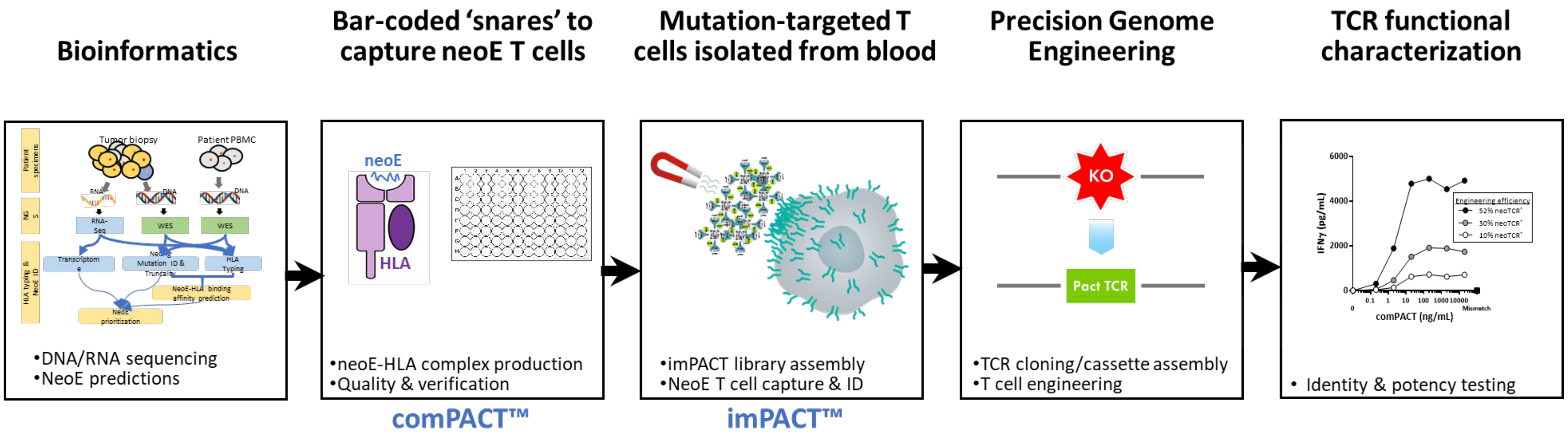

HLA = human leukocyte antigen; NeoE = neoepitope; NGS = next generation sequencing; PBMC = peripheral blood mononuclear cell; TCR = T-cell receptor; WES = whole exome sequencing.

Figure 4 summarizes results from earlier studies demonstrating that the imPACT technology™ processing of samples yielded neoepitope-specific TCRs across different cancer types and HLA-types.

Figure 4 Summary of Isolated Neoepitope-Specific T cell and Cognate neoTCRs

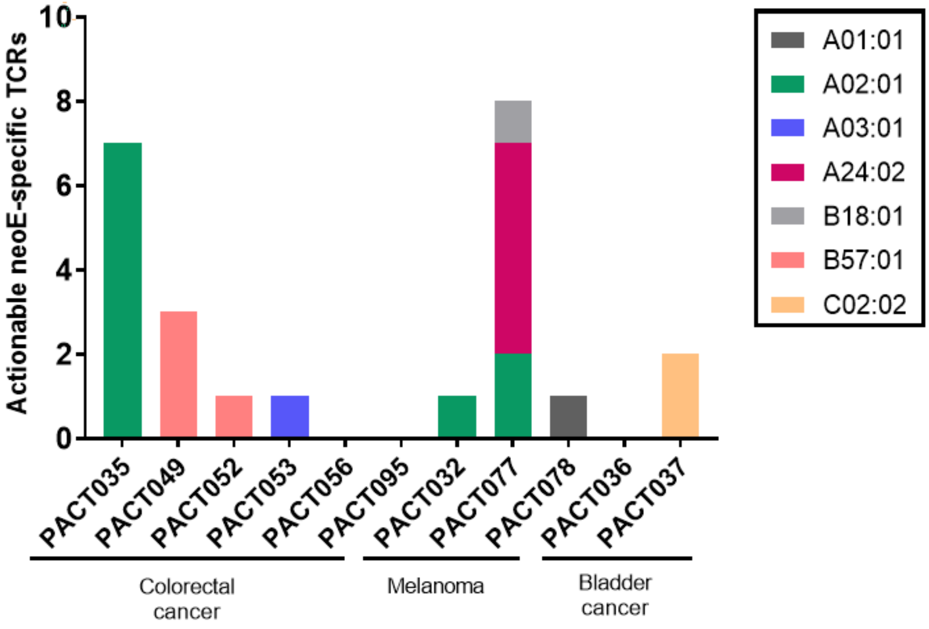

* TCRs from these patient samples are still under validation (PACT036, 131, and 133).

Note: imPACT processing of PBMCs from 11 patient samples yielded 24 neoepitope-specific T cells across 3 cancer types that recognize the neoepitope peptide-complexed to 7 HLA types. All blood samples were obtained from treatment-naïve patients with cancer. Three patient specimens (PACT036, *056*, and 095) did not yield any neoepitope-specific T cells upon completion of the imPACT process. Neoepitope-specific T cells confirmed to bind multiple neoepitope HLA targets per patient were isolated from each of the following 4 samples: PACT035, 037, 049, and 077

A total of 11 patient samples from 3 different cancer types have been analyzed: CRC (6), melanoma (3), and bladder cancer (2). All of these patients were treatment naïve at the time of PBMC sample collection. Neoepitope-specific T cells with actionable neoTCRs upon confirmatory functional characterization have been captured from the peripheral blood PBMCs from 8 of those patients: CRC (4), melanoma (3), and bladder cancer (1) (Figure 4). These results indicate that circulating neoepitope-specific T cells were effectively captured from the peripheral blood of patients with solid tumors, even from treatment-naïve patients.

Neoepitopes derived from tumor-exclusive expressed neoantigen targets that were recognized by these T cells were complexed to a total of 7 different HLA alleles across all 3 class I HLA loci. Four patient samples (PACT032, 052, 053, and 078) yielded a single unique actionable neoTCR sequence at the end of the imPACT process, while the other 4 patient samples (PACT035, 037, 049, and 077) yielded multiple actionable TCRs per patient at the end of the imPACT process.

2.6. Summary of Nonclinical Studies Supporting NeoTCR-P1

Published studies have shown proof-of-concept that adoptive transfer of neoantigen-specific TCR engineered T cells can be effective in mouse tumor models. For example, human T cells transduced with the neoepitope-specific TCR significantly reduced tumor growth and enhanced survival in a NOD/SCID mice engrafted with colorectal cell lines expressing the mutated neoantigen (Indergerg et al., 2017). Furthermore, mouse T cells engineered to express a TCR targeting the shared antigen MART-1 led to the rejection of large, well-established solid tumors (Leisegang et al., 2016). However, animal models testing fully human T cells targeting bona fide patient-specific neo-epitope mutations are not relevant, since the display of epitopes by human HLA molecules to the circulating T cells is critically dependent on multiple factors, such as the protein expression profile in a particular cell, the antigen processing machinery that processes peptides from cell-expressed proteins for loading onto HLA receptors in the endoplasmic reticulum, and the patient-specific repertoire of HLA molecules expressed on the cell surface. Each person expresses up to 6 HLA clonotypes (depending on hetero- or homozygosity of encoded HLA A-, B-, and C-genes in each person) of the >13 thousand HLA allele types present in the human population. Since these HLA repertoires are so highly diverse across individual humans, it is improbable to anticipate that any model cell or animal system would recapitulate the HLA repertoire or antigen processing conditions that would be relevant to the patient tumor. Furthermore, a rigorous *in vivo* assessment of human neoTCR T-cell proliferation, persistence, and function requires additional human-specific immune accessory signals beyond those conferred by implanted xenograft tumors, such as cytokines, chemokines, receptor signaling, and patient-specific tumor that are absent in immunodeficient animal models. Hence, studies with rodent, large animal, or non-human primate animal models or studies with xenograft human cell lines are not objectively relevant to assess the potential for off-target/on-target neoTCR T-cell binding and signaling cascades. None of these models, and thus only the clinical studies, would properly expose the consequences of personalized adoptive neoTCR T-cell transfer into the same cancer patient from whom the neoepitope-specific T cells were originally isolated.

Therefore, the pharmacology and safety related studies performed under this Investigational New Drug Application (IND) and proposed to support clinical trials of NeoTCR-P1 adoptive cell therapy, comprise *in vitro* (molecular) and ex vivo human T-cell assays only. The nonclinical overview given here summarizes the IND-enabling studies performed to demonstrate the safety/accuracy of PACT's precision genome engineering process and of neoTCR-Targeting, as well as describe the specificity and functionality of the generated NeoTCR-P1 T cells.

Mutation-targeted, personalized, adoptive, T-cell receptor (NeoTCR-P1) therapy is an immunotherapy modality designed to unleash the immune system's ability to specifically recognize and kill cells displaying tumor-exclusive mutational targets. Figure 5 demonstrates that neoTCR-expressing T cells produced with PACT's ex vivo manufacturing process show potent antigen-specific killing and proliferative activity on contact with cognate neoantigen-expressing tumor cells *in vitro*. Importantly, no target cell killing, or neoTCR T-cell proliferation was observed following contact with target cells not displaying the HLA-bound mutated target peptide. Our data indicate that PACT's neoTCR-expressing CD8 and CD4 T cells constitute highly active tumor killing lymphocytes with the potential to eradicate tumor cells throughout the body.

Figure 5 Specific Killing of Antigen-Expressing Surrogate Tumor Target Cells and Antigen-Specific Proliferation of NeoTCR-P1 T Cells

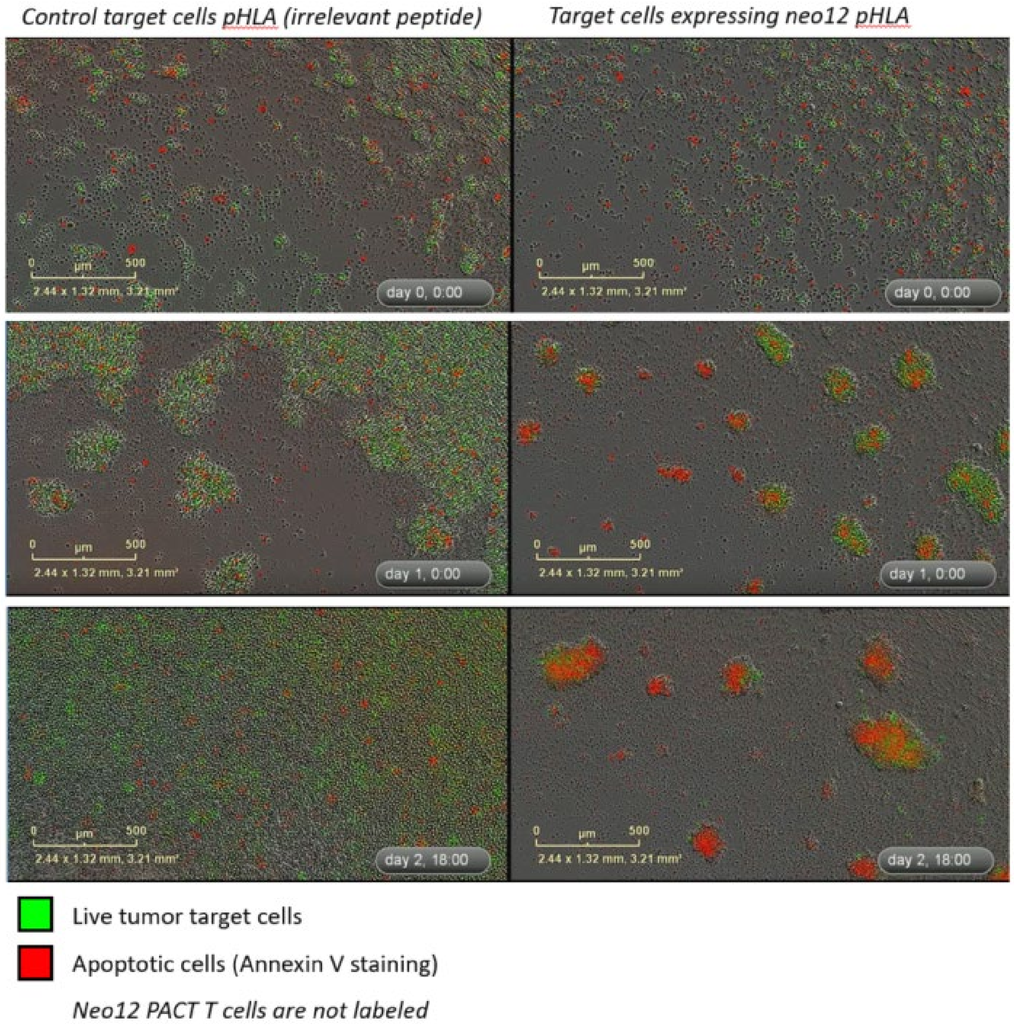

Representative images obtained with time-lapse live microscopy (Days 0, 1, and 2) show antigen-specific cytotoxic activity and proliferation by Neo12-TCR-T cells co-cultured with target cells expressing cognate Neo12 peptide-HLA (right column), but not when co-cultured with target cells expressing an irrelevant peptide (left column). Neo12 is a PACT neoTCR. Tumors not displaying the appropriate antigen continue to grow in the presence of Neo12-TCR-T cells (left column). In contrast, the majority of Neo12 antigen displaying tumor cells are apoptotic or dead within 2 days (right column). T cells are not labeled in this experiment, but antigen-specific proliferation can be appreciated visually by increased numbers of T cells over the course of 2 days (right column).

PACT's manufacturing process was developed using healthy donor leukopaks as source material. To ensure that PACT's manufacturing process can successfully generate product from T cells in patients with cancer, ex vivo mechanism-of-action (MOA) studies were performed in which the activity of NeoTCR-P1 T cells derived from PBMCs of patients with cancer was compared to the activity of T cells isolated from PBMCs obtained from healthy donors expressing the same neoTCR. Antigen-specific activity was characterized by incubating neoTCR T cells with surrogate tumor cells expressing cognate or irrelevant HLA-peptide complexes (see Figure 6). Comparable gene editing efficiencies and functional activity, as measured by antigen-specific

T cell killing, proliferation, and cytokine production, were observed with neoTCR-expressing CD8 and CD4 T cells from patients with cancer and from healthy donors (see Figure 6). No target cell killing or neoTCR T-cell proliferation was observed upon contact with target cells not displaying the HLA-bound mutated target peptide, thus demonstrating the specificity of the response.

Figure 6 Antigen-Specific Activity of Precision Genome-Engineered Human NeoTCR T Cells – Comparable Functional Activity of T Cells derived from Patients with Cancer or Healthy Donors

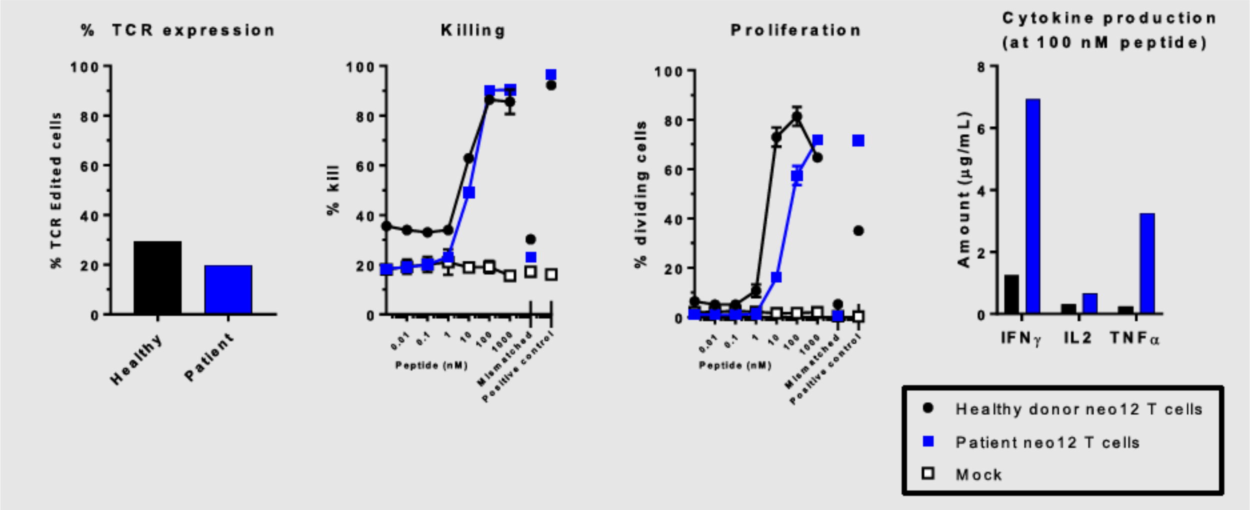

NeoTCR-P1 T cells expressing the Neo12 TCR were generated from blood of a patient with cancer or healthy donors showed comparable precision genome engineering efficiency (percentage of TCR-edited T cells expressing the neoTCR), and functional activity as measured by target cell killing, proliferation, and cytokine production. No activity was observed when mock control T cells were used.

A polyfunctional phenotype, defined as a single T cell secreting multiple effector proteins upon encounter of cognate antigen, has been shown to be an important parameter when characterizing engineered T cells administered in clinical trials. Patients with non-Hodgkin's lymphoma (NHL) dosed with CD19 CAR-T cells exhibiting polyfunctionality prior to infusion were significantly more likely to experience an objective clinical response than patients dosed with engineered CAR-T cells that did not exhibit polyfunctionality (Rossi et al., 2018). Dose-dependent polyfunctional secretion of cytokines from activated CD8 NeoTCR-P1 T cells was demonstrated by single cell secretome analysis (see Figure 7). These data also reveal that CD4 T cells engineered to express an MHC class I-restricted TCRs successfully exhibit effector function after exposure to cognate neoepitope-HLA target.

Figure 7 Single Cell Analysis of NeoTCR-P1 T Cells Reveals Polyfunctional Phenotype

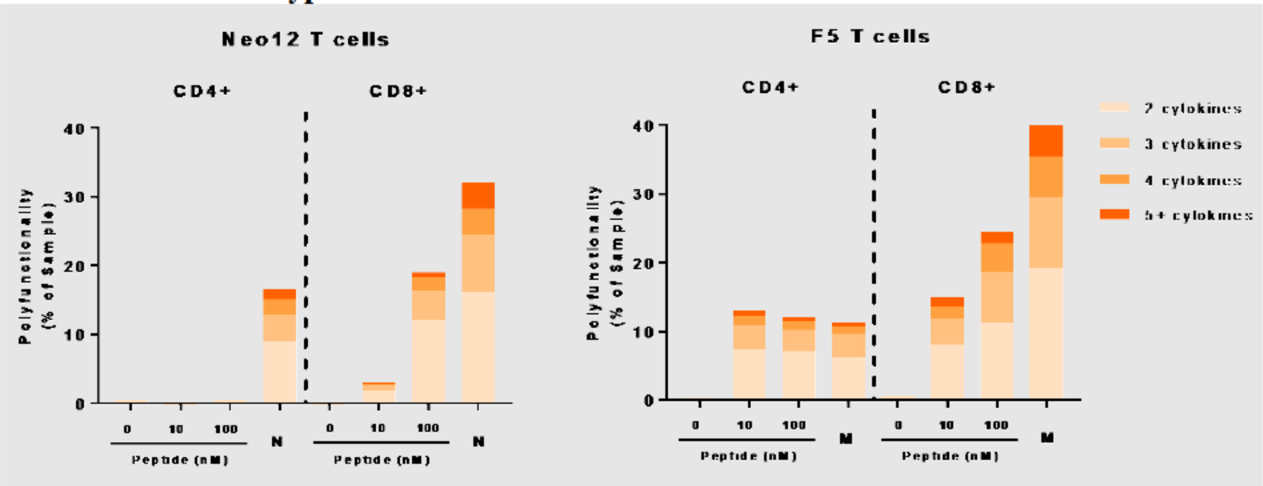

Graphs showing the percentage of CD4$^+$ and CD8$^+$ T cells engineered to either express 2 tumor-specific TCRs (i.e., neo12 or F5 TCR) that secrete 2, 3, 4, or more than 5 cytokines (shades of orange) when exposed to the cognate neoepitope-HLA target antigen. Cells were co-cultured with target cells pulsed with no peptide, 10 nM, or 100 nM specific peptide or with target cells constitutively expressing pHLA on their surface (i.e., N: Neo12 HLA-A2 cells; M: MART1 HLA-A2 cells, respectively). Secreted cytokine levels were assessed after 24 hours of co-culture. Cells secreting 2 or more cytokines (or effector molecules such as granzyme B and perforin) are defined as polyfunctional T cells.

Published reports from animal models and clinical studies have suggested that adoptive cell therapies comprising T cells with "younger" memory stem cell phenotypes achieve an improved overall response and clinical outcome than studies where "older" T effector cells were administered. Stemberger et al., (2014) have demonstrated in a mouse model of listeria infection that the adoptive transfer of even a single antigen-specific CD62L^{high} memory CD8 T cell could develop into fully protective diverse T-cell progenies (Stemberger et al., 2014). Data from mouse models and in clinical trials of CD19-targeted CAR-T cells demonstrated a correlation of the younger phenotype with cell persistence and overall response rate (Klebanoff et al., 2012; Sabatino et al., 2016; Busch et al., 2018). Thus, the administration of "younger" T-cell phenotypes is of potentially higher benefit to patients with cancer, being associated with improved engraftment potential, prolonged persistence post infusion, and rapid differentiation into effector T cells upon exposure to their cognate antigen. These properties highlight the value of infusing neoTCR engineered T cells of the predominant T memory stem cell (T_{msc}) and T central memory (T_{cm}) phenotypes, as observed in the NeoTCR-P1 T-cell product profile.

The composition of T-cell phenotypes resulting from the NeoTCR-P1 cell manufacturing process was interrogated by flow cytometric analysis (see Figure 8). NeoTCR-P1 cells of memory stem cell and central memory phenotypes represent the predominant T-cell phenotypes from PACT's *ex vivo* manufacturing process.

Figure 8 NeoTCR-P1 T Cells are Mainly of the "Younger" Tscm and Tcm Phenotype

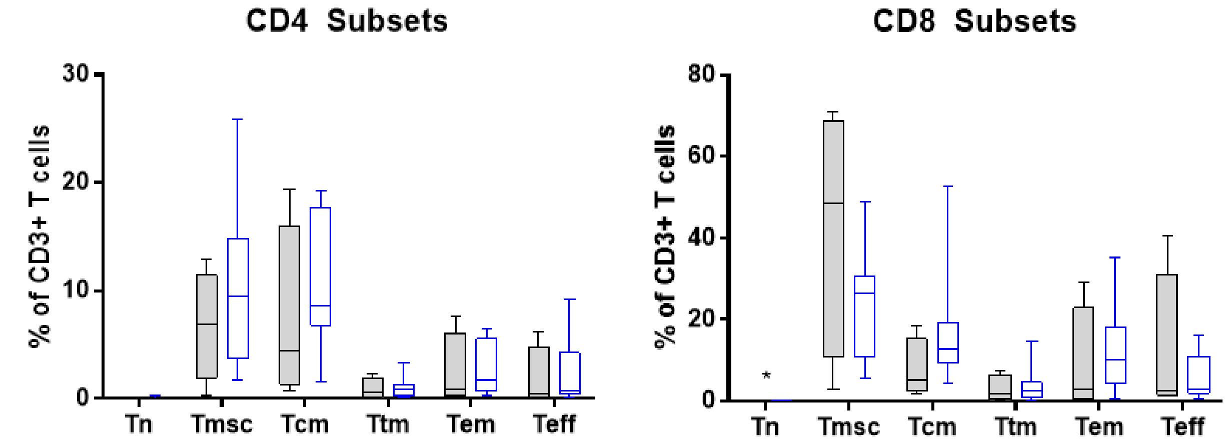

	Tn	Tmsc	Tcm	Ttm	Tem	Teff
CD45RA	+	+	-	-	-	+
CD62L	+	+	+	-	-	-
CD27	+	+	+	+	-	+/-
CD95	-	+	+	+	+	+

CD4 (left panel) and CD8 subset distribution after laboratory-scale manufacturing from blood of healthy donors (black) or patients with cancer (blue). CD4 T cell and CD8 T cell subset phenotypes in the final product are predominantly T_{msc} and T_{cm} (both populations are $CD62L^{high}$). Tn subsets are not visible on the graph as percentages are <1% after activation of T cells during manufacturing process.
* $p<0.05$. Healthy donors: n=4 from 3 unique donors; patients: n= 14 from 8 unique donors.
Gating strategy: single cells, live cells, CD3+ cells, phenotype subsets. Markers used for subset distribution definition and analysis are indicated below the graphs.

Together, these *ex vivo* MOA studies demonstrate that the majority PACT NeoTCR-P1 T cells generated from healthy donors or patients with cancer consists of CD8 and CD4 T cells of the desired younger phenotype subsets (T_{msc} and T_{cm}). Upon encounter of cognate peptide-HLA these cells rapidly transition into polyfunctional effector cells that demonstrate potent antigen-specific cytokine production, tumor killing activity, and proliferative capacity, with the potential to eradicate tumor cells throughout the body.

2.7. Nivolumab

The PD-L1/PD-1 pathway is involved in peripheral immune tolerance. Nivolumab is a potent and highly selective humanized monoclonal antibody of the IgG4/kappa isotype designed to directly block the interaction between PD-1 and its ligands PD-L1 and PD-L2. Blockade of their targets by these antibodies restores functional activity of T cells in situ and, thus, facilitate tumor regression but may also increase the risk of immune-mediated adverse events, specifically, the induction of autoimmune conditions. Adverse events with potentially immune-mediated causation including rash, hypothyroidism, hyperthyroidism, adrenal insufficiency, Type 1 diabetes mellitus, pancreatitis, hepatitis, pneumonitis, colitis, meningoencephalitis, myasthenia gravis/myasthenic syndrome, and Guillain-Barré syndrome have been observed in clinical studies with nivolumab.

Nivolumab is approved for multiple indications, including patients with previously treated metastatic NSCLC, melanoma, advanced renal cell cancer (RCC), and small cell lung cancer

(SCLC) that has progressed after 2 or more lines of therapy; recurrent or metastatic HNSCC on or after platinum-based therapy; hepatocellular carcinoma (HCC) treated with sorafenib; patients with previously treated or locally advanced or metastatic urothelial carcinoma; and MSI-H/dMMR metastatic CRC. Nivolumab is approved for dosing every 4 weeks (480 mg Q4W) across the above indications. Nivolumab has very limited single-agent activity in patients with metastatic prostate cancer, HR+ breast cancer, MSS stable CRC, and ovarian cancer and will be administered as an investigational agent in this study.

For further details regarding clinical efficacy and safety, including a detailed description of potential safety risks for nivolumab, refer to the United States product insert (USPI) or Summary of Product Characteristics (SmPC).

2.8. Adoptive T-Cell Therapy (ACT) in Combination with Checkpoint Blockade

While CAR-T cell therapy has altered the paradigm for treating hematologic malignancies and is an emerging approach in some solid tumor indications, many patients exhibit suboptimal initial response or have responses that wane over time. Adoptively transferred T cells may be susceptible to immune inhibition and must execute cytolytic and proliferative function in a hostile tumor microenvironment that may harbor immune inhibitory signals. Strategies that involve checkpoint inhibitor therapy to augment the immune response may extend the durability and function of T cells that express PD-1. Recent experiments have demonstrated the restoration of effector function of CAR-T cells *in vitro* and *in vivo* following PD-1 blockade (Wherry et al., 2007; Cherkassky et al., 2016; Rupp et al., 2017).

The clinical experience to inform the safety and efficacy of CAR-T cell therapy in combination with checkpoint inhibitors is rapidly accumulating. A recent single-patient report of a patient with relapsed diffuse large B-cell lymphoma (DLBCL) suggests that PD-1 inhibition with pembrolizumab following CAR-T cell therapy may have activity and be well tolerated in patients who fail to respond to CAR-T cell therapy alone (Holtzman et al., 2019). A larger study of 12 patients with progressive or relapsed NHL, whose immediate prior therapy was *treatment with* CD19-targeted CAR-T *cells,* enrolled patients to pembrolizumab (Chong et al., 2018). Of the 11 patients with DLBCL and 1 patient with follicular lymphoma, 9 had progressive disease at a median of 3.3 months following receipt of CAR-T therapy. Eight of 11 patients demonstrated re-expansion of CAR-T cell populations after receiving pembrolizumab. The combination was well tolerated with adverse events considered possible related including Grade 3/4 neutropenia (3 patients) and 2 cases of Grade 1 and 3 cytokine release syndrome (CRS). The best overall response was 25% and include 1 CR. A second study evaluated either pembrolizumab or nivolumab in pediatric patients with relapsed/refractory *B-cell acute lymphoblastic leukemia (*B-ALL*)* treated with CD19-directed CAR-T-cell therapy. In 4 patients with bulky extramedullary disease, 2 partial and 2 CRs were observed. In a second cohort of 6 patients with poor persistence of CAR-T cell therapy, pembrolizumab was associated with the reoccurrence of B-cell aplasia (a signal of CAR-T cell function). CRS symptoms and fever were observed in 3/14 patients within 2 days of starting pembrolizumab. Other potential treatment-related adverse effects associated with PD-1 inhibition included 1 case each of acute pancreatitis, hypothyroidism, arthralgias, urticaria, as well as 4 patients with Grade 3–4 cytopenias. No Grade 5 toxicities or graft-versus-host disease flares occurred (Li et al., 2018). A third study of 15 patients treated with the combination of durvalumab (either administered 1 day prior or

21-28 days after CAR-T) and CD19 CAR-T cell therapy (JCAR014) is ongoing (Hirayama et al., 2018). Preliminary data has demonstrated that the combination is safe with some demonstrated CRs in patients initially failing to achieve CR.

Multiple trials are exploring the combination of checkpoint inhibitors with CAR-T cell therapy (ClinicalTrials.gov identifiers NCT02026833, NCT03310619, NCT03630159).

2.9. Interleukin-2 (Aldesleukin Recombinant Interleukin 2)

Approximately 10-20 participants in one of the Phase 1a basket-expansion cohorts will receive IL-2 (aldesleukin [Proleukin®]) to evaluate its effect on the expansion of neoTCR T-cells post-adoptive transfer (Section 4.2.6).

IL-2 is a cytokine that promotes the survival, proliferation, and differentiation of activated T cells. It is commercially available as aldesleukin (Proleukin®), a human recombinant product. IL-2 is approved for the treatment of metastatic RCC and melanoma. Toxicity from IL-2 at the approved dose (high-dose IL-2; 600,000 IU/kg administered every 8 hours by intravenous infusion for a maximum of 14 doses) can manifest in multiple organs, most prominently the heart, lungs, kidneys, and central nervous system (CNS). Proleukin® administration has been associated with capillary-leak syndrome (CLS) which is characterized by a loss of vascular tone and fluid accumulation in the extravascular space. CLS results in hypotension and reduced organ perfusion which may be severe and can be fatal. Proleukin® treatment is also associated with impaired neutrophil function and an increased risk of infection, including sepsis and bacterial endocarditis.

For further details regarding clinical efficacy and safety, including a detailed description of potential safety risks for aldesleukin, refer to the Proleukin® United States Prescribing Information (USPI).

2.10. Interleukin-2 as Adjunctive Therapy with ACT

Early studies of ACT in a tumor model system demonstrated that infusion of lymphokine-activated killer (LAK) cells together with IL-2 was 50-100x more effective in mediating tumor regression than LAK cells alone (Rosenberg et al., 1985). Since the time of this observation, studies of adoptive immunotherapy with TILs or engineered TCR T cells have used high-dose IL-2 in order to improve the persistence and activity of the transferred cells. High-dose intravenous bolus IL-2 (600,000 to 720,000 IU/kg every 8 hours) is normally given to tolerance with a maximum of about 15 doses total (Rosenberg et al., 1994), with the injunction to stop IL-2 at the onset of Grade 3 or 4 toxicity that is not easily reversible. In general, the reported toxicities of the ACT regimens are similar to those attributed to the IL-2 and lymphodepletion regimens (Table 11).

High-dose IL-2 as a single agent is approved in 2 indications (i.e., metastatic melanoma and RCC) where it produces response rates <20%. Responses to single-agent IL-2 in other tumor types, including those under study in PACT-0101 (i.e., colorectal, breast, ovarian, prostate, and bladder cancer), have been reported, but only rarely (Rosenberg et al., 2014).

Table 11 *Cell* Therapy with IL-2

Indication (Ref)	No. of Subjects Treated (Evaluable)	IL-2 Regimen	Efficacy	Safety
Solid tumors (melanoma, RCC, BC, CRC) / *TIL* (Topalian et al., 1988)	12	10K-100K U/kg TID to tolerance	2 PR (melanoma and RCC)	Toxicities consistent with IL-2 profile; none attributed to TILs
Melanoma / *TIL* (Rosenberg et al., 2014)	86	720,000 IU/kg q8h	ORR 34%	Transient side effects easily managed
Melanoma / *TIL* (Dudley et al., 2005)	35	720,000 IU/kg q8h to tolerance	ORR 51%	No Grade 3/4 toxicities attributable to administration of TILs; febrile neutropenia (37%), transient toxicities associated with IL-2
Cervical carcinoma / *TIL* (Jazaeri et al., 2019)	27	600,000 IU/kg up to 6 doses	ORR 44.4%	Consistent with underlying disease and profile of lymphodepletion and IL-2 regimens; anemia (55.6% Grade 3/4), thrombocytopenia (44.4% Grade 3/4), neutropenia (29.6% Grade 3/4), hypotension (14.8% Grade 3/4)
Melanoma / *TIL* (Sarnaik et al., 2018)	66	600,000 IU/kg up to 6 doses	ORR 38%	Consistent with underlying disease and profile of lymphodepletion and IL-2 regimens; thrombocytopenia (80.3 % Grade 3/4), anemia (54.5% Grade 3/4), febrile neutropenia (53% Grade 3/4), neutropenia (37.9% Grade 3/4), hypotension (10.6% Grade 3/4)
Synovial cell sarcoma & melanoma / NY-ESO-1 engineered TCR (Robbins et al., 2011)	*17*	*720,000 IU/kg q8h to tolerance*	*5/11 PR (melanoma) 4/6 PR (sarcoma)*	*Transient neutropenia and thrombocytopenia from preparative regimen and transient IL-2 toxicities*
HPV-associated epithelial cancer / HPV16 E6 engineered TCR (Doran et al., 2019)	*12*	*720,000 IU/kg q8h to tolerance*	*2/12 PR*	*Grade 3/4 cytopenias secondary to conditioning regimen, expected IL-2 toxicities resolved with discontinuation*

BC = breast cancer; CRC = colorectal cancer; IL-2 = interleukin 2; ORR = objective response rate; *PR = partial response; RCC = renal cell cancer; Ref = reference*; TIL = tumor-infiltrating T cells; *q8h = every 8 hours.*

High-dose IL-2 has been used extensively with ACT (Table 11), including with genetically engineered T-cell therapy (Doran et al., 2019). It has been administered with total engineered cell doses several orders of magnitude greater than the highest doses proposed to be evaluated in this study (e.g., Doran et al., 2019 where subjects received 1-2 × 10^{11} genetically engineered T cells followed by high-dose IL-2). The toxicity profile has been predictable and manageable across the published experiences, with no reported association between cell dose and the severity of IL-2-associated adverse events.

In order to potentially mitigate IL-2 related toxicity, PACT *initially* proposes to use low dose IL-2 (i.e., 500,000 IU/m^2 administered subcutaneously twice daily) because of data which

indicates that IL-2 receptors are saturated at low doses of IL-2 and that adoptively transferred T-cell clones can persist *in vivo* in response to low dose IL-2, preferentially localize to tumor sites and mediate an antigen-specific immune response characterized by the elimination of antigen positive tumor cells and regression of individual metastases (Yee et al., 2002, Mackensen 2006) (see Section 4.2.6 regarding further rationale for low dose IL-2). *Following review of safety and PK data from the first 6 participants in the IL-2 basket expansion cohort, the SRT may recommend evaluating a more intensive IL-2 regimen, not to exceed a customary high-dose regimen (600,000 IU/kg IV every 8 hours to a maximum of 15 doses and a maximum of 5 doses when given in the Phase 1b portion of the study in combination with Nivolumab, e.g., Jazaeri et al., 2019, Sarnaik et al., 2018, Chatzkel et al., 2019).*

2.11. Benefit/Risk Assessment

NeoTCR-P1 is not approved and clinical development is ongoing. Participation in this study will expose participants to genetically engineered autologous T cells.

Based upon the experience of autologous stem cell transplant across a number of indications, the risk of the infusion of unedited autologous cells alone is generally low and directly related to the types of high-dose conditioning chemotherapy used (Burt et al., 2010). Participants in this study will receive a nonmyeloablative conditioning regimen that is associated with less cytopenias (but significant lymphopenia) and more rapid immune reconstitution than myeloablative regimens (Alyea et al., 2005).

There is limited clinical experience with gene-edited TCR-T cells. Many of the short-term risks, such as CRS and neurotoxicity (described in Table 12), are based upon the clinical experience with CAR-T cells that contain artificial receptors (i.e., scFv) and costimulatory domains (e.g., CD28 or 4-1BB) that alter the natural kinetics of T-cell expansion and contraction. In addition, the majority of the CAR-T studies have occurred in patients with hematologic malignancies where the rate at which CAR-T cells engage tumor may be different than solid tumors. The incidence, severity, and kinetics of these potential adverse events in patients with solid tumor treated with gene-edited TCR T cells are limited and may differ from those observed with CAR T cells.

The unknown long-term risks of gene-edited T cells are related to the potential for expression of mixed TCR species arising from incomplete deletion of both chains of the endogenous TCR together with expression of the neoTCR species. Although a rare event for dual TCR species to be expressed among all of the engineered T cells in the NeoTCR-P1 product, there is the formal possibility that a mixed population of dual α and β chain TCR polypeptides may bind to unexpected peptide-HLA targets with the potential to result in autoimmune sequelae similar to graft-versus-host disease (GVHD). In the risk assessment, the following factors were identified:

- Neoantigens are tumor-exclusive mutant proteins, so targeting of neo-epitopes by neoTCRs should be restricted exclusively to tumor cells
- The neoTCR is the native TCR sequence cloned from antigen-experienced individually captured T cells from the same *participant* prior to dosing

- NeoTCR-P1 T cells are produced by precision genome engineering ex vivo using Cas9 nuclease ribonucleoproteins introduced during the manufacturing process, which limits the duration of *ex vivo* T-cell exposure to gene-editing reagents
- Plasmid DNA is used as the template for genome engineering, rather than viral vectors, eliminating the potential that viral sequences may alter T-cell function
- The neoTCR sequence is precisely integrated into the TCRα genomic locus, tightly controlling the copy number, which limits the potential for insertional mutagenesis
- Personalized adoptive T-cell receptor (NeoTCR) expression is under control of the endogenous TCR promoter, which, with the elimination of competing endogenous TCR chains, results in expression levels equivalent to natural TCR expression levels for each person
- The resultant engineered T cells exhibit "native" properties of T-cell regulation of function, proliferation, and persistence
- Plasmid DNA does not harbor any promoters for human-gene expression, significantly reducing the potential to drive ectopic expression from off-target integration, which limits the potential for insertional mutagenesis
- Gene-edited neoTCR sequence expressed by engineered autologous T cells is of native sequence to the individual from whose CD8 neoepitope-specific T cells the neoTCR sequence was cloned, and therefore should not elicit a neutralizing antibody-mediated or neutralizing cell-mediated immune response
- The 2A linker, the sole nonnative peptide sequence, is expressed as part of the neoTCR gene cassette within the cell but is not displayed on the surface of the engineered T cells, avoiding antibody-mediated responses. 2A peptides have been used in previous cell therapies without observed issues or immunogenicity (Arber et al., 2013)
- Binding of neoTCRs is restricted to recognition of peptide-HLA complexes expressed on the surface of specific target cells, wherein T-cell activation is naturally triggered and naturally terminated (in contrast to antibody-mediated binding of targets, as by CAR-T cells)
- T cells engineered to disrupt the endogenous TCR without concomitant introduction of neoTCR sequences are still autologous T cells for that *participant*, yet lack ability to persist in the absence of TCR signaling, which is critical for long-term T-cell maintenance
- Potential for expressing mixed TCR species, while low, may arise from incomplete disruption (mediated by CRISPR/Cas9) of both chains of the endogenous TCR in cells that also express the neoTCR species. Mixed TCR species hold the potential to bind to unexpected peptide-HLA targets that could result in autoimmunity similar to GVHD. However, the minor population of cells with randomly-paired TCR chains are unlikely to have functional targets in the patient, particularly compared with other therapies wherein no endogenous TCR removal is executed prior to administration of those therapies.
- Given the fact that native neoTCR sequences are being integrated into the genome of T cells ex vivo and are under endogenous cell regulation and control (in contrast to

CAR-T cells where the signaling domain is engineered without shut-off mechanisms), the overall risk of delayed adverse events should be low.

The potential safety risks for participants in this study are outlined below (see Table 12). This is the first study in which NeoTCR-P1 will be administered either alone or in combination with nivolumab. The following information is based on the anticipated MOA results from other gene-edited TCR-T therapies and published data on similar molecules (e.g., CAR-T, TIL therapy).

Participants in this study may receive up to 3 distinct gene-engineered T-cell species in the NeoTCR-P1 product. The potential risks from administering a polyclonal product are not believed to be meaningfully greater than those associated with the single-clonal neoTCR product. Specifically, the chance of generating mixed TCR species that could result in autoimmunity should not be increased by the additional number of neoTCRs in the product. All gene-engineered T cells in a product are expected to exhibit natural properties of proliferation and persistence, and participants who receive more than 1 neoTCR are not believed to be at significantly greater risk of adverse events than have been observed with adoptive cellular therapy (e.g., CRS, hemophagocytic lymphohistiocytosis [HLH] or neurotoxicity; see Table 12) compared to those who receive only 1 at the same total gene-edited cell dose. Nevertheless, to account for any potential increased risk associated with the administration of a polyclonal TCR product, dose escalation will proceed conservatively, and only participants who received a 3TCR product will be counted towards clearing a given dose level.

More detailed information about the known and expected benefits and risks and reasonably expected adverse events of NeoTCR-P1 may be found in the NeoTCR-P1 Investigators' Brochure.

Table 12 Risk Assessment

Potential Risk of Clinical Significance	Summary of Data/Rationale for Risk	Mitigation Strategy
Myelosuppression	• Risk of myelosuppression and prolonged cytopenias related to conditioning chemotherapy (ELIANA study of Kymriah, Grade ≥3 cytopenias not resolved by Day 28 included neutropenia (40%) and thrombocytopenia (27%) among 52 responding patients<br>• JULIET study of Kymriah, Grade ≥3 cytopenias not resolved by Day 28 included thrombocytopenia (40%) and neutropenia (25%) among 106 treated patients)	• *Participants* in monitored setting with frequent assessment of CBCs<br>• *Participants* will receive transfusion of blood products per institutional guidelines<br>• Prolonged cytopenias (not resolved by Day 28) are included as an *AESI*
Febrile reaction	Transient fever and chills	• An evaluation for infection will be initiated, and *participants* will be managed appropriately with antibiotics, fluids, and other supportive care as medically indicated and per the discretion of the treating physician and following institutional guidelines<br>• In the event the *participant* develops sepsis or bacteremia following NeoTCR-P1 infusion, appropriate cultures, antibiotics, and medical management should be initiated<br>• If a contaminated NeoTCR-P1 T-cell product is suspected, the product will be retested for sterility using archived samples that are stored in the CPF
Febrile Neutropenia	Risk of febrile neutropenia related to conditioning chemotherapy	• *Participants* will be monitored while an inpatient for signs and symptoms of infection<br>• Local institutional guidelines with appropriate cultures, antibiotics, and medical management will be followed for evaluation of any suspected infection
Acute infusion reaction	Transient fever, chills, nausea, and rigors are expected.	• Acetaminophen and diphenhydramine will be administered as premedication and may be repeated every 6 hours, if needed<br>• Non-steroidal anti-inflammatory medication may be prescribed if the *participants* continue to experience symptoms

Potential Risk of Clinical Significance	Summary of Data/Rationale for Risk	Mitigation Strategy
Hypersensitivity reaction	Serious hypersensitivity reactions, including anaphylaxis, may be due to the DMSO	• Acetaminophen and diphenhydramine will be administered as premedication<br>• NeoTCR-P1 will be administered in a monitored setting with pulse oximetry and telemetry being available<br>• Slowing rate of infusion
Cytokine release syndrome (CRS)	• CRS is related to T-cell expansion and has been observed in patients after treatment with CAR-T cells and gene-edited TCRs. Severity has ranged from mild to life-threatening (requiring ICU admission, intubation, dialysis, etc.) and fatal in some cases. Disease burden predictive of severe CRS<br>• Incidence of Grade ≥3 CRS in CD19 CAR-T studies has ranged from 49% (ELIANA Study in childhood ALL) to 13% (ZUMA-1 Study in adult DLBCL)<br>• Concurrent neurotoxicity and overlap with HLH/MAS has also been reported<br>• Symptoms typically occur 1–14 days after CAR-T-cell infusion	• Study conducted with investigators at centers experienced with the administration of cellular therapies and management of CRS<br>• Inpatient hospitalization/observation for initial infusion of NeoTCR-P1 with first *participant* in each dose-escalation cohort staggered by 7 and 14 days in the 1a and 1b, respectively<br>• *Participants* and caregiver counseled on CRS symptoms and to return to clinic if any symptoms occur<br>• *Participants* will monitor fever daily for the first 28 days<br>• CRS has been abrogated in many cases with anti-cytokine directed therapy including tocilizumab and steroids<br>• Protocol includes CRS management guidelines (modified CARTOX) and sites will be required to have tocilizumab available on site and access to siltuximab prior to administration of NeoTCR-P1
Hemophagocytic lymphohistiocytosis (HLH) or macrophage activation syndrome (MAS)	HLH/MAS has been described in patients receiving adoptive cellular therapy and may overlap with CRS	• Study conducted with investigators at centers experienced with the administration of cellular therapies and management of HLH<br>• Protocol includes HLH management guidelines

Potential Risk of Clinical Significance	Summary of Data/Rationale for Risk	Mitigation Strategy
Neurotoxicity	• Neurotoxicity has been described with CAR-T-cell therapy and blinatumomab.<br>• Incidence of Grade ≥3 neurotoxicity in CD19 CAR-T studies has ranged from 31% (ZUMA-1 Study in adult DLBCL) to 18% (JULIET study in adult DLBCL)<br>• Symptoms include word-finding difficulties, delirium, encephalopathy, aphasia, seizure, hallucinations, tremor/myoclonus and in rare cases, diffuse cerebral edema<br>• Cerebral edema has been fatal in some studies of CAR-T (i.e., JC015 ROCKET trial)	• Study conducted with investigators at centers experienced with the administration of cellular therapies and management of neurotoxicity related to cellular therapy<br>• Inpatient hospitalization/observation for initial infusion of NeoTCR-P1 with first *participant* in each dose-escalation cohort staggered by 7 and 14 days in the Phase 1a and 1b, respectively<br>• Protocol includes neurotoxicity management guidelines (Modified CARTOX) that include anti-cytokine directed therapy including steroids and regular monitoring of mental status using a validated instrument (ICE modification of CARTOX10)<br>• *Consider earlier administration of steroids – at Grade 1-2 neurologic symptoms to prevent higher grades of neurotoxicity*
Tumor Lysis Syndrome (TLS)	Based upon an expert TLS consensus panel, the risk of tumor lysis syndrome is dependent on the disease type and burden of disease. This risk is deemed to be low for patients with non-bulky solid tumors that are not highly sensitive to chemotherapy (highly sensitive solid tumors include neuroblastoma, germ cell cancer, SCLC) (Cairo et al., 2010)	• Inpatient monitoring before and after conditioning chemotherapy and infusion of NeoTCR-P1 including blood tests for potassium and uric acid<br>• *Participants* with bulky tumors will receive hydration and allopurinol prophylactically per the discretion of the investigator<br>• TLS resulting in renal insufficiency, rapidly rising uric acid or evidence of organ dysfunction will be managed with IV fluids and rasburicase as needed and determined by the treating physician
Capillary Leak Syndrome (CLS)	• IL-2 administration has been associated with CLS<br>• Characterized by a loss of vascular tone and fluid accumulation in the extravascular space<br>• Hypotension and reduced organ perfusion may be severe and can be fatal	• *Participants* will *initially* receive subcutaneous low-dose IL-2, which is better tolerated than high-dose IL-2 administered intravenously (Ellebaek et al., 2012)<br>• Participants will be closely monitored for CLS, including generalized edema, weight gain, pulmonary congestion, pleural effusion, and ascites (Schwartz et al., 2002; Schwartzentruber,2001)<br>• IL-2 will be skipped or omitted per guidelines in Section 6.7.3

Potential Risk of Clinical Significance	Summary of Data/Rationale for Risk	Mitigation Strategy
Graft-versus-host disease/Autoimmunity	Potential for mixed TCR chains could also result in autoimmunity (see bullet points above)	• Eligibility criteria exclude patients with underlying autoimmune conditions<br>• *Participants* will be monitored at regular intervals both clinically and with labs to assess for autoimmunity. *Participants* with evidence of autoimmunity will be evaluated for expansion of certain T-cell subsets to determine if autoimmunity is related to NeoTCR-P1<br>• Protocol includes management guidelines for treating autoimmune toxicities
Lack of engraftment due to immune response directed against gene-engineered T cells or Cas9 Protein	• Although no antibody-mediated response is expected, a T-cell mediated response against an intracellular non-native proteins within neoTCR-P1 T cells is theoretically possible and carries some low risk (see bullet points above) of immunogenicity<br>• Cas9 is derived from a bacterial protein. Cas9 is transiently delivered as an RNP, rather than RNA or DNA. Evidence in the literature shows turnover of RNPs within approximately 3 days (Liang et al., 2015)	• NeoTCR-P1 cells in peripheral blood will be closely monitored
Uncontrolled T cell Proliferation	A leukemic-like process is theoretically possible. Nonetheless, autologous T cells retain their native signaling networks and therefore should retain their normal regulation of T-cell function	• CBCs will be closely monitored and neoTCR cells will be followed by flow cytometric analysis<br>• *Participants* would be given corticosteroids, chemotherapy, and possibly alemtuzumab (anti-CD52) to eradicate the genetically engineered T cells (Lamers et al., 2006) if excessive NeoTCR-P1 proliferation is noted
Clonality and insertional mutagenesis	• CRISPR/Cas9 introduces DNA breaks that resolve by mutagenic NHEJ resulting in insertions and deletions<br>• Risk that people who receive gene-edited cells may develop new tumors derived from their genetically modified cells. However, the risk is low (see bullet points above)	• *Participants* will be followed yearly for up to 15 years both clinically and with CBCs to assess for clonal outgrowth and secondary malignancies<br>• Off-target editing has been evaluated by multiple methodologies to identify potential “hot spots” during development and will be evaluated as part of the initial release process for *participants* in the Phase 1 study

Potential Risk of Clinical Significance	Summary of Data/Rationale for Risk	Mitigation Strategy
Manufacturing delays and failures	In studies of CAR-T-cell, manufacturing failures affected up to 10% of enrolled subjects in pivotal studies. Affected subjects died during delay or were dropped from the protocol	• Begin Screening Part 1 in earlier lines of treatment to reduce the observed manufacturing TAT<br>• Permit bridging therapy to contain disease while awaiting manufacture
**Study Procedures**		
Leukapheresis risks	Hypocalcemia, vasovagal response, venous access/need for catheter, blood loss, discomfort at venipuncture site, and infection	• *Participants* will be monitored during and after leukapheresis
Tumor biopsy risks	Bleeding, infection and depending upon location (e.g., lung) could include pneumothorax	• Biopsy by trained physicians<br>• IR guided biopsies should be obtained when needed

AESI = adverse event of special interest; ALL = acute lymphocytic leukemia; CBC = complete blood count; CLS = capillary leak syndrome; CPF = cell processing facility; CRS = cytokine release syndrome; DLBCL = diffuse large B-cell lymphoma; DMSO = dimethyl sulfoxide; ICF = informed consent form; ICU = intensive care unit; HLH = hemophagocytic lymphohistiocytosis; IV = intravenous; NHEJ = non-homologous end joining; MAS = macrophage activation syndrome; RNP = ; SCLC = small cell lung cancer; TLS = tumor lysis syndrome; TAT = turnaround time.

Refer to Toxicity Management Guideline (See Section 6.11) for additional details.

2.11.1. Potential for Overlapping Toxicities

Participants receiving NeoTCR-P1 plus IL-2 in the Phase 1a dose-expansion basket cohort may be at risk for additive or overlapping toxicities. IL-2 toxicity is mediated through lymphoid infiltration, capillary leak syndrome, and local effects of induced cytokines (Schwartz et al., 2002). Toxicities in multiple organ systems, including the heart, lungs, kidneys, and CNS may manifest similarly to symptoms of CRS or neurotoxicity potentially associated with NeoTCR-P1.

There is a risk for potential overlapping toxicities for participants in the Phase 1b of the Expansion Phase. Nivolumab is associated with infusion reactions, which in some patients have been severe and life threatening. Given the risk for febrile reactions and hypersensitivity reactions there is a potential that participants receiving the combination may be at increased risk. Checkpoint inhibitors have been associated with rare reports of both CRS and HLH. Given the potential for NeoTCR-P1 to induce CRS and HLH there may be some increased risk with the combination. Finally, nivolumab has been associated with severe acute and chronic GVHD in patients receiving nivolumab following allogeneic HSCT. Given the potential for mixed TCR chains in participants receiving NeoTCR-P1, there is a risk of increased toxicity in participants receiving the combination.

2.11.2. Benefit Assessment

Based upon inclusion criteria and published literature, eligible *participants* will have a median anticipated survival of <1–2 years and have no curative treatment options. The risks associated with alternative treatments generally are those associated with chemotherapy, targeted therapy, and other standard of care interventions

It is possible that NeoTCR-P1 T cells as single agent without or with IL-2 or the combination of NeoTCR-P1 plus nivolumab may exert rapid and durable anti-tumor effects as demonstrated by adoptive TIL therapy targeting neoantigens (Stevanović et al., 2017; Tran et al., 2016; Tran et al., 2014; Zacharakis et al., 2018) and gene-edited TCR-T cells targeting cancer testis and viral antigens (D'Angelo et al., 2018; Norberg et al., 2018).

2.11.3. Overall Benefit/Risk Conclusion

Considering the measures taken to minimize participant risk in this study, the potential risks identified in association with NeoTCR-P1, IL-2, or the combination of NeoTCR-P1 combined with nivolumab *without or with IL-2* are justified by the anticipated benefits that may be afforded to participants with advanced solid tumors.

3. Objectives and Endpoints

Objectives	Endpoints
**Primary**	
**Safety**	
• To evaluate the safety and tolerability of a single dose of NeoTCR-P1 administered as a single agent without or with IL-2 (Phase 1a), *and* in combination with nivolumab *without or with IL-2* (Phase 1b) in *participants* with locally advanced or metastatic tumors<br>• To determine the maximum tolerated dose (MTD) of NeoTCR-P1<br>• To identify a recommended Phase 2 dose (RP2D) of NeoTCR-P1	• Incidence and nature of DLTs<br>• Incidence, nature, and severity of adverse events graded according to NCI CTCAE v5.0 (ABSMT consensus grading criteria will be used for CRS and neurotoxicity)<br>• Incidence, nature and severity of adverse events of special interest (AESI) according to NCI CTCAE v5.0
**Feasibility**	
• To evaluate the feasibility of manufacturing NeoTCR-P1	• Percentage of screened *participants* in whom product selection is successful<br>• Percentage of screened *participants* that enroll on study<br>• Percentage of enrolled *participants* who receive NeoTCR-P1<br>• Percentage of manufactured products that meet release criteria for neoTCR-positive editing efficiency, T-cell viability, sterility, T-cell number<br>• Median time from leukapheresis to product delivery<br>• Median time from signing informed consent to infusion
**Secondary**	
**Pharmacokinetic/Pharmacodynamic**	
• To evaluate the pharmacokinetics of NeoTCR-P1 after administration as a single agent without or with IL-2 *and* in combination with nivolumab *without or with IL-2*	• Proliferation, engraftment, persistence, and trafficking of *neoTCR* T cells when administered as a single agent without or with IL-2 *and* in combination with nivolumab *without or with IL-2*<br>• Median values of C_{max} and $AUC_{0\text{–}28d}$ of neoTCR-*P1* cells administered as a single agent without or with IL-2 *and* in combination with nivolumab *without or with IL-2*<br>• Evaluation of neoTCR T-cell infiltration in biopsy specimens post-dosing
• To evaluate the pharmacodynamics of NeoTCR-P1 when administered as a single agent without or with IL-2 *and* in combination with nivolumab *without or with IL-2*	• Changes in blood cytokine levels following administration of NeoTCR-P1 as a single agent without or with IL-2 *and* in combination with nivolumab *without or with IL-2*
• To characterize the pharmacokinetics of fludarabine when administered as part of the conditioning chemotherapy regimen	• Fludarabine plasma and intracellular triphosphate concentrations

Objectives	Endpoints
**Immunogenicity (Phase 1b only)**	
• To evaluate the effect of NeoTCR-P1 administration on the immunogenicity of nivolumab	• Incidence of ADAs to nivolumab
**Activity**	
• Preliminary assessment of the anti-tumor activity of NeoTCR-P1 administered as a single agent, without or with IL-2 *and* in combination with nivolumab *without or with IL-2* in *participants* with locally advanced or metastatic solid tumors	• Objective response rate, defined as CR or PR per RECIST v1.1 as determined by the investigator<br>• Duration of response, defined as time from the first occurrence of a documented objective response to the time of relapse or death from any cause<br>• Progression-free survival (PFS), defined as the time from the first study treatment to the first occurrence of progression or death, whichever occurs first<br>• Overall survival (OS), defined as the time from the first study treatment to death
**Exploratory**	
• Preliminary assessment of biomarkers that might act as pharmacodynamic indicators of anti-tumor activity of NeoTCR-P1 when administered as a single agent without or with IL-2 *and* in combination with nivolumab *without or with IL-2*<br>• Preliminary assessment of biomarkers that might act as predictors of toxicity or anti-tumor activity of NeoTCR-P1 when administered as a single agent without or with IL-2 *and* in combination with nivolumab *without or with IL-2*<br>• Preliminary assessment of the relationship between fludarabine exposure and the pharmacokinetics and anti-tumor activity of NeoTCR-P1	• CD39 /CD103 as marker for homing to tumor<br>• Changes in T-cell functional markers and relative T-cell subsets (e.g., T_{scm}, T_{cm}, T_{em}, T_{eff}) of neoTCR-*P1* T cells when administered as a single agent without or with IL-2 *and* in combination with nivolumab *without or with IL-2*<br>• Incidence of adverse events and ORR as a function of maximum value of neoTCR T-cell expansion post-infusion, CD4/CD8 ratio of the product, baseline T-cell phenotypes, transfection ratio of the product, and tumor expression of the targeted neoepitope

ADA = anti-drug antibody; AESI = adverse event of special interest; ASTCT = American Society for Transplantation and Cellular Therapy; AUC_{0-28d} = area under the concentration-time curve from 0 to 28 days; C_{max} = maximum concentration; CR = complete response; CRS = cytokine release syndrome; DLT = dose-limiting toxicity; MTD = maximum tolerated dose; NCI CTACAE = National Cancer Institute Common Terminology of Adverse Events; IL-2 = interleukin 2; neoTCR = neoepitope-specific T-cell receptor; OS = overall survival; PFS = progression-free survival; PR = partial response; RECIST = Response Evaluation Criteria in Solid Tumors; RP2D = recommended Phase 2 dose; T_{cm} = T central memory; T_{eff} = T effector; T_{em} = T effector memory; T_{scm} = T stem cell memory.

4. Study Design

4.1. Overall Design

This is a first-in-human, single-arm, open-label, Phase 1a/1b study to determine the safety, feasibility, and efficacy of a single dose of NeoTCR-P1 T cells in participants with solid tumors. Study PACT-0101 will be separated into 2 distinct phases designated as the Initial Phase and the Expansion Phase as shown in Figure 9. Enrollment into the Expansion Phase will only commence following *SRT* review.

Figure 9 Phase 1a/Phase 1b Design

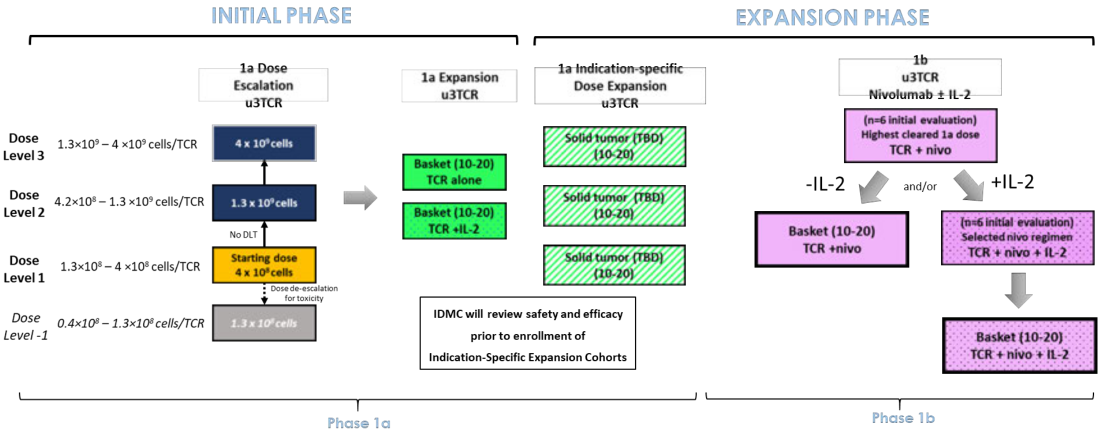

MTD/MAD = maximum tolerated dose/maximum administered dose; TBD = to be determined.
Dose based upon neoTCR-positive T cells (includes CD4 and CD8 cells).
Flat-fixed cell dose based on an average participant weight of approximately 80 kg. The total gene-edited cell dose at each dose level is independent of the number of neoTCRs in the product. The number of neoTCR+ T cells/TCR at each dose level is approximately inversely proportional to the number of neoTCRs in the product.
Dose escalation may continue in half-log increments beyond upper dose level if enabled by manufacturing.
Basket and expansion cohorts may be enrolled at any dose at or below the MTD/MAD.
Solid tumor (TBD) expansion cohort to be determined by Sponsor based upon data from basket or data external to the study.

The **Initial Phase** consists of a dose-escalation portion and 2 dose-expansion basket cohorts. The dose-escalation portion will evaluate the safety and feasibility of cell products containing gene-edited TCRs with up to 3 distinct clonal populations (u3TCR). If acceptable safety and anti-tumor activity is observed in the Initial Phase of the study, then enrollment into the Expansion Phase will proceed following review by *the SRT* (see Section 4.1.4 for further details regarding SRT).

The **Expansion Phase** of the study will comprise 2 portions. The Phase 1a portion will include indication-specific dose-expansion cohorts to provide a preliminary estimation of single-agent activity in 3 different solid tumor indications. The Phase 1b portion is designed to evaluate the safety, tolerability, and efficacy of NeoTCR-P1 when administered in combination with nivolumab *without or with IL-2. Testing of the combination with nivolumab will commence at the highest cell dose cleared during the Initial Phase with the potential for evaluation of staggered nivolumab dosing if the concomitant dosing on Day 1 is not tolerated. If higher dose levels of NeoTCR-P1 are subsequently cleared in the Phase 1a portion of the study, the NeoTCR-P1 dose in the Phase 1b portion of the trial may be increased accordingly.*

After a tolerable nivolumab combination regimen is established, the addition of IL-2 to this regimen may be evaluated. Up to a maximum of 20 participants may be enrolled under each of the Phase 1b regimens (nivolumab and nivolumab + IL-2 combinations).

If multiple cohorts are open concurrently, participants will be allocated according to several criteria, including the number of TCRs in the product (participants with 3 TCRs being preferentially enrolled in dose escalation cohorts), tumor type (if indication-specific expansion cohorts are open), and their eligibility for combination treatment (if Phase 1b cohorts are open). In general, except for patients in active dose escalation cohorts, participants will be targeted to receive the highest cell dose previously cleared during dose escalation.

Phase 1a (Initial) Phase

During dose escalation, the total number of gene-edited cells administered to participants for a 1TCR, 2TCR, or 3TCR product will be the same at a given dose level. The number of neoTCR+ T cells/TCR at each dose level is approximately inversely proportional to the number of neoTCRs in the product.

Only participants who received a 3TCR product will be counted towards clearing a given dose level.

After preliminary safety of NeoTCR-P1 has been established at a given dose level, additional participants may be enrolled into 2 separate dose-expansion basket cohorts (up to 20 participants each) within the Initial Phase to receive product containing up to 3 neoTCRs (u3TCR) without or with low dose IL-2. Enrollment into the dose-expansion basket cohorts may commence after the dose-limiting toxicity (DLT) window has been cleared and at least 6 participants dosed with 1, 2, or 3TCR products have been evaluated at or above a given dose level during dose-escalation. As successive doses are cleared during dose escalation, additional dose levels may be included in the basket cohorts.

In the Phase 1a Initial Phase of the study, potentially up to 76 participants with melanoma, urothelial carcinoma, NSCLC, HNSCC, CRC, ovarian cancer, HER2-negative breast cancer (HR+ or triple negative), or prostate cancer will be enrolled (see Section 4.2.1 for Rationale for Patient Population). The primary objectives of this phase are to evaluate the safety and tolerability, determine the MTD, and evaluate manufacturing feasibility. The Phase 1a Initial Phase design is presented in Figure 10.

Figure 10 Phase 1a Initial Phase

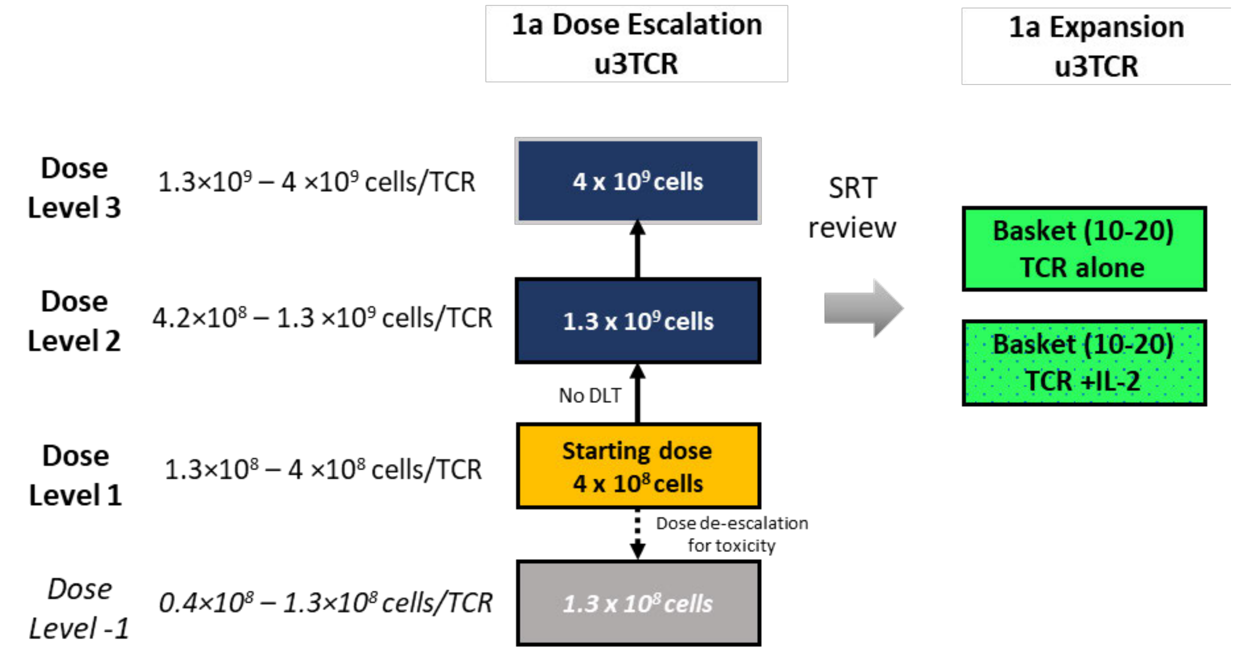

neoTCR = personalized adoptive T-cell receptor; SRT = safety review team.
Dose based upon neoTCR-positive T cells (includes CD4 and CD8 cells). The total gene-edited cell dose at a given dose level is the same independent of the number of neoTCRs administered.

Approximately 3 dose levels (Table 13) ranging from 4×10^8 (Dose Level 1) to 4×10^9 (Dose Level 3) neoTCR-positive T cells may be evaluated to determine the safety of NeoTCR-P1 (see Section 4.1.5 for DLT rules and further details regarding enrollment of dose-escalation cohort).

The starting dose will be administered by intravenous (IV) infusion on Day 0 (see Section 4.2.11 for justification of starting dose of NeoTCR-P1).

If DLTs are observed at Dose Level 1 that prevent escalation to the next level, lower dose levels decreasing in half-log increments from the starting dose may be evaluated.

In the absence of DLTs that prevent escalation to the next dose level and depending on new nonclinical efficacy, clinical safety, feasibility, manufacturing, and clinical pharmacokinetic (PK) data, additional dose levels beyond Dose Level 3 increasing in half-log increments may be evaluated to determine the MTD or MAD.

A 3 + 3 dose escalation (or de-escalation in the case of DLTs at the initial starting dose) will be employed for all cohorts. A minimum of 3 participants will be enrolled at each dose level. If the first 3 participants enrolled in a dose-escalation cohort complete dosing through the DLT assessment window without experiencing DLTs, participants may be enrolled at the next higher dose level.

Prior to the determination of the MTD or MAD, additional participants may be enrolled at each dose level (to a maximum of 12) to further characterize the safety at a given dose, interrogate a potential biomarker signal (e.g., PD-L1 immunohistochemistry [IHC]) and/or help to facilitate the logistics related to enrollment of participants with a personalized product.

Phase 1a Expansion (u3TCR)

A safety review team (SRT), internal to the study Sponsor, in combination with study investigators will review the safety data at each dose level and make recommendations on further study conduct of the Phase 1a portion (see Section 4.1.4 regarding structure of the SRT). Once a tolerable dose is established within the Phase 1a dose escalation cohorts and the safety data is reviewed by the SRT, up to 20 participants may be enrolled in 2 dose-expansion basket cohorts within the Phase 1a portion in order to further characterize the safety of NeoTCR-P1 as a single agent without and with IL-2 *to* more thoroughly understand relationships between dose and safety or dose and efficacy, and to assess biomarkers of activity in different cancer types.

Enrollment into the dose-expansion basket cohorts may occur in parallel with ongoing dose escalation after the DLT window has been cleared and at least 6 participants have been evaluated at or above a given dose level (e.g., if early anti-tumor activity is observed, an MTD is not reached at Dose Level 2 and manufacturing feasibility permits exploration of Dose Level 3, then participants may be enrolled in the basket cohorts at Dose Levels ≤2).

Table 13 Cell Dose as a Function of the Number of Administered NeoTCRs

		NeoTCR+ T Cells per/TCR Clonal Product Population		
**Dose Level (DL)**	**Total Dose of NeoTCR+ T cells (±20%)**	**1TCR**	**2TCRs**	**3TCR**
**DL 3**	4.0×10^9	4.0×10^9	2.0×10^9	1.3×10^9
**DL 2**	1.3×10^9	1.3×10^9	6.3×10^8	4.2×10^8
**DL 1**	4.0×10^8	4.0×10^8	2.0×10^8	1.3×10^8

Note: different manufacturing and cell expansion efficiencies for each TCR may result in non-idealized ratios (i.e., not 1:1 or 1:1:1 for 2TCR and 3TCR products, respectively).

Enrollment in the IL-2 dose-expansion basket cohort will initially be limited to 6 participants *who will receive low-dose IL-2 (500,000 IU/m² subcutaneous twice daily for a maximum of 14 doses)*. After the 6th participant has completed their IL-2 regimen, the SRT in consultation with study investigators will review aggregate safety data and decide whether to continue enrollment (up to a maximum of 20 participants). *If data from this study suggests that the low-dose IL-2 regimen is not sufficient to support in vivo expansion and persistence of neoTCR T cells and/or emerging data external to this study suggest that a conventional high-dose (HD) regimen is superior in supporting the persistence and expansion of adoptively transferred T cells, the SRT may recommend evaluating a more intense regimen, not to exceed 600,000 IU/kg IV every 8 hours for a maximum of 15 doses (e.g., Jazaeri et al., 2019, Sarnaik et al., 2018). The SRT recommendation will be formally communicated to site investigators. If a high dose regimen is adopted, a review of consensus management practices will be conducted with all investigators before the next participant in the cohort*

is dosed. An alternative regimen will initially be evaluated in 6 participants, at which time the SRT in consultation with study investigators will decide whether to continue enrollment, and with which of the tested regimens, to a maximum of 20 participants.

The SRT will review data after 10 and 20 participants in the basket cohorts have *had the opportunity to be followed to the first* response assessment (see Figure 11). The *SRT* will make recommendations as to whether to open up to 3 indication-specific expansion cohorts in the Expansion Phase (i.e., beyond the basket-expansion cohorts in the Initial Phase) based upon aggregate feasibility, safety, and efficacy data.

Figure 11 Schema for Opening Dose Expansion Cohorts

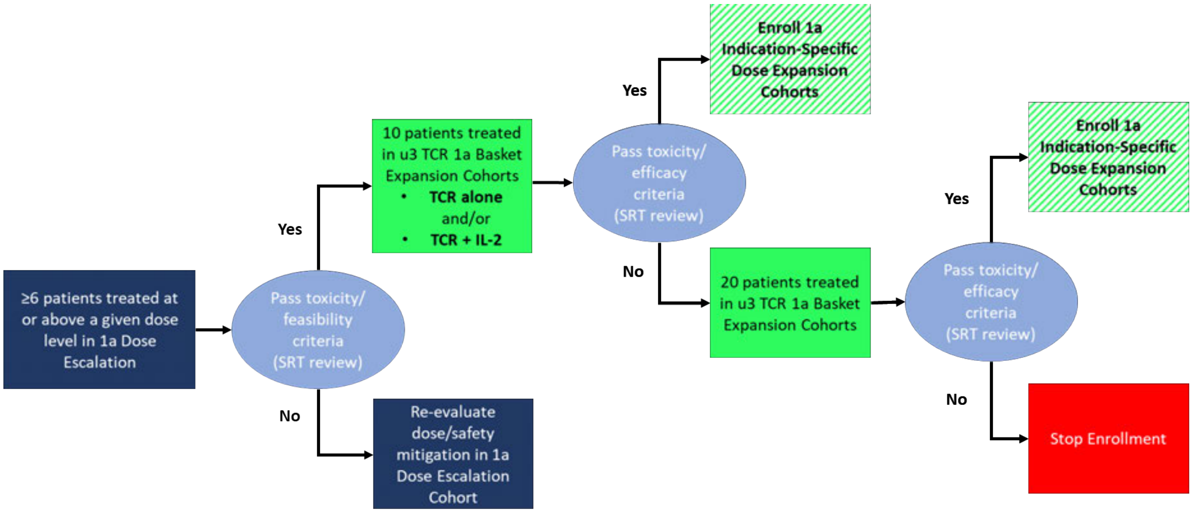

Minimum number of patients treated	6	16	26

SRT = safety review team.
Note: Opening of the indication-specific expansion cohorts will be based primarily on acceptable feasibility, safety and efficacy data from participants treated with NeoTCR-P1 alone in the 1a dose-escalation and dose-expansion basket cohorts (minimum 16).

Expansion Phase. In the Expansion Phase of the study, *up to 112* participants with metastatic melanoma, urothelial carcinoma, NSCLC, HNSCC, CRC, ovarian cancer, HER2-negative breast cancer, or prostate cancer may be enrolled.

Phase 1a Indication-specific Expansion Cohorts. *Participants in the dose-expansion cohorts will be enrolled at or below the MTD or MAD that has been cleared in the initial Phase 1a dose-escalation portion of the study.* The 3 indication-specific expansion cohorts will enroll participants with tumor types that will be selected by the Sponsor based upon potential efficacy signals observed in the initial *dose-escalation portion of the study, the participants treated in the two* basket *cohorts and/*or other supportive data external to the study. The purpose of the indication-specific expansion cohort is to more accurately estimate the efficacy in specific tumor types and to determine the breadth of activity across different tumor types with different mutational burdens and immune contextures. If cumulative data suggests

that IL-2 is a necessary component of the regimen, NeoTCR-P1 (u3TCR) may be administered with IL-2 in the indication-specific expansion cohorts.

Phase 1b portion. *Enrollment into the Phase 1b portion may begin after preliminary safety and optimal dosing of single agent NeoTCR-P1 is reviewed by the SRT after clearance of at least one dose level in Phase 1a Dose Escalation and at least 12 participants in the Initial Phase have had the opportunity to be followed until their first tumor assessment.*

Phase 1b participants will receive the NeoTCR-P1 at the highest dose level cleared during the Initial Phase. Higher doses cannot be administered until the corresponding dose level has been cleared in the Phase 1a portion of the study. The gates for opening Phase 1b cohorts are presented in Table 14.

NeoTCR-P1 + Nivolumab

The Phase 1b portion of the study will begin with the evaluation of a single infusion of NeoTCR-P1 (u3TCR) in combination with nivolumab administered following NeoTCR-P1 on Day *1 and then every 4 weeks for up to 7 cycles.*

Enrollment will initially be limited to 6 participants. After the 6th participant treated with NeoTCR-P1 plus nivolumab has been followed for at least 28 days, the SRT in consultation with study investigators will review aggregate safety data and decide whether to continue enrollment (up to a maximum of 20 participants). If any of the first 6 participants experiences a DLT, the SRT may recommend exploring an alternative nivolumab schedule.

If the combination with nivolumab on Day *1* is not well tolerated, then an alternative schedule with nivolumab *may* be tested (3 + 3*). The SRT may recommend administering the first dose of nivolumab on Day -1,* Day 14, *or* Day 29. *In addition, lower doses* of nivolumab *may* be *tested*. In the case of staggered dosing, the DLT window will begin after the first infusion of nivolumab. *Each schedule will be evaluated initially in up to 6 participants.*

Following SRT review, up to a maximum of *20* participants *may* be enrolled *under the chosen NeoTCR-P1 + nivolumab regimen.*

NeoTCR-P1 + IL-2 and Nivolumab

If warranted by data from the Initial Phase and/or supportive data external to the study, the Sponsor may optionally test the combination of NeoTCR-P1 with IL-2 plus nivolumab in the Phase 1b portion of the study. Testing of this combination may begin after a safe and tolerable NeoTCR-P1 + nivolumab regimen has been established as described above, as well as SRT review of safety data from 6 participants treated in the NeoTCR-P1 + IL-2 basket expansion cohort in the Initial Phase. The recommended IL-2 regimen will not exceed that tested by Chatzkel et al. (2019) in their trial of coordinated pembrolizumab and high-dose IL-2 in patients with RCC. Enrollment will initially be limited to 6 participants, and following SRT review up to a maximum of 20 participants may be enrolled to receive NeoTCR-P1 with IL-2 plus nivolumab. If any of the first 6 participants experiences a DLT, enrollment in the cohort will be halted.

Table 14 Gates for Opening Phase 1b Cohorts (Expansion Phase)

Cohort	Gate (SRT review)
TCR + nivo	• *Clearance of at least one dose level in Initial Phase dose escalation*<br>*AND*<br>• *≥12 participants treated in Initial Phase*<br>***Full enrollment (up to 20) following SRT review of first 6 treated with TCR + nivo***
TCR + IL-2 + nivo	• *≥6 participants treated with TCR + nivo regimen*<br>*AND*<br>• *≥6 participants treated in TCR + IL-2 basket expansion cohort with selected regimen in Initial Phase*<br>***Full enrollment (up to 20) following SRT review of first 6 treated with TCR + IL-2 + nivo***

IL-2 = interleukin 2; nivo = nivolumab; SRT = safety review team; TCR = T-cell receptor.

Study Phases and Procedural Requirements

Independent of the portion of the study, each participant will follow the same study treatment schedule and procedural requirements as depicted in Figure 12. Each participant will proceed through the following study phases:

- Screening Part 1*: assessment of eligibility Part 1*
- Identification of neoepitope-specific TCR
- Screening Part 2*: assessment of eligibility Part 2*
- Enrollment/Leukapheresis
- *Bridging Therapy, if applicable*
- *Baseline visit:* re-evaluation *prior to conditioning chemotherapy*
- Conditioning chemotherapy
- Investigational product infusion
- Primary safety and efficacy follow-up
- Long-term safety follow-up (15 years following administration of neoTCR)

For study requirements assigned to each study period, please refer to the Schedule of Assessments (SOA) and Section 1.4 for details.

During Screening Part 1, participants will *submit tumor specimens and whole blood for PBMC isolation after Part 1 performance status, medical and cancer history, and end organ function eligibility criteria are met. Participants will then* be assessed for the presence of neoepitope-specific T cells.

In Screening Part 2, *if TCRs are identified,* participants will undergo staging and other screening procedures *prior to being approved for leukapheresis.*

Note: Participants who enter this study with TCRs identified under a separate screening protocol are not required to undergo Screening Part 1 assessments and will enter this study at Screening Part 2.

Participants who meet *Screening Part 2* eligibility criteria will undergo leukaphereses to obtain large numbers of PBMCs for NeoTCR-P1 manufacturing (see Section 8.5.3). The T cells will be purified from the PBMCs, transfected with DNA plasmids, expanded ex vivo and then cryopreserved for future administration after passing all release tests.

Prior to starting conditioning chemotherapy, participants will be re-evaluated for select eligibility criteria (see Table 2, Section 1.4 for the procedures to be performed and the applicable windows with respect to the start of chemotherapy). Unless contraindicated (i.e., due to a change in participant status or laboratory values since meeting eligibility criteria in Screening Part 2), participants will be given conditioning chemotherapy before NeoTCR-P1 infusion. The chemotherapy will be planned so that it is completed 2 days BEFORE the planned infusion of the NeoTCR-P1 product.

Participants will be hospitalized for a minimum of 7 days after their infusion of NeoTCR-P1 and must meet pre-specified criteria prior to discharge (see Section 8.5.7). Participants will be assessed for clinical response according to Response Evaluation Criteria in Solid Tumors (RECIST v1.1) (as described in Appendix 9.6). All participants will have blood tests conducted to assess safety, engraftment, expansion, and persistence of NeoTCR-P1 T cells at regular intervals as outlined in the SOA. Trafficking of NeoTCR-P1 cells to the tumor will be assessed in on-treatment tumor biopsies.

Following the initial infusion period, participants will return to the clinic weekly until Week 4, monthly until Month 6, and then every 3 months until Month 24. Following this evaluation, participants will enter a long-term follow up (LTFU) study for annual follow up.

Participants who discontinue from Post-Treatment Follow-Up before Month 24 (e.g., for unequivocal disease progression; see Section 7.1) will enter the Long-Term Follow-Up period at that time.

A flowchart of the study design is presented in Figure 12.

Figure 12 Study Design

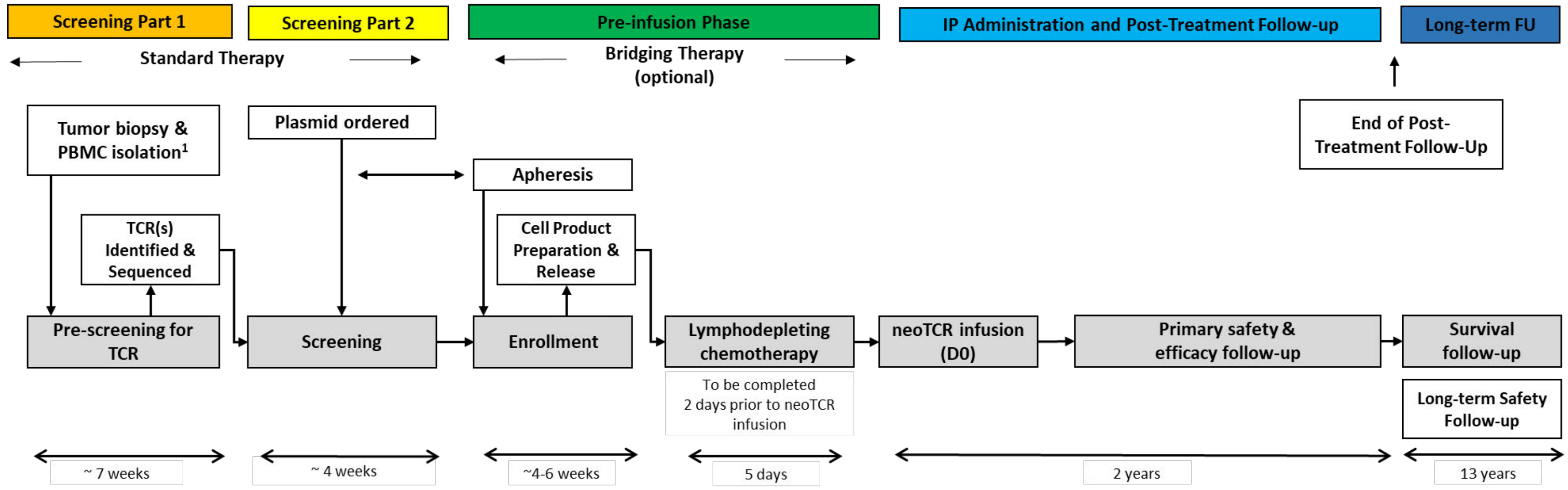

FU = follow-up; neoTCR = personalized adoptive T-cell receptor; PBMC = peripheral blood mononuclear cell; TCR = T-cell receptor.
In Screening Part 1 tumor and blood for isolation of PBMCs will be collected in order to identify neoepitope-specific T cells recognizing tumor neoantigens. This can be performed in participants in either the locally advanced or metastatic setting while participants are receiving other therapies.
Progression/relapse event triggers the start of Screening Part 2. Following discussion between the Investigator and Sponsor, participants may undergo leukapheresis and product manufacture during earlier lines of therapy in order to decrease the time from disease progression/relapse to neoTCR infusion. In this case enrollment will *occur* once all eligibility criteria in Screening Part 2 are met.
Bridging therapy is permitted during the Pre-Infusion Phase.
Long-term safety follow-up may be conducted under a separate rollover protocol.

4.1.1. Participating Sites

The study will be initiated at approximately *12* sites in the United States experienced in the administration of cellular therapy (e.g., CAR-T, adoptive transfer of T cells). During the conduct of the study, the number of sites will be gradually be expanded to include additional experienced sites in the United States and potentially outside of the United States.

4.1.2. Number of Participants

It is anticipated that potentially *9–188* participants will be enrolled into this study as detailed below. The sample size for this study will be determined by the dose-escalation rules as well as by the number and size of expansion cohorts, which will be overseen by *the SRT*.

The sample size estimates are as follows:

- Initial Phase: potentially 9–76 participants
- Expansion Phase: potentially 0–*112* participants

The minimum and maximum numbers of participants for each phase of the study are listed in Table 15.

Table 15 Minimum and Maximum Numbers of Participants

**INITIAL PHASE**	**Minimum Number of Participants**	**Maximum Number of Participants**
Phase 1a Dose Escalation		
u3TCR	9	36
Phase 1a Dose-Expansion Baskets		
u3TCR	-	20
u3TCR + IL-2	-	20
**Initial Total**	**9**	**76**
**EXPANSION PHASE**		
Phase 1a Indication-Specific Dose Expansions		60
Solid Tumor 1 (TBD)	-	20
Solid Tumor 2 (TBD)	-	20
Solid Tumor 3 (TBD)	-	20
**Phase 1a Total**		**136**
Phase 1b	-	
u3TCR + nivolumab		*32*
u3TCR + nivolumab + IL-2		*20*
**Phase 1b Total**		***52***
**Expansion Total**		***112***
**OVERALL TOTAL (Initial + Expansion/Phase 1a + 1b)**	**9**	***188***

TBD = to be determined; u3TCR = up to 3TCR product.

4.1.3. Replacement of Participants

DLT evaluable participants are those who have received the protocol-specified dose of study treatment.

Participants with a manufactured cell dose that is less than the protocol-specified dose for their cohort will receive their cell infusion, provided that the manufactured dose is above the cell processing facility (CPF) minimum acceptable dose for infusion (1.6×10^7 neoTCR+ T cells per TCR) and all other manufacturing release criteria are met. If they experience a DLT during the 28-day post-infusion window, it will be counted in the assessment of DLTs for that cohort. However, if they do not experience a DLT they will not contribute towards clearing a dose-escalation level and will be considered DLT non-evaluable. Participants that are infused with a dose below Dose Level 1 will be excluded from the efficacy evaluable population as part of the secondary endpoint analysis but will be included in the Full Analysis population (see Section 8.14) regarding Population for Analyses).

For the purposes of clearing dose-escalation levels, DLT non-evaluable participants will be replaced. DLT evaluable and non-evaluable participants will be followed in the same manner according to the Schedule of Assessments (see Section 1.4).

4.1.4. Safety Review Team

An SRT will be specifically chartered to review safety data during Phase 1 of the study and make recommendations on further study conduct in Phase 1. The SRT will comprise 2 physicians and 1 statistician. The *recommendation* to *proceed to the expansion phase (i.e.,* indication-specific cohorts *and Phase 1b)* will be made by the *SRT* and will be based upon the incidence of NeoTCR-P1 DLTs as well as a review of serious adverse events (SAEs) and efficacy. The *SRT will review safety data at 3 timepoints as described in Figure 11.*

4.1.5. Definition of Dose-Limiting Toxicity and DLT Rules

Adverse events will be graded according to NCI CTCAE v5.0. The American Society for Transplantation and Cellular Therapy (ASTCT) consensus grading criteria will be used for CRS and neurotoxicity.

For this Phase 1a/1b study, a DLT is defined as any 1 of the following events occurring during the DLT-assessment window and is considered to be possibly, probably, or definitely related to NeoTCR-P1 (Phase 1a) or the combination of NeoTCR-P1 and nivolumab (Phase 1b):

- Any Grade 3 or higher toxicity: non-hematologic, non-hepatic adverse event
- DLT exceptions include the following:
  - Immediate hypersensitivity reactions occurring within 2 hours of cell infusion (related to cell infusion) that are reversible to a Grade 2 or less within 24 hours of cell administration with standard therapy
  - Grade 3 acute infusion reactions lasting <72 hours
  - Grade 3 CRS (ASTCT criteria) lasting <72 hours

  - Grade 3 neurotoxicity by the ASTCT immune-effector cells associated neurotoxicity syndrome (ICANS) criteria lasting <72 hours that resolves to Grade 1 or less within 2 weeks
  - Grade 3 nausea, vomiting, or diarrhea that resolves to Grade ≤2 with treatment in ≤3 days and does not require total parenteral nutrition or hospitalization
  - Grade 3 fatigue that resolves to Grade ≤2 in ≤7 days
  - Grade 3 laboratory abnormalities that are asymptomatic and considered by the investigator not to be clinically significant
  - Grade 3 rash that resolves to Grade ≤2 in ≤7 days with topical treatment or therapy equivalent to prednisone 10 mg/day or less
  - Grade 3 arthralgia that can be adequately managed with supportive care or that resolves to Grade ≤2 in ≤7 days
  - Grade 3 autoimmune thyroiditis or other endocrine abnormality that can be managed by endocrine therapy that would not necessitate initiation of systemic corticosteroids (with the exception of replacement steroid for adrenal insufficiency)
- Grade 4 neutrophil count decrease (afebrile) lasting longer than 7 days from the day of cell transfer
- Grade 4 thrombocytopenia lasting longer than 7 days from cell transfer or Grade 3 thrombocytopenia associated with clinically significant bleeding
- Grade ≥3 febrile neutropenia
- Grade 4 anemia lasting longer than 7 days from the day of cell transfer
- Grade ≥3 elevation of serum hepatic transaminase (ALT or AST) lasting >7 days
- DLT exceptions include the following:
  - Grade 3 ALT or AST elevation lasting <3 days in the context of CRS
- Grade ≥3 elevation of serum total bilirubin
- ALT or AST >3X upper limit of normal (ULN) in combination with total bilirubin >2 × ULN or clinical jaundice in the absence of cholestasis or other causes of hyperbilirubinemia

Dose escalation will occur in accordance with the following rules:

- Each of the 3+3 cohorts will initially enroll a minimum of 3 participants. Dosing of the first and subsequent participant will be staggered by ≥7 days in the Phase 1a and ≥14 days in the Phase 1b. In the absence of a DLT, dose escalation may proceed. If a DLT occurs in a cohort, that cohort will be expanded to a minimum of 6 participants.
- If fewer than one-third of DLT evaluable participants in a given cohort experiences a DLT (i.e., DLT in fewer than 1 of 3 or 2 of 6 participants), escalation may proceed to the next higher dose level.
- If a DLT is observed in 2 of 6 participants, the SRT may recommend enrolling 2 additional sets of 3 participants (up to 12 total) at the same dose level. In this scenario, progression to an additional cohort or to the dose-expansion basket cohort will proceed if

≤2 of the first 9 or if ≤3 of the 12 participants present with a DLT. If the DLT rate is >2/6, >3/9, or ≥4/12 participants, the MTD will have been exceeded.

- If the MTD is exceeded at a dose level, the previous highest dose level at which fewer than one-third of at least 6 participants experiences a DLT will be declared the MTD; additional participants may be enrolled at the preceding dose level to achieve a total of at least 6 evaluable.
- If fewer than 2 of 6, 3 of 9, or 4 of 12 evaluable participants at the highest dose level experiences a DLT, then this dose level will be declared the MAD.

Participants with 1, 2, or 3 neoTCRs will be enrolled in the dose-escalation cohorts. All participants will be evaluated for DLTs; however, only participants who received a product containing 3 neoTCRs will contribute to clearing a given dose level.

Figure 13 Phase 1 DLT Evaluation Schema (Alternative)

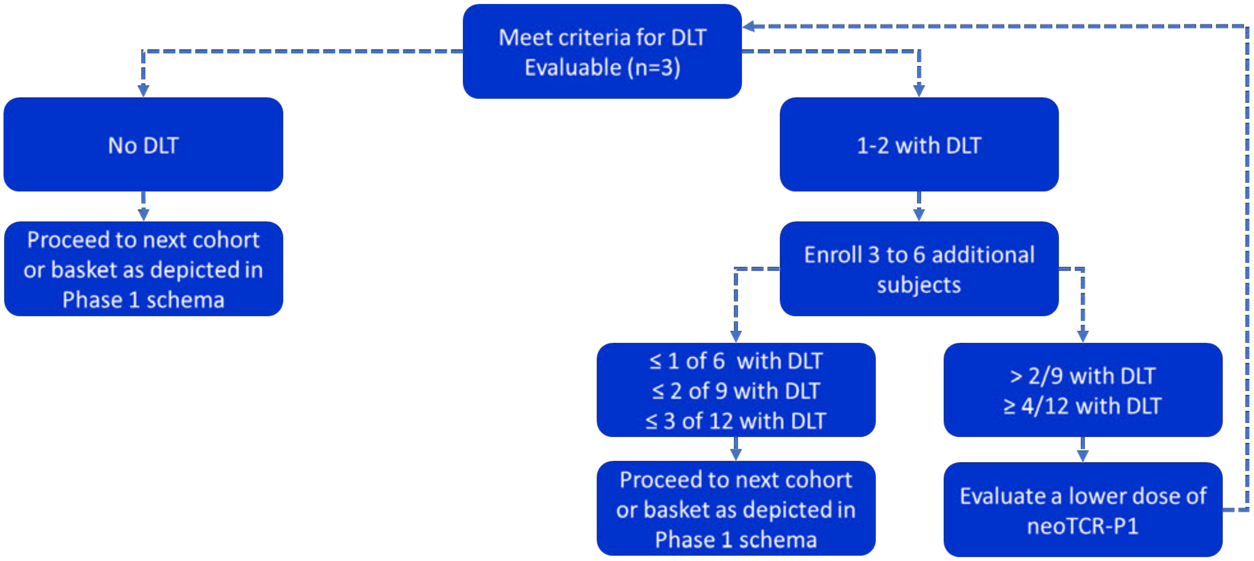

DLT = dose-limiting toxicity; neoTCR = personalized adoptive T-cell receptor.

The same DLT rules above will apply in the Phase 1a and Phase 1b *cohorts. When a regimen is initially evaluated in 6 participants (i.e., TCR + IL-2 Basket Expansion, Phase 1b nivolumab and nivolumab + IL-2 cohorts) accrual will be stopped if a DLT occurs. In that case, following SRT review in consultation with site investigators, enrollment may be resumed with appropriate modifications to the regimen (e.g., staggered nivolumab dosing as described).*

Participants in each cohort will be evaluated for DLTs within the first 28 days following the completion of their respective infusion. The analysis of DLTs will be based on the DLT-evaluable set of participants. The SRT will make recommendations based on the incidence of DLTs and overall safety profile of the regimen.

Adverse events that meet the definition of a DLT should be recorded on the Adverse Event electronic case report form (eCRF). In addition, a written DLT assessment worksheet should be completed and submitted immediately to the Medical Monitor. Investigators will also participate in frequent teleconferences with the Sponsor; during which, they will report any DLTs observed during the DLT assessment window for each participant in the dose-escalation stage of the study.

4.2. Scientific Rationale for Study Design

Encouraging clinical data emerging in the field of tumor immunotherapy have demonstrated that therapies focused on enhancing T-cell response against cancer can result in a significant survival benefit in patients with advanced metastatic cancer (Hodi et al., 2010; Kantoff et al., 2010; Chen et al., 2012).

4.2.1. Rationale for Patient Population

The indications for the Phase 1a and Phase 1b dose escalation and basket portions of the study were selected based upon the unmet need, biology, ability to harmonize manufacturing with standard of care therapies, likelihood of obtaining surgical specimen, ability to identify neoTCRs in nonclinical studies, and the potential to study tumor indications across the mutational spectrum. Specifically, the following tumor types were selected: 4 tumor types with high mutational load (melanoma, bladder, NSCLC, and HNSCC), 2 tumor types with intermediate mutational load (CRC and ovarian cancer), and 2 tumor types with low mutational load (prostate and breast cancer). These tumor types were selected in order to more thoroughly understand the potential breadth of activity across different immune contextures (see Figure 14 for additional details about the mutational landscape).

Figure 14 Tumor Mutational Load Across Landscape of Cancer Indications

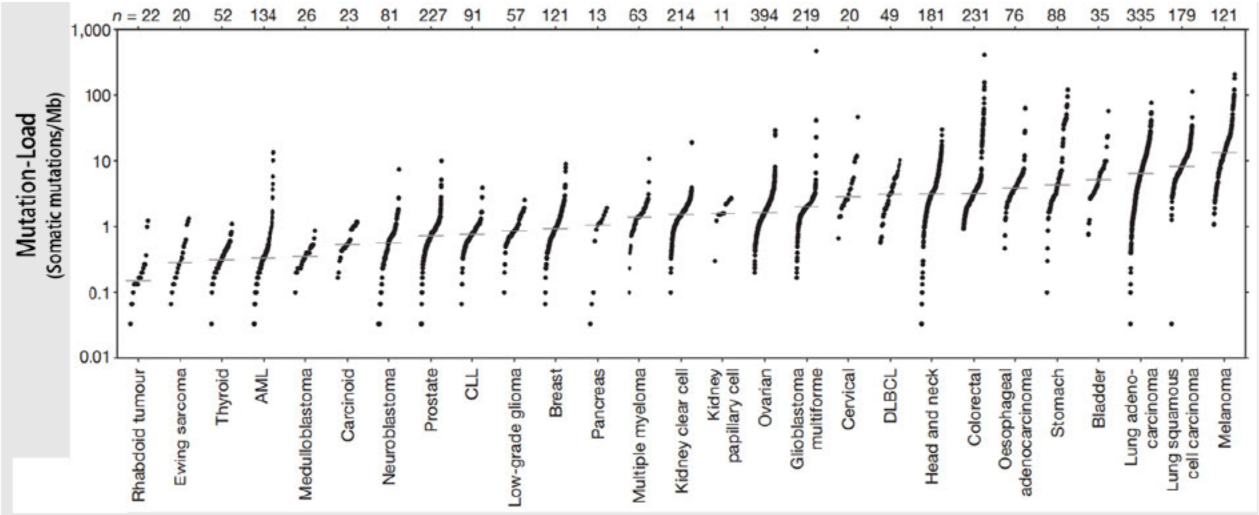

AML = acute myelogenous leukemia; CLL = chronic lymphocytic leukemia; DLBCL = diffuse large B-cell lymphoma.

Source: Lawrence et al., 2013.

Participants in this study are required to have tumors that have progressed on at least 1 first-line regimen and have no additional curative options. Given the turnaround time for manufacturing and concerns about heavily pretreated participants having a significantly lower percentage of CD3+ T cells in leukapheresis products than less heavily pretreated participants (thus limiting the ability to manufacture product), participants are not required to exhaust all approved therapies prior to enrollment (Schuster et al., 2019). The patient population that has been selected for this study is expected to have a median OS of approximately 1–2 years with available salvage therapies.

In summary, novel therapies for relapsed solid tumors are urgently needed. Targeting tumors with engineered TCRs has already demonstrated benefits in patients with very advanced solid tumors and has the potential to induce durable responses in patients otherwise beyond treatment.

4.2.2. Rationale for Study Design and Endpoints

A single-arm open-label study design is supported by the first-in-human nature of the study.

The 2-stage structure that includes an Initial and Expansion Phase was utilized to balance the first-in-human nature of this study with the potential to expedite development by seamlessly proceeding to larger cohorts that can estimate anti-tumor activity in a given indication. The use of an SRT to make decisions about the expansion of the study safeguards participants while allowing for an efficient transition to larger populations. In addition, in order to mitigate challenges in disseminating new safety information to investigators, IRBs, and regulators in a timely manner, the Investigator Brochure will be updated twice yearly following the opening of the Phase 1a expansion cohort or more frequently, if new safety signals arise.

Safety, as assessed by the incidence of DLTs as well as the incidence and severity of adverse events, is a relevant endpoint for this first-in-human trial. In addition, given the individualized manufacturing of this cell therapy product, feasibility of manufacturing is also a relevant endpoint to assess. In addition, safety will be monitored for up to 15 years per health authority guidelines (FDA, European Medicines Agency [EMA]) for studies of genome-editing technologies.

4.2.3. Rationale for Evaluating Polyclonal TCR Products

The up-to-3TCR product (abbreviated u3TCR) will include as many as 3 populations of neoTCR-targeted T cells (i.e., 3 distinct clonal populations of neoepitope-specific T cells). These T cells may target the same or different neoantigens in the context of the same or different HLA restrictions. While a single neoTCR product is anticipated to deliver meaningful clinical benefit, a polyclonal TCR T-cell product holds the potential to improve efficacy through target diversification. Targeting more than 1 tumor specific neoantigen and/or diversification of target HLA molecules presenting the neoepitopes is designed to address the potential for tumor immune evasion mediated by downregulation of target antigen presentation and/or HLA expression (McGranahan et al., 2017; Paulson et al., 2018; Tran et al., 2016; Verdegaal et al., 2016).

The hypothesis under test is that a polyclonal product is likely to confer improved clinical benefit as well as longer persistence of clinical benefit than one consisting of a single gene-engineered

T cell. However, it will in general not be possible to predictively characterize the contribution of each clone to any observed anti-tumor activity. Because each product is exquisitely personalized, a controlled evaluation of the effect of additional TCRs is not feasible. Therefore, a product containing up to 3 neoTCRs will be manufactured whenever possible to maximize the potential therapeutic benefit for every individual patient.

Results from other ACT studies suggest that the clonality of T cells in the infused products is not, ipso facto, a safety concern. TIL infusion products are highly individualized and contain numerous different (and largely uncharacterized) T cells with varying specificities and functionalities, but collectively they exhibit a relatively consistent tolerable toxicity profile over a wide range of total cell doses. A recent study by Hont et al. (2019) provides a granular illustration of this general experience. The authors treated 18 patients with mixtures of autologous ex vivo expanded multiantigen-associated cytotoxic T cells. T cells recognizing 3 tumor associated antigens (TAA; PRAME, survivin, and WT1) were isolated from the peripheral blood of patients with tumor types known to express high levels of these targets. T cells in the products from individual patients varied with respect to phenotype (CD8+ vs CD4+), specificity for the targeted antigens (with some products demonstrating specificity to only 1 of the 3), and functionality (as evaluated by *enzyme-linked immunosorbent spot* [ELISPOT]). The authors evaluated 3 dose levels (1, 2, and 4 × 107 cells/m^2; patients were allowed to receive multiple infusions) and reported that there were no DLTs or infusion-related adverse events. They did not report specific adverse events attributable to the observed differences in the clonality or functionality of the products between patients. The results of this study suggest that the toxicity of an u3TCR product will not be intrinsically different than that of a 1 TCR product and that controlling for total cell dose, regardless of the clonality of the product, is a safe approach to determining the optimal dose for NeoTCR-P1. Consistent with this, PACT proposes use a cell dose escalation scheme (Table 13) and control the total number of gene-edited cells administered to patients in successive cohorts, regardless of the number of TCRs in the product.

4.2.4. Rationale for Evaluating NeoTCR-P1 in Combination with Nivolumab

The rationale for combining NeoTCR-P1 with the anti–PD-1 antibody nivolumab is aimed at reversing the potential loss of functionality of the adoptively transferred lymphocytes by PD-1/PD-L1 immune-inhibitory signaling in the tumor environment. Many of the tumor responses observed with the adoptive transfer of gene-edited T cells have been transient. The inhibitory receptor PD-L1 is known to negatively regulate the signaling and responses of CD8+ T cells expressing PD-1 on their surface.

Blocking the negative immune-regulatory pathway elicited by the co-inhibitory receptor PD-1 results in antitumor activity in some patients with metastatic cancers (Yee et al., 2002; Tumeh 2014; Ribas et al., 2012). Emerging data suggest that the antitumor response to anti–PD-1 blockade is mediated by an increase intratumoral infiltration by CD8+ T cells. Combining anti–PD-1 blockade with NeoTCR-P1 may lead to an increased pool of tumor-infiltrating antigen-specific T cells that could lead to presumed benefit (Cherkassky et al., 2016; Yoon et al., 2018).

4.2.5. Rationale for Dose of Nivolumab

The dose of nivolumab selected for this trial is 480 mg IV administered every 4 weeks for up to *7* doses. This nivolumab flat-dosing regimen has been approved in the United States, Canada, and the European Union as an alternative dosing regimen for several indications. The approval was based on population-pharmacokinetic analyses that established exposure-response relationships and clinical safety (Long et al., 2018).

Nivolumab will be administered for up to *7* cycles as opposed to treatment until progression. The goal of the NeoTCR-P1 development program is to deliver sustained remissions to participants while off therapy. In addition, nivolumab is being administered in this study as a way to potentially induce further expansion of the NeoTCR-P1 T-cell population and will be administered to participants who have already progressed on checkpoint inhibitors or in populations where prior studies have determined that there is limited-to-no benefit (e.g., prostate cancer or MSS CRC).

4.2.6. Rationale for IL-2 Regimen

The classical high-dose IL-2 regimen is associated with transient but potentially severe systemic toxicities (Atkins et al., 1999) *and has been evaluated in multiple TIL and TCR adoptive cell therapy studies at the NCI (Table 11).* Several groups have evaluated low-dose IL-2 in an attempt to mitigate IL-2-related side effects. Ellebaek et al (2012) reported that low-dose IL-2 (14 daily subcutaneous injections, 2 mIU) was well-tolerated by 6 subjects in a pilot trial of patients with metastatic melanoma: patients developed fever, chills a few hours after injection, and nausea and fatigue, none of which exceeded Grade 2 in severity. In a study of 12 patients with metastatic melanoma who were treated with non-myeloablative lymphodepleting chemotherapy, TIL, and low-dose subcutaneous IL-2, Nguyen et al. (2019) reported that low-dose IL-2 (125,000 IU/kg/day administered as an inpatient over a 12-day period with a 2-day break after the first 4–5 doses of IL-2 (maximum 9–10 doses) was relatively well-tolerated. The majority of the toxicities attributed to IL-2 were Grade 1 or 2 events associated with vascular leak syndrome, including peripheral edema, pulmonary edema, hypotension, increased creatinine or decreased urine output, as well as fever, fatigue and neurological symptoms. These events could generally be managed with supportive measures or by delaying or omitting doses of IL-2.

The overall response rates achieved with ACT + low-dose IL-2 in these small studies (2/6 or 33% in Ellebaek et al. (2012); 2/12 or 17%, with 1 additional *unconfirmed response*, in Nguyen et al. (2019) is comparable to that reported in a large institutional experience with 86 consecutive patients with metastatic melanoma (34%; Rosenberg et al., 1994), suggesting that low-dose IL-2 may be equally efficacious when administered following ACT, with potentially decreased toxicity.

Thus, this study will *initially* test the safety of *neoTCR* when administered with subcutaneous IL-2 (500,000 IU/m^2 twice daily for a maximum of 14 doses). *However, following review of safety and PK data from the first 6 participants in the IL-2 basket expansion cohort, the SRT may recommend evaluating a more intensive IL-2 regimen, not to exceed 600,000*

IU/kg IV every 8 hours for a maximum of 15 doses (e.g., Jazaeri er al. 2019, Sarnaik et all, 2018).

Because there is no definitive evidence for the necessity of IL-2 in supporting response to cell-based immunotherapy, IL-2 will be tested in a separate cohort to enable a preliminary assessment of its effects on the safety, pharmacokinetics and anti-tumor activity of NeoTCR-P1.

4.2.7. Rationale for the DLT Criteria and Assessment Window

The DLT assessment window of 28 days following the infusion of NeoTCR-P1 or following the initial dose of nivolumab for participants who receive the combination of NeoTCR-P1 plus nivolumab is expected to allow for the adequate assessment of the nature and incidence of acute toxicities related to the study drug(s). While the toxicities related to gene-edited TCRs likely differ in both timing and severity from CAR-T cells, a DLT window of 28 days was chosen based upon the kinetics of CRS observed in the studies of CAR-T therapies. In Study B2202, 47% (32/68) of participants who received tisagenlecleucel experienced Grade 3/4 CRS with a median onset of 3 days and a range of 1–22 days (Oncologic Drugs Advisory Committee). A larger more comprehensive analysis of 133 adult participants with CD19+ relapsed/refractory B-ALL, CLL, or NHL who received lymphodepletion chemotherapy followed by infusion of CD19 CAR-T cells identified CRS risk factors before and after CAR-T-cell infusion and described the time course of presentation and biomarkers of CRS. In this analysis, the median time to onset of fever (i.e., first objective sign of CRS) was 2.2 days. Compared with participants who had Grade 1 to 3 CRS, participants who had Grade ≥4 CRS experienced fever earlier after CAR-T-cell infusion and the fever peaked earlier. Neurotoxicity typically presented after CRS, with the first neurologic adverse event (AE) of any kind presenting a median of 4 days after infusion of CAR-T cells and a range of 1-22 days (Schreier 2017).

Other short-term adverse events that may occur include infusion-related reactions, chemotherapy adverse events, tumor lysis syndrome (TLS), uncontrolled T-cell proliferation, and potentially acute GVHD-like pathology. Therefore, a DLT window of 28 days should be sufficient to detect acute toxicities and safeguard subsequent patients in the enrollment queue.

4.2.8. Rationale for Collection of Baseline and On-Treatment Biopsy

For the PACT-0101 study, an archival sample may be used to generate the library of candidate mutations for the upstream selection of neoepitope-specific T cells from the peripheral blood that will be used to manufacture the gene-edited TCR product. The protocol allows for product selection in earlier lines of therapy and also allows for bridging therapy to be administered following leukapheresis and prior to conditioning chemotherapy. While selecting clonal or truncal mutations will be critical to the product selection process, there is a potential for a change in the neoantigen landscape during this time, as well as the emergence of resistance mechanisms – such as HLA loss – wherein both mechanisms would result in potential loss of the neoTCR-tumor target (Riaz et al., 2017; Angelova 2018; Paulson et al., 2018). In order to understand whether any shift has occurred, a fresh biopsy is required, if clinically feasible, prior to the start of conditioning chemotherapy in participants who have received intervening therapy from the time of obtaining the tumor tissue used for manufacturing and the start of the study. This tumor tissue will be analyzed as part of a retrospective analysis to understand potential

alterations in the tumor mutational burden during the interval between initial enrollment and NeoTCR-P1 product dosing. Participants whose biopsy is found to be non-evaluable (e.g., insufficient material, low tumor content) will still receive NeoTCR-P1. Participants who cannot be safely biopsied at baseline may still receive NeoTCR-P1, but this option should be discussed with the Medical Monitor.

In order to evaluate the presence of intratumoral T cells and potential trafficking of neoTCR T cells to the site of the tumor, a tumor biopsy will be obtained between Days +5–7 *and/or Days 28–42* after infusion if clinically feasible and safely accessible.

4.2.9. Rationale for Collecting Tumor Specimens at the Time of Progression

Antitumor immune responses, such as those associated with immunotherapy, may result in objective responses that are delayed and can, in some instances, be preceded by initial apparent radiographic progression. This initial apparent progression may occur as a result of either delayed anti-tumor activation and/or robust immune infiltration of the tumor. In addition, lesions that would otherwise be undetectable with conventional imaging (i.e., micrometastatic disease) may increase in size as a result of these processes and be recorded as new lesions (Hales et al., 2010).

If clinically feasible, it is recommended that a tumor biopsy be performed at the time of radiographic progression in order to better understand the biological changes that drive the increase in size of the progressing lesion(s). In addition, mechanisms relating to progression, resistance, predictive, prognostic, and pharmacodynamic relationships in tumor biomarkers (potentially including, but not limited to PD-L1, CD8, and mutation status), and their efficacy will be evaluated. DNA and RNA isolation may also be performed to enable next generation sequencing (NGS) to identify changes in somatic mutations that are associated with disease progression or acquired resistance to NeoTCR-P1 or the combination of NeoTCR-P1 plus nivolumab and to increase the understanding of disease pathobiology.

Other specimens, including but not limited to fluid from ascites and pleural effusions and left-over tissue from standard-of-care or palliative procedures, may be submitted for exploratory analyses with the participant's consent.

4.2.10. Rationale for Conditioning Chemotherapy

Lymphodepletion has been observed in nonclinical models and clinical studies to increase the effectiveness of adoptive cellular therapy. Suboptimal outcomes occurred in clinical trials that did not include lymphodepleting chemotherapy, and other studies have compared cyclophosphamide alone versus the combination of fludarabine and cyclophosphamide (Khalil et al., 2016; Lee et al., 2016; Turtle et al., 2016). Compared with participants who did not receive fludarabine, the following were observed: intensified immunosuppression associated with higher serum concentrations of IL-7 and IL-15 on the day of CAR-T-cell infusion, markedly greater expansion of CD4+ and CD8+ CAR-T cells in the blood in the first 10 days after infusion, and higher numbers of CAR-T cells in blood 1 and 3 months later. The addition of fludarabine was also associated with an improvement in therapeutic efficacy and the depth of response. In a recent CD30 CAR-T-cell study (RELY-30), the expansion of CD30-directed CAR-T cells in patients who received lymphodepleting chemotherapy was approximately

10-fold higher than that observed in patients who did not receive conditioning chemotherapy prior to the infusion of CAR-T cells (Ramos et al., 2019).

Adoptive immunotherapy strategies may be able to capitalize on homeostatic T-cell proliferation (Dummer et al., 2002), a recent finding that naïve T cells begin to proliferate and differentiate into memory-like T cells when total numbers of naïve T cells are reduced below a certain threshold (Goldrath et al., 1999; Surh et al., 2000). Lymphodepletion may also eliminate regulatory T cells and other competing elements of the immune system that act as "cytokine sinks," enhancing the availability of cytokines such as IL-7 and IL-15 (Klebanoff et al., 2005). Finally, lymphodepletion may reduce other immunosuppressive cytokines. Fludarabine and cyclophosphamide have been found to downregulate indoleamine 2, 3-dioxygenase expression in lymphoma cells and in an animal model of CD19 CAR-T improved the antitumor activity of CD19-CAR-T cells *in vivo* (Ninomiya et al., 2015).

Participants in the PACT-0101 study will receive fludarabine (30 mg/m^2 of body surface area) *daily for 4 days (Day -6 through Day -3)* and cyclophosphamide (*600* mg/m^2) daily for 3 days (*Day -6 through Day -4)*. This same conditioning regimen was administered to participants (n=6) in a recent Phase 1 study of anti-*MAGE-A4 TCR therapy (Hong et al., 2020). The total amount of cyclophosphamide under this regimen is approximately 70% less than that administered as part of the typical NCI solid tumor lymphodepleting preparative regimen (e.g., NCT02133196; Zacharakis et al., 2018). All 6 patients in the Hong et al. Phase 1 study experienced Grade ≥3 lymphopenia and neutropenia with no episodes of sepsis or Grade 5 events related to the cytopenias.Five of 6 patients experienced tumor shrinkage, with two RECIST PRs. Thus, this regimen is expected to produce lymphodepletion adequate to support the activity of neoTCR-T cells, while being less intensive than other common conditioning regimens with expected toxicity.*

The study includes sparse sampling for fludarabine PK and pharmacogenetics. Characterizing the association between fludarabine exposure during conditioning therapy and outcome after study treatment may enable individualized model-based fludarabine dosing to optimize the efficacy of NeoTCR-P1 (Sanghavi et al., 2016).

4.2.11. Justification for Starting Dose of NeoTCR-P1

The starting dose for this trial is a single infusion of 4 × 10^8 neoTCR-positive T cells (equivalent to 5 × 10^6 cells/kg for an average participant who weighs 80 kg; https://www.cdc.gov/nchs/data/series/sr_03/sr03_039.pdf) based upon available data from clinical studies of gene-edited TCR studies. In these studies (see Table 16), a similar or higher starting dose was used, and higher doses were well tolerated in later dose-escalation cohorts. A clear response between response and dose of infused TCR-T cells has not yet been established in studies to date, which may be a consequence of the ability of gene-edited TCR-T cells to rapidly proliferate and expand.

Table 16 TCR-Target and Engineering

TCR-Target and Engineering (Reference)	Dose	Number of Subjects Treated	Safety
NY-ESO-1/LAGE-1 (NY-ESO^{c259}) (Rapoport et al., 2015)	≥2 x10^6 CD3+ T cells/kg body weight<br>Mean total dose 2.4×10^9 NY-ESO^{c259}-engineered CD3+ T cells	20	No treatment-related fatalities.<br>Skin rash with lymphocytosis occurred in 3/20 and 3/20 developed GVHD (GI).<br>SAEs included hypoxia, neutropenia, hyponatremia, hypotension, pancytopenia, dehydration
NY-ESO-1/LAGE-1[a] (NY-ESO^{c259}) (D'Angelo et al.2018)	$0.45–14.4 \times 10^9$ cells/m^2 NY-ESO^{c259}-engineered CD3 T cells<br>Median total dose 3.6×10^9	12	No fatal AEs. 11/12 reported TRAEs Grade ≥3.<br>Most common AEs were lymphopenia (100%), leukopenia (92%), neutropenia (83%), anemia (83%), hypophosphatemia (67%), thrombocytopenia (67%), *and* FN (17%). 2/12 participants (17%) experienced CRS.<br>No neurotoxicity reported
EBV-derived tumor antigens (LMP-1 and LMP-2)<br>T cells expressed DNRII<br>(Bollard et al., 2018)	$2 \times 10^7 – 1.5 \times 10^8$ cells/m^2 of DNRII-expressing T cells	8	No DLT occurred. No AI, GVHD, or CRS observed.<br>Most common TRAEs Grade ≥3 were anemia, lymphopenia, leukopenia, and neutropenia each of which occurred in all 7 evaluable participants.
HPV-16 E7 (Norberg et al., 2018)	$1 \times 10^9 – 125 \times 10^9$ CD3 T cells/dose<br>94%–99% E7 T cells/CD3 T cells	12	TRAEs Grade ≥3. Most common AEs were lymphopenia (100%), leukopenia (100%), neutropenia (100%), anemia (100%), thrombocytopenia (67%), FN (67%), hypophosphatemia (50%) pulmonary edema (25%), hypoxia (25%) and AST increased 25%.
NY-ESO-1<br>(Nowicki et al., 2018)	$7.7 \times 10^8 – 1 \times 10^9$ transgenic TCR lymphocytes<br>(pts also received IL-2)	10	No fatal AEs. 9/10 reported TRAEs Grade ≥3.<br>Most common Gr 3-4 TRAEs occurring in >1 participant were neutropenia fever, pancytopenia (6/10), neutropenic fever (5/10), cytokine storm (2/10), neutropenia (2/10), weakness (2/10)
NY-ESO-1<br>(Stadtmauer et al., 2020)	6.0 × 107 to 7.1 × 108 CRISPR-Cas9-engineered T cells (no cytokines were administered)	3	No CRS or overt side effects attributed to cell infusion

AI = aromatase inhibitor; AST = aspartate aminotransferase; DLT = dose-limiting toxicity; DNRII = *dominant negative TGF-β type II receptor*; EBV = Epstein-Barr virus; FN = *febrile neutropenia*; GI = gastrointestinal; GVHD = graft-versus-host disease; HPV = human papilloma virus; LMP = latent membrane protein; SAE = serious adverse event; sCRS = severe cytokine release syndrome; pts = patients; TRAE = treatment-related adverse event.

Unlike standard drugs that are metabolized, gene-edited T cells are “living drugs” that are able to proliferate extensively in participants and, thus, the actual *in vivo* amount of NeoTCR-P1 T cells after engraftment and expansion will vary from participant to participant. Thus, the administered dose may underestimate the *in vivo* amount of NeoTCR-P1 T cells in a given participant.

In this study, 3 different doses (and potentially higher doses if supported by manufacturing) will be evaluated and potential differences in dosing in a non-randomized fashion in the dose-expansion basket cohort will continue to be explored. If new information regarding an optimal dose level and corresponding clinical response becomes available, the trial will be appropriately modified.

An additional consideration that will be important is the ability to manufacture the targeted dose for the majority of participants in the study. In the event that manufacturing feasibility is not met for a particular participant, but it is above the minimum threshold of 1.6×10^7 neoTCR-positive T cells then we will proceed with the infusion. These participants will be included in the safety-evaluable population. Data will be accumulated on a continuous basis in this regard concerning the relationship of cell dose to toxicity and response.

4.3. Rationale for Flat-Fixed Dosing Regimen

Adjustment for weight or body surface area has been used historically to ensure that patients receive a drug dose that is associated with an acceptable degree of toxicity without sacrificing the agent’s therapeutic effects. Retrospective analyses have demonstrated that in many cases such adjustments do not in fact reduce interindividual variation in pharmacokinetics, and flat-fixed dosing regimens are increasingly used, particularly for monoclonal antibodies (Bai et al., 2012; Mathijssen et al., 2007). For monoclonal antibodies, the contribution of body size to pharmacokinetic variability is likely to be minor (Bai et al., 2012; Mathijssen et al., 2007).

For adoptive cellular therapy, the administered cell dose may bear limited to no relationship to the number of cells *in vivo* at later timepoints (i.e., dose-exposure relationship). An analysis of the ELIANA and ENSIGN studies of tisagenlecleucel showed no relationship between the product transduction efficiency, cell viability and total T cells across a wide range of doses and patient weights (Mueller et al., 2018). The degree of expansion may show significant interindividual variation depending on the phenotypic characteristics of the starting cellular material composition at the start of the engineering, manufacturing process, antigen availability, and pre-infusion tumor burden (Elavia et al., 2019; Mueller et al., 2017; Blair et al., 2011). These downstream sources of inter-individual pharmacokinetic variability may outbalance body weight-based dose normalization measures. Nonetheless several studies have noted that the number of infused cells is associated with the likelihood of clinical response (e.g., Radvanyi et al., 2012), and many adoptive cell therapy trials continue to use a traditional dose escalation design to define the optimal product dose. However, in the majority of cases a flat-fixed cell dose is tested (e.g., Hont et al., 2019; Doran et al., 2019).

There is no evidence that changes in ACT-cell therapy doses resultant from different weights in adult populations will increase treatment-related toxicity, in particular considering that other ACT-cellular products using TCR-transgenic T cells or TIL therapy have been administered at

total cell doses several orders of magnitude greater than the highest doses proposed to be evaluated in this study (e.g., Doran et al., 2019; Dudley et al., 2005, 2008).

Flat-fixed dosing has practical advantages compared to weight- or body surface area-based dosing, including improved manufacturing predictability and the elimination of potential dose calculation errors.

4.4. End of Study and Length of Study

A participant is considered to have completed the study if he/she has completed all phases of the study including the last scheduled procedure as shown in the SOA.

The end of the study is defined as the date of the last participant last visit shown in the SOA for the last participant in the trial globally (see Section 1.4).

Additionally, the Sponsor may decide to terminate the study at any time.

The total length of the Initial Phase 1a portion of the study, from screening of the first participant to the end of the study (excluding LTFU), is expected to be approximately 36 months.

5. Study Population

Eligibility criteria are designed to include a broad spectrum of participants with a limited number of solid tumors across the tumor mutational spectrum who have no available curative treatment options and who have a limited prognosis with currently available therapies.

During Screening Part 1, participants will be comprehensively evaluated to ensure they do not have irreversible disqualifying conditions that would prohibit the administration of experimental treatment. To decrease the risk of significant decline during product selection and manufacturing, participants should have very good performance status and organ function at this time.

Part 1 performance status, medical and cancer history, and end organ function eligibility criteria should be met before blood collection for PBMC isolation and submission of tumor tissue for TCR selection.

If TCR selection is successful, re-evaluation of the participant will take place prior to leukapheresis (Screening Part 2 eligibility assessment). The echocardiogram (ECHO), electrocardiogram (ECG), pulmonary function test (PFT), tumor assessment, and brain magnetic resonance imaging (MRI) must be performed during Screening Part 2.

Note: Participants who enter this study with neoTCR product candidates identified under a separate screening protocol are not required to undergo Screening Part 1 eligibility assessments or data collection, and will enter this study at Screening Part 2 (see Section 5.3 Leukapheresis Criteria).

Additional criteria should be evaluated (see Section 6.1.3 for details) prior to the infusion of conditioning chemotherapy.

Participants enrolled in this study are permitted to participate in parallel institutional biomarker studies (e.g., analysis of gut microbiome).

Prospective approval of protocol deviations and eligibility exceptions, also known as protocol waivers or exemptions, is not permitted.

5.1. *Screening Part 1* Inclusion Criteria

Participants must meet the following criteria within 28 days after the date of informed consent in order to proceed with product selection activities:

1) *Age ≥18 years of age at the time of signing informed consent*
2) *Histologically or cytologically documented incurable or metastatic solid tumors of the following types: cutaneous melanoma, urothelial carcinoma, NSCLC, HNSCC, ovarian cancer, CRC, breast cancer (HER2-negative), or prostate cancer.*

   Participants who are receiving adjuvant therapy or are under observation following adjuvant therapy for high risk locally advanced disease may be eligible following discussion with Medical Monitor.

   The number of participants with specific tumor types may be controlled to allow adequate representation of other tumor types.
3) *Life expectancy >6 months*
4) *Eastern Cooperative Oncology Group (ECOG) performance status of 0 or 1*
5) *Adequate hematologic and end organ function determined defined as the following:*
   - *ANC ≥1500/μL without granulocyte colony-stimulating support within 14 days*
   - *Absolute lymphocyte count (ALC) ≥500/μL*
   - *Platelet count ≥100,000/μL without transfusion within 14 days*
   - *Hemoglobin ≥9 g/dL without transfusion within 14 days*
   - *Creatinine clearance (as estimated by Cockcroft Gault) ≥60 mL/min*

     Participants with creatinine clearance <60 mL/min but ≥50 mL/min may be eligible if they meet all the following criteria:
     - *Have serum creatinine and blood urea nitrogen (BUN) levels within normal limits (per local ranges)*
     - *No evidence of chronic renal insufficiency (e.g., acidosis or electrolyte abnormalities)*
     - *Normal urinalysis and urine output (per local ranges)*
     - *Negative urine cultures*
     - *Renal ultrasound negative for hydronephrosis or other renal pathology*

     Note: Participants with baseline creatinine clearance <60 mL/min may not receive IL-2 as part of their experimental treatment, but can receive neoTCR P1 in combination with nivolumab
   - *Serum AST/ALT ≤2.5 times the ULN*

- *Total bilirubin ≤1.5 × ULN (except for participants with documented Gilbert's disease who have serum bilirubin level ≤3 × ULN)*
- *Baseline oxygen saturation >92% on room air*
- *Serum albumin ≥3.0 g/dL*

6) *Able to provide adequate tumor tissue for sequencing based on total and viable tumor content.*

   If archival tissue is provided, it must have been obtained within 1 year prior to consent, unless otherwise agreed to by the Sponsor/Medical Monitor

7) *Willing and able to undergo a leukapheresis procedure*

5.2. Screening Part 1 Exclusion Criteria

Participants who meet any of the following will be excluded from study:

1) *More than two systemic chemotherapy regimens for advanced disease*

   Participants may be enrolled during their first or second regimen or after completing a second regimen, but may not have started a third chemotherapy regimen:

   - *Regimens are defined by interval progressions*
   - *In participants who relapsed within 6 months after completing (neo)adjuvant chemotherapy for early stage disease, that regimen will count towards the allowed maximum of two*
   - *More heavily pretreated participants who meet all other eligibility criteria may be enrolled by exception with the approval of the Medical Monitor*

   There is no restriction on the number of prior targeted or biological therapies, including immunotherapy

2) *History of malignancies other than the disease under study within 2 years prior to enrollment except for nonmelanoma skin cancer, carcinoma in situ (e.g., cervix, bladder, breast), or very low risk prostate cancer as defined by the National Comprehensive Cancer Network guidelines that is under active surveillance.*

3) *Known clinically significant liver disease, including active viral, alcoholic, or other hepatitis, cirrhosis, and/or inherited liver disease*

4) *History of myocardial infarction, cardiac angioplasty or stenting, unstable angina, active atrial fibrillation requiring therapy, symptomatic sinus bradycardia (resting heart rate <50 beats per minute), or other clinically significant cardiac disease within 6 months*

5) *History of symptomatic deep venous thrombosis or pulmonary embolism requiring anticoagulation within 6 months of enrollment*

6) *Cardiac involvement of primary malignancy*

7) *Known primary CNS malignancy or symptomatic CNS metastases*

8) *Symptomatic or uncontrolled pleural effusion, pericardial effusion, or ascites requiring recurrent drainage procedures (once monthly or more frequently)*
   - *Participants with indwelling catheters (e.g., PleurX®) are allowed regardless of drainage frequency*
9) *Percutaneous nephrostomy, indwelling Foley catheter, Ommaya reservoir or other intraventricular drains, biliary drains*
   - *If treating physician feels that the above indwelling catheters/drains are a temporary measure and can be discontinued prior to leukapheresis, participants may be enrolled after discussion with the Medical Monitor*
   - *Port-a-cath or peripherally inserted central catheter (PICC) or Hickman catheters will be allowed.*
10) *Uncontrolled or symptomatic hypercalcemia*
11) *Primary immunodeficiency*
12) *Active HIV, Hepatitis B, or Hepatitis C infection*
13) *Prior allogeneic stem cell transplant and/or solid organ transplant*
14) *Autologous stem cell transplant within 6 months prior to enrollment*
15) *Prior chimeric antigen receptor therapy or other genetically modified T-cell therapy*

    Prior treatment with neoantigen vaccines is acceptable, provided that a tumor specimen obtained after the completion of treatment is provided for sequencing.
16) *Known hypersensitivity or contraindication to dimethyl sulfoxide (DMSO) or any of the agents used in the administration of potential investigational treatment (i.e., cyclophosphamide and fludarabine or their components).*
17) *History or risk of autoimmune disease (see Appendix 9.2 for comprehensive list of excluded pre-existing autoimmune diseases). The following exceptions may be eligible:*
   - *Participants with a history of hypothyroidism receiving a stable dose of thyroid replacement hormone may be eligible*
   - *Participants with controlled Type 1 diabetes mellites receiving a stable insulin regimen may be eligible*
18) *Chronic steroid therapy for adrenal insufficiency, hypophysitis, or other conditions*
19) *History of idiopathic pulmonary fibrosis or active restrictive or obstructive pulmonary disease*
20) *Participants with illnesses or conditions that interfere with their capacity to understand, follow, and/or comply with study procedures*
21) *Any other diseases, metabolic dysfunction, physical examination finding, or clinical laboratory test giving reasonable suspicion of a disease or condition that contraindicates the use of an investigational drug or that may affect the interpretation of the results or render the participant at high risk from treatment-related complications*

5.3. *Screening Part 2 Inclusion Criteria*

Screening Part 2 is gated by the identification of TCRs either during Screening Part 1 of this study, or under a separate screening protocol.

Participants must *be assessed against* the following criteria during Screening Part 2.

Participants should not experience a significant change in clinical or performance status compared to initial eligibility criteria (assessed during Screening Part 1, or at the time of enrollment in the screening protocol).

If any results are beyond the criteria established for initial eligibility or the thresholds listed below, participants may not undergo leukapheresis until the abnormalities have resolved unless otherwise agreed to by Sponsor/Medical Monitor.

General Criteria

1) *Actionable TCRs identified during Screening Part 1 of this study or under a separate screening protocol*
2) *Signed informed consent from participants who enter the study at Screening Part 2 with TCRs identified under a separate screening protocol*
3) Life expectancy >6 months
4) Eastern cooperative oncology group (ECOG) performance status of 0 or 1
5) Adequate hematologic and end organ function determined within 30 days prior to *leukapheresis* defined as the following:
   - *ANC ≥1000*/µL without granulocyte colony-stimulating support within 14 days
   - Absolute lymphocyte count (ALC) ≥300/µL
   - Platelet count ≥*75*,000/µL without transfusion within 14 days
   - Hemoglobin ≥9 g/dL without transfusion within 14 days
   - Creatinine clearance (as estimated by Cockcroft Gault) ≥60 mL/min

     Participants with creatinine clearance <60 mL/min but ≥50 mL/min may be eligible if they meet all the following criteria:
     - *Have serum creatinine and BUN levels within normal limits (per local ranges)*
     - *No acidosis and normal electrolytes*
     - *Normal urinalysis (per local ranges) and negative urine cultures*
     - *Normal urine output*
     - *Renal ultrasound negative for hydronephrosis or other renal pathology*

     Note: Participants with baseline creatinine clearance <60 mL/min may not receive IL-2 as part of their experimental treatment, and will be assigned to receive neoTCR-P1 ± nivolumab.

- Serum AST/ALT ≤2.5 times the ULN
- Total bilirubin ≤1.5 × ULN (except for participants with documented Gilbert's disease who have serum bilirubin level ≤3 × ULN)
- Cardiac ejection fraction ≥45%
- Baseline oxygen saturation >92% on room air

6) Willing and able to remain within approximately a 60-minute travel radius (during peak traffic) from the treatment site for 28 days following the infusion of neoTCR-P1

   Participants who live beyond the 60-minute radius must agree to hoteling within close proximity to the treatment site for 28 days following the infusion of neoTCR-P1.

7) Willing and able to undergo a leukapheresis procedure

8) Women of childbearing potential (WOCBP) must have a negative serum or urine pregnancy test (females who have undergone surgical sterilization or who have been postmenopausal for least 2 years are not considered to be of childbearing potential) and agree to remain abstinent (refrain from heterosexual intercourse) or use contraceptive methods that result in a failure rate of <1% during the treatment period. For those women participating in the Phase 1b this period extends to at least 5 months following the last administration of anti–PD-1.

9) Men must agree to remain abstinent (refrain from heterosexual intercourse) or use contraceptive measures, as defined below:

   With female partners of childbearing potential, men must remain abstinent or use a condom during treatment and for at least 6 months after the last dose of NeoTCR-P1 or 5 months after the last dose of nivolumab, whichever comes later.

10) *Measurable disease per RECIST v1.1*
    - *During Phase 1a dose escalation, additional participants with evaluable disease only (i.e., without measurable disease but able to be evaluated by means of serum tumor markers or non-measurable lesions [e.g., peritoneal carcinomatosis, bone disease only]) may be eligible following discussion with the Medical Monitor*
    - *Disease-specific criteria will be used for participants with prostate cancer (see below)*

11) *Toxicities due to prior therapy must be stable and recovered to Grade ≤1 (except for clinically non-significant toxicities such as alopecia)*

Cancer-Specific Criteria

In addition to the criteria above, participants must meet the following disease-specific criteria:

Melanoma

M1) Unresectable stage III or metastatic melanoma not amenable to local therapy

M2) Participants may not have a diagnosis of uveal or ocular melanoma.

M3) Prior treatment with pembrolizumab, nivolumab, or other approved anti-PD-1 or anti-PD-L1 agents.

- Participants who received adjuvant PD-1 therapy who then develop measurable disease within 6 months of their last dose of PD-1 blockade are eligible for enrollment in this study and not required to receive anti-PD-1 in the metastatic setting

M4) Participants must have testing for a BRAF mutation prior to study entry. Participants with *BRAF*^{V600} mutant-positive melanoma should have received prior BRAF inhibitor therapy. At the discretion of the investigator, participants with *BRAF*^{V600} mutant melanoma who have NOT received a BRAF inhibitor are also eligible for this study if they meet the following additional criteria:

- Lactate dehydrogenase (LDH) < local ULN
- No clinically significant tumor related symptoms in the judgment of the investigator
- Absence of rapidly progressing metastatic melanoma in the judgment of the investigator

Urothelial Carcinoma

UC1) Locally advanced (inoperable) or metastatic transitional cell carcinoma of the urothelium (including renal pelvis, ureters, urinary bladder, and urethra)

UC2) Participants may not have a diagnosis of small cell, urachal, primary adenocarcinoma, or squamous cell carcinoma

UC3) Previous treatment with at least 1 platinum-containing regimen (e.g., GC, MVAC, carboplatin and gemcitabine [CarboGem]) and/or an approved anti-PD-1 or anti-PD-L1 agent (e.g., atezolizumab, pembrolizumab, nivolumab)

- Participants who received prior adjuvant/neoadjuvant chemotherapy and progressed within 12 months of treatment with a platinum-containing adjuvant/neoadjuvant regimen may be eligible

Non-Small Cell Lung Cancer

LC1) Metastatic stage disease or recurrent/locally advanced stage not amenable to definitive surgical or radiation therapy

LC2) Previously received treatment with at least one standard systemic therapy for metastatic/recurrent stage disease per *National Comprehensive Cancer Network (*NCCN*)* or institutional practice

- Participants who develop metastasis/recurrence within 6 months of completing adjuvant or neoadjuvant chemotherapy will be considered to have met this criterion

LC3) Previously received anti-PD-1 or anti-PD-L1 therapy.

- Participants who received neoadjuvant or adjuvant anti-PD-1 or PD-L1 therapy will be considered to have met this criterion
- Participants with activating mutations or re-arrangements for which FDA-approved tyrosine kinase inhibitors exist are exempt from this criterion

LC4) Participants with an activating mutation or rearrangement for which there is a first-line FDA-approved tyrosine kinase inhibitor must have experienced disease progression (during or after treatment) or intolerance to treatment with at least one appropriate tyrosine kinase inhibitor

Head and Neck Squamous Cell Cancer

HN1) Recurrent and/or metastatic squamous cell carcinoma of the head and neck (both HPV-positive and negative) that is not appropriate for curative-intent therapy

HN2) Prior treatment with pembrolizumab, nivolumab, or other approved anti-PD-1 or anti-PD-L1 agents.

Colorectal Cancer

CC1) Histologically or cytologically confirmed metastatic or recurrent colorectal carcinoma

CC2) Previous treatment with fluoropyrimidine-based regimen that includes either oxaliplatin (e.g., FOLFOX, CAPEOX) or irinotecan (e.g., FOLFIRI)

CC3) Participants must have testing for mismatch repair prior to study entry. *Participants* with dMMR/MSI-H should have received prior treatment with pembrolizumab, a nivolumab-containing regimen, or other approved anti-PD-1, or anti-PD-L1 agents

Ovarian Cancer

OC1) Histologically and/or cytologically confirmed high grade serous epithelial cancer of the ovary, fallopian tube, or peritoneum, including malignant mixed Müllerian tumors with high-grade serous component

- Non-epithelial tumor or ovarian tumors with low malignant potential (i.e., borderline tumors) are not eligible

OC2) Platinum resistant/refractory disease, defined as disease progression within 6 months following the last administered dose of platinum therapy or lack of response or disease progression while receiving the most recent platinum-based therapy, respectively

OC3) Participants with platinum-sensitive disease, defined as disease progression occurring greater than 6 months following the last administered dose of first-line platinum chemotherapy must also have received niraparib, olaparib, rucaparib, or other approved PARP inhibitors

Hormone Receptor-Positive Breast Cancer

BC1) Histologically and/or cytologically confirmed diagnosis of estrogen-receptor positive (ER+) and/or *PR*+ and HER2-negative breast cancer by local pathology assessment

BC2) Participant has received ≥2 endocrine therapies for the treatment of advanced/MBC at least 1 of which was in combination with a CDK4/6 inhibitor

Participants with hormone-unresponsive disease treated with only 1 prior endocrine therapy regimen may be eligible.

BC3) Participant has received ≥1 chemotherapy regimen for the treatment of advanced/MBC

BC4) Absence of rapid clinical progression, life-threatening visceral metastasis or the need for rapid symptom and/or disease control, which may require combination chemotherapy

Triple Negative Breast Cancer

TN1) Histologically and/or cytologically confirmed diagnosis of TNBC, defined as absence of HER2, ER, and PR expression by local pathology assessment

Participants with ER Low Positive tumors (1%–10% ER positive cells; Allison et al. 2020) may be eligible if they are clinically treated as having TNBC.

TN2) Participant has received >1 chemotherapy regimen for the treatment of advanced/MBC

TN3) Previous treatments must have included a taxane

Participants who received neoadjuvant or adjuvant taxane therapy will be considered to have met this criterion.

TN4) Absence of rapid clinical progression, life-threatening visceral metastasis or the need for rapid symptom and/or disease control, which may require combination chemotherapy

Prostate Cancer

PC1) Histologically or cytologically confirmed adenocarcinoma of the prostate without small cell histology

PC2) Participant has measurable prostate cancer on computed tomography (CT) or MRI scans for ORR by RECIST v1.1 and/or detectable bone metastases by whole body bone scintigraphy evaluable by Prostate Cancer Working Group 3 (PCWG3) criteria.

PC3) Participant has either metastatic or locally confined inoperable disease that cannot be treated with definitive intent.

PC4) Participant has serum testosterone level <50 ng/dL. Castrate testosterone levels (testosterone concentration of ≤50 ng/dL) must be from orchiectomy AND/OR current therapy with luteinizing hormone-releasing hormone agonist.

PC5) Participant has evidence of progressive disease since the most recent change in therapy. Progressive disease is defined as any 1 of the following:

- Radiographic progression in measurable disease (RECIST v1.1 criteria) with or without PSA progression.

- Bone scan progression: Appearance of 2 or more new lesions on bone scan (Tc-99m MDP) attributable to prostate cancer
- PSA progression: Sequence of rising values measured at least 1 week apart. Minimum starting value 2 ng/mL. If PSA increase is the only indication of progression 1 ng/mL is the minimum starting value.

PC6) Participant has progressive castration-resistant prostate cancer despite prior treatment with docetaxel or systemic agents that interfere with androgenic signaling (e.g., enzalutamide, abiraterone, apalutamide)

5.4. *Screening Part* 2 Exclusion Criteria

Participants who meet any of the following may *not proceed to leukapheresis*:

1) Any anti-cancer therapy within 2 weeks or 5 half-lives, whichever is shorter, prior to planned leukapheresis.
    - The following exceptions to the above are allowed:
        - Hormonal therapy with gonadotropin-release hormone (GnRH) agonists or antagonists for prostate cancer
        - Hormone-replacement or oral contraceptives
2) Known primary CNS malignancy or symptomatic CNS metastases

    Participants with treated CNS disease may be enrolled after consultation with the Medical Monitor provided all of the following criteria are met:
    - Evaluable or measurable disease outside the CNS
    - No history of intracranial hemorrhage or spinal cord hemorrhage
    - No ongoing corticosteroid requirement for CNS disease within 14 days prior to enrollment
    - Participants receiving a stable dose of anticonvulsants are permitted
    - No stereotactic radiation or whole brain radiation within 14 days prior to enrollment
3) Pregnancy, lactation, or breastfeeding
4) Treatment with or potential requirement for systemic corticosteroids (>5 mg prednisone equivalent per day) or immunosuppressive medications within 7 days *prior to* leukapheresis
    - Inhaled or topical steroids at standard doses are permitted
    - Participants who have received a one-time dose of corticosteroids (e.g., dexamethasone for nausea, contrast allergy) may be enrolled in the study after discussion with and approval by the Medical Monitor

5) Pulmonary function test abnormalities as evidenced by a forced expiratory volume in one second (FEV1)/forced vital capacity (FVC) <70% of predicted for normality
6) Any diseases, metabolic dysfunction, physical examination finding, or clinical laboratory test giving reasonable suspicion of a disease or condition that contraindicates the use of an investigational drug or that may affect the interpretation of the results or render the Participant at high risk from treatment-related complications
7) Severe infection within 2 weeks prior to *leukapheresis*.
8) Major surgical procedure within 4 weeks prior to enrollment or anticipation of need for a major surgical procedure, including for impending oncologic emergency, during the study.
   - Participants undergoing minor surgical procedure (e.g., venous access for pheresis) are eligible *to proceed*

Exclusion criteria specific to participants in the Phase 1b only:

9) Administration of a live attenuated vaccine (e.g., *measles, mumps, rubella* [MMR*]*, rotavirus, varicella) within 2 weeks before enrollment or anticipation that such a live attenuated vaccine will be required during the study or within 5 months following the last dose of nivolumab

5.5. Screen Failures

Screen failures are defined as participants who sign the informed consent but are not subsequently enrolled. *Enrollment is defined as meeting as meeting Screening Part 2 eligibility criteria and approved for leukapheresis.*

A minimal set of screen failure information is required to ensure transparent reporting of screen failure participants to meet the Consolidated Standards of Reporting Trials (CONSORT) publishing requirements and to respond to queries from regulatory authorities. Minimal information includes demography, screen failure details, eligibility criteria, and any SAE.

Individuals who do not meet the criteria for participation in this study (screen failure) may be rescreened. Rescreened participants should be assigned the same participant number as for the initial screening (see Section 8.5.2).

6. Study Intervention Terminology

The following terms will be used to describe and define protocol treatment:

- The conditioning chemotherapy regimen used for this study will be fludarabine and cyclophosphamide
- The investigational products (IPs) for this study are neoTCR-P1, IL-2, and nivolumab
- The term study treatment refers to all protocol-related therapies

6.1. Study Intervention(s) Administered

6.1.1. Conditioning Chemotherapy

Conditioning chemotherapy will be supplied by the investigative site unless otherwise noted. Refer to the current product label for guidance on packaging, storage, preparation, administration, and toxicity management associated with the administration of each of these chemotherapy agents. A list of conditioning chemotherapies is presented in Table 17.

NeoTCR-P1 is administered after a conditioning chemotherapy regimen consisting of fludarabine 30 mg/m^2/day × *4* days administered IV, and cyclophosphamide *600* mg/m^2/day administered IV × 3 days. Maximum dose calculated based on actual body weight should not exceed 140% of doses calculated on ideal body weight. Adjustments to the conditioning chemotherapy dose should be discussed with the Medical Monitor. Mesna and antimicrobial prophylaxis should be administered per institutional guidelines.

Table 17 Conditioning Chemotherapies

Name	Conditioning Chemotherapy	Conditioning Chemotherapy	Cytoprotective agent, Noncytotoxic
**Intervention Name**	Fludarabine	Cyclophosphamide	Mesna
**Type**	Synthetic purine nucleoside	Nitrogen mustard-derivative alkylating agent	Synthetic sulfhydryl compound that protects the bladder from urotoxic metabolites
**Dosage Level(s)**	30 mg/m^2/day for *4* days	*600* mg/m^2/day for 3 days	Per institutional guidelines
**Route of Administration**	IV infusion	IV infusion	IV infusion
**Use**	Refer to the most recent version of the package insert for specific details surrounding the administration of fludarabine	Refer to the most recent version of the package insert for specific details surrounding the administration of cyclophosphamide	Mesna should be administered per institutional guidelines. Refer to the most recent version of the package insert for specific details surrounding the administration of cyclophosphamide
**Sourcing**	Provided locally by the trial site	Provided locally by the trial site	Provided locally by the trial site
**Current/Former Name(s)**	Fludara®	Cytoxan®, Neosar®	Mesnex®

IV = intravenous; IMP = investigational medicinal product; NIMP = non-investigational medicinal product.

6.1.2. NeoTCR-P1

The neoTCR-P1 drug product produced using the Phase 1 manufacturing process is cryopreserved in *single-use bags*. Depending upon the dose and the number of distinct neoTCRs in the product, 2-6 bags may be shipped to the site for each participant.

The final product will contain 5% DMSO [redacted].

For further details, see the neoTCR-P1 Investigator's Brochure and Investigational Product Manual.

6.1.3. *Baseline* Evaluation *Prior To* Conditioning Chemotherapy and NeoTCR-P1 T Cell *Infusion*

Prior to the infusion of conditioning chemotherapy participants must be reassessed by the treating physician per the SOA (see Section 1.4).

- Participants should not experience a significant change in clinical or performance status compared to initial eligibility criteria (assessed during Screening Part 2) that, in the opinion of the treating physician, would increase the risks of experimental cell infusion. See Section 8.5.5 for further details.
- In addition, participants experiencing significant toxicities from their preceding conditioning chemotherapy will have their neoTCR-P1 infusion schedule delayed until these toxicities have resolved. The specific toxicities warranting delay of T-cell infusions are detailed in Section 8.5.55.
- *Laboratory panels and tumor assessment must be performed prior to conditioning chemotherapy to establish a baseline (see Table 2 for assessment windows)*

6.1.3.1. Premedication

Participants will receive the following premedication approximately 60 minutes prior to the infusion:

- Acetaminophen 650 mg *oral*
- Diphenhydramine 12.5–25 mg IV (or another H1 antihistamine)

Following the infusion, acetaminophen and diphenhydramine may be repeated every 6 hours as needed. A course of nonsteroidal anti-inflammatory drugs (NSAIDs) may be prescribed if the participant continues to have fever not relieved by acetaminophen. Participants will also receive 1 liter of IV hydration, per institutional standard, prior to the infusion to ensure adequate hydration. It is recommended that participants NOT receive systemic corticosteroids at any time, except in the case of a life-threatening emergency, since this may have an adverse effect on T cells.

6.1.3.2. Treatment Regimen

A single dose of neoTCR-P1 T cells will be administered by intravenous infusion. The infusion will be scheduled to occur approximately 2 days following the completion of chemotherapy.

6.1.3.3. Accountability

NeoTCR-P1 will be prepared by the CPF and released by the Sponsor to the study site after all required safety and release criteria have been met. The investigator or designee must confirm appropriate temperature conditions have been maintained during transit for all study intervention received and any discrepancies are reported and resolved before use of the study intervention. The investigator, institution, or the head of the medical institution (where applicable) is

responsible for study intervention accountability, reconciliation, and record maintenance (i.e., receipt, reconciliation, and final disposition records).

6.1.3.4. Storage

The neoTCR-P1 cell product should be kept in the vapor phase of liquid nitrogen until infusion. For additional details related to storage, see the Investigational Product Manual.

6.1.3.5. Administration

Administration of neoTCR-P1 will be performed in an inpatient hospital setting. Emergency medical equipment (i.e., emergency trolley) will be available during the infusion in the event the participant has an allergic response, or severe hypotensive crises, or any other reaction to the infusion.

Following administration in approximately 20 participants and a review by the SRT, consideration may be given for administration of neoTCR-P1 in an extremely closely monitored outpatient setting with transplant or prior outpatient CAR-T-cell transplant experience.

The target starting dose of neoTCR-P1 is 4×10^8 neoTCR-positive T cells.

NeoTCR-P1 will be delivered by rapid intravenous infusion at 10 mL to 20 mL per minute. A leukoreduction filter **must not be used for the infusion of the T-cell product**. The duration of the infusion will be based on the total volume to be infused and the recommended infusion rate. Each infusion bag will have affixed to it a label containing the following "FOR AUTOLOGOUS USE ONLY." In addition, the label will have at least 2 unique identifiers. Prior to the infusion, 2 individuals will independently verify all this information in the presence of the participant and so confirm that the information is correctly matched to the participant.

Following the infusion, each infusion bag should be rinsed with normal saline while maintaining a closed tubing system to assure as many cells as possible are infused into the participant.

If a participant is to receive a 2 or 3 TCR product, a minimum 15-minute interval must be observed between the administration of separate $TCR\ products$. Vital signs will be assessed in the interval between infusions of successive clones. If the participant experiences a Grade ≤2 infusion-related reaction during the infusion of the cell product (e.g., prior to administration of a second infusion bag, if applicable), administer aggressive symptomatic treatment (e.g., oral or IV antihistamine, anti-pyretic medication, glucocorticoids, epinephrine, bronchodilators, oxygen). After symptoms have resolved to baseline, the infusion may be resumed at a slower rate. Do not resume infusion in the event of Grade 3 or 4 infusion-related reaction. The Medical Monitor should be contacted to discuss the management of participants who experience Grade >2 infusion-related reactions.

The participant's vital signs (heart rate, respiratory rate, blood pressure, temperature) and pulse oximetry (oxygen saturation) will be assessed prior to dosing, and at the end of the infusion (Table 27). If the participant's vital signs are not satisfactory and stable, vital signs and pulse oximetry will continue to be monitored at a minimum of every hour or as clinically indicated until stable.

For additional details related to administration, see the Investigational Product manual.

6.1.3.6. Interleukin-2 (aldesleukin, Proleukin, recombinant human Interleukin 2)

6.1.3.6.1. Description

Human recombinant IL-2 is a highly purified protein with a molecular weight of approximately 15,300 daltons. It is a lymphokine produced by recombinant DNA technology using a genetically engineered E. coli strain containing an analog of the human IL-2 gene.

6.1.3.6.2. Administration

Participants in designated cohorts will receive IL-2 at *the prescribed dose per protocol* for 7 days *(see Section 4.1 under "Phase 1a Expansion (u3TCR)".*

Doses will be skipped for Grade 3 or 4 toxicity due to IL-2 except for Grade 3 toxicities common to IL-2 that are reversible within 12 hours, such as diarrhea, nausea, vomiting, medically manageable hypotension, skin changes, anorexia, mucositis, dysphagia, or constitutional symptoms and laboratory changes.

Refer to Section 6.7.3 for IL-2 dose modifications for toxicity.

For further details, including a detailed description of potential safety risks for aldesleukin, refer to the Proleukin® USPI or SmPC.

6.1.4. Nivolumab

6.1.4.1. Description

Nivolumab is an anti–PD-1 monoclonal antibody.

6.1.4.2. Administration

Nivolumab 480 mg *first dose* will be administered *by IV infusion 1 day after* neoTCR-P1 *infusion*. Nivolumab will be administered every 4 weeks intravenously × 7 doses.

Participants may temporarily suspend study treatment with nivolumab as appropriate for management of toxicity. The known MOA and the potential overlap with neoTCR-P1 toxicity (i.e., autoimmunity) may make it challenging to distinguish causality for participants in the Phase 1b. Potential immune-mediated adverse events may be due to 1 or both agents in the Phase 1b. If causality cannot be determined, then attribution should be attributed to both agents in the Phase 1b.

6.2. Treatment Assignment

This is an open-label study. Once all required screening test results are available and eligibility has been confirmed, the study site will be required to fax or email to PACT Pharma information regarding the participant's eligibility for approval prior to enrollment.

Participants will be assigned to dose levels and dose cohorts prior to starting conditioning chemotherapy.

6.3. IP Accountability and Study Intervention Compliance

NeoTCR-P1 will be provided by the Sponsor. All other IPs (i.e., fludarabine, cyclophosphamide, IL-2, and nivolumab) will be sourced from commercial supply. The study site will acknowledge the receipt of Sponsor-provided IP with Sponsor. Any damaged shipments will be replaced.

IPs will either be disposed of at the study site according to the study site's institutional standard operating procedure or returned to the Sponsor with the appropriated documentation. The site's method of IP destruction must be agreed to by the Sponsor. The site must obtain written authorization from the Sponsor before any IP is destroyed, and IP destruction must be documented on the appropriate from.

Accurate records of all Sponsor-provided IPs received at, dispensed from, returned to, and disposed of by the study site should be recorded on the Drug Inventory Log.

When participants are dosed at the site, they will receive study intervention directly from the investigator or designee, under medical supervision. The date and time of each dose administered in the clinic will be recorded in the source documents and recorded in the eCRF. The dose of study intervention and study participant identification will be confirmed at the time of dosing by a member of the study site staff other than the person administering the study intervention.

Further guidance and information for the final disposition of unused study interventions are provided in the Investigational Product Manual.

6.4. Concomitant Therapy

Any medication or vaccine (including over-the-counter or prescription medicines, vitamins, and/or herbal supplements) or other specific categories of interest that the participant is receiving at the time of enrollment or receives during the study must be recorded along with:

- Reason for use
- Dates of administration including start and end dates
- Dosage information including dose and frequency

During the course of the study, investigators may prescribe any concomitant medications or treatment deemed necessary to provide adequate supportive care except those medications listed in Excluded Medications (see Section 6.6).

The Medical Monitor should be contacted if there are any questions regarding concomitant or prior therapy

All concurrent therapies, including medications, intubation, dialysis, oxygen, and blood products, will be recorded from 7 days prior to Screening Part 2 of screening through 90 days after completing treatment with neoTCR-P1 or the combination of neoTCR-P1 and nivolumab. After 90 days of follow-up, only targeted concomitant medications will be collected for 24 months

after neoTCR-P1 infusion or disease progression, whichever occurs first. Targeted concomitant medications include immunosuppressive drugs and anti-infective drugs.

For participants who are enrolled but not dosed with neoTCR-P1, concurrent therapies will only be recorded from 7 days prior to Screening Part 2 through 30 days after the last study specific procedure (e.g., leukapheresis, conditioning chemotherapy) For participants who are not enrolled (e.g., screen failure, not leukapheresed), only concurrent therapies related to any *serious adverse events (*SAEs*)* will be recorded.

Specific concomitant medication collection requirements and instructions are included in the eCRF completion guidelines.

6.5. Bridging Therapy

Bridging therapy is permitted at the discretion of the investigator in the interval between the completion of leukapheresis and up to 2 weeks prior to the start of conditioning chemotherapy. Investigational therapy is not allowed in this interval. In participants who receive bridging therapy, tumor assessments must be reassessed to establish a new baseline prior to the start of conditioning chemotherapy. Participants must have evidence of measurable disease on their repeat baseline assessments in order to proceed with conditioning chemotherapy.

6.6. Excluded Medications

Corticosteroid therapy at a pharmacologic dose (>5 mg/day of prednisone equivalent) and other immunosuppressive drugs must be avoided for 7 days prior to leukapheresis, and 5 days prior to neoTCR-P1 administration.

Corticosteroids and other immunosuppressive drugs *(e.g., cyclophosphamide, cyclosporine, azathioprine, methotrexate, thalidomide, and thalidomide analogues)* should also be avoided for 3 months after NeoTCR-P1 administration, unless used to manage neoTCR-P1 related toxicities. Other medications that might interfere with the evaluation of the investigational medical product, such as non-steroidal anti-inflammatory agents should also be avoided for the same time period unless medically necessary.

Granulocyte-macrophage colony-stimulating factor (GM-CSF) should be avoided due to potential to worsen cerebrospinal fluid (CSF) symptoms. Granulocyte-colony stimulating factor (G-CSF) would be the preferred myeloid growth factor over GM-CSF, if medically indicated. The effects of G-CSF on neoTCR-P1 are unknown. Unless medically indicated, short acting G-CSF should not be given within 72 hours of neoTCR-P1 infusion and long-acting G-CSF should not be given within 10 days of NeoTCR-P1.

Treatment *(including investigational agents)* for solid tumors such as chemotherapy, immunotherapy, hormonal therapy, targeted therapy, radiation, and high-dose corticosteroids, other than defined/allowed in this protocol is not permitted *during the 2-year Treatment Follow-up Period. Participants with disease progression who require new treatment for their tumors should enter the Long-Term Follow-Up (Section 8.5.9) and will continue to be followed for survival and subsequent anti-cancer therapy.*

After Day 28, certain forms of radiotherapy may be considered for pain palliation if participants are deriving benefit. Participants experiencing a mixed response requiring local therapy (e.g., surgery, stereotactic radiosurgery, radiofrequency ablation) for the control of 3 or fewer lesions may still be eligible to continue study treatment (i.e., with nivolumab). Such cases must be discussed with and approved by the Medical Monitor.

With the exception of those who will receive IL-2 in the designated Phase 1a basket expansion cohort, participants are not allowed to receive immunostimulatory agents, including but not limited to interferon (IFN)-α, IFN-γ, or IL-2 during the entire study.

If permissibility of a specific medication/treatment is in question, please contact the Medical Monitor.

Participants in the Phase 1b receiving nivolumab who experience infusion-associated symptoms may be treated symptomatically with acetaminophen, ibuprofen, diphenhydramine, and/or other H2 receptor antagonist as per standard practice.

6.7. Dose Modification

The treating physician may use discretion in modifying or accelerating the guidelines described below depending on the severity of toxicity and an assessment of the risk versus benefit for the participant, with the goal of maximizing compliance and access to supportive care.

6.7.1. NeoTCR-P1

There will be no dose modification for neoTCR-P1 in this study.

6.7.2. Nivolumab

There will be no dose modifications for nivolumab in this study.

Participants may temporarily suspend study treatment with nivolumab for up to 56 days beyond the next scheduled dose if they experience an adverse event that requires a dose to be withheld. If nivolumab is withheld because of adverse event for more than 56 days beyond the last dose, then the participant will remain in the study, but nivolumab treatment will be discontinued. Exceptions require Medical Monitor approval. Participants will continue to be followed for safety and efficacy per the SOA.

Dose interruptions for reason(s) other than adverse events, such as surgical procedures, may be allowed with Medical Monitor approval. The acceptable length of interruption will depend upon agreement between the investigator and the Medical Monitor.

Nivolumab administration should be interrupted and increased monitoring of participants should ensue if any of the following drug related adverse event(s) occurs:

- Any persistent Grade ≥2 nonlaboratory nonskin drug related adverse event except for Grade 2 fatigue and weakness
- Any Grade ≥2 endocrine drug-related adverse event

- Any Grade ≥3 skin drug related adverse event
- Any Grade ≥3 drug-related laboratory abnormality (except lymphopenia, asymptomatic lipase or amylase increase, or any electrolyte abnormality without any clinical sequelae that is either spontaneously reversible or resolves with clinical management to Grade 2 or less within 72 hours
- ALT or AST >3 × ULN (treatment may resume when the AE resolves to Grade ≤1)
- Any adverse event, laboratory abnormality, or intercurrent illness that, in the judgment of the investigator, warrants skipping the dose of study medication

Nivolumab drug administration must be discontinued if at least 1 of the following drug-related adverse event(s) occurs:

- Any Grade ≥2 eye pain or reduction of visual acuity that does not respond to topical therapy and does not improve to Grade 1 severity within 2 weeks of starting therapy or requires systemic treatment
- Any Grade ≥3 non-laboratory drug-related adverse event with the exception of fatigue, and endocrine drug related AEs which are stable with hormone replacement therapy
- Any Grade ≥3 bronchospasm, hypersensitivity reaction, or infusion reaction
- Any participant that experiences any grade of allergic reaction while receiving nivolumab at a slower infusion rate and/or with premedication due to a prior allergic reaction
- Any drug-related Grade 4 laboratory abnormalities (except lymphopenia, asymptomatic lipase/amylase increases or any electrolyte abnormality without any clinical sequelae that is either spontaneously reversible or resolves with clinical management to Grade ≤2 within 72 hours
- Hepatotoxicity as evidenced by any of the following
  - AST or ALT >5–10 × ULN for >2 weeks
  - AST or ALT >10 × ULN
  - Total bilirubin >5 × ULN
  - Concurrent AST or ALT >3 × ULN and total bilirubin >2 × ULN
- Any adverse event, laboratory abnormality, or intercurrent illness that, in the judgment of the investigator, presents a substantial clinical risk to the participants.

Refer to the Nivolumab USPI for more detailed information regarding drug administration.

6.7.3. IL-2

Doses will be skipped for Grade 3 or 4 toxicity due to IL-2 except for Grade 3 toxicities common to IL-2 that are reversible within 12 hours, such as diarrhea, nausea, vomiting, medically manageable hypotension, skin changes, anorexia, mucositis, dysphagia, or constitutional symptoms and laboratory changes. The simultaneous occurrence of several toxicities may require IL-2 to be held. If a participant is not able to tolerate 4 consecutive doses of IL-2, further IL-2 will not be given. A maximum of 7 doses (out of a maximum of 14) can be skipped before IL-2 must be discontinued.

IL-2 will be held per the parameters below:

- Hypotension <80 systolic or hypertension >160 systolic
- Sinus tachycardia >120, sinus bradycardia <60, or any other arrythmias
- Syncope
- Chest pain
- Seizures
- Respirations of <10 or >30 per minute, or O_2 saturation <90% room air
- Urine output of less than 100 mL q4h
- Change in mental status
- Temperature more than 103°C, unresponsive to medication

IL-2 will be permanently discontinued for the following Grade ≥3 toxicities:

- Renal failure
- Somnolence requiring intubation
- Bowel perforation
- Myocardial infarction

Consensus best management practices for the administration of high dose IL-2 are reproduced in Table 18 (from Dutcher et al., 2014).

Table 18 Clinical Management Recommendations for High Dose IL-2 Therapy

Issue	***Considerations***	***Management***
Venous access	*Central line (for possible vasopressors)*<br>*Double or triple lumen*<br>*Power inject and large volume capacity*<br>*Minimize catheter-associated infection*	***Typical***<br>*PICC line placement*<br>*Remove temporary lines at end of cycle*<br>***Variations***<br>*Broviac/Hickman catheter*<br>*Subclavian/IJ catheter*
IV fluids	*Maintenance of volume with CLS*<br>*Boluses for BP support*<br>*Administration of drugs*<br>*Replacement of electrolytes*<br>*IL-2 only compatible with D5W*	***Typical***<br>*D5NS or D5LR 10 ml – 125 ml/hr*<br>*KCL, HCO_3, Mg replacement*<br>***Variations***<br>*D5W, NS, 0.45% NaCl*
Infections	*No active infections*<br>*Prevention*<br>*IV catheter likeliest source*<br>*Avoid unnecessary in-dwelling catheters*	***Typical***<br>*Gram + prophylactic antibiotic*<br>***Variations***<br>*Expanded coverage per hospital*

Issue	***Considerations***	***Management***
Chills/rigors<br><br>***Fever***<br><br>***Constitutional symptoms***	*Chills and rigors occur 1-2 hrs after IL-2*<br><br>*Fever is common 2-4 hrs after IL-2*<br><br>*Muscle & joint aches continuous and progressive during IL-2 treatment*	***Fever – Typical***<br>*Prophylaxis*<br>*Acetaminophen 650 mg 30 mins pre-dose, q 4-6 hrs and as needed*<br>*Indomethacin 25 mg q 6-8 hrs*<br>***Fever – Variation***<br>*Naproxen*<br>*Ibuprofen*<br>***Chills – Typical***<br>*Meperidine 25 mg IV q 15 mins prn*<br>*Morphine 2-4 mg IV q 15 mins prn*
Nausea/ vomiting	*Episodic occurrence throughout therapy*<br>*Nausea > vomiting*	***Typical – Prophylaxis***<br>*Ondansetron 0.15 mg/kg q 8 hrs*<br>***Variations***<br>*Granisetron 1 mg daily*<br>*Ondansetron at longer interval*<br>*Compazine 10 mg po q 6 hrs*<br>*Use of antinausea agents as needed*
Epigastric distress	*Gastritis induced by stress, medications*	***Typical***<br>*H2 blocker prophylaxis*<br>***Variation***<br>*PPI prophylaxis*
Mucositis/ stomatitis	*Progressive with continued treatment*	***Typical***<br>*No prophylaxis*<br>*Oncology mouthwash*
Diarrhea	*Can be profuse and increases* with therapy<br>*5HT-3 antagonist anti-emetic prophylaxis may have positive impact*	***Typical***<br>*Imodium*<br>*Lomotil*<br>*Narcotic*<br>*Break between IL-2 doses*
Patient monitoring	*Input & output, weight*<br>*BP, pulse, respirations, temp*<br>*Blood work*<br>*EKG*<br>*O2 saturation*<br>*Mental status examination*	*Per shift and daily*<br>*Q 2-4 hrs*<br>*Daily*<br>*Continuous cardiac monitoring*<br>*Q 2-4 hrs*<br>*Q 8 hrs*<br>***Increase frequency as needed***
Aldesleukin/ Interleukin-2 dose and administration	*IL-2 incompatible* with salt solutions<br>*Dissolve in sterile water for injection*<br>*Dilute into 50 cc D5W*<br>*Stop infusion, flush IV tubing with 50 cc D5W before and after each dose*	***Typical****: 600,000 IU/kg over 15 mins q 8 hrs up to 14 doses*<br>***Variations****: 720,000 IU/kg q 12 hrs*<br>*<14 maximum doses*
Hypotension	*Maintain systolic BP 80-90 mmHg*<br>*BP nadirs 4-6 hrs after each dose with diminished recovery with cumulative dosing*	*Fluid boluses, 250-500 ml NS 2xday*<br>*Increase maintenance fluid rate*<br>*Phenylephrine 0.1-4.0 mcg/kg/min*<br>*Hold next dose*

Issue	*Considerations*	*Management*
	Prior to each dose anticipate ability to respond to next nadir<br>*Progressive refractoriness to support measures*	*Discontinue IL-2*<br>***Variations:***<br>*Dopamine 1-6 µg/kg/min*<br>*Pressors with minimal fluids*<br>*Fluids without pressors*
Cardiac arrhythmias	***Sinus tachycardia***<br>*Common and progresses over a cycle*<br>*Peaks 2-4 hrs after dose with fever and hypotension*<br>*Must resolve prior to next dose*<br>***Supraventricular tachycardia***<br>*Less common*<br>***Atrial fibrillation***<br>***Ventricular tachycardia***	*Manage BP and fever*<br><br><br><br><br>*Medical Conversion*<br>*Cardizem as needed*<br>*Digoxin*<br>*Medical Conversion*<br>*Acute treatment*<br>*Discontinue IL-2*
Renal function	 ***Oligourea***<br><br><br>***Rising creatinine***<br><br><br>*Urine output and creatinine resolve after discontinuation of IL-2*<br>*If only one kidney always consider obstruction of ureter*	***Typical***<br>*Output less than 50-100 cc/8 hrs Fluid bolus, if no improvement next shift hold IL-2 dose*<br>*Creatinine >3-4*<br>*Stop NSAIDS and nephrotoxic antibiotics*<br>*Hold overnight dose*<br>*If AM improved continue*<br>***Variations***<br>*Dopamine 1-6 µg/kg/min*<br>*Furosemide*
Pulmonary	*Tachypnea/Dyspnea*<br>*Diagnose etiology and treat*<br><br>*Hypoxic causes – Fluid overload. capillary leak, bronchospasm*<br>*Non-hypoxic causes*<br>*Anxiety, fever, acidosis*<br><br>***Maintain O_2 saturation >92%-95%***	***Typical***<br>*Oxygen 2-4 L nasal cannula, increasing up to 35% rebreather*<br>*Reassurance or sedative for anxiety, treat bronchospasm or acidosis if appropriate*<br>*Hold IL-2 dose if O_2 saturation <95%*<br><br>***Variation***<br>*Furosemide*<br>*Bronchodilators*<br>*Monitor bicarbonate*
Peripheral edema	*Expect to gain 5%-10% body weight*<br><br>*Treat edema symptomatically*<br><br><br>*Entrapment of peripheral nerves in upper extremity may need therapy*	*Elevation, compression, limit fluid support in subsequent cycles*<br>*Diuretics upon conclusion of IL-2 dosing are not necessary buy may speed process*<br>*Treat peripheral nerve pain*

Issue	Considerations	Management
**Neurotoxicity**	 Protean manifestations<br>Gradual onset with sudden worsening near end of cycle<br>May persist after cessation of therapy<br>Delusions, Visual hallucinations	**Typical**<br>Formal neuro checks<br>Enlist family evaluation<br>Lorazepam and Haloperidol<br>Hold IL-2 liberally for suspected neurotoxicity<br>Warn patient of vivid dreams after discharge
**Dermatologic**	 Rash, erythema, dry desquamation<br>Pruritis<br>Moist dermatitis	**Typical**<br>Emollient lotions and creams Oatmeal bath<br>Antihistamines<br>Hold IL-2 dose<br>**Variations**<br>Crisco<br>Gabapentin<br>Naloxone<br>Narcotics<br>Nonalcohol, no steroid topicals
**Metabolic**	Hypomagnesemia, hypocalcemia (but low albumin – so corrected may be WNL), Hypokalemia<br>Acidosis due to diarrhea, hypoperfusion<br>Hypothyroidism a slow onset problem	Daily electrolyte panels<br>Correct electrolytes cautiously prn<br>Magnesium and HCO_3, particularly if diarrhea a problem<br>HCO_3 <18 meq/L hold dose of IL-2<br>Check TSH at beginning of cycle<br>RL as support fluids may decrease need for HCO_3
**Hepatic**	↑Bilirubin(up to 10)<br>↓Albumin (down to 1.8)<br>↑Hepatic aminotransferases	Monitor daily<br>No intervention except if SGOT/SGPT are >5x<br>Resolves spontaneously<br>Stop acetaminophen if bilirubin >5
**Hematologic**	↓Platelets<br>Lymphs ↓ during IL-2, ↑ post therapy<br>Eosinophils progressively ↑ with several cycles	Transfuse if platelets < 20 K<br>Other abnormalities require no intervention<br>Significant anemia needs evaluation for cause
**Endocrine**	Hypothyroidism – slow onset after completion of treatment<br>Requires serial monitoring	Check TFTs at beginning of cycle and monitor TFTs with subsequent visits

Source: Dutcher et al., 2014.

BP = blood pressure; hrs = hours; IV = intravenously; Lymphs = lymphocytes; mins = minutes; NS = normal saline; NSAIDS = non-steroidal anti-inflammatory drugs; PPI = proton pump inhibitor; Q/q = every; prn = as needed; RL = Ringer's lactate; SGOT = serum glutamic-oxaloacetic

transaminase; SGPT = serum glutamate-pyruvate transaminase; TFT = thyroid function test; TSH = thyroid stimulating hormone; WNL = within normal limits.

6.7.4. Rescue Medications

Rescue medications are medications given for severe CRS or neurotoxicity due to NeoTCR-P1 or the combination of NeoTCR-P1 and nivolumab. NeoTCR-P1 administration may require tocilizumab, steroids, and siltuximab for the treatment of suspected CRS toxicities as described below in Section 6.11.3. All rescue medications must be listed as concomitant medication.

6.7.5. Retreatment Criteria

Under circumstances where participants initially respond (i.e., CR, PR, or prolonged stable disease [SD] >6 months) and subsequently relapse or progress, participants may be eligible for a second course of NeoTCR-P1 with or without conditioning chemotherapy if they meet the following criteria:

- Disease progression must occur greater than 3 months after NeoTCR-P1 infusion.
- Continues to meet the original study eligibility criteria with the exception of prior NeoTCR-P1 use in the study.
- Participant has not received subsequent therapy for the treatment of their underlying malignancy with the exception of local therapy of a brain metastasis.
- Participant did not experience a DLT in the dose-escalation component or a comparable toxicity in the expansion cohorts.
- Toxicities related to conditioning chemotherapy (i.e., fludarabine and cyclophosphamide), with the exception of alopecia, have resolved to Grade ≤1 or returned to baseline prior to re-treatment.
- If medically feasible, participants should submit a tumor biopsy prior to retreatment.

6.8. Subsequent Therapy

Subsequent therapy administered after the NeoTCR-P1 infusion for a participant’s disease, such as non-study specified chemotherapy, immunotherapy, targeted agents, and radiation therapy, will be recorded until the participant completes the LTFU, is considered lost to follow up, withdraws consent, or dies.

6.9. Intervention After the End of the Study

There is no intervention following the end of the study.

6.10. Study Treatment Schedule

6.10.1. Leukapheresis

Participants who meet the eligibility criteria will undergo leukapheresis to obtain leukocytes for the manufacturing of NeoTCR-P1 within approximately 5 days of eligibility confirmation.

The leukapheresed cells are then packaged for expedited shipment to the CPF.

Manufacturing is initially expected to take up to approximately 4-6 weeks with a goal to decrease manufacturing turnaround time to approximately 3 weeks. Manufacturing times may vary for individual participants. See Section 8.5.3 regarding additional details related to leukapheresis.

6.10.2. Study Treatment

6.10.2.1. Conditioning Chemotherapy

Participants will initiate a 4-day conditioning cycle with cyclophosphamide and fludarabine within approximately 6 days prior to the NeoTCR-P1 infusion (i.e., Day -6, -5, Day -4, and Day -3). The 4-day conditioning regimen may be administered as an outpatient regimen by a participant's local oncologist within the specified time frame depending upon the need for intravenous fluids. See Section 8.5.5 for additional details.

6.10.2.2. NeoTCR-P1 Infusion

Following completion of each participant's conditioning chemotherapy regimen, all participants will be hospitalized to receive NeoTCR-P1 followed by an observation period of a minimum of approximately 7 days. Participants will remain in the hospital until pre-specified criteria are met (Section 8.5.7).

6.11. Toxicity Management

6.11.1. Acute Infusion Reaction

Acetaminophen/paracetamol and diphenhydramine/H1 antihistamine may be repeated every 6 hours as needed. A course of non-steroidal anti-inflammatory medication may be prescribed if the participant continues to have fever not relieved by acetaminophen/paracetamol. It is recommended that participants not receive corticosteroid at any time, except those already on physiologic replacement therapy, or in the case of a life-threatening emergency since this may have an adverse effect on NeoTCR-P1 T cells.

6.11.2. Febrile Reaction

In the event that a participant develops fever, with or without chills or rigors, an evaluation for infection should be initiated and participants managed appropriately with antibiotics, fluids, and other supportive care as medically indicated as determined by the treating physician or institutional protocols. In the unlikely event that the participant develops sepsis or systemic bacteremia following NeoTCR-P1 T-cell infusion, appropriate cultures and medical management should be initiated. If a contaminated NeoTCR-P1 T-cell product is suspected (see Section 8.6 regarding further details about reporting requirements), the product can be retested for sterility using an archived sample that is stored in the CPF. Consideration of CRS (see Section 6.11.3) should be given.

6.11.3. Cytokine Release Syndrome

Cytokine-associated toxicity, also known as CRS, is a non–antigen-specific toxicity that occurs as a result of high-level immune activation. CRS clinically manifests when large numbers of lymphocytes and/or myeloid cells become activated and release cytokines, which induce a wide

variety of cardiac, gastrointestinal, laboratory (e.g., renal, hepatic, coagulation), respiratory, skin, vascular and constitutional signs and symptoms (Shimabukuro-Vornhagen et al., 2018).

Table 19 Clinical Signs and Symptoms Associated with Cytokine Release Syndrome

Organ System	Signs and Symptoms
Constitutional	Fever ± rigors, malaise, fatigue, anorexia, myalgias, arthralgias, headache
Skin	Rash
Gastrointestinal	Nausea, vomiting, diarrhea
Respiratory	Tachypnea, hypoxemia
Cardiovascular	Tachycardia, widened pulse pressure, hypotension, increased cardiac output (early), potentially decreased cardiac output (late)
Coagulation	Elevated D-dimer, hypofibrinogenemia ± bleeding
Renal	Azotemia
Hepatic	Elevated LFTs, hyperbilirubinemia
Neurologic	Headache, mental status changes, confusion, delirium, word finding difficulty, aphasia, hallucinations, tremor, dysmetria, altered gait, seizures

LFT = liver function test.

Table 20 CRS Reported in Recent Clinical Trials of CD19 CAR-T

Reference	Maude et al., 2018	Park et al., 2018	Neelapu et al., 2017	Schuster et al., 2017	Turtle et al., 2017	Gardner et al., 2017	Lee et al., 2015	Maude et al., 2015
No. of participants	75	53	101	28	24	45	20	30
% sCRS	46	26	13	18	8	23	32	27
Treatment-related deaths	1	1	3	1	1	0	0	0

CAR = chimeric antigen receptor; CRS = cytokine release syndrome; sCRS = severe CRS.

The timing of symptom onset and severity depends on the inducing agent and the magnitude of immune cell activation. Symptom onset usually occurs with the first week and typically peaks within 1–2 weeks after the CAR-T-cell administration. Participants at high risk of severe CRS include those with bulky disease, comorbidities and those who develop early onset CRS within 3 days of cell infusion (Neelapu et al., 2017). The incidence, severity onset and peak of potential CRS events for gene-edited TCR-T cells is less well described.

The goal of CRS management is to prevent life-threatening conditions while preserving anti-tumor effects. Multiple CRS grading systems have been created (e.g., Lee, Penn, MSKCC, CARTOX) (Lee et al., 2018) that build on the traditional NCI CTC AE criteria and were recently harmonized by the ASTCT in a set of Consensus Grading Criteria that will (see Table 21) will be used to assess CRS in this study.

Table 21 Cytokine Release Syndrome Consensus Grading Scale (ASTCT)

CRS Parameter	Grade 1	Grade 2	Grade 3	Grade 4
Fever [a] with	Temperature ≥38°C	Temperature ≥38°C	Temperature ≥38°C	Temperature ≥38°C
Hypotension and/or [b]	None	Not requiring vasopressors	Requiring a vasopressor with or without vasopressin	Requiring multiple vasopressors (excluding vasopressin)
Hypoxia	None	Requiring low-flow nasal cannula [c] or blow-by	Requiring high-flow nasal cannula, [c] facemask, nonrebreather mask, or Venturi mask	Requiring positive pressure (e.g., CPAP, BiPAP, intubation, and mechanical ventilation)

BiPAP = bilevel positive airway pressure; CPAP = continuous positive airway pressure; CRS = cytokine release syndrome; NCI CTCAE v5.0 = National Cancer Institute Common Terminology Criteria for Adverse Events version 5.0.

Note: Organ Toxicities associated with CRS may be graded according to NCI CTCAE v5.0, but they do not influence CRS grading.

a Fever is defined as temperature ≥38°C not attributable to any other cause. In participants who have CRS then receive antipyretics or anti-cytokine therapy such as tocilizumab or steroids, fever is no longer required to grade subsequent CRS severity. In this case, CRS grading is driven by hypotension and/or hypoxia.

b CRS grades is determined by the more severe event: hypotension or hypoxia not attributable to any other cause. For example, a participant with a temperature of 39.5°C, hypotension requiring 1 vasopressor, and hypoxia requiring low-flow nasal canula is classified as Grade 3 CRS.

c Low-flow nasal cannula is defined as oxygen delivered at ≤6 L/minute. Low flow also includes blow-by oxygen delivery, sometimes used in pediatrics. High-flow nasal cannula is defined as oxygen delivers at >6 L/minute.

Source: Lee et al., 2018.

The management guideline in Table 22 is built upon the work of the CARTOX group at MDACC and uses the CRS grading system outlined in Table 21 to direct the management of CRS potentially associated with treatment with NeoTCR-P1 (Neelapu et al., 2017). Modifications to the CARTOX CRS management guidelines are based upon recommendations from Teachey et al. (2018). Sites may also consult institution-specific guidelines for the management of CRS.

Table 22 Cytokine Release Syndrome Management Guidelines

CRS Grade	Symptom or sign	Treatment
**Grade 1**	Fever or organ toxicity	• Acetaminophen and hypothermia blanket for the treatment of fever<br>• Ibuprofen can be used as second treatment option for fever, if not contraindicated<br>• Assess for infection using blood and urine cultures, and chest radiography<br>• Empiric broad-spectrum antibiotics and filgrastim if neutropenia<br>• Maintenance IV fluids for hydration<br>• Symptomatic management of constitutional symptoms and organ toxicities<br>• Consider tocilizumab 8 mg/kg [a] IV for persistent (lasting >3 days) and refractory fever
**Grade 2**	Hypotension	• IV fluid bolus of 500–1000 mL of normal saline<br>• Can give a second IV fluid bolus if systolic blood pressure remains <90 mmHg<br>• Administer tocilizumab 8 mg/kg [a] IV for treatment of hypotension that is refractory to fluid boluses<br>• Tocilizumab can be repeated after 6 hours, if needed<br>• If hypotension persists after 2 fluid boluses and anti-IL-6 therapy, start vasopressors, consider transfer to ICU, obtain echocardiogram, and initiate other methods of hemodynamic monitoring<br>• In participants at high-risk [b] or if hypotension persists after 1–2 doses of anti-IL-6 therapy, methylprednisolone can be used at 2 mg/kg (initial dose) followed by 1–2 mg/kg per day
	Hypoxia	• Supplemental oxygen<br>• Tocilizumab or siltuximab ± corticosteroids and supportive care, as recommended for the management of hypotension
	Organ toxicity	• Symptomatic management of organ toxicities, as per standard guidelines<br>• Tocilizumab or siltuximab ± corticosteroids and supportive care, as indicated for hypotension
**Grade 3**	Hypotension	• IV fluid boluses as needed, as recommended for the treatment of Grade 2 CRS<br>• Tocilizumab as recommended for Grade 2 CRS, if not administered previously<br>• Vasopressors as needed<br>• Transfer to ICU, obtain echocardiogram, and perform hemodynamic monitoring as in the management of Grade 2 CRS<br>• Dexamethasone 10 mg IV every 6 hours<br>• If refractory, increase to 20 mg IV every 6 hours<br>• Consider siltuximab 11 mg/kg IV as third-line treatment for CRS after failure of both tocilizumab and corticosteroids<br>• Manage fever and constitutional symptoms as indicated for Grade 1 CRS
	Hypoxia	• Supplemental oxygen including high-flow oxygen delivery and non-invasive positive pressure ventilation<br>• Tocilizumab plus corticosteroid and siltuximab and supportive care, as described above
	Organ toxicity	• Symptomatic management of organ toxicities as per standard guidelines<br>• Tocilizumab plus corticosteroid and siltuximab and supportive care, as described above

CRS Grade	Symptom or sign	Treatment
**Grade 4**	Hypotension	• IV fluids, anti-IL-6 therapy, vasopressors, and hemodynamic monitoring as defined for the management of Grade 3 CRS<br>• Methylprednisolone 1 g/day IV<br>• Administer siltuximab 11 mg/kg [c] IV as third-line treatment for CRS after failure of both tocilizumab and corticosteroids. If ongoing CRS despite prior therapy, consider anti-T-cell therapies<br>• Manage fever and constitutional symptoms as in Grade 1 CRS
	Hypoxia	• Mechanical ventilation<br>• Tocilizumab plus corticosteroid, siltuximab, and supportive care, as described above
	Organ toxicity	• Symptomatic management of organ toxicities as per standard guidelines<br>• Tocilizumab plus corticosteroid, siltuximab, and supportive care, as described above

CAR = chimeric antigen receptor; CRS = cytokine release syndrome; ICU = intensive care unit; *IL-6 = interleukin 6;* IV = intravenous.

Note: All medication doses indicated are for adults.

[a] Maximum amount of tocilizumab per dose is 800 mg. Doses can be given 8 hours apart.

[b] High-risk participants include those with bulky disease, those with comorbidities, and those who develop early onset CRS within 3 days of CAR-T-cell infusion.

[c] No more than 1 dose of siltuximab in a 3-week period should be administered.

Actemra® (tocilizumab), a monoclonal antibody to the IL-6 receptor is approved for the treatment of CRS in adults and pediatric patients 2 years of age and older with CAR-T-cell induced severe or life threating CRS. Tocilizumab should be administered at a dose of 8 mg/kg infused intravenously over 1 hour (dose not to exceed 800 mg) and repeated every 4-6 hours, as needed, based on response in patients weighing >30 kg. Up to 3 doses may be administered in a 24-hour period (see USPI or SmPC for additional details).

Tocilizumab can be adminsitered per the table above or under the following additional circumstances, if a symptom is thought to be related to cytokine release:

- Left ventricular ejection fraction 40% or less by echocardiogram
- Creatinine greater than 3X higher than prior to NeoTCR-P1 infusion
- Norepinephrine requirement for 36 hours since the first administration of norepinephrine even if norepinephrine administration was not continuous

If there is no significant improvement with tocilizumab (e.g., decreased pressor or oxygen requirement), corticosteroids should be administered. The choice of steroid is at the discretion of the principal investigator and/or institutional guidelines (e.g., methylprednisolone 1 mg/kg BID or dexamethasone 10 mg every 6 hours). High doses of corticosteroids (e.g., methylprednisolone 1 g/day × 3 days, followed by a rapid taper, based on response, consisting of 250 mg BID × 2 days, then 125 mg BID × 2 days and then 60 mg BID × 2 days) should be considered for life-threatening CRS.

Sylvant® (siltuximab), a monoclonal antibody to IL-6 is approved for the management of multicentric Castleman disease and prevents IL-6 from interacting with both the membrane-bound and soluble form of the IL-6 receptor (Shimabukuro-Vornhagen et al., 2018).

Other immunosuppressive agents that target IL-6, tumor necrosis factor (TNF)-alpha, and IL-1 are also available. Hence anti-TNF-alpha antibody (infliximab) soluble TNF-alpha receptor (etanercept), and IL-1-receptor antagonist (anakinra) might also prove benefit and have been used to manage CRS (Frey et al., 2014; Lee et al., 2014; Chen et al., 2016).

These agents may be considered in cases where anti-IL-6 directed therapy and corticosteroids do not effectively control NeoTCR-P1-related cytokine-mediated toxicity in consultation with the Medical Monitor.

6.11.4. Hypotension and Renal Toxicity

Hypotension and renal insufficiency should be treated as described here or according to institutional guidelines. Vigorous IV fluid hydration may be needed to manage hypotension and vascular leak in the setting of CRS. Participants should be closely monitored to prevent fluid overload, and in some cases continuous veno-venous hemodialysis may be required. Invasive hemodynamic monitoring, for example with a pulmonary artery catheter, may be helpful to optimize fluid management in settings of concurrent severe capillary leak, aggressive IV fluid administration, and/or pulmonary edema. Antihypertensives should be withheld whenever blood pressure begins to decrease below baseline values.

The baseline blood pressure is defined for this guideline as the average of blood pressure readings obtained during the 24 hours prior to the NeoTCR-P1 infusion. The first treatment for hypotension is administration of IV fluid boluses. Management of hypotension should be per recommendations below and may be modified based on institutional guidelines as well as the characteristics of individual participants, such as pulmonary status, and cardiac function.

- Participants with a systolic blood pressure, diastolic blood pressure, or mean arterial pressure that is ≤80% of their baseline or less than the lower limit of normal should receive 1 liter of normal saline bolus.
- If the hypotension does not respond adequately to a fluid challenge within 1 hour, a second fluid bolus (volume per the investigator discretion) should be administered.
- If hypotension persists despite 2 fluid boluses, consideration should be given for monitoring in the intensive care unit (or similar setting) and administering vasopressor support.

6.11.5. Cardiac Toxicity

Cardiac manifestations of CRS may include arrhythmias, decreased ejection fraction/heart failure, myocardial ischemia, and cardiac arrest. Tachycardia is common in the setting of CRS and medications to slow sinus tachycardia should be avoided. Hypotension should be managed as described in Section 6.11.4. Participants with persistent hypotension not responsive to fluids, delayed response to vasopressors and/or severe fluid overload should be evaluated for decreased ejection fraction/heart failure by echocardiogram. These toxicities should be emergently managed per medical judgment and institutional practice guidelines.

Participants with Grade ≥2 cardiac toxicity should be monitored with continuous cardiac telemetry. Anti–IL-6 directed therapy and corticosteroids should be administered per institution-specific guidelines or per guidelines outlined in Section 6.11.3. Follow-up ECGs are recommended to monitor the course of toxicity to resolution.

6.11.6. Hemophagocytic Lymphohistiocytosis

HLH is an aggressive and potentially life-threatening clinical syndrome of excessive immune activation that results in cytokine release and multi-organ dysfunction (Jordan et al., 2011; La Rosée, 2015). Symptoms include fevers, cytopenias, hepatic dysfunction, hyperbilirubinemia, coagulopathy, hemophagocytosis with marked elevations in ferritin, C-reactive protein (CRP), and soluble interleuken-2 receptor (sCD25) (Porter et al., 2015). Neurologic findings have also been observed in approximately one-third of cases.

Severe HLH may be triggered by infections, treatment with immunotherapy, or neoplastic or autoimmune processes (Abe et al., 2002; Ferreria et al. 2006; Lackner et al., 2008; La Rosée, 2015). Rare cases of fulminant HLH after treatment with blinatumomab or anti-CD19 CAR-T cells have been reported (Teachey et al., 2013; Barrett et al., 2014; Maude et al., 2014; Maude et al., 2015; Porter et al., 2015). CRS and HLH may possess similar clinical syndromes with overlapping clinical features and pathophysiology. Cytokine production from activated T cells may lead to excessive macrophage activation and HLH.

HLH should be considered if there are unexplained elevated liver function tests or cytopenias with or without other evidence of CRS. Additional monitoring of CRP, ferritin, and sCD25 levels may assist with the diagnosis and define the clinical course. Bone marrow biopsy should be considered to evaluate for hemophagocytosis.

Diagnostic criteria for ACT-related HLH/MAS include a peak serum ferritin level of >10,000 ng/mL during the cytokine-release syndrome part of therapy plus subsequent development of any of the following:

- Grade ≥3 increase in serum bilirubin, AST, or ALT
- Grade ≥3 oliguria or increase in serum creatinine levels
- Grade ≥3 pulmonary edema
- Presence of hemophagocytosis in bone marrow or organs based on histopathological assessment of cell morphology and/or CD68 IHC

Given the overlap with CRS, participants should be managed per CRS Treatment Guidelines (Section 6.11.3). Suspected cases of HLH should be discussed with the Medical Monitor.

Participants with HLH who have deteriorating organ function (e.g., cardiovascular, pulmonary, renal, hepatic, or neurologic) should be treated immediately with HLH-specific treatment. Treatment should not be delayed while awaiting genetic or specialized immunologic testing.

Among participants who are acutely ill or deteriorating and do not respond to anti-IL-6 therapy and corticosteroids with 48 hours, additional therapy with etoposide 75–100 mg/m^2 should be

considered. Etoposide can be repeated after 4–7 days, as indicated clinically or serologically, to achieve adequate disease control.

If the participant is not severely ill (e.g., does not have deteriorating cardiovascular, pulmonary, renal, hepatic, or neurologic function), it may be possible to treat the triggering condition with the addition of corticosteroids, and to observe the participant for a response prior to initiating chemotherapy. For those who show clinical improvement upon treatment of the triggering condition, it may be possible to avoid chemotherapy, although this is the rare exception rather than the rule.

Other agents for HLH are also available. Emapalumab-Lzsg is a fully human monoclonal antibody that it is a potent inhibitor of IFN-γ and was recently approved by the FDA for the treatment of pediatric and adult patients with refractory, recurrent, or progressive disease, or intolerance to conventional HLH therapy.

6.11.7. Neurotoxicity

Neurotoxicity has been observed in participants receiving CAR-T-cell therapies and can range from headache, confusion, altered level of consciousness, word-finding difficulties, dysarthria, encephalopathy, and rarely to seizure (Neelapu et al., 2017). Fatal neurological events have occurred with some CAR-T-cell products (Gilbert, 2017). This syndrome has also been termed ICANS or CAR-T-cell related encephalopathy syndrome. Neurotoxicity typically manifests as encephalopathy with the earliest symptoms being diminished attention, impaired handwriting, and language disturbance. The onset may be biphasic with the first phase occurring concurrently with CRS symptoms within the first 5 days and the second phase occurring after the fevers and CRS symptoms subside. Delayed neurotoxicity has been noted to occur in the third or fourth week after CAR-T infusion and can present with seizures or episodes of confusion. The incidence, severity, and kinetics are not well characterized for engineered TCR-T-cell products.

The goal of neurotoxicity management is to prevent life-threatening conditions while preserving antitumor effects. Multiple neurotoxicity grading systems have been developed (e.g., National Cancer Institute Common Terminology Criteria for Adverse Events [NCI CTCAE] v5.0, CARTOX) criteria and were recently harmonized by the ASTCT in a set of Consensus Grading Criteria that will (Table 23) will be used to assess neurotoxicity in this study (Lee et al., 2018).

Table 23 Neurotoxicity/ICANS Consensus Grading Scale (ASTCT)

Neurotoxicity Domain	Grade 1	Grade 2	Grade 3	Grade 4
ICE Score [a]	7–9	3–6	0–2	0 (participant is unarousable and unable to perform ICE)
Depressed level of consciousness [b]	Awakens spontaneously	Awakens to voice	Awakens only to tactile stimuli	Participant is unarousable or requires vigorous or repetitive tactile stimuli to arouse. Stupor or coma
Seizure	N/A	N/A	Any clinical seizure focal or generalized that resolves rapidly or nonconvulsive seizures on EEG that resolve with intervention	Life-threatening prolonged seizure (>5 min)<br>or repetitive clinical or electrical seizures without return to baseline in between
Motor findings [c]	N/A	N/A	N/A	Deep focal motor weakness such as hemiparesis or paraparesis
Elevated ICP/cerebral edema	N/A	N/A	Focal/local edema on neuroimaging [d]	Diffuse cerebral edema on neuroimaging<br>Decerebrate or decorticate posturing<br>or Cranial nerve VI palsy<br>or Papilledema<br>or Cushing's triad

ASTCT = Cytokine Release Syndrome Consensus Grading Scale; EEG = electroencephalogram; ICANS = immune effector cell-associated neurotoxicity; ICE = immune effector cell associated encephalopathy; ICP = intracranial pressure; *NCI CTCAE v5.0 = National Cancer Institute Common Terminology Criteria for Adverse Events version 5.0.*

Neurotoxicity/ICANS Grade is determined by the most severe event (ICE score, level of consciousness, seizure, motor findings, raised ICP/cerebral edema) not attributable to any other cause; for example, a participant with an ICE score of 3 who has a generalized seizure is classified as Grade 3 ICANS; N/A = not applicable.

a A participant with an ICE score of 0 may be classified as Grade 3 ICANS/neurotoxicity if awake with global aphasia, but a participant with an ICE score of 0 may be classified as Grade 4 if unarousable.

b Depressed level of consciousness should be attributable to no other cause (e.g., no sedating medication).

c Tremors and myoclonus associated with immune effector cell therapies may be Graded according to NCI CTCAE v5.0, but they do not influence ICANS/neurotoxicity grading.

d Intracranial hemorrhage with or without associated edema is not considered a neurotoxicity feature and is excluded from ICANS grading. It may be graded according to NCI CTCAE v5.0.

The Immune Effector Cell-Associated Encephalopathy (ICE) score has been developed as standardized screening tool to assess some of the components of neurotoxicity related to cellular therapies and includes elements to measure receptive aphasia and handwriting. ICE is a key component of neurotoxicity grading.

Table 24 Encephalopathy Assessment Tool for Grading Neurotoxicity/ICANS

ICE	Scoring
Add each of the points below to derive the ICE score:<br>1. Orientation: orientation to year, month, city, hospital. **4 points**<br>2. Naming: ability to name 3 objects (e.g., point to clock, pen, button). **3 points**<br>3. Follow commands: ability to follow simple commands (e.g., "show me 2 fingers" or "Close your eyes and stick out your tongue"). **1 point**<br>4. Writing: ability to write a standard sentence (e.g., "Our national bird is the bald eagle"). **1 point**<br>5. Attention: ability to count backwards from 100 by 10. **1 point**	10, no impairment<br>7–9, Grade 1 ICANS<br>3–6, Grade 2 ICANS<br>0–2, Grade 3 ICANS<br>0 due to participant unarousable and unable to perform ICE assessment, Grade 4 ICANS

ICE = Immune Effector Cell Associated Encephalopathy; ICANS = immune effector cell-associated neurotoxicity.

Evaluation of any new onset Grade 2 or greater neurotoxicity should include a neurological examination (including ICE), brain MRI, electroencephalogram (EEG) and examination of CSF. In addition, participants with Grade ≥3 neurotoxicity should be monitored with continuous cardiac telemetry and pulse oximetry as clinically indicated. Endotracheal intubation may be needed for airway protection in severe cases. Corticosteroids may be considered for any severe or life-threatening neurotoxicity and anti-seizure and sedatives may be considered as clinically indicated.

In some cases, multiple anti-epileptic medications may be needed to control seizures. Medications with sedative properties should be avoided if possible unless required to manage seizures.

Leukoencephalopathy has been observed on MRI in the setting of neurotoxicity. Participants should be managed based on clinical symptoms and not imaging alone. Follow up MRI is recommended to monitor the course of leukoencephalopathy to potential resolution.

Late onset of neurotoxicity (within approximately a month of discharge from the hospital) has been observed in patients receiving CAR-T therapies. Participants and their families/caregivers should be warned of the risk at hospital discharge and told to seek immediate medical attention for any new signs of neurotoxicity. Participants who develop neurotoxicity should be advised not to drive or operate heavy machinery for 1 month after complete resolution of neurotoxicity symptoms.

The management guideline in Table 25 is built upon the work of the CARTOX group and uses the ICANS grading system outlined in Table 23 to direct the management of neurotoxicity potentially associated with treatment with NeoTCR-P1 (Neelapu et al., 2017). Modifications to the CARTOX management guidelines are based upon recommendations from Teachey et al. (2018).

Table 25 Neurotoxicity Management Guidelines

Neurotoxicity Grading	Treatment	Evaluation
**Grade 1**	• Vigilant supportive care<br>• Aspiration precautions<br>• Intravenous hydration<br>• Withhold oral intake of food, medicines, and fluids, and assess swallowing<br>• Convert all oral medications and/or nutrition to IV if swallowing is impaired<br>• Avoid medications that cause central nervous system depression<br>• Low doses of lorazepam (0.25–0.5 mg IV every 8 hours) or haloperidol (0.5 mg IV every 6 hours) can be used, with careful monitoring in agitated patients<br>• Consider tocilizumab 8 mg/kg IV, if neurotoxicity is associated with concurrent CRS	• Frequent neurological examination and neurology consultation<br>• Additional work-up as clinical indicated<br>• Fundoscopic examination to assess for papilledema<br>• MRI of the brain with and without contrast<br>• Diagnostic lumbar puncture with measurement of opening pression<br>• MRI of the spine if that patient has focal peripheral neurological deficits<br>• CT scan of the brain can be performed if MRI of the brain is not feasible<br>• Daily 30-minute EEG until toxicity symptoms resolve<br>• If no seizures are detected on EEG, consider levetiractam 750 mg every 12 hours
**Grade 2**	• Supportive care and neurological work-up as described for Grade 1 neurotoxicity<br>• Tocilizumab 8 mg/kg IV if associated with concurrent CRS<br>• Dexamethasone 10 mg IV every 6 hours or methylprednisolone 1 mg/kg IV every 12 hours if refractory to anti-IL-6 therapy, or for neurotoxicity without concurrent CRS	• Consider transferring patient to intensive-care until if neurotoxicity is associated with Grade ≥2 CRS
**Grade 3**	• Supportive care and neurological work-up as described for Grade 1 neurotoxicity<br>• Tocilizumab 8 mg/kg IV if associated with concurrent CRS and not administered previously<br>• Corticosteroids as outlined for Grade 2 neurotoxicity if symptoms worsen despite tocilizumab or for neurotoxicity without concurrent CRS, continue corticosteroids until improvement to Grade 1 neurotoxicity and the taper<br>• Consider siltuximab 11 mg/kg IV as third-line treatment for neurotoxicity after failure of both tocilizumab and corticosteroids	• Transfer to ICU<br>• Consider repeat neuroimaging (CT or MRI) every 2–3 days if patient has persistent neurotoxicity

Neurotoxicity Grading	Treatment	Evaluation
**Grade 4**	• Supportive care and neurological work-up as described for Grade 1 neurotoxicity<br>• Anti-IL-6 therapy as described for Grade 3 neurotoxicity<br>• High-dose corticosteroids continued until improvements to Grade 1 neurotoxicity and then taper; for example, methylprednisolone IV 1 g/day for 3 days, followed by rapid taper at 250 mg every 12 hours for 2 days, 125 mg every 12 hours for 2 days, and 60 mg every 12 hours for 2 days<br>• For convulsive status epileptics, treat per institutional guidelines	• ICU monitoring: consider mechanical ventilation for airway protection<br>• Repeat neuroimaging

CRS = cytokine release syndrome; CT = computed tomography; EEG = electroencephalogram; ICU = intensive care unit; IL = interleukin; IV = intravenous; MRI = magnetic resonance imaging.

The following guidelines for the management of cerebral edema are adapted from Rabinstein et al (2006):

Table 26 Cerebral Edema Management Guidelines

Supportive Therapy	Tocilizumab	Corticosteroids	Follow-up
As above for neurologic events Grade 4, to include:<br>• Intensive care unit supportive therapy<br>• Optimal head position with elevation of head of bed and straight neck positioning<br>• Administration of diuretics and osmotherapy (e.g., mannitol, hypertonic saline)<br>• If cerebral edema documented or strongly suspected, recommend neurosurgical consult<br>• Early tracheal intubation with controlled mechanical mild hyperventilation and good oxygenation<br>• Maintain cerebral perfusion pressure with mild hypervolemia<br>• Avoid hypertension with use of anti-hypertensives (labetalol, nicardipine)<br>• Avoid potent vasodilators<br>• Pharmacological cerebral metabolic suppression (barbiturates, sedation, analgesia, and neuromuscular paralysis, as indicated)<br>• Maintain rigorous glycemic control	• Tocilizumab as above in Grade 4 neurologic event management (tocilizumab should be given only if concurrent CRS)	• High-dose corticosteroids: methylprednisolone 1000 mg/day	Improving:<br>• Very slow steroid taper recommended<br>• Repeat neuro-imaging as indicated<br>• Serial neurologic exams as indicated<br>• Consider early neuro-rehabilitation<br>• Not improving:<br>• Consider alternate immunosuppressants<br>• Consult medical monitor

CRS = cytokine release syndrome.

6.11.8. Tumor Lysis Syndrome

TLS is an oncologic emergency that is caused by massive tumor cell lysis with the release of large amounts of potassium, phosphate, and nucleic acids into the systemic circulation and has been described in patients with hematologic malignancies treated with CAR-T-cell therapy.

Laboratory TLS is defined as 2 or more of the following values within the days following NeoTCR-P1 infusion:

- Uric acid ≥8 mg/dL or 25% increase from baseline
- Potassium ≥6 mEq/L or 25% increase from baseline
- Phosphorous ≥4.5 mg/dL or 25% increase from baseline
- Calcium ≤7 mg/dL or 25% decrease from baseline

TLS has been rarely described after treatment of non-hematologic solid tumors and, therefore, patients with solid tumors are generally considered at low risk.

Prophylaxis should be considered in participants with bulky solid tumor who have renal dysfunction and/or renal involvement or uric acid, potassium, or phosphate levels above the ULN. Participants without contraindications may be started on prophylaxis (e.g., allopurinol) as per institutional guidelines prior to NeoTCR-P1 infusion. Prophylaxis should be discontinued when the risk of tumor lysis has passed.

If 0 or 1 of the laboratory values above are abnormal, continue to manage with allopurinol or a non-allopurinol alternative and hydration. Consider IV hydration and rasburicase if uric acid levels remain elevated and consider in hospital monitoring (if outpatient).

If laboratory TLS exists, manage with intravenous fluids, and monitor laboratory blood tests every 6–8 hours. Cardiac monitoring should be considered as well as rasburicase if uric acid levels are elevated.

If clinical TLS exists (Cairo and Bishop, 2004), manage with intravenous fluids, cardiac monitoring, frequent blood draws, rasburicase/allopurinol/febuxostat and inpatient care or per institutional guidelines.

6.11.9. Uncontrolled T-cell Proliferation

Insertional mutagenesis resulting in tumorigenesis of T cells is a potential toxicity related to gene-edited T-cell therapies. Participants should be treated with high dose steroids if there is evidence of uncontrolled T-cell proliferation related to transferred T cells. Chemotherapy and alemtuzumab (anti-CD52) may also be used to eradicate T cells.

6.11.10. Graft-Versus-Host Disease/Autoimmunity

In the event that endogenous TCR gene disruption is not comprehensive, the neoTCR alpha and beta chains expressed by the gene-edited T cells could undergo heterologous pairing with endogenous TCR alpha and beta chains. Such TCR chimeras hold the potential for newly

acquired specificities for peptide-HLA complexes expressed on the surface of cells other than tumor cells. These novel specificities would be unpredictable and since these gene-edited T cells are not subjected to thymic selection, there exists the potential for these T cells to possess autoreactive specificities resulting in autoimmunity. If evidence of autoimmunity develops, participants should receive immune suppressive therapies as clinically indicated based on the severity of symptoms, using medications like corticosteroids, cyclosporine-A, mycophenolate, mofetil, anti–TNF-α antibodies, or antithymocyte globulin. Investigators should refer to the American Society of Bone Marrow Transplant recommendations for the first- and second-line treatment of acute Graft-versus-Host Disease for further management (Martin et al., 2012).

All participants in the Phase 1b who receive nivolumab should be closely monitored for any signs of gastrointestinal, pulmonary, or endocrine system toxicity and aggressively managed at the first onset of clinical symptoms. A high index of suspicion for colitis, endocrinopathy (e.g., hypoparathyroidism, adrenal insufficiency or hypophysitis) or pneumonitis should be maintained in all participants who present with any symptom referable to these events, including, but not limited to diarrhea, abdominal symptoms, fatigue, lethargy, weakness, dehydration, or shortness of breath. Prompt initiation of appropriate diagnosis and therapy, including early administration of steroids, should be instituted following conversation with the Medical Monitor.

Investigators should refer to the Nivolumab USPI, American Society of Clinical Oncology Guidelines for the Management of Immune-related Adverse Events (Brahmer et al., 2018), or institution specific guidelines.

6.11.11. Capillary Leak Syndrome

Participants who receive IL-2 should be closely monitored for signs of capillary leak syndrome, including generalized edema, weight gain, pulmonary congestion, pleural effusion, and ascites (Schwartz et al., 2002; Schwartzentruber, 2001).

Investigators should refer to the USPI or institution specific guidelines for the safe administration of IL-2.

6.11.12. Fever and Neutropenia

Evaluation for a source of infection should be performed per institutional guidelines.

Fevers should be treated with acetaminophen and supportive measures. NSAIDs and corticosteroids should be avoided. Participants who are neutropenic and febrile should receive broad-spectrum antibiotics. Maintenance intravenous fluids per institutional guidelines should be administered to participants with high fevers to mitigate insensible loses.

6.11.13. Infection Prophylaxis

Participants should receive prophylaxis for infection with pneumocystis pneumonia, herpes virus, and fungal infections according to NCCN guidelines or institutional guidelines.

6.11.14. Blood Product Support

All blood products should be irradiated. Participants should receive platelets and packed red blood cells as needed to maintain hemoglobin >8.0 gm/dl and platelets >20,000/mm^3 or as per institutional guidelines. Leukocyte filters should be utilized for all blood and platelet transfusion to decrease sensitization to transfused leukocytes and decrease the risk of cytomegalovirus (CMV) infection.

7. Discontinuation of Study Intervention and Participant Discontinuation/Withdrawal

7.1. Discontinuation of Study Intervention

Once a participant has received NeoTCR-P1 they should be followed until the participant withdraws consent, dies, or is lost to follow-up. In gene therapy studies, participants should be followed for up to 15 years to evaluate specific long-term adverse events potentially-related to the study product.

Participants in this study will receive 1 of the following treatments:

- NeoTCR-P1 alone (a single dose of study treatment on Day 0),
- A single dose of NeoTCR-P1 followed by IL-2 on Days 1-7, or
- A single dose of NeoTCR-P1 in combination with nivolumab, with nivolumab administered every 4 weeks for up to 7 doses in total, *without or with IL-2.*

Participants will undergo scheduled study procedures (periodic safety and efficacy assessments and *pharmacokinetic/pharmacodynamic* (PK/PD) collection as described in the SOA) for 2 years from Day 0 (Post-Treatment Follow-Up).

Because participants receive only a single dose on Day 0, study treatment discontinuation criteria do not apply to NeoTCR-P1.

Where applicable, nivolumab and IL-2 study treatment should be discontinued for toxicity as described in Section 6.7.2 and Section 6.7.3.

In the absence of unequivocal disease progression, Post-Treatment Follow-Up study procedures per the Schedule of Assessments should be continued in all participants for 2 years, including those who experience a DLT, complete treatment (i.e., NeoTCR-P1 *without or with IL-2, or NeoTCR-P1 with 7 doses of nivolumab without or with* IL-2) or discontinue study treatment for intolerable toxicity.

Reasons for discontinuation from study treatment or Post-Treatment Follow-up include any of the following:

- Adverse event
- Withdrawal by participant

- Lost to follow-up
- Completed Post-Treatment Follow-up per protocol
- Unequivocal disease progression, unless eligible for retreatment (Section 8.5.11)
- Use of another systemic anti-cancer therapy
- Death
- Pregnancy
- Physician decision
- Sponsor decision

The reasons for discontinuation from study treatment or Post-Treatment Follow-Up must be recorded on the eCRF.

See the SOA (see Section 1.4) for data to be collected at the time of Post-Treatment Follow-Up discontinuation and follow-up and for any further evaluations that need to be completed.

After discontinuation from the Post-Treatment Follow-Up period, participants will enter the LTFU period (Section 8.5.9).

7.2. Participant Discontinuation/Withdrawal from the Study

Long-term follow up after gene modified adoptive cell therapy is specified by the FDA and other health authorities in order to monitor for potential toxicity and any long-term adverse events. Nonetheless, a participant may withdraw from the study at any time at his/her own request or may be withdrawn at any time at the discretion of the investigator for safety, behavioral, compliance, or administrative reasons without prejudice to their future medical care by the study physician or institution.

A participant can withdraw from study treatment and/or protocol required procedures at any time but may continue to be followed for survival and subsequent anti-cancer therapy during Long-Term Follow-Up. This is referred to as a partial withdrawal of consent.

If possible, an End of Post-Treatment Follow-Up visit should be conducted, as shown in the SOA (see Section 1.4).

If a participant withdraws or is withdrawn entirely from the study, including Long-Term Follow-Up, the following apply:

- The participant will be permanently discontinued both from the study intervention and from the study at that time.
- If the participant withdraws consent for disclosure of future information, the Sponsor may retain and continue to use any data collected before such a withdrawal of consent.
- If a participant withdraws from the study, he/she may request destruction of any samples taken and not tested, and the investigator must document this in the site study records.

- The investigator and/or Sponsor can also decide to withdraw a participant from the investigational product and/or protocol-required therapies, protocol-specific procedures, or the study as a whole at any time prior to study completion.

In rare circumstances where participants relocate during the course of the study or for those who have geographical concerns (e.g., participant referred from out of state but cared for at another center) toxicity and other clinical assessments may be collected via notes from the local physician and/or phone interviews/videoconferencing during the Post-Treatment Follow-Up portion of the study as described in the SOA with periodic assessments done at the study site.

7.3. Post-Study Access

Currently, PACT Pharma does not have any plans to provide NeoTCR-P1 or other study interventions to participants who have completed the study or who have withdrawn earlier for any reason.

7.4. Lost to Follow Up

A participant will be considered lost to follow-up if he or she repeatedly fails to return for scheduled visits and is unable to be contacted by the study site.

The following actions must be taken if a participant fails to return to the clinic for a required study visit:

- The site must attempt to contact the participant and reschedule the missed visit as soon as possible and counsel the participant on the importance of maintaining the assigned visit schedule and ascertain whether or not the participant wishes to and/or should continue in the study.
- Before a participant is deemed lost to follow up, the investigator or designee must make every effort to regain contact with the participant (where possible, 2 telephone calls and, if necessary, a certified letter to the participant’s last known mailing address or local equivalent methods). These contact attempts should be documented in the participant’s medical record.
- Should the participant continue to be unreachable, he/she will be considered to be lost to follow-up.

Discontinuation of specific sites or of the study as a whole are handled as part of Appendix 9.1.

8. Study Assessments and Procedures

The following assessments and procedures apply during this study:

- Study procedures and their timing are summarized in the SOA (see Section 1.4). Protocol waivers or exemptions are not allowed.
- Immediate safety concerns should be discussed with the Sponsor immediately upon occurrence or awareness to determine if the participant should continue or discontinue study intervention.

- Adherence to the study design requirements, including those specified in the SOA (see Section 1.4), is essential and required for study conduct.
- All screening evaluations must be completed and reviewed to confirm that potential participants meet all eligibility criteria. The investigator will maintain a screening log to record details of all participants screened and to confirm eligibility or record reasons for screening failure, as applicable.
- Procedures conducted as part of the participant's routine clinical management (e.g., blood count) and obtained before signing of the informed consent form (ICF) may be utilized for screening or baseline purposes provided the procedures met the protocol-specified criteria and were performed within the time frame defined in the SOA (see Section 1.4).

8.1. Informed Consent

Written informed consent for participation in the study must be obtained before performing any study-related procedures. ICFs for enrolled participants and for *participants* who are not subsequently enrolled will be maintained at the study site.

The investigator will maintain a screening log to record details of all *participants* screened and to record reasons for screening failure.

8.2. Screening Assessments

8.2.1. Medical and Treatment History, Concomitant Medications, and Demographic Information

Medical history includes clinically significant diseases within the previous 5 years and all medications used by the *participant* within 7 days before the Screening Part 2 visit (including prescription, over-the-counter and herbal/homeopathic remedies and therapies).

Cancer history including characteristics about the tumor, such as cancer stage, hormone receptor status, prior cancer therapies, and procedures.

Demographic data will include age, sex, and self-reported race/ethnicity to study their possible association with participant safety and anti-tumor activity.

8.2.2. Physical Examination

Physical examinations will be performed during screening and at times noted in the SOA.

A complete physical examination will include, at a minimum, assessments of the head, eyes, ears, nose, and throat, the cardiovascular, respiratory, gastrointestinal, dermatological, musculoskeletal, and neurological systems. If indicated based upon the medical history, symptoms, and/or the disease under study rectal, external genitalia, breast, and pelvic exams should be performed. Any abnormality identified at baseline should be recorded in the eCRF. Height and weight will also be measured and recorded.

At subsequent visits (or as clinically indicated) limited physical examination should be performed and will include, at a minimum, assessments of the skin, lungs, cardiovascular system, and abdomen (liver and spleen) as well as any symptom-directed assessments.

All participants should be monitored for symptoms of brain metastases and neurotoxicity. Symptoms suggesting of either should prompt a full neurological examination.

Changes from baseline abnormalities should be recorded in participant notes. New or worsened clinically significant abnormalities should be recorded as adverse events.

8.2.3. Vital Signs

Vital signs will include measurements of temperature, pulse rate, respiratory rate, and systolic and diastolic blood pressure. Blood pressure and pulse measurements should be preceded by at least 5 minutes of rest for the participant in a quiet setting. Vital signs should be measured while the participant is in a semi-supine position

During IP administration/hospitalization, vital signs should be monitored before and after the NeoTCR-P1 infusion and then routinely (every 4–6 hours) while hospitalized. If the participant has a fever (temperature 38.3ºC or greater) at any time during hospitalization, vital signs should be monitored more frequently, as clinically indicated.

Vital signs will be measured and recorded in the eCRF as described in Table 27.

Table 27 Vital Sign Measurement at Day 0

Day 0	Timepoints
Phase 1a	• Within 60 minutes prior to NeoTCR-P1 infusion<br>• During the infusion period (every 15 [±5] minutes) and 30 (±10) minutes after NeoTCR-P1 infusion has completed
Phase 1b	• Within 60 minutes prior to NeoTCR-P1 infusion<br>• During the NeoTCR-P1 infusion period (every 15 [±5] minutes) and 30 (±10) minutes after NeoTCR-P1 infusion followed by every 15 [±5] minutes during the nivolumab infusion and within 30 (±10) minutes after nivolumab infusion has completed
**Subsequent Cycles**	
Phase 1b	• Within 60 minutes prior to nivolumab infusion<br>• During and 30 (+/-10) minutes after nivolumab infusion if clinically indicated or symptoms occurred during the prior infusion

PD-1 = programmed death 1.

8.3. Efficacy Assessments

8.3.1. Tumor and Response Evaluations

All known sites of disease must be documented at screening and re-assessed at each subsequent tumor evaluation. Response will be assessed by the investigator per RECIST v1.1 (see Appendix 9.6). Scans may be collected to enable a central independent review.

Screening assessments must include CT scans of the chest, abdomen, and pelvis (with oral/IV contrast unless otherwise contraindicated) or MRIs of the abdomen and pelvis along with a noncontrast CT of the chest in *participants* with contraindications to contrast.

If a CT scan for tumor assessments is performed in a positron emission tomography (PET)/CT scanner, the CT acquisition must be consistent with the standards for a full-contrast diagnostic CT scan. For *participants* with liver metastases, a multiphasic MRI or CT scan of the liver should be performed. Bone scans and CT scans of the neck should also be performed if clinically indicated. At the investigator's discretion, other methods of assessment of measurable disease as per RECIST v1.1 may be used.

For subsequent tumor assessments, procedures for tumor assessment should performed per the SOA and as clinically indicated. Unless there is a contraindication, the same imaging method used at screening must be used throughout the study. If local health authorities require the use of specific imaging modalities (e.g., MRI) and/or a different imaging schedule for disease response assessments purposes a decision will be made on a case-by-case basis upon discussion between the Sponsor and the Investigator.

Assessment of tumor markers should be performed with each tumor assessment and as clinically indicated per Table 28.

Table 28 Tumor Assessments per Indication

	Physical Examination	CT/MRI	Tumor Markers	On-Tx Biopsy
Melanoma	x	x	NA	x
Urothelial Carcinoma	x	x	NA	x
NSCLC	*x*	*x*	*NA*	*x*
HNSCC	*x*	*x*	*NA*	*x*
CRC	x	x	CEA	x
Ovarian Cancer	x	x	CA-125	x
HER2-negative Breast Cancer	x	x	CEA, CA27.29 and/or CA15-13 (if positive at baseline)	x
Prostate Cancer	x	x (Bone scan if applicable)	PSA	x

CEA = carcinoembryonic antigen; CT = computed tomography; CRC = colorectal cancer; HR+ = hormone receptor positive; MRI = magnetic resonance imaging; NA = not applicable; PSA = prostate-specific antigen; Tx = treatment.

8.3.2. *Participants* with Prostate Cancer

Screening assessments must include CT scans of the chest, abdomen, and pelvis (with oral/IV contrast unless contraindicated) and a bone scan. Bone scans are required to assess tumor progression in *participants* with prostate cancer. Therefore, if bone lesions are observed at baseline, then bone scans should be obtained at the same frequency as CT scans, at the time of the protocol-specified tumor assessment. For *participants* with prostate cancer who do not have bone lesions at baseline, bone scans will be initiated per investigator discretion when clinically indicated. If bone lesions develop during the study, the bone scans should continue to be done at the same frequency as CT scans.

If a participant is eligible for retreatment with NeoTCR-P1, the last scan prior to retreatment will be considered the baseline for the purpose of evaluating the response to retreatment.

8.4. Safety Assessments

Planned timepoints for all safety assessments are provided in the SOA (see Section 1.4). Baseline and ad hoc safety assessments are described below

8.4.1. Cardiac Function

Each participant's cardiac function, as measured by echocardiography will be assessed during the screening period to confirm eligibility. Both left ventricular ejection fraction and the pericardial space (to rule out a pericardial effusion) will be assessed prior to study entrance by echocardiography. An echocardiography performed following the participant's last chemotherapy treatment and within 28 days prior to signing the consent may be used for the confirmation of eligibility.

At Screening, a 12-lead ECG will also be performed to establish a baseline.

8.4.2. Neurological Assessment

Neurological assessments will be standardized by using the ICE, version 2.0 (see Table 24). The ICE is a 5–10 minute screening tool that examines various areas of cognitive function: orientation, attention, immediate recall, short-term recall, language, and the ability to follow verbal and written instructions.

A full neurological assessment will be completed during screening to establish a baseline. Subsequent assessments will be performed before NeoTCR-P1 per the SOA. Every attempt should be made to standardize the assessment and to minimize interrater variability.

8.4.3. MRI/CT Brain

A brain scan (CT with IV contrast or MRI *with contrast whenever possible or without contrast in case of contraindication*) must be obtained at baseline.

Symptoms suggestive of new or worsening CNS metastases should prompt a full neurological examination. A CT or MRI scan of the head should be done as clinically indicated to confirm or refute new or worsening brain involvement. Stable brain metastases must be evaluated with each tumor assessment with the same radiographic procedure as at the baseline study. Participants without brain metastases do not need brain scans for tumor assessment unless clinically warranted.

Evaluation of any new onset of Grade ≥2 neurotoxicity should include a brain MRI as described in Section 6.11.7.

8.4.4. Electrocardiograms

Twelve-lead ECG will be obtained as outlined in the SOA (see Section 1.4) and as clinically indicated. ECGs should be obtained on the same machine whenever possible. Lead placement

should be as consistent as possible. ECG recordings should be performed after the participant has been resting in a supine position for at least 10 minutes.

For safety monitoring purposes, the investigator must review, sign, and date all ECG tracings. Clinically significant findings must be discussed with the Medical Monitor prior to enrolling the *participant* in the study. Paper copies of ECG tracings will be kept as part of the *participant's* permanent study file at the site. Any morphologic waveform changes or other ECG abnormalities must be documented on the eCRF. The date and time of the ECG and the following parameters will be collected and assessed: QTcF, PR, QT, and QRS interval in msec, and heart rate (bpm).

8.4.5. Bone Marrow Biopsy

Bone marrow aspirate/biopsy should be considered to evaluate HLH as indicated in Section 6.11.6. If a bone marrow sample is collected to evaluated HLH or other toxicities (e.g., prolonged cytopenia) a portion should be submitted to the central laboratory for additional analysis.

8.4.6. Lumbar Puncture

Participants with new symptoms of CNS involvement such as new onset severe headaches, neck stiffness, any focal neurologic findings or new onset Grade ≥2 neurotoxicity following NeoTCR-P1 infusion may have lumbar puncture performed. Adequate platelet support should be provided prior to performing a lumbar puncture (e.g., platelet count >50,000/mm^3).

8.4.7. Clinical Laboratory Assessments

Samples for hematology, serum chemistries, urinalysis, and the pregnancy test will be analyzed at the study site's local laboratory. Central laboratories will coordinate the collection of archival tumor, newly collected tumor and leftover tumor tissue and blood samples for the assessment of NeoTCR-P1 pharmacokinetics and pharmacodynamics, exploratory biomarker assessment, anti-drug antibody assays (Phase 1b only). Instruction manuals and laboratory kits will be provided for all central laboratory assessments.

8.4.7.1. Local Laboratory Assessments Will Include the Following:

- Hematology (complete blood count [CBC] including hemoglobin, hematocrit, *white blood cell (*WBC*)* count (with differential) and platelet count
- CD3+ T-cell count (during Screening Part 2)
- Chemistries (glucose, BUN, creatinine, sodium, potassium, chloride, bicarbonate, calcium, phosphate, uric acid, total bilirubin, ALT, AST, alkaline phosphatase, LDH, and albumin)

  Phosphate and uric acid may be omitted after Day 28.
- Amylase and lipase
- Urine or serum pregnancy test
- Urinalysis
- Thyroid function testing

- HBV serology
- *Hepatitis C virus (*HCV*)* serology (This test is not required if documentation of a negative result of an HCV RNA test performed within 60 days prior to screening is provided)
- HIV
- EBV serology
- *Cytomegalovirus (*CMV*)* serology
- PSA, CA125, CA27.9, and *carcinoembryonic antigen (*CEA*)* (if applicable)
- CRP
- Ferritin

The investigator must review the laboratory report, document this review, and record any clinically relevant changes occurring during the study in the AE section of the *electronic* case report form (*e*CRF). The laboratory reports must be filed with the source documents. Clinically significant abnormal laboratory findings are those that are not associated with the underlying disease, unless judged by the investigator to be more severe than expected for the participant's condition.

All laboratory tests with abnormal values that are considered clinically significantly should be repeated until the values return to normal or baseline or are no longer considered clinically significant by the investigator or Medical Monitor.

8.4.7.2. Central Laboratory Assessments

All central laboratory assessments should be collected at *the* intervals specified in the SOA. The following assessments will be performed at a central laboratory or at PACT:

- PK, Biomarker assessment, immune monitoring, and imPACT analysis from PBMC samples
- Biomarker assessments from plasma
- PK/PD analysis from serum/plasma
- Sequencing and exploratory biomarker assessments from tumor tissue

Refer to the central laboratory manual *and supply kits provided* for additional details on sample collection, processing, and shipping instructions.

Samples collected may be exhausted or stored up to 15 years from final clinical study report at PACT or designated 3rd party repository.

8.4.8. Leukapheresis

A leukapheresis procedure will be carried out at the apheresis center for manufacturing. PBMCs are obtained for NeoTCR-P1 manufacture during this procedure. Baseline blood leukocytes for

FDA look-back requirements and for research are also obtained and cryopreserved. See Section 8.5.3 for additional details.

A single volume leukapheresis for immune monitoring is required in dose-escalation *participants*. See the SOA and leukapheresis manual for additional details.

8.5. Description of Study Periods

Investigative sites will maintain a log of all screened participants who were reviewed and evaluated for study participation. Information collected on the screening log should include limited information such as the date of screening, date the participant was enrolled or the reason for screen failure.

8.5.1. Screening

The screening period begins on the date the participant signs the informed consent and continues through enrollment. Informed consent must be obtained before any non-standard of care procedures. Procedures that are part of standard of care are not considered study specific procedures and may be performed prior to obtaining consent and used to confirm eligibility. Confirmation of this data must occur within the time window as outlined in the SOA (see Section 1.4).

Screening will be separated into 2 parts (i.e., Part 1 and Part 2). The purpose of this separation is to allow for the identification of neoepitope-specific TCRs while participants are receiving other therapies.

During Screening Part 1, participants will be comprehensively evaluated to ensure they do not have irreversible disqualifying conditions that would prohibit the administration of experimental treatment.

Participants with successful TCR selection, either during Part 1 of this study or under a separate screening protocol, may proceed to Screening Part 2.

Participants who meet the *Screening Part 2* eligibility criteria and *are approved to* commence leukapheresis will be *considered* enrolled in the study. If at any time prior to enrollment the *participant* fails to meet the eligibility criteria, the *participant* should be designated as a screen failure on the *participant* screening log with the reasons for failing screening.

8.5.1.1. Screening Part 1

Screening Part 1 *eligibility assessments must be completed within 28 days after the date of informed consent.*

Tumor samples and whole blood sample *for PBMCs* are to be *collected* during Part 1 of screening *after initial eligibility criteria have been met and following approval by the Medical Monitor. If whole blood samples are determined not to be optimal for imPACT analysis, participants may be requested to undergo a small-volume apheresis to increase the number of cells available for processing.*

Tumor specimens and blood samples for PBMCs must be submitted within 28 days after Medical Monitor approval to proceed.

To decrease the risk that participants suffer a significant decline in clinical status during the time required for product selection and manufacturing (approximately 4-6 weeks), it is encouraged that Screening Part 1 be initiated during earlier lines of therapy in order to decrease the time from disease progression/relapse to neoTCR infusion. Measurable disease is not required to be demonstrated at this time.

Figure 15 Screening Part 1

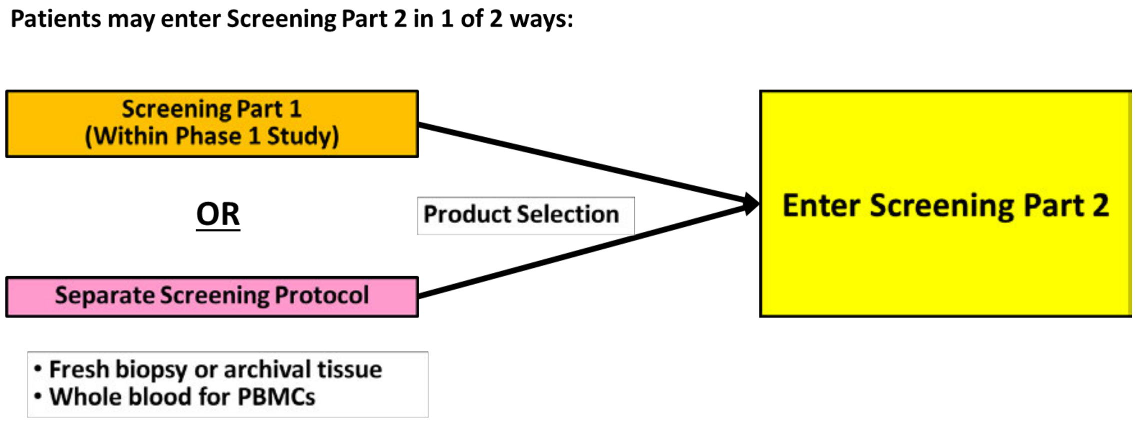

8.5.1.1.1 Tumor Tissue

Tumor tissue provided during Screening Part 1 should be of good quality based on total and viable tumor content. Tissue must have been obtained within 1 year prior to consent unless discussed with medical monitor.

Tumor tissue samples consisting of surgical resection, core needle biopsies for deep tumor tissue/organs, or excisional/punch biopsies for cutaneous or subcutaneous lesions will be accepted:

- For cutaneous or subcutaneous lesions, tumors should be 5 mm in diameter
- For core needle biopsy specimens, a minimum of 3 × (18G or larger) cores, 1 cm long or greater should be submitted for evaluation.

Minimum sample requirements to enable sequencing are *≥25 mm^2 surface area, with* ≥20% tumor content *and* approximately ≥1.0 mm^3 tumor volume*/10 mm^2 tumor surface area.*

For archival tumor tissue, paraffin blocks are preferred or 20 (or more) 5-micron, serial, freshly cut, unstained slides or curls must be submitted. The site pathologist should confirm that the specimen meets requirements to enable sequencing prior to shipment.

Fine needle aspiration (FNA), brushing, and lavage samples are <u>not acceptable</u>. Tumor tissue from bone metastasis is not advised because decalcification procedures may degrade DNA in the specimen.

In addition, *separate from the tumor tissue provided in Screening Part 1,* NGS data (e.g., BAM file, FASTQ files) or archival tumor tissue >1 year old, or tissue from multiple primary/metastatic sites may be collected and submitted for analysis.

Further details can be found in the Laboratory Manual.

8.5.1.2. Screening Part 2

Participants with successful TCR selection will be assessed against Screening Part 2 eligibility criteria prior to undergoing leukapheresis. The ECHO, ECG, PFT, tumor assessment, and brain MRI must be performed during Screening Part 2.

There is a 28-day window for *Screening* Part 2.

8.5.2. Rescreening

Participants who fail to meet the eligibility criteria will be allowed to rescreen 1 time. Participants will perform the assessment that initially resulted in the participant failing screening including any other procedures that fell outside of the designated screening window (i.e., laboratory assessments, CT scans).

8.5.3. Enrollment/Leukapheresis

Participants who meet the *Screening Part 2* eligibility criteria *and are approved for leukapheresis are considered to be enrolled. Enrolled participants should* undergo leukapheresis to obtain leukocytes for the manufacturing of NeoTCR-P1 within approximately 5 days. *In some circumstances, participants may be approved to undergo leukapheresis prior to meeting all eligibility criteria (e.g., in the absence of measurable disease, but at an opportune moment in their treatment course). In these cases, enrollment will be defined to occur when the participant subsequently does meet all the criteria.* Participants must have no evidence of a clinically significant infection prior to leukapheresis. Should a participant have a clinically significant infection immediately prior to leukapheresis, cell collection must be delayed until the event resolves. If leukapheresis is delayed beyond 5 days, then baseline CBC with differential and chemistry panel must be repeated to confirm eligibility.

It is strongly recommended that leukapheresis be scheduled prior to any planned bridging chemotherapy as a low absolute T-cell count will result in poor T-cell collection and a potential dose failure. In case of emergent logistical challenges (e.g., availability of manufacturing slots) or where there is concern that subsequent lines of chemotherapy may affect PBMC yield or impair cell therapy potential, *participants* may undergo leukapheresis prior to the completion of all eligibility criteria, following discussion between the Investigator and Medical Monitor (Das et al., 2019).

Once an eligible participant commences leukapheresis, the participant is considered enrolled in the study. For participants who undergo leukapheresis prior to completion of all eligibility criteria, enrollment begins once all eligibility criteria are met in Screening Part 2.

Immunosuppressive drugs including corticosteroids must be avoided for 7 days prior to leukapheresis. In addition, a wash-out is required following anticancer therapy and the start of leukapheresis.

Figure 16 Cessation of Therapies Prior to Leukapheresis

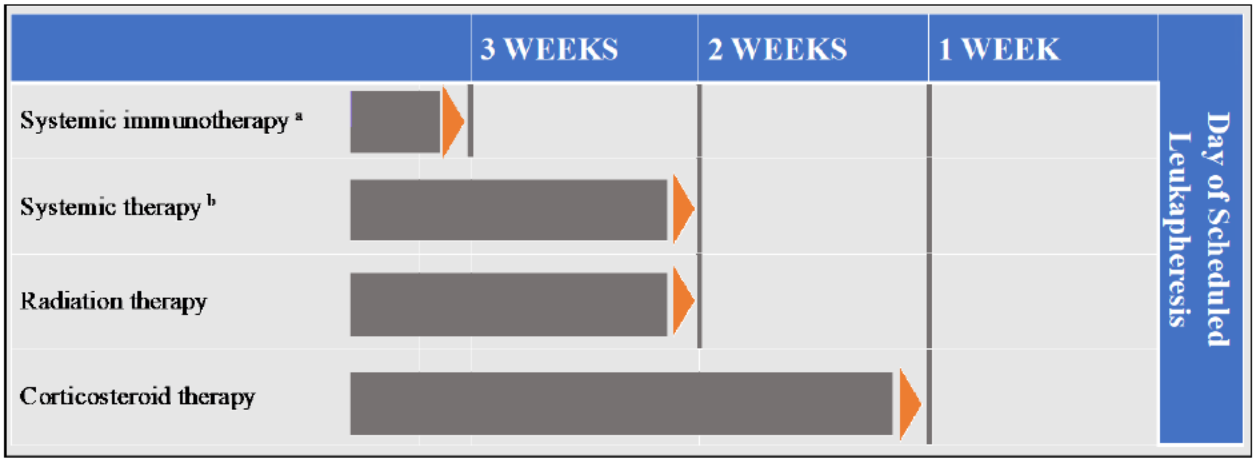

Note: The figure is to provide general guidelines.

[a] At least 3 weeks must have elapsed from any prior systemic inhibitory/stimulatory immune checkpoint molecule therapy (e.g., ipilimumab, nivolumab, pembrolizumab, atezolizumab, OX40 agonists, 4-1BB agonist, anti-TIGIT, LAG3 inhibitor) prior to planned leukapheresis.

[b] At least 2 weeks or 5 half-lives, whichever is shorter, must have elapsed from any approved anti-cancer therapy (chemotherapy, hormonal therapy, targeted therapy, or radiotherapy) prior to planned leukapheresis

Mononuclear cells will be obtained by leukapheresis (12–15 liters will be performed with a goal to target approximately 5–10 × 10^9 mononuclear cells). The leukapheresed cells are then packaged for expedited shipment to the CPF. If a single apheresis does not yield an adequate manufactured dose or a manufacturing failure occurs, then participants may undergo an additional apheresis as needed.

Upon arrival at the CPF, each participant's leukapheresed product will be processed to enrich for T cells. The T cells are then stimulated to expand and transfected with a plasmid to knock out the native, endogenous TCR in each T cell and to introduce the mutation-targeted neoTCR genes into the T cell genome, precisely at the endogenous TCR genome locus. The cells are then expanded and cryopreserved to generate the investigational product. Once the NeoTCR-P1 product has passed release and sterility testing, it will be shipped back to the treating facility.

Following leukapheresis, participants may receive bridging therapy per Principal Investigator discretion with available standard of care for that tumor type to maintain disease control, but must discontinue 2 weeks prior the start of conditioning chemotherapy (see Section 6.5). Investigational therapy is not permitted after leukapheresis.

8.5.4. Baseline Biopsy

Following leukapheresis and prior to the initiation of conditioning chemotherapy, participants who submitted tumor tissue for the identification of a neoepitope-specific TCR and have

received intervening therapy must undergo collection of a fresh tumor specimen if clinically feasible in order to retrospectively determine whether there have been any changes in the presence of tumor neoantigens or HLA loss and for exploratory biomarker analysis.

Tumor tissue samples consisting of surgical resection, core needle biopsies for deep tumor tissue/organs, or excisional/punch biopsies for cutaneous or subcutaneous lesions will be accepted.

- For cutaneous or subcutaneous lesions, tumors should be ≥5 mm in diameter
- For core needle biopsy specimens, a minimum of 3 × (18G or larger) cores, 1 cm long or greater should be submitted for evaluation.

FNA, brushing, and lavage samples are not acceptable. Tumor tissue from bone metastasis is not advised because decalcification procedures may degrade DNA in the specimen.

Refer to Central Laboratory Manual for additional details.

8.5.5. Re-evaluation Prior to Conditioning Chemotherapy

In the time between enrollment and the start of conditioning chemotherapy, changes may occur relative to the initial eligibility assessment (Screening Part 2) that could increase the risk to study participants. Prior to the infusion of conditioning chemotherapy, participants must be reassessed by the treating physician per the SOA (see Section 1.4). If any results are beyond the criteria established for eligibility, participants may not *proceed with conditioning chemotherapy* until the abnormalities have resolved unless discussed with the Sponsor.

If Screening Part 2 tumor assessments were performed >28 days prior to the initiation of conditioning chemotherapy or if a participant received bridging anti-cancer therapy after enrollment, scans must be repeated prior to conditioning chemotherapy to establish a new baseline. Baseline imaging studies should be performed as close to the initiation of conditioning chemotherapy as possible.

8.5.6. Conditioning Chemotherapy

Participants will receive a non-myeloablative conditioning regimen consisting of fludarabine 30 mg/m^2 IV daily *(Day -6 through Day -3)* and cyclophosphamide *600* mg/m^2 IV daily *(Day -6 through Day -4)* prior to the NeoTCR-P1 infusion to induce lymphocyte depletion and create an optimal environment for expansion of neoTCR-engineered T cells *in vivo*. Participants will initiate the *4*-day conditioning cycle within *6* days prior to the NeoTCR-P1 infusion (i.e., Day *-6,* -5, Day -4, and Day -3) to allow for at least 48 hours from the last dose of chemotherapy to NeoTCR-P1 infusion.

Conditioning chemotherapy should commence only after cell product is available at the site.

The 4-day conditioning regimen may be administered as an outpatient regimen by a *participant's* local oncologist within the specified time frame depending upon the need for intravenous fluids. Participants should be kept well hydrated and closely monitored to prevent fluid overload.

8.5.7. Inpatient IP Administration

On Day 0 prior to the infusion, participants will have a physical exam, medical history, performance status evaluation, and CBC with differential to reassesses suitability for administration.

Prior to NeoTCR-P1 infusion, participants must not have:

- Pulmonary: Requirement for supplemental oxygen to keep saturation greater than 95% or presence of radiographic abnormalities on chest x-ray that are progressive
- Cardiac: Any cardiac arrhythmia or other significant cardiac dysfunction not controlled with medical management
- Hypotension requiring pressor support
- Active Infection: Positive blood cultures or tests for bacteria, fungus, or viruses
- Neurological: Acute neurological toxicity Grade >1 (with the exception of peripheral sensory neuropathy)
- Renal: Serum creatinine >2.0 mg/dL

In addition to the above criteria, if the participants' temperature is ≥38.0º C within 48 hours prior to NeoTCR-P1 infusion a call must be made to the Medical Monitor prior to proceeding with the NeoTCR-P1 infusion. Furthermore, participants must not be receiving systemic anti-microbials for the treatment of an active infection within 48 hours before NeoTCR-P1 administration (prophylactic use of anti-microbials [e.g., chronic recurrent urinary tract infection] are permitted). Corticosteroids (≥5 mg per day of prednisone equivalent) and other immunosuppressive drugs must be avoided for at least 5 days prior to NeoTCR-P1 administration.

Prior to infusion, investigators must document that a participant has met infusion criteria and has not experienced a significant change in performance status compared to initial eligibility screening. Should an event exceed these criteria immediately prior to receiving NeoTCR-P1, the infusion must be held until the event resolves.

If the NeoTCR-P1 infusion is delayed >2 weeks, conditioning chemotherapy must be repeated unless otherwise agreed between the investigator and the Medical Monitor. In all cases of NeoTCR-P1 infusion delays, contact the Medical Monitor for guidance.

Investigators will need to confirm that 2 doses of tocilizumab are available on site prior to NeoTCR-P1 infusion and 1 dose of siltuximab (if available in country) must be accessible within 24 hours of infusion in order to manage suspected toxicities.

Following NeoTCR-P1 infusion, participants will remain in the hospital until the following criteria are met and following the clinical judgment of the treating physicians:

- ANC >500/µL
- Platelet count >20,000/µL

- Hemodynamically stable
- Creatinine on downward trend
- Liver function tests stable
- Not requiring daily blood product infusion

Participants may be discharged with non-critical and clinically stable or slowly improving toxicities (e.g., mildly elevated creatinine) even if Grade >1.

Following discharge, participants will need to remain within 60-minutes travel of the investigative site for the first 28 days. Participants will be required to monitor their temperature at least once daily during the first 28 days following infusion of NeoTCR-P1 *and record their temperature on a paper diary provided by study staff. Study staff must review the participant's completed diary and retain it in the participant's study records.* Participants and their caregivers will be counseled regarding the signs and symptoms of CRS and neurotoxicity and will be instructed to immediately contact their study physician and/or seek medical attention. In addition, a wallet card will be provided to participants who have been infused with NeoTCR-P1.

8.5.8. Post Treatment Assessment Period

After completing the NeoTCR-P1 infusion and following discharge from the hospital, all participants will be followed in the post treatment assessment period.

Following the initial infusion period, participants will return to the clinic weekly until Week 4, monthly until Month 6, and then every 3 months until Month 24.

On approximately Day 28, participants will undergo evaluation to assess engraftment and to assess any changes in the T-cell repertoire. A blood draw will be performed to obtain PBMCs to monitor change in the presence of neoepitope-specific TCRs (i.e., imPACT assay).

Response assessments will be performed at Day 28, Day 56 and then approximately every 2 months for the first year and every 3 months for the second year.

Tumors will be evaluated using RECIST v1.1. Assessment of tumor markers (e.g., PSA, CA125, CEA) should be performed with each tumor assessment and as clinically indicated.

A tumor biopsy must be obtained between Days 5-7 *and/or Days 28–42,* if clinically feasible and safely accessible, to evaluate for the presence of intratumoral T cells and for exploratory biomarker analysis.

An additional on-treatment biopsy sample may be collected per investigator discretion in consenting participants at the time of radiographic progression to evaluate for mechanisms of resistance and for exploratory analysis.

Tumor tissue consisting of surgical resection, core needle biopsies for deep tumor tissue/organs or excisional/punch biopsies for cutaneous or subcutaneous lesions is requested:

- For cutaneous or subcutaneous lesions, tumors should be ≥5 mm in diameter.
- For core needle biopsy specimens, a minimum of 3× (18G or larger) cores, 1 cm long or greater should be submitted for evaluation.

FNA, brushing, and lavage samples are not acceptable. Tumor tissue from bone metastasis is not advised because decalcification procedures may degrade DNA in the specimen.

Refer to the central laboratory manual for additional details.

For participants in the dose-escalation portion of the study a single volume leukapheresis procedure will also be obtained between Days 28-35 for immune monitoring.

If a participant is discharged from the hospital and is subsequently re-admitted to the hospital with any potential NeoTCR-P1 related adverse event(s), the following ad hoc labs will be collected in addition to the scheduled labs in the SOA:

- Whole blood for PBMCs on day of admission, 24 hours later, then weekly and on day of discharge
- Cytokine levels on day of admission, 24 hours later, then weekly and on day of discharge

At any time during the post treatment assessment period, if a participant did not respond to treatment (i.e., did not achieve a CR, PR or SD >6 months) or progresses following a response and is either not eligible for retreatment or chooses not to pursue re-treatment, the participant may move to long-term follow up and be followed for survival, subsequent therapy and disease outcomes in the long-term follow-up. A whole blood collection for PBMCs and a biopsy should be collected at the time of progression (biopsy if clinically feasible), prior to starting any subsequent new anti-cancer therapy.

8.5.9. Long-Term Follow-Up Period

All enrolled participants will be followed in the long-term follow-up period for survival and safety.

After 2 years (or less in non-responders), participants will enter the LTFU period where they will be assessed annually for targeted adverse events and OS. If a participant's disease has not progressed by Month 24, disease assessments will continue to be performed per the local standard of care.

Targeted AE/SAE reporting should include neurological, hematological, infections, autoimmune disorders, and secondary malignancies.

Targeted concomitant medication documentation should include immunosuppressive drugs and anti-infectives.

The overall follow up from the date of the initial cell transfer will be 15 years. Procedures for participants who are enrolled and receive NeoTCR-P1 are detailed in the SOA. Participants in

LTFU may be contacted by telephone to confirm survival status, targeted AE/SAE reporting and to report targeted concomitant medication use.

At a future date, long-term follow-up may be conducted under a separate protocol.

Should the participant fail to return to clinic or withdraw from the study, the site's staff may use a public information source (e.g., county records) to obtain information about survival status only.

8.5.10. Cross-Over

Cross-over of participants in the Phase 1a to nivolumab is not permitted.

8.5.11. Retreatment

Under circumstances where participants initially respond (i.e., CR, PR, or prolonged SD >6 months) and subsequently relapse or progress, participants may be eligible for a second course of NeoTCR-P1 (at DLT cleared dose) with or without conditioning chemotherapy if they meet the following criteria:

- Disease progression must occur greater than 3 months after NeoTCR-P1 infusion.
- Continues to meet the original study eligibility criteria with the exception of prior NeoTCR-P1 use in the study.
- Participant has not received subsequent therapy for the treatment of their underlying malignancy with the exception of radiation or surgical excision of a brain metastasis.
- Participant did not experience a DLT in the dose-escalation component or a comparable toxicity in the expansion cohorts.
- Toxicities related to conditioning chemotherapy (i.e., fludarabine and cyclophosphamide), with the exception of alopecia, have resolved to Grade ≤1 or returned to baseline prior to re-treatment.
- Sufficient cell dose available from original manufacturing procedure to allow for retreatment

The decision to administer re-treatment should be made in consultation with the Medical Monitor. In addition, a discussion regarding benefits and risks of retreatment including potentially available therapies should occur with the participant prior to performing any study-related procedures or treatment. This conversation should be documented in the *participant's* medical record.

A maximum of 1 retreatment course per participant may occur. Participants who are retreated will continue to undergo study procedures per the Schedule of Activities for 2 years from Day 0.

Participants will receive the NeoTCR-P1 dose selected for the expansion cohorts if they are retreated. If the expansion cohort dose has not yet been selected, participants will receive the last NeoTCR-P1 regiment that was determined to be safe by the SRT.

The allowance for retreatment is based upon clinical experience reported in studies conducted at the NCI where subjects were re-treated upon progression and experienced durable response to retreatment (Tran et al., 2014; Kochenderfer et al., 2015; Lee et al., 2015).

8.6. Adverse Events and Serious Adverse Events

AEs will be reported by the participant (or, when appropriate, by a caregiver, surrogate, or the participant's legally authorized representative).

The investigator and any qualified designees are responsible for detecting, documenting, and recording events that meet the definition of an AE or SAE and remain responsible for following up AEs that are serious, considered related to the study intervention or study procedures, or that caused the participant to discontinue fludarabine, cyclophosphamide, NeoTCR-P1, IL-2, or the combination of NeoTCR-P1 and nivolumab (see Section 7).

All adverse events, whether reported by the participant or noted by the participant or noted by study personnel, will be recorded in the participant's medical record on the Adverse Event eCRF.

8.6.1. Time Period and Frequency for Collecting AE and SAE Information

Report all SAEs deemed related to protocol-mandated procedures from the signing of informed consent through to administration of conditioning chemotherapy (fludarabine/ cyclophosphamide).

Report all AEs, including SAEs, from the time of conditioning chemotherapy administration until 90 days after the last dose of study drug (NeoTCR-P1, IL-2, or nivolumab), whichever was administered last.

After 90 days, report any serious targeted adverse events (e.g., neurological, hematological, infections, autoimmune disorders, and secondary malignancies) observed by the investigator or reported by the participant, regardless of causality to study drug. In addition, report any SAEs considered related to NeoTCR-P1 or the combination of NeoTCR-P1 and nivolumab.

For participants who screen fail or are enrolled, but do not receive Neo-TCR-P1, the reporting period for SAEs ends 30 days after the last procedure (e.g., screening procedure, leukapheresis, conditioning chemotherapy).

Table 29 Summary of AE Collection Periods

Time Period	AE Collection
ICF signature to conditioning chemotherapy	Protocol/procedure-related SAEs only
Conditioning chemotherapy to 90 days after last study drug dose (NeoTCR-P1, IL-2, or nivolumab, whichever was administered last)	All AEs and SAEs
Beyond 90 days after last study drug dose (NeoTCR-P1, IL-2, or nivolumab, whichever was administered last)	Serious, targeted adverse events (e.g., neurological, hematological, infections, autoimmune disorders, and secondary malignancies), regardless of causality to study drug<br>Any related SAEs to NeoTCR-P1 with or without IL-2 or to the combination of NeoTCR-P1 and nivolumab (1b)

AE = adverse event; ICF = informed consent form; SAE = serious adverse event.

All SAEs and non-serious adverse events of special interest will be recorded and reported to Sponsor or designee within 24 hours following the investigators knowledge of the events, as indicated in Appendix 9.3. Refer to Section 8.6.7 for a list of protocol-defined adverse events of special interest. The investigator will submit any updated SAE data to the Sponsor within 24 hours of it being available.

Investigators are not obligated to actively seek AE or SAE after conclusion of the study participation. However, if the investigator learns of any SAE, including a death, at any time after a participant has been discharged from the study, and he/she considers the event to be reasonably related to the study intervention or study participation, the investigator must promptly notify the Sponsor.

8.6.2. Method of Detecting AEs and SAEs

The method of recording, evaluating, and assessing causality of AE and SAE and the procedures for completing and transmitting SAE reports are provided in Appendix 9.3.

Care will be taken not to introduce bias when detecting AEs and/or SAEs. Open-ended and non-leading verbal questioning of the participant is the preferred method to inquire about AE occurrences.

8.6.3. Follow-up of AEs and SAEs

After the initial AE/SAE report, the investigator is required to proactively follow each participant at subsequent visits/contacts. All SAEs, and AEs of special interest (as defined in Section 8.6.7), will be followed until resolution, stabilization, the event is otherwise explained, or the participant is lost to follow-up (as defined in Section 7.4). Further information on follow-up procedures is provided in Appendix 9.3.

If a participant begins a new anticancer therapy, the adverse event reporting period for non-SAEs ends at the time the new treatment is started.

8.6.4. Regulatory Reporting Requirements for SAEs

Any SAEs occurring during this study must be reported as follows:

- Prompt notification by the investigator to the Sponsor of a SAE is essential so that legal obligations and ethical responsibilities towards the safety of participants and the safety of a study intervention under clinical investigation are met.
- The Sponsor has a legal responsibility to notify both the local regulatory authority and other regulatory agencies about the safety of a study intervention under clinical investigation. The Sponsor will comply with country-specific regulatory requirements relating to safety reporting to the regulatory authority, Institutional Review Boards (IRB)/Independent Ethics Committees (IEC), and investigators.
- For all studies except those utilizing medical devices investigator safety reports must be prepared for suspected unexpected serious adverse reactions (SUSAR) according to local regulatory requirements and Sponsor policy and forwarded to investigators as necessary.
- An investigator who receives an investigator safety report describing a SAE or other specific safety information (e.g., summary or listing of SAEs) from the Sponsor will review and then file it along with the Investigator's Brochure and will notify the IRB/IEC, if appropriate according to local requirements.
- The following describes the safety reporting requirements by timeline for reporting and associated type of event:
  - Within 7 Calendar Days

    Any study event that is:
    - Unexpected, fatal, or life-threatening suspected adverse reaction.
  - Within 15 Calendar Days

  Any study event that is:

    - Unexpected
    - Suspected adverse reaction that is serious, but not fatal or life-threatening

  -OR-

    - A previous adverse event that was not initially deemed reportable but is later found to fit the criteria for reporting (reporting within 15 calendar days from when event was deemed reportable).

  Any finding from tests in laboratory animals that:

    - Suggests a significant risk for human subject including reports of mutagenicity, teratogenicity, carcinogenicity, or reports of significant organ toxicity at or near the expected human exposure.

  Increase in rate of occurrence of serious suspected adverse reactions:

    - Any clinically important increase in the rate of a serious suspected adverse reaction over that listed in the protocol or Investigator Brochure.

If the adverse event does not meet expedited reporting requirements, the Sponsor will report the AE in the IND Annual Report.

8.6.5. Pregnancy

There is no relevant clinical experience with NeoTCR-P1 in pregnant or lactating women, and animal reproductive studies have not been performed. Women of child bearing potential must have a negative pregnancy test prior to enrollment because of the potentially dangerous effects of the preparative chemotherapy on the fetus. This experimental therapy should not be administered to pregnant women or women who are breastfeeding.

- Details of all pregnancies in female participants and female partners of male participants will be collected for pregnancies occurring after the start of study intervention until 6 months after completing the NeoTCR-P1 infusion or 5 months after the last dose of nivolumab (whichever comes last).
- If a pregnancy is reported, the investigator should inform the Sponsor within 24 hours of learning of the pregnancy and should follow the procedures outlined in Appendix 9.4.
- Abnormal pregnancy outcomes (e.g., spontaneous abortion, fetal death, stillbirth, congenital anomalies, ectopic pregnancy) are considered SAEs.
- Details of all lactation cases in female participants will be collected while taking protocol required therapies.
- If a lactation case is reported, the investigator should inform the Sponsor within 24 hours of learning of the lactation case and should follow the procedures outlined in Appendix 9.4.

In addition to reporting any pregnancies or lactation cases occurring during the study, investigators should monitor for pregnancies or lactation case that occur after the last dose of NeoTCR-P1 through 6 months for female participants and for 6 months for the female partner of male participants.

8.6.6. Death Events

All deaths occurring from the time of conditioning therapy to 90 days after the last dose of study drug, regardless of attribution will be recorded as an Adverse Event and expeditiously reported to the Sponsor. This includes death attributed to the progression of malignant disease.

When recording a death, the event or condition that caused or contributed to the fatal outcome should be recorded as the single medical condition on the Adverse Event *e*CRF. If the cause of death is unknown and cannot be ascertained at the time or reporting, then “unexplained death” should be recorded on the Adverse Event *e*CRF. If the death is attributed to progression of malignancy, “malignant neoplasm progression” should be recorded on the Adverse Event *e*CRF. The term “disease progression” should be avoided since it is not distinctly connected to a participant’s underlying malignancy.

8.6.7. Adverse Events of Special Interest

The following events are events of special interest and will need to be reported to the Sponsor expeditiously irrespective of regulatory seriousness criteria. Specific guidelines for protocol-defined events of special interest by NCI CTCAE v5.0 grade are provided below:

- Conditions suggestive of an autoimmune disorder including, but not limited to, hepatitis, pneumonitis, colitis, endocrinopathies, thyroiditis, arthritis, diabetes, vasculitis, neuritis, systemic lupus erythematosus, Sjogren's syndrome, multiple sclerosis, vitiligo, dermatitis, iritis
- Grade ≥3 acute infection (bacterial, viral, zoonotic, or fungal)
- Grade ≥3 events suggestive of hypersensitivity, CRS, HLH, febrile reactions or infusion related reactions, including but not limited to fever, chills, rash, urticaria, dyspnea, wheezing, angioedema, tachycardia, hypotension, and so forth
- Grade ≥2 hypoxia or dyspnea
- Grade ≥3 cytopenia lasting >28 days
- Grade ≥3 neurologic toxicity including but not limited to confusion, headache, difficulty speaking, mental status change, decreased level of consciousness and so forth

8.7. Pharmacokinetics

PBMCs from whole blood will be used for the measurement of NeoTCR-P1 as specified in the SOA (see Section 1.4).

Blood samples will be collected to evaluate the PK of fludarabine conditioning chemotherapy.

Instructions for the collection and handling of biological samples will be provided by the Sponsor in the *central* laboratory manual. The actual date and time (24-hour clock time) of each sample will be recorded.

Samples will be used to evaluate the PK of NeoTCR-P1. Samples collected for analyses of NeoTCR-P1 kinetics may also be used to evaluate safety or efficacy aspects related to concerns arising during or after the study.

8.8. Pharmacodynamics

Pharmacodynamic parameters will be evaluated in this study. The evaluation will include the analysis of the following cytokines: [redacted] Additional cytokines may be assessed on an exploratory basis.

8.9. Genetics

The analysis of NGS is an essential part of the study and required to manufacture the NeoTCR-P1 product. A blood and tissue sample for DNA isolation will be collected from participants. Participants who do not wish to participate in the genetic research may not have product manufactured.

See Appendix 9.5 for Information regarding genetic research. Details on processes for collection and shipment and destruction of these samples can be found in the *central* laboratory manual.

8.10. Biomarkers

Collection of samples for biomarker research will be performed as specified in the SoA.

Biomarker analyses may be performed on blood and tumor samples to evaluate predictive, pharmacokinetic, pharmacodynamic, and resistance markers for NeoTCR-P1.

Analysis may be performed on biomarker variants thought to play a role in the immune response including, but not limited to, NGS, genome-wide analysis for RNA, serum analytes, or tissue biomarkers to evaluate any association with observed clinical responses to NeoTCR-P1 or the combination of NeoTCR-P1 and nivolumab or IL-2.

The expansion and persistence of NeoTCR-P1 T cells will be monitored in the blood by flow cytometry and may be complemented by polymerase chain reaction (PCR) analysis. Exploratory analysis may include immunophenotyping to evaluate T-cell populations. In addition, PBMCs may be evaluated for changes in the TCR repertoire (i.e., epitope spreading) following the infusion of NeoTCR-P1.

Levels of circulating cytokines (see Section 8.8 for additional details) will also be evaluated in the blood at timepoints specified in SOA. Additional cytokines may be assessed on an exploratory basis.

CSF and additional samples may be harvested from participants who develop neurotoxicity to enable evaluation of inflammatory cytokine levels. As applicable, lymphocyte populations residing in the CSF, or other participant samples may also be monitored for the purpose of understanding the safety profile of NeoTCR-P1.

Bone marrow samples collected from participants who develop study treatment-emergent toxicities may be analyzed centrally by immunohistochemistry for evidence of disease, toxicity-related changes, and presence of NeoTCR-P1 T cells.

Ascites and pleural effusion samples may be collected from participants as part of standard of care and residual used for sample exploratory biomarker

In addition, baseline leukapheresis and samples of final manufactured NeoTCR-P1 products will be banked and may be analyzed by immunophenotyping, qPCR, and/or gene expression profiling. Remaining samples may be stored for future exploratory analysis of DNA, RNA, or protein markers.

Tumor tissue will be collected for central pathology review. Additional analysis may include but not limited to molecular analysis or IHC for PD-L1, CD8, MHC, and/or expression and viral (HPV/EBV) status.

On-study biopsies of tumor will be performed after NeoTCR-P1 infusion if safely accessible and clinically feasible. In addition, persisting, relapsing, or newly emergent lesions could be biopsied to help understand mechanisms of resistance.

Samples for biomarker research that should be collected from participants in the study *if clinically feasible* are the following:

- On-treatment tumor biopsy sample at progression/relapse

Samples may be used for research to develop methods, assays, prognostics, and/or companion diagnostic related to T-cell responses and the tumor microenvironment.

8.11. Immunogenicity Assessments

NeoTCR-P1 may elicit an immune response that could affect the prevalence of anti-nivolumab antibodies in the Phase 1b. Participants with signs of any potential immune response to nivolumab will be closely monitored.

Antibodies to nivolumab will be evaluated in serum samples collected from all participants in the Phase 1b according to the SOA. Additionally, serum samples should also be collected at the final visit from participants who discontinued study intervention or were withdrawn from the study. These samples will be tested by the Sponsor or Sponsor's designee.

Serum samples will be screened for antibodies binding to nivolumab and the titer of confirmed positive samples will be reported. Other analyses may be performed to verify the stability of antibodies to nivolumab and/or further characterize the immunogenicity of NeoTCR-P1.

The detection and characterization of antibodies to nivolumab will be performed using a validated assay method by or under the supervision of the Sponsor. Antibodies may be further characterized and/or evaluated for their ability to neutralize the activity of nivolumab.

8.12. Statistical Considerations and Hypotheses

No formal hypothesis will be tested in the Phase 1 study. Design considerations were not made with regard to explicit power and type I error considerations, but were made to obtain preliminary safety, feasibility, PK, PD, and antitumor activity information in this population.

The final analysis will be based on *participant* data collected through study discontinuation. The analyses will be based on the Full Analysis population (see Table 31 for further details). In general, data will be summarized as warranted, and listings will be used in place of tables when the sample sizes are small. All summaries will be presented by the assigned dose level, *treatment cohort,* or tumor type, when appropriate.

8.13. Sample Size Determination

Up to approximately 76 evaluable participants will be enrolled into the Initial Phase. The planned enrollment for the Expansion Phase study is potentially up to *112* participants, depending on the number and size of the cohorts. The total anticipated enrollment in this study is approximately *9–188* participants (see Table 16).

Three to 12 participants will be enrolled into each dose level cohort in the Phase 1a portion of the study. If the study proceeds to the dose-expansion basket cohorts in the Phase 1a, up to 40 additional participants may be enrolled (up to 20 each in the TCR alone and TCR + IL-2 baskets).

The dose-escalation stage sample size for this study is based on the dose-escalation rules described in Section 4.1.5.

Any participant who does not complete the DLT assessment window for any reason other than a DLT will be considered non-evaluable for dose-escalation decision and MTD assessment and will be replaced by an additional *participant* at that same dose level.

Table 30 describes the probability of not observing any DLTs in 3 participants, and the probability of observing fewer than 2 DLTs in 6 participants for underlying DLT rates during the dose-escalation stage.

Table 30 Probability of Observing DLTs for Different Underlying DLT Rates

Underlying DLT Rate	Probability of Observing No DLTs in 3 Participants	Probability of Observing Fewer than 2 DLTs in 6 Participants
0.10	0.73	0.89
0.20	0.51	0.66
0.33	0.30	0.36
0.40	0.22	0.23
0.50	0.13	0.11
0.60	0.06	0.04

DLT = dose-limiting toxicity.

To better characterize the safety of the single-agent and combination MTD identified in the dose-escalation stage, additional basket expansion cohorts of up to approximately 20 participants will be enrolled in the Phase 1a and Phase 1b. For a given adverse event with a true rate of 10%, 5%, or 1%, the probability of observing at least 1 such adverse event in a given cohort of 6 participants is 47%, 26% and 5.8% respectively. The corresponding probabilities of observing at least 1 such adverse event in the expanded cohort of 20 participants will increase to 87.8%, 64.2% and 18.2% respectively.

A key secondary activity endpoint of this study is overall response rate (i.e., either CR or PR) evaluated *up through* 6 months post infusion. With the proposed 20 evaluable confidence intervals (CIs) for various observed ORR will be as follows:

	N=10 Evaluable Participants	N=20 Evaluable Participants
Observed ORR	95% Exact CI (Clopper-Pearson)	
10%	0%–45%	1–32%
20%	3%–56%	6–44%
30%	7%–65%	12–54%
40%	12%–74%	19%–64%
50%	19%–81%	27%–73%
60%	26%–88%	36%–81%

CI = confidence intervals; ORR = objective response rate.

Based on the above table, if 10 out of 20 evaluable participants have a CR or PR at *any tumor assessment up through* 6 months, there will be strong evidence (i.e., 95% CI) that the true ORR following NeoTCR-P1 infusion is greater than or equal to an ORR of 27%, which would be considered clinically significant in this population.

Accrual to the initial portion of the study is anticipated to take approximately 12–15 months.

8.14. Populations for Analyses

The following populations are defined in Table 31.

Table 31 Definitions of Populations for Analysis

Population	Description
Feasibility-Evaluable	Comprises all participants who sign the ICF. The Feasibility-Evaluable Set will be used in the analysis of feasibility endpoints
Enrolled-Evaluable	Comprises all participants who sign the ICF and are enrolled in the study (meet eligibility criteria and commence leukapheresis). This Enrolled-Evaluable Set will be used in the analysis of feasibility endpoints.
DLT-Evaluable	Comprises all participants who receive the targeted dose and were followed for at least 28 days after the NeoTCR-P1 infusion or who received a dose of NeoTCR-P1 T cells *at target or* lower than the target for that cohort and experience a DLT during the 28-day post-infusion period. The DLT-Evaluable Set will be used for analysis of the primary safety endpoint.
Efficacy Evaluable	Comprises all participants who receive NeoTCR-P1 infusion at the intended dose range, with measurable disease at baseline and who complete the response assessments for the efficacy endpoint as planned by the protocol. Efficacy evaluable participants also include those with disease progression or death prior to the efficacy endpoint response assessment. The Efficacy Evaluable Set will be used for the analysis of efficacy endpoints
Full Analysis	Comprises all participants who receive any dose of NeoTCR-P1. This set includes the efficacy evaluable and non-evaluable participants. The Full Analysis Set will used for the primary analysis of safety, PK/PD, efficacy, and other exploratory endpoints

DLT = dose-limiting toxicity; ICF = informed consent form; PD = pharmacodynamic; PK =pharmacokinetic.

The efficacy non-evaluable set includes any participant who is infused with NeoTCR-P1 at less than the protocol-specified dose and/or who does not have measurable disease at baseline.

8.15. Statistical Analyses

The Statistical Analysis Plan will be finalized prior to database lock and it will include a more technical and detailed description of the statistical analyses described in this section. This section is a summary of the planned statistical analyses of the most important endpoints including primary and key secondary endpoints.

8.15.1. General Considerations

Descriptive statistics will be computed for all study variables for the evaluable populations as whole and within histology subgroups.

8.15.2. Primary Endpoint(s)

- Incidence and nature of AEs defined as DLTs, and AEs according to NCI CTCAE v5.0 with the exception of CRS and neurotoxicity, which will use the ASTCT consensus criteria.
- All AEs will be described, and exact 95% CI will be produced for AE rates, both overall and within major categories.
- Number of manufactured products that do not meet release criteria for transfection efficiency, T-cell viability, and sterility will be determined.
- The number of *participants* enrolled versus the number of *participants* infused will be described as a measure of the feasibility of this therapy.

8.15.3. Secondary Endpoint(s)

Key secondary endpoints are described below.

8.15.3.1. Activity Analysis

ORR is defined as a CR *plus* PR, as determined by investigator assessment using RECIST v1.1 and confirmed by repeat assessment ≥4 weeks after initial documentation. Participants with missing or no response assessments post-baseline will be classified as non-responders. ORR will be estimated and summarized by tumor type*, treatment cohort,* and by dose, if applicable.

Participants with missing baseline assessments or without measurable disease at baseline will not be evaluable for ORR but may be evaluable for progression-free survival.

Overall response will be summarized, and a 95% CI will be calculated based on binomial distribution.

Duration of response is defined as the time from the initial CR or PR to the time of disease progression or death from any cause, whichever occurs first. For participants who do not experience disease progression or do not die before the end of the study or who are lost to follow-up, duration of objective response will be censored at the day of the last *evaluable* tumor assessment.

Progression free survival (PFS) is defined as the time from the first day of study treatment with NeoTCR-P1 until documented disease progression or death, whichever occurs first. For participants who do not have documented progressive disease or death before the end of the study or who are lost to follow-up, PFS will be censored at the date of the last *evaluable* tumor assessment.

OS is defined as the time from the first day of study treatment with NeoTCR-P1 until death. For participants who do not die before the end of the study or who are lost to follow-up, OS will be censored at the date of last contact.

For the evaluation of PFS and OS, Kaplan-Meier methodology will be used to estimate the median OS and to construct survival curves. Median survival time and survival probability at selected timepoints (e.g., 6 months) will be computed with associated CIs.

8.15.3.2. Safety Analysis

In addition, safety will be assessed through the summaries of changes in laboratory test results, changes in vital signs, and ECGs, exposure to NeoTCR-P1 and exposure to nivolumab (Phase 1b). All participants who receive any amount of NeoTCR-P1 will be included in the safety analysis.

Verbatim descriptions of adverse events will be mapped to thesaurus terms. Adverse event data will be listed by study site, dose cohort, treatment arm, *participant* number, and study day. Events occurring on or after treatment on Day 0 will be summarized by mapped term, appropriate thesaurus levels, and NCI CTCAE v5.0 grade. In addition, SAEs, including deaths, will be listed separately and summarized.

Adverse events leading to treatment discontinuation will be listed. Adverse events leading the declaration of DLTs will be listed. Participants who withdraw from the study prior to completing the DLT assessment window (i.e., Day 28) for reasons other than a DLT will be considered unevaluable for DLT and MTD assessments.

Relevant laboratory and vital sign data will be de displayed by time, with NCI CTCAE Grade 3 and 4 values identified where appropriate. Additionally, all laboratory data will be summarized by grade with use of NCI CTCAE v5.0.

8.15.3.3. Immunogenicity Analysis

- Incidence of anti-nivolumab antibodies

8.15.3.4. PK/PD Analysis

- Levels of NeoTCR-P1-positive T cells in blood and levels of circulating cytokines will be summarized

8.15.4. Exploratory Endpoint(s)

Investigation of potential biomarker development based on assessment of blood cells, tumor cells, and the proposed action of the investigational product.

8.16. Interim Analyses

8.16.1. Interim Analysis and Early Stopping Rules

The SRT will be chartered to review safety during the dose-escalation portions of the Phase 1a and Phase 1b portions of the study, progression to the dose-expansion basket cohorts, and to make recommendations on study conduct in Phase 1.

The *SRT* will also monitor criteria to pause enrollment (see Section 8.17). It is expected that AEs will occur frequently in this population based on the underlying advance malignancy and that these can be SAEs. Therefore, there is no specific occurrence of SAEs that define a stopping rule, but the review of SAEs will form the basis for potential early stopping of the study.

The study will be stopped if:

- Any participant develops uncontrolled T-cell proliferation that does not respond to management
- The Sponsor decides to discontinue the development of the intervention to be used in this study

See Section 8.17 for additional criteria to pause enrollment.

8.16.2. Efficacy Interim Analyses

Within the Phase 1, 2 interim, and 1 primary analysis will be performed:

- Interim analysis 1 will be conducted after 10 participants have had the opportunity to be evaluated for response approximately 1 month after the NeoTCR-P1 infusion.
- Interim analysis 2 will be conducted after 20 participants have had the opportunity to be evaluated for response approximately 2 months after the NeoTCR-P1 infusion (minimum of approximately 2 tumor assessments per participant).
- The primary analysis of Phase 1 will occur after all participants in the Full Analysis Set have had the opportunity to be assessed for response 6 months after the NeoTCR-P1 infusion.

Accrual to the study will continue during the interim analysis.

Within each of the indication-specific cohorts, the following rule will apply: if no responders (CR or PR) are observed from the first 10 participants who are considered to be more likely to respond on the basis of the presence of biomarkers potentially predictive of anti-tumor activity (e.g., PD-L1 IHC, tumor mutational burden), enrollment will be suspended for that indication.

The Statistical Analysis Plan will describe the planned interim analyses in greater detail.

8.17. Criteria to Pause Enrollment

As part of its oversight of the study, the SRT will assess criteria to pause enrollment.

Enrollment will be paused if any of the following criteria are met:

- Any Grade 5 NeoTCR-P1-possibly, probably, or definitely related adverse events within 30 days.
- If the incidence of NeoTCR-P1-related DLTs is >33% of the following:
  - Neurotoxicity
  - CRS (per ASTCT criteria)
  - Other non-hematological SAE
  - Infection (treatment-related)
- Manufacturing process fails to meet the minimum protocol-specified dose (i.e., 1.6×10^7 neoTCR-positive cells) range in ≥33% of study participants.

If the study is paused for safety (i.e., 1 and 2 above), the SRT and members of the study team will meet within 24 hours of the event to discuss the event. Site investigators will be consulted as necessary for case details and management. If all parties are in agreement as to the event resolution, then the pause will be lifted.

If the study is paused for manufacturing feasibility (i.e., third bullet above), members of the study team, clinical operations, and Chemistry, Manufacturing, and Controls will meet to identify manufacturing or dose failures. The team will make recommendations for process improvements to be implemented. Pending successful completion of a process validation run, the manufacturing pause will be lifted.

8.18. Study Sample Storage and Retention

After the *participant* has signed the study informed consent, samples will be collected and tested as outlined in the study assessment and procedure section of the protocol. Study samples and any other components from these samples may be stored up to 15 years (or in accordance to local regulations) following the last *participant's* last visit to address exploratory research scientific questions related to the treatment or disease under study. Each participant will have the right to have the sample material destroyed at any time by contacting the investigator. The investigator should provide to the Sponsor the study and participant number consent withdraw notice so that the sample can be destroyed.

For participants who withdraw consent, any data collected up to that time will remain in the study database. Any samples that were not requested or required to be returned or destroyed will remain with the Sponsor and any data that may be generated will be entered into the study database.

Additional information about sample collected and storage will be outlined in the study *central* laboratory manual and sites informed consent.

9. Supporting Documentation and Operational Considerations

9.1. Appendix 1. Regulatory, Ethical, and Study Oversight Considerations

9.1.1. Regulatory and Ethical Considerations

- This study will be conducted in accordance with the protocol and with the following:
  - Consensus ethical principles derived from international guidelines including the Declaration of Helsinki and Council for International Organizations of Medical Sciences (CIOMS) International Ethical Guidelines
  - Applicable ICH E6 Good Clinical Practice (GCP) Guidelines
  - Applicable laws and regulations
- The protocol, protocol amendments, ICF, Investigator Brochure, and other relevant documents (e.g., advertisements) must be submitted to an IRB/IEC by the investigator and reviewed and approved by the IRB/IEC before the study is initiated.
- Any amendments to the protocol will require IRB/IEC approval before implementation of changes made to the study design, except for changes necessary to eliminate an immediate hazard to study participants.
- The investigator will be responsible for the following:
  - Providing written summaries of the status of the study to the IRB/IEC annually or more frequently in accordance with the requirements, policies, and procedures established by the IRB/IEC
  - Notifying the IRB/IEC of SAEs or other significant safety findings as required by IRB/IEC procedures
  - Providing oversight of the conduct of the study at the site and adherence to requirements of 21 CFR, ICH guidelines, the IRB/IEC, European regulation 536/2014 for clinical studies (if applicable), and all other applicable local regulations

9.1.2. Financial Disclosure

Investigators and sub-investigators will provide the Sponsor with sufficient, accurate financial information as requested to allow the Sponsor to submit complete and accurate financial certification or disclosure statements to the appropriate regulatory authorities. Investigators are responsible for providing information on financial interests during the course of the study and for 1 year after completion of the study.

9.1.3. Informed Consent Process

PACT Pharma's sample ICF will be provided to each site. PACT Pharma or its designee must review and approve any proposed deviations from the sample ICF template or any alternate

consent forms proposed by the site before the IRB/EC submission. The final IRB/EC-approved consent forms must be provided to PACT Pharma for regulatory purposes.

- The investigator or his/her representative will explain the nature of the study to the participant or his/her legally authorized representative and answer all questions regarding the study.
- Participants must be informed that their participation is voluntary. Participants or their legally authorized representative (see Section 8.1) will be required to sign a statement of informed consent that meets the requirements of 21 CFR 50, local regulations, ICH guidelines, Health Insurance Portability and Accountability Act (HIPAA) requirements, where applicable, and the IRB/IEC or study center.
- The medical record must include a statement that written informed consent was obtained before the participant was enrolled in the study and the date the written consent was obtained. The authorized person obtaining the informed consent must also sign the ICF.
- Participants must be re-consented to the most current version of the ICF(s) during their participation in the study.
- A copy of the ICF(s) must be provided to the participant or the participant's legally authorized representative.

9.1.4. Data Protection

- Participants will be assigned a unique identifier by the Sponsor. Any participant records or datasets that are transferred to the Sponsor will contain the identifier only.
- Participant names or any information that would make the participant identifiable will not be transferred.
- The participant must be informed that his/her personal study-related data will be used by the Sponsor in accordance with local data protection law. The level of disclosure must also be explained to the participant who will be required to give consent for their data to be used as described in the informed consent.
- The participant must be informed that his/her medical records may be examined by Clinical Quality Assurance auditors or other authorized personnel appointed by the Sponsor, by appropriate IRB/IEC members, and by inspectors from regulatory authorities.

9.1.5. Administrative Structure

This study is Sponsored by PACT Pharma. *Participant* dose assignment and drug supply will be managed manually. Data will be recorded via an electronic data capture (EDC) system from Medidata Solutions, Inc. (New York, NY) with the use of eCRF. Central laboratories will coordinate the collections of archival and newly collected tumor tissue and of blood samples for the assessment of PK, PD, and predictive biomarkers.

9.1.6. Dissemination of Clinical Study Data

9.1.6.1. Publication Policy

The Sponsor will comply with the requirements for publication of study results. In accordance with standard editorial and ethical practice, the Sponsor will generally support publication of multicenter studies only in their entirety and not as individual site data. In this case, a coordinating investigator will be designated by mutual agreement.

Authorship of publications from data generated in study PACT-0101 will be determined based on the uniform requirements for manuscripts submitted to biomedical journals (as outlined in the International Committee of Medical Journal editors December 2013), which states that authorship should be based on the following 4 criteria:

- Substantial contributions to the conception or design of the work
- Or the acquisition, analysis, or interpretation of data for the work

AND

- Drafting the work or revising it critically for important intellectual content

AND

- Final approval of the version to be published

AND

- Agreement to be accountable for all aspects of the work in ensuring that questions related to the accuracy or integrity of any part of the work are appropriately investigated and resolved.

In addition to being accountable for the parts of the work he or she has done, an author should be able to identify which co-authors are responsible for specific other parts of the work. In addition, authors should have confidence in the integrity of the contributions of their co-authors.

All those designated as authors should meet all 4 criteria for authorship, and all who meet the 4 criteria should be identified as authors. Those who do not meet all 4 criteria should be acknowledged.

Funding, collection of data, or general supervision of the research alone or in combination does not qualify an individual for authorship.

Any publication, in any form, that is derived from this study must be submitted to PACT Pharma for review and approval. The study contract between the institution, principal investigation and PACT Pharma or its delegate will outline the requirement for publication review.

9.1.7. Data Quality Assurance

- All participant data relating to the study will be recorded on printed or electronic *e*CRF unless transmitted to the Sponsor or designee electronically (e.g., laboratory data). The

investigator is responsible for verifying that data entries are accurate and correct by physically or electronically signing the *e*CRF.

- The investigator must maintain accurate documentation (source data) that supports the information entered in the *e*CRF.
- The investigator must permit study-related monitoring, audits, IRB/IEC review, and regulatory agency inspections and provide direct access to source data documents.
- Monitoring details describing strategy (e.g., risk-based initiatives in operations and quality such as Risk Management and Mitigation Strategies and Analytical Risk-Based Monitoring), methods, responsibilities and requirements, including handling of noncompliance issues and monitoring techniques (central, remote, or on-site monitoring) are provided in the Monitoring Plan.
- The Sponsor or designee is responsible for the data management of this study including quality checking of the data.
- The Sponsor assumes accountability for actions delegated to other individuals (e.g., Contract Research Organizations).
- Study monitors will perform ongoing source data verification to confirm that data entered into the *e*CRF by authorized site personnel are accurate, complete, and verifiable from source documents.
- That the safety and rights of participants are being protected
- And that the study is being conducted in accordance with the currently approved protocol and any other study agreements, ICH GCP, and all applicable regulatory requirements.
- Records and documents, including signed ICFs, pertaining to the conduct of this study must be retained by the investigator for a period of 2 years following the date a marketing application is approved for the drug for the indication for which it is being investigated; or, if no application is to be filed or if the application is not approved for such indication, until 2 years after the investigation is discontinued and FDA is notified unless local regulations or institutional policies require a longer retention period. No records may be destroyed during the retention period without the written approval of the Sponsor. No records may be transferred to another location or party without written notification to the Sponsor.

9.1.8. Source Documents

- Source documents provide evidence for the existence of the participant and substantiate the integrity of the data collected. Source documents are filed at the investigator's site.
- Data reported on the *e*CRF or entered in the eCRF that are transcribed from source documents must be consistent with the source documents or the discrepancies must be explained. The investigator may need to request previous medical records or transfer records, depending on the study. Also, current medical records must be available.
- Definition of what constitutes source data can be found in the study manual.

9.1.9. Study and Site Start and Closure

The study start date is the date on which the clinical study will be open for recruitment of participants.

The first act of recruitment is the first site open and will be the study start date.

The Sponsor designee reserves the right to close the study site or terminate the study at any time for any reason at the sole discretion of the Sponsor. Study sites will be closed upon study completion. A study site is considered closed when all required documents and study supplies have been collected and a study-site closure visit has been performed.

The investigator may initiate study-site closure at any time, provided there is reasonable cause and sufficient notice is given in advance of the intended termination.

Reasons for the early closure of a study site by the Sponsor or investigator may include but are not limited to:

- Failure of the investigator to comply with the protocol, the requirements of the IRB/IEC or local health authorities, the Sponsor's procedures, or GCP guidelines
- Inadequate recruitment of participants by the investigator
- Discontinuation of further study intervention development

If the study is prematurely terminated or suspended, the Sponsor shall promptly inform the Investigators, the IECs/IRBs, the regulatory authorities, and any contract research organization(s) used in the study of the reason for termination or suspension, as specified by the applicable regulatory requirements. The Investigator shall promptly inform the participant and should assure appropriate participant therapy and/or follow-up.

9.2. Appendix 2. Preexisting Autoimmune Diseases

Patients should be carefully questioned regarding their history of acquired or congenital autoimmune disease. Patients with a history of autoimmune disease *listed below* are excluded from the study. *Other autoimmune diseases not listed below may also be exclusionary if requiring management with chronic steroids (>5 mg/day prednisone or dose equivalent).*

Possible exceptions to this exclusion *after discussion with the Medical Monitor* could be patients with a medical history of such entities as atopic disease or childhood arthralgias where the clinical suspicion of autoimmune disease is low. *In addition, patients* with a history of autoimmune-mediated hypothyroidism *or Type 1 diabetes, asymptomatic lichen planus, and vitiligo mellitus* may be eligible for this study.

- *Achalasia*
- *Addison's disease*
- *Adult Still's disease*
- *Agammaglobulinemia*
- *Alopecia areata*
- *Goodpasture's syndrome*
- *Granulomatosis with Polyangiitis*
- *Graves' disease*
- *Guillain-Barre syndrome*
- *Polyglandular syndromes type I, II, III*
- *Polymyalgia rheumatica*
- *Polymyositis*
- *Postmyocardial infarction syndrome*

- *Amyloidosis*
- *Ankylosing spondylitis*
- *Anti-GBM/Anti-TBM nephritis*
- *Antiphospholipid syndrome*
- *Autoimmune angioedema*
- *Autoimmune dysautonomia*
- *Autoimmune encephalomyelitis*
- *Autoimmune hepatitis*
- *Autoimmune inner ear disease (AIED)*
- *Autoimmune myocarditis*
- *Autoimmune oophoritis*
- *Autoimmune orchitis*
- *Autoimmune pancreatitis*
- *Autoimmune retinopathy*
- *Autoimmune urticaria*
- *Axonal & neuronal neuropathy (AMAN)*
- *Baló disease*
- *Behcet's disease*
- *Benign mucosal pemphigoid*
- *Bullous pemphigoid*
- *Castleman disease (CD)*
- *Celiac disease*
- *Chagas disease*
- *Chronic inflammatory demyelinating polyneuropathy (CIDP)*
- *Chronic recurrent multifocal osteomyelitis (CRMO)*
- *Churg-Strauss Syndrome (CSS) or Eosinophilic Granulomatosis (EGPA)*
- *Cicatricial pemphigoid*
- *Cogan's syndrome*
- *Cold agglutinin disease*
- *Congenital heart block*
- *Coxsackie myocarditis*
- *CREST syndrome*
- *Crohn's disease*
- *Dermatitis herpetiformis*
- *Dermatomyositis*
- *Devic's disease (neuromyelitis optica)*
- *Hashimoto's thyroiditis*
- *Hemolytic anemia*
- *Henoch-Schonlein purpura (HSP)*
- *Herpes gestationis or pemphigoid gestationis (PG)*
- *Hidradenitis Suppurativa (HS) (Acne Inversa)*
- *Hypogammalglobulinemia*
- *IgA Nephropathy*
- *IgG4-related sclerosing disease*
- *Immune thrombocytopenic purpura (ITP)*
- *Inclusion body myositis (IBM)*
- *Interstitial cystitis (IC)*
- *Juvenile arthritis*
- *Juvenile diabetes (Type 1 diabetes)*
- *Juvenile myositis (JM)*
- *Kawasaki disease*
- *Lambert-Eaton syndrome*
- *Leukocytoclastic vasculitis*
- *Lichen planus*
- *Lichen sclerosus*
- *Ligneous conjunctivitis*
- *Linear IgA disease (LAD)*
- *Lupus*
- *Lyme disease chronic*
- *Meniere's disease*
- *Microscopic polyangiitis (MPA)*
- *Mixed connective tissue disease (MCTD)*
- *Mooren's ulcer*
- *Mucha-Habermann disease*
- *Multifocal Motor Neuropathy (MMN) or MMNCB*
- *Multiple sclerosis*
- *Myasthenia gravis*
- *Myositis*
- *Narcolepsy*
- *Neonatal Lupus*
- *Neuromyelitis optica*
- *Neutropenia*
- *Ocular cicatricial pemphigoid*
- *Postpericardiotomy syndrome*
- *Primary biliary cirrhosis*
- *Primary sclerosing cholangitis*
- *Progesterone dermatitis*
- *Psoriasis*
- *Psoriatic arthritis*
- *Pure red cell aplasia (PRCA)*
- *Pyoderma gangrenosum*
- *Raynaud's phenomenon*
- *Reactive arthritis*
- *Reflex sympathetic dystrophy*
- *Relapsing polychondritis*
- *Restless legs syndrome (RLS)*
- *Retroperitoneal fibrosis*
- *Rheumatic fever*
- *Rheumatoid arthritis*
- *Sarcoidosis*
- *Schmidt syndrome*
- *Scleritis*
- *Scleroderma*
- *Sjögren's syndrome*
- *Sperm & testicular autoimmunity*
- *Stiff person syndrome (SPS)*
- *Subacute bacterial endocarditis (SBE)*
- *Susac's syndrome*
- *Sympathetic ophthalmia (SO)*
- *Takayasu's arteritis*
- *Temporal arteritis/Giant cell arteritis*
- *Thrombocytopenic purpura (TTP)*
- *Thyroid eye disease (TED)*
- *Tolosa-Hunt syndrome (THS)*
- *Transverse myelitis*
- *Type 1 diabetes*
- *Ulcerative colitis (UC)*
- *Undifferentiated connective tissue disease (UCTD)*
- *Uveitis*
- *Vasculitis*
- *Vitiligo*

- *Discoid lupus*
- *Dressler's syndrome*
- *Endometriosis*
- *Eosinophilic esophagitis*
- *Eosinophilic fasciitis*
- *Erythema nodosum*
- *Essential mixed cryoglobulinemia*
- *Evans syndrome*
- *Fibromyalgia*
- *Fibrosing alveolitis*
- *Giant cell arteritis (temporal arteritis)*
- *Giant cell myocarditis*
- *Glomerulonephritis*
- *Optic neuritis*
- *Palindromic rheumatism (PR)*
- *Paraneoplastic cerebellar degeneration (PCD)*
- *Paroxysmal nocturnal hemoglobinuria (PNH)*
- *Parry Romberg syndrome*
- *Pars planitis (peripheral uveitis)*
- *Parsonage-Turner syndrome*
- *Pemphigus*
- *Peripheral neuropathy*
- *Perivenous encephalomyelitis*
- *Pernicious anemia (PA)*
- *POEMS syndrome*
- *Polyarteritis nodosa*
- *Vogt-Koyanagi-Harada Disease*

9.3. Appendix 3. Adverse Events. Definitions and Procedures for Recording, Evaluating, Follow-up, and Reporting

9.3.1. Definition of *Adverse Event* (AE)

**AE Definition**
• An *adverse event* (AE) is any untoward medical occurrence in a patient or clinical study participant, temporally associated with the use of study intervention, whether or not considered related to the study intervention.<br>• NOTE. An AE can therefore be any unfavorable and unintended sign (including an abnormal laboratory finding), symptom, or disease (new or exacerbated) temporally associated with the use of study intervention.<br>The investigator is responsible for ensuring that any adverse events observed by the investigator or reported by the participant are recorded in the participant's medical record.

**Events Meeting the AE Definition**
• Any abnormal laboratory test results (hematology, clinical chemistry, or urinalysis) or other safety assessments (e.g., *electrocardiogram* [ECG], radiological scans, vital signs measurements), including those that worsen from baseline, considered clinically significant in the medical and scientific judgment of the investigator (i.e., not related to progression of underlying disease). **In general, abnormal laboratory findings without clinical significance (based on the investigator's judgment) are not to be recorded as adverse events. However, abnormal laboratory findings that result in new or worsening clinical sequelae, require therapy, or adjustment in current therapy are considered adverse events. Where applicable, clinical sequelae (not the laboratory abnormality) are to be recorded as the adverse event.** If the clinically significant laboratory abnormality is a sign of a disease or syndrome (e.g., alkaline phosphatase and bilirubin 5 × >*upper limit of normal* (ULN) associated with cholecystitis) only the diagnosis (e.g., cholecystitis) needs to be recorded on the Adverse Event *electronic Case Report Form* (eCRF).<br>• Exacerbation of a chronic or intermittent pre-existing condition including either an increase in frequency and/or intensity of the condition.<br>• New conditions detected or diagnosed after study intervention administration even though it may have been present before the start of the study.<br>• Signs, symptoms, or the clinical sequelae of a suspected drug-drug interaction.<br>• Signs, symptoms, or the clinical sequelae of a suspected overdose of either study intervention or a concomitant medication. Overdose *per se* will not be reported as an AE/*serious adverse event* (SAE) unless it is an intentional overdose taken with possible suicidal/self-harming intent. Such overdoses should be reported regardless of sequelae.<br>Lack of efficacy will not be reported as an AE or SAE. Such instances will be captured in the efficacy assessments. Nonetheless, the signs, symptoms, and/or clinical sequelae resulting from lack of efficacy will be reported as an AE or SAE if they fulfill the definition of an AE or SAE.<br>The term "disease progression" or "progressive disease" should not be reported as an AE. Death due to disease progression in the absence of signs and symptoms should be reported as the primary tumor type (e.g., melanoma, ovarian cancer)<br>For situations when an adverse event is due to the disease under investigation report the signs and symptoms.

**Events NOT Meeting the AE Definition**
• Any clinically significant abnormal laboratory findings or other abnormal safety assessments that are associated with the underlying disease, unless judged by the investigator to be more severe than expected for the participant's condition.<br>• The disease/disorder being studied or expected progression, signs, or symptoms of the disease/disorder being studied, unless more severe than expected for the participant's condition.<br>• Interventions for pretreatment conditions (e.g., elective cosmetic surgery) or medical procedures that were planned before study participation (e.g., port placement) are not considered an AE.<br>• For unplanned, medical or surgical procedure (e.g., endoscopy, appendectomy). The condition that leads to the procedure is the AE.<br>• Situations in which an untoward medical occurrence did not occur (social, logistical, and/or convenience admission to a hospital).<br>• Anticipated day-to-day fluctuations of pre-existing disease(s) or condition(s) present or detected at the start of the study that do not worsen.

9.3.2. Definition of SAE

If an event is not an AE per definition above, then it cannot be an SAE even if serious conditions are met (e.g., hospitalization for signs/symptoms of the disease under study, death due to progression of disease).

**A SAE is defined as any untoward medical occurrence that, at any dose:**
**a. Results in death**
**b. Is life-threatening**<br>The term "life-threatening" in the definition of "serious" refers to an event in which the participant was at risk of death at the time of the event. It does not refer to an event that hypothetically might have caused death, if it were more severe.
**c. Requires inpatient hospitalization or prolongation of existing hospitalization**<br>• In general, hospitalization signifies that the participant has been detained (usually involving at least an overnight stay) at the hospital or emergency ward for observation and/or treatment that would not have been appropriate in the physician's office or outpatient setting. Complications that occur during hospitalization are AEs. If a complication prolongs hospitalization or fulfills any other serious criteria, the event is serious. When in doubt as to whether "hospitalization" occurred or was necessary, the AE should be considered serious.<br>• Events that require an escalation of care when the patient is already hospitalized should be recorded as a SAE. Examples of such event include movement from routine care in the hospital to the *intensive care unit* (ICU) or if that event resulted in a prolongation of the existing planned hospitalization.<br>• Hospitalization for conditioning chemotherapy, infusion, and monitoring as required by the protocol and elective treatment of a pre-existing condition that did not worsen from baseline is not considered an AE.<br>• Planned admissions to hospital for administration of conditioning chemotherapy, NeoTCR-P1, nivolumab, IL-2, or other protocol-mandated procedures do not qualify as serious unless a new AE occurs that results in prolongation or meets any of the other "serious" criteria.

**d. Results in persistent disability/incapacity**<br>• The term disability means a substantial disruption of a person's ability to conduct normal life functions.<br>• This definition is not intended to include experiences of relatively minor medical significance such as uncomplicated headache, nausea, vomiting, diarrhea, influenza, and accidental trauma (e.g., sprained ankle) that may interfere with or prevent everyday life functions but do not constitute a substantial disruption.
**e. Is a congenital anomaly/birth defect**
**f. Other situations:**<br>• Medical or scientific judgment should be exercised in deciding whether SAE reporting is appropriate in other situations such as important medical events that may not be immediately life-threatening or result in death or hospitalization but may jeopardize the participant or may require medical or surgical intervention to prevent 1 of the other outcomes listed in the above definition. These events should usually be considered serious.<br>• Examples of such events include invasive or malignant cancers, intensive treatment in an emergency room or at home for allergic bronchospasm, blood dyscrasias, or convulsions that do not result in hospitalization, or development of drug dependency or drug abuse.

9.3.3. Recording and Follow-Up of AE and/or SAE

**AE and SAE Recording**
• When an AE/SAE occurs, it is the responsibility of the investigator to review all d When an AE/SAE occurs, it is the responsibility of the investigator to review all documentation (e.g., hospital progress notes, laboratory reports, and diagnostics reports) related to the event.<br>• The investigator will then record all relevant AE/SAE information in the *e*CRF.<br>• It is not acceptable for the investigator to send photocopies of the participant's medical records to the Sponsor in lieu of completion of the AE/SAE *e*CRF page.<br>• There may be instances when copies of medical records for certain cases are requested by Sponsor. In this case, all participant identifiers, with the exception of the participant number, will be redacted on the copies of the medical records before submission to Sponsor.<br>• The investigator will attempt to establish a diagnosis of the event based on signs, symptoms, and/or other clinical information. Whenever possible, the diagnosis (not the individual signs/symptoms) will be documented as the AE/SAE (e.g., record liver failure or hepatitis rather than jaundice, asterixis, and elevated transaminases). In addition, adverse events occurring secondary to other events (e.g., cascade events or clinical sequelae) should be identified by their primary cause (e.g., if diarrhea is known to have resulted in dehydration, then it is sufficient to record only diarrhea as an adverse event on the eCRF).
**Assessment of Intensity**
Adverse event grading scale used will be the *National Cancer Institute Common Terminology Criteria for Adverse Events* version 5.0 (NCI CTCAE v5.0) with the exceptions of *cytokine release syndrome* (CRS) and neurotoxicity. A copy of the grading scale can be downloaded from the CTEP web site (https://ctep.cancer.gov/protocolDevelopment/electronic_applications/ctc.htm#ctc_50). Cytokine release syndrome and neurotoxicity events will be reported using the *Cytokine Release Syndrome*

Consensus Grading Scale (ASTCT) Consensus Grading scale as outlined in Table 21 of Section 6.11.3 and Table 23 of Section 6.11.7.

**Assessment of Causality**
• The investigator is obligated to assess the relationship between study intervention (Flu/Cy, NeoTCR-P1, IL-2, and/or nivolumab) and each occurrence of each AE/SAE.<br>• A "reasonable possibility" of a relationship conveys that there are facts, evidence, and/or arguments to suggest a causal relationship, rather than a relationship cannot be ruled out.<br>• The investigator will use clinical judgment to determine the relationship.<br>• Alternative causes, such as underlying disease(s), concomitant therapy, and other risk factors, as well as the temporal relationship of the event to study intervention administration will be considered and investigated.<br>• The investigator will also consult the Investigator's Brochure and/or Product Information, for marketed products, in his/her assessment.<br>• For each AE/SAE, the investigator must document in the medical notes that he/she has reviewed the AE/SAE and has provided an assessment of causality.<br>• There may be situations in which an SAE has occurred, and the investigator has minimal information to include in the initial report to the Sponsor's Drug Safety Group. However, it is very important that the investigator always make an assessment of causality for every event before the initial transmission of the SAE data to the Sponsor's Drug Safety Group or designees.<br>• The investigator may change his/her opinion of causality in light of follow-up information and send a SAE follow-up report with the updated causality assessment.<br>• The causality assessment is 1 of the criteria used when determining regulatory reporting requirements.<br>In reviewing adverse events, investigators must assess whether the adverse event is possibly related to the 1) NeoTCR-P1, 2) nivolumab, 3) conditioning chemotherapy (fludarabine, cyclophosphamide), 4) IL-2, or 5) any protocol required study procedure (e.g., leukapheresis, biopsy). The relationship is indicated by a yes or no response and entered in the *e*CRF. A yes response should indicate that there is evidence to suggest a causal relationship between the study treatment or procedure and the adverse event.

**Follow-up of AEs and SAEs**
• The investigator is obligated to perform or arrange for the conduct of supplemental measurements and/or evaluations as medically indicated or as requested by the Sponsor's Drug Safety Group or designee to elucidate the nature and/or causality of the AE or SAE as fully as possible. This may include additional laboratory tests or investigations, histopathological examinations, or consultation with other health care professionals.<br>• If a participant dies during participation in the study or during a recognized follow-up period, the investigator will provide Sponsor with a copy of any post-mortem findings including histopathology.<br>• The investigator will submit any updated SAE data to Sponsor within 24 hours of receipt of the information.

9.3.4. Reporting of SAEs and Adverse Events of Special Interest (AESI)s

SAE Reporting to Sponsor via the SAE/AESI Report Form
The investigator is responsible for ensuring that adverse events are monitored and reported. See Section 8.6.1 for greater details regarding the AE and SAE reporting periods.<br>• Report SAEs by completing the paper SAE/AESI Report Form (see next section) in order to report the event within 24 hours.<br>• Contacts for SAE reporting can be found in the study manual.<br>• In rare circumstances and in the absence of email or facsimile equipment, notification by telephone is acceptable with a copy of the SAE/AESI data collection tool sent by overnight mail or courier service.<br>• Initial notification via telephone does not replace the need for the investigator to complete and sign the SAE/AESI Report Form within the designated reporting time frames.

9.4. Appendix 4. Contraceptive Guidance and Collection of Pregnancy Information

Definitions:

Woman of Childbearing Potential (WOCBP)

A woman is considered fertile following menarche and until becoming post-menopausal unless permanently sterile (see below).

If fertility is unclear (e.g., amenorrhea in adolescents or athletes) and a menstrual cycle cannot be confirmed before first dose of study intervention, additional evaluation should be considered.

Women in the following categories are not considered WOCBP:

- Premenarchal
- Premenopausal female with 1 of the following:
  - Documented hysterectomy
  - Documented bilateral salpingectomy
  - Documented bilateral oophorectomy
  - For individuals with permanent infertility due to an alternate medical cause other than the above, (e.g., Müllerian agenesis, androgen insensitivity), investigator discretion should be applied to determining study entry.

  Note. Documentation can come from the site personnel's review of the participant's medical records, medical examination, or medical history interview.

- Postmenopausal female
  - A postmenopausal state is defined as no menses for 12 months without an alternative medical cause.
    - A high follicle stimulating hormone (FSH) level in the postmenopausal range may be used to confirm a postmenopausal state in women not using hormonal contraception or hormonal replacement therapy (HRT). However, in the absence

of 12 months of amenorrhea, confirmation with more than 1 FSH measurement is required.

- Females on HRT and whose menopausal status is in doubt will be required to use 1 of the non-estrogen hormonal highly effective contraception methods if they wish to continue their HRT during the study. Otherwise, they must discontinue HRT to allow confirmation of postmenopausal status before study enrollment.

Contraception Guidance:

Collection of Pregnancy Information

Male Participants With Partners who Become Pregnant

- The investigator will attempt to collect pregnancy information on any male participant's female partner who becomes pregnant while the male participant is in this study. This applies only to male participants who receive study intervention.
- After obtaining the necessary signed informed consent from the pregnant female partner directly, the investigator will record pregnancy information on the appropriate form and submit it to the Sponsor within 24 hours of learning of the partner's pregnancy. The female partner will also be followed to determine the outcome of the pregnancy. Information on the status of the mother and child will be forwarded to the Sponsor. Generally, the follow-up will be no longer than 6 months following the estimated delivery date. Any termination of the pregnancy will be reported regardless of fetal status (presence or absence of anomalies) or indication for the procedure.

Female Participants Who Become Pregnant

- The investigator will collect pregnancy information on any female participant who becomes pregnant while participating in this study. The initial information will be recorded on the appropriate form and submitted to the Sponsor within 24 hours of learning of a participant's pregnancy.
- The participant will be followed to determine the outcome of the pregnancy. The investigator will collect follow-up information on the participant and the neonate and the information will be forwarded to the Sponsor. Generally, follow-up will not be required for longer than 6 months beyond the estimated delivery date. Any termination of pregnancy will be reported, regardless of fetal status (presence or absence of anomalies) or indication for the procedure.
- While pregnancy itself is not considered to be an AE or SAE, any pregnancy complication or elective termination of a pregnancy for medical reasons will be reported as an AE or SAE.
- A spontaneous abortion (occurring at <22 weeks gestational age) or still birth (occurring at >22 weeks gestational age) is always considered to be an SAE and will be reported as such.
- Any post-study pregnancy-related SAE considered reasonably related to the study intervention by the investigator will be reported to the Sponsor as described in

Section 8.6.3. While the investigator is not obligated to actively seek this information in former study participants, he or she may learn of an SAE through spontaneous reporting.

- Any female participant who becomes pregnant while participating in the study will discontinue study intervention.

9.5. Appendix 5. Genetics

Use/Analysis of DNA

- Genetic variation may impact a participant's response to study intervention, susceptibility to, and severity and progression of disease. Variable response to study intervention may be due to genetic determinants that impact drug absorption, distribution, metabolism, and excretion.
- *Mechanism of action (*MOA*)* of the drug
- Disease etiology
- And/or molecular subtype of the disease being treated. Therefore, where local regulations and *Investigational Review Board/Independent Ethics Committees (*IRB/IEC*)* allow, a blood sample will be collected for DNA analysis from consenting participants.
- DNA samples will be used for research related to NeoTCR-P1 or solid tumors and related diseases. They may also be used to develop tests/assays including diagnostic tests related to study intervention and/or interventions of this drug class and solid tumors. Genetic research may consist of the analysis of 1 or more candidate genes or the analysis of genetic markers throughout the genome or analysis of the entire genome (as appropriate).
- DNA samples will be analyzed for the presence of neoantigens as well as potential markers of resistance. Additional analyses may be conducted if it is hypothesized that this may help further understand the clinical data.
- The samples may be analyzed as part of a multi-study assessment of genetic factors involved in the response to NeoTCR-P1 or study interventions of this class to understand study disease or related conditions.
- The results of genetic analyses may be reported in the clinical study report (CSR) or in a separate study summary.
- The Sponsor will store the DNA samples in a secure storage space with adequate measures to protect confidentiality.
- The samples will be retained while research on NeoTCR-P1 and the immune response to cancer continues but no longer than 15 years or other period as per local requirement.

9.6. Appendix 6. Response Evaluation Criteria in Solid Tumors. Modified Excerpt from Original Publication

Selected sections from the *Response Evaluation Criteria in Solid Tumors version 1.1 (*RECIST v1.1*)* are presented below, with slight modifications and the addition of explanatory text for clarity.

MEASURABILITY OF TUMORS AT BASELINE

DEFINITIONS

At baseline, tumor lesions/lymph nodes will be categorized as either measurable or non-measurable as follows:

Measurable Tumor Lesions

Tumor Lesions. Tumor lesions must be accurately measured in at least 1 dimension (longest diameter in the plan of measurements is to be recorded) with a minimum size of

- 10 mm by *computed tomography* (CT) or magnetic resonance imaging (MRI) scan (CT/MRI scan slice thickness/interval no greater than 5 mm)
- 10 mm caliper measurements by clinical examination (lesions that cannot be accurately measured by calipers should be recorded as non-measurable)
- 20 mm by chest X-ray

Malignant Lymph Nodes. To be considered pathologically enlarged and measurable, a lymph node must be ≥15 mm in the short axis when assessed by CT scan. At baseline and in follow-up, only the short axis will be measured and followed. See also notes below on "Baseline Documentation of Target and Non-Target Lesions" for information on lymph node measurement.

Non-Measurable Tumor Lesions

Non-measurable tumor lesions encompass small lesions (longest diameter <10 mm or pathological lymph nodes ≥10 to <15 mm short axis as well as truly non-measurable lesions. Lesions considered truly non-measurable include lymphangitic involvement of skin or lung, peritoneal spread, inflammatory breast disease, and abdominal masses/abdominal organomegaly identified by physical examination that is *not* measurable by reproducible imaging techniques.

Special Consideration Regarding Lesions Measurability

Bone lesions, cystic lesions, and lesions previously treated with local therapy require special considerations as outlined below:

Bone lesions:

- Bones scan, *positron emission tomography* (PET) scan, or plain films are not considered adequate imaging techniques to measure bone lesions. However, these techniques can be used to confirm the presence or disappearance of bone lesions.
- Lytic bone lesions lesion or mixed lytic-blastic lesions, with identifiable soft tissue components, that can be evaluated by cross-sectional imaging techniques such as CT or MRI can be considered measurable lesions if the soft tissue component meets the definition of measurability described above.
- Blastic bone lesions are non-measurable.

Cystic lesions:

- Lesions that meet the criteria for radiographically defined simple cysts should not be considered malignant lesions (neither measurable nor no-measurable since they are, by definition, simple cysts.
- Cystic lesions thought to represent cystic metastases can be considered measurable lesions if they meet the definition of measurability described above. However, if non-cystic lesions are present in the same *participant*, these are preferred for selections as target lesions.

Lesions with prior local treatment:

- Tumor lesion situated in a previously irradiated area or in an area subjected to other local-regional therapy are usually not considered measurable unless there has been demonstrated progression in the lesion. Study protocols should detail the conditions under which such lesions would be considered measurable.

TARGET LESIONS: SPECIFICATIONS BY METHODS OF MEASUREMENT

Measurement of Lesions

All measurements should be recorded in metric notation, using calipers if clinically assessed. All baseline evaluations should be performed as close as possible to the treatment start and never more than 4 weeks before the beginning of the treatment.

Method of Assessment

The same method of assessment and the same technique should be used to characterize each identified and reported lesion at baseline and during the study. Imaging-based evaluation should always be the preferred option.

Clinical Lesions. Clinical lesions will be considered measurable only when they are superficial and ≥10 mm in diameter as assessed using calipers (e.g., skin nodules). For the case of skin lesions, documentation by color photography, including a ruler to estimate the size of the lesion is required.

Chest X Ray. Chest CT scan is preferred over chest X-ray, particularly when progression is an important endpoint, since CT is more sensitive than X-ray, particularly in identifying new lesions. However, lesions on chest X-ray may be considered measurable if they are clearly defined and surrounded by aerated lung.

CT or MRI. CT is the best currently available and most reproducible method to measure lesions selected for response assessment. This guideline has defined measurability of lesions on CT scan on the basis of the assumption that CT slice thickness is 5 mm or less. When CT scans have slice thickness greater than 5 mm, the minimum size for a measurable lesion should be twice the slice thickness. MRI is also acceptable.

If prior to enrollment it is *known* that a *participant* is unable to undergo CT scans with intravenous (IV) contrast because of allergy or renal insufficiency, then the decision as to whether a non-contrast CT scan or MRI scan (without IV contrast) will be used to evaluate the

participant at baseline and during the study should be guided by the tumor type under investigation and the *anatomic* location of the disease. For *participants* who develop contraindications to contrast after a baseline contrast CT scan is *done*, the decision as to whether non-contrast CT or MRI (enhanced or non-enhanced) scan will be performed should also be based on the tumor type and the anatomic location of the disease and should be optimized to allow for comparison with the prior studies if possible. Each case should be discussed with the radiologist to determine if substitution of these other approaches is possible and, if not, the *participant* should be considered not evaluable from that point forward. Care must be taken in measurement of target lesion on a different modality and interpretation of non-target disease or new lesions since the same lesion may appear to have a different size using a new modality.

Ultrasound. Ultrasound is not useful in the assessment of lesion size and should not be used as a method of measurement.

Endoscopy, Laparoscopy, Tumor Markers, Cytology, Histology. The utilization of these techniques for objective tumor evaluation cannot generally be advised.

TUMOR RESPONSE EVALUATION

ASSESSMENT OF OVERALL TUMOR BURDEN AND MEASURABLE DISEASE

To assess objective response or future progression, it is necessary to estimate the overall tumor burden at baseline and to use this as a comparator for subsequent measurements. Measurable disease is defined by the presence of at least 1 measurable lesion, as defined above.

BASELINE DOCUMENTATION OF TARGET AND NON-TARGET LESIONS

When more than 1 measurable lesion is present at baseline, all lesions up to a maximum of 5 lesions total (and a maximum of 2 lesions per organ) representative of all involved organs should be identified as target lesions and will be recorded and measured at baseline. This means in instances where *participants* have only 1 or 2 organ sites involved, a maximum of 2 lesions (one site) and 4 lesions (2 sites), respectively will be recorded. Other lesions (albeit measurable) in those organs will be recorded as non-target lesions (even if the size if >10 mm by CT scan).

Target lesions should be selected on the basis of their size (lesions with the longest diameter) and be representative of all involved organs, but additionally, should lend themselves to reproducible repeated measurements. It may be the case that on occasion, the largest lesion does not lend itself to reproducible measurements, in which circumstance the next large lesion that can be measured reproducibly should be selected.

Lymph nodes merit special mention since they are normal anatomical structures that may be visible by imaging eve in tot involved by tumor. As noted above, pathological nodes that are defined as measurable and may be identified as target lesions must meet the criterion of a short axis of ≥15 mm by CT scan. Only the short axis of these nodes will contribute to the baseline sum. The shore axis of these node is the diameter normally used by radiologists to judge if a node is involved by solid tumor. Nodal size is normally reported as 2 dimensions in the plane in which the image is obtained (for CT scan, this is almost always the axial plan

for MRI scan the plane of acquisition may be axial, sagittal, or corona,). The smaller of these measures is the short axis. For example, an abdominal node that is reported as being

20 mm × 30 mm has a short axis or 20 mm and qualifies as a malignant measurable lymph node. All other pathological nodes (those with short axis ≥10 mm but <15 mm) should be considered non-target lesions. Nodes that have a short axis <10 mm are considered non-pathological and should not be recorded or followed.

Lesions irradiated within 3 weeks prior to Day 0 may not be counted as target lesions.

A sum of the diameters (longest of non-nodal lesions, short axis for nodal lesions) for all target lesions will be calculated and reported as the baseline sum of diameters. If lymph nodes are to be included in the sum, then, as noted above, only the short axis is added into the sum. The baseline sum of diameters will be used as a reference to further characterize any objective tumor regression in the measurable dimension of the disease.

All other lesions (or sites of disease), including pathological lymph nodes, should be identified as non-target lesions and should also be recorded at baseline. Measurements are not required, and these lesions should be followed as “present,” “absent,” or in rare cases “unequivocal progression.”

In addition, it is possible to record multiple non-target lesions involving the same organ as a single item on the *e*CRF (e.g., “multiple enlarged pelvic lymph nodes” or “multiple liver metastasis”).

RESPONSE CRITERIA

Evaluation of Target Lesions

The section provides the definition of the criteria used to determine objective tumor response for target lesions

- **Complete response (CR):** Disappearance of all target lesions

  Any pathological lymph nodes (whether target or non-target) must have reduction in short axis to <10 mm.
- **Partial response (PR):** At least a 30% decrease in the sum of diameters of target lesions, taking as reference the baseline sum of diameters
- **Progressive disease (PD):** At least a 20% increase in the sum of diameters of target lesions, taking as reference the smallest sum on study (nadir), including baseline

  In addition to the relative increase of 20%, the sum must also demonstrate an absolute increase of at least 5 mm.
  The appearance of 1 or more new lesions is also considered progression.
- **Stable disease (SD):** Neither sufficient shrinkage to quality for PR no sufficient increase to qualify for PD, taking as reference the smallest sum on study.

Special Notes on the Assessment of Target Lesions

Lymph Nodes. Lymph nodes identified as target lesions should always have the actual short axis measurement recorded (measured in the same anatomical plane as the baseline examination), even if the nodes regress to <10 mm on study. This means that when lymph nodes are included

as target lesions, the sum of lesions may not be zero even if CR criteria are met since a normal lymph node is defined as having a short axis <10 mm.

Target Lesions that Become Too Small to Measure. While on study, all lesions (nodal and non-nodal) recorded at baseline should have their actual measurements recorded at each subsequent evaluation, even when very small (e.g., 2 mm). However sometimes lesions or lymph nodes that are recorded as target lesions at baseline become so faint on CT scan that the radiologist may not feel comfortable assigning an exact measure and may report them as being too small to measure. When this occurs, it is important that a value be recorded on the *e*CRF as follows:

- If it is the opinion of the radiologist that the lesion has likely disappeared, the measurement should be recorded as 0 mm.
- If the lesion is believed to be present and is faintly seen but too small to measure, a default value of 5 mm should be assigned and BML (below measurable limits) should be ticked. (Note. It is less likely that this rule will be used for lymph nodes since they usually have a definable size when normal and are frequently surrounded by fat such as in the retroperitoneum; however, if a lymphoma node is believed to be present and is faintly see but too small to measure, a default value of 5 mm should be assigned in this circumstance as well and BML should also be ticked.)

Lesions that Split or Coalesce on Treatment. When non-nodal lesions fragment, the longest diameters of the fragmented portions should be added together to calculate the target lesions sum. Similarly, as lesions coalesce, a plane between them may be maintained that would aid in obtaining maximal diameter measurements of each individual lesion. If the lesions have truly coalesced such that the are no longer separable, the vector of the longest diameter in this instance should be the maximal longest diameter for the coalesced lesion.

Evaluation of Non-Target Lesions

This section provides the definition of the criteria used to determine the tumor response for the group of non-target lesions. Although some non-target lesions may actually be measurable, they need not be measured and, instead, should be assessed only qualitatively at the timepoints specified in the protocol.

- CR: Disappearance of all non-target lesions

  All lymph nodes must be non-pathological in size (<10 mm in short axis).
- Non-CR/Non-PD: Persistence of 1 or more non-target lesion(s)
- PD: Unequivocal progression of existing non-target lesions

Special Notes on Assessment of Progression of Non-Target Disease

When the *Participant*

Also Has Measurable Disease. In this setting, to achieve unequivocal progression on the basis of the non-target disease, there must be an overall level of substantial worsening in non-target

disease in a magnitude that, even in the presence of SD or PR in target disease, the overall tumor burden has increased sufficiently to merit discontinuation of therapy. A modest increase in the size of 1 or more non-target lesions is usually not sufficient to qualify for unequivocal progression status. The designation of overall progression solely on the basis of change in non-target disease in the face of SD or PR of target disease will therefore be extremely rare.

When the *Participant* Only Has Non-Measurable Disease. This circumstance arises in some studies when it is not a criterion of study entry to have measurable disease. The same general concepts apply here as noted above; however, in this instance, there is no measurable disease assessment to factor into the interpretation of an increase in non-measurable disease burden. Because worsening in no-target disease cannot be easily quantified, a useful test that can be applied when assessing *participants* for unequivocal progression is to consider if the increase in overall disease burden based on the change in non-measurable disease is comparable in magnitude to the increase that would be required to declared PD for measurable disease, that is, an increase in tumor burden representing an additional 73% increase in volume (which is equivalent to a 20% increase in diameter in a measurable lesion). Examples include an increase from a pleural effusion from "trace" to "large" or an increase in lymphangitic disease from localized to widespread or may be described in protocols as "sufficient to require a change in therapy." If unequivocal progression is observed, the *participant* should be considered to have had overall PD at that point. Although it would be ideal to have objective criteria to apply to non-measurable disease, the very nature of that disease makes it impossible to do so, therefore, the increase must be substantial.

New Lesions

The appearance of new malignant lesions denotes disease progression; therefore, some comments on detection of new lesions are important. There are no specific criteria for the identification of new radiographic lesions; however, the finding of a new lesion should be unequivocal, that is, not attributable to differences in scanning technique, change in imaging modality, or findings *thought* to represent something other than tumor (for example, some "new" bone lesions may be simply healing or flare of preexisting lesions). This is particularly important when the *participant's* baseline lesions show PR or CR. For example, necrosis of a liver lesion may be reported on a CT scan reports as a "new" cystic lesion, *but should not be considered to be a new lesion for tumor assessment purposes.*

A lesion identified during the study in an anatomical location that was not scanned at baseline is considered a new lesion and will indicated disease progression.

If a new lesion is equivocal, for example because of its small size, continued therapy and follow-up evaluation will clarify if it represents truly new disease. If repeat scans confirm there is definitely a new lesion, then progression should be declared using the date of the initial scan.

EVALUATION OF RESPONSE

Timepoint Response (Overall Response)

It is assumed that at each protocol-specified timepoint, a response assessment occurs. Table 1 provides a summary of the overall response status calculation at each timepoint for *participants* who have measurable disease at baseline.

When *participants* have non-measurable (therefore, non-target) disease only, Table 2 is to be used.

Table 1 Timepoint Response: *Participants* with Target Lesions (with or without Non-Target Lesions)

Target Lesions	Non-Target Lesions	New Lesions	Overall Response
CR	CR	No	CR
CR	Non-CR/Non-PD	No	PR
CR	Not evaluated	No	PR
PR	Non-PD or not all evaluated	No	PR
SD	Non-PD or not all evaluated	No	SD
Not all evaluated	Non-PD	No	NE
PD	Any	Yes or no	PD
Any	PD	Yes or no	PD
Any	Any	Yes	PD

CR = complete response; NE = Not evaluable; PR = partial response; PD = progressive disease.

Table 2 Timepoint Response: *Participants* with Non-Target Lesions Only

Non-Target Lesions	New Lesions	Overall Response
CR	No	CR
Non-CR/Non-PD	No	Non-CR/Non-PD [a]
Not all evaluated	No	NE
Unequivocal PD	Yes or no	PD
Any	Yes	PD

CR = complete response; *NE = Not evaluable*; PD = progressive disease.

[a] "Non-CR/Non-PD" is preferred over "stable disease" for non-target disease since stable disease is increasingly used as an endpoint for assessment of efficacy in some studies, thus, assigning "stable disease" when no lesions can be measured is not advised.

Missing Assessments and Not-Evaluable Designation

When no imaging/measurement is done at all at a particular timepoint, the *participants* is not evaluable at that timepoint. If only a subset of lesion measurements ate made at an assessment, usually the case is also considered not evaluable at that timepoint, unless a convincing argument can be made that the contributing of the individual missing lesions (s) would not change the assigned timepoint response. This would be most likely to happen in the case of PD. For example, if a *participant* had a baseline sum of 50 mm with 3 measured lesions and, during the

study, only 2 lesions were assessed, but these gave a sum of 80 mm; the *participants* will have achieved PD status, regardless of the contribution of the missing lesion.

If 1 or more target lesions were not assessed, either because the scan was not done or the scan could not be assessed because of poor image quality or obstructed view, the response for the target lesion should be "unable to assess" since the *participant* is not evaluable. Similarly, if 1 or more non-target lesions are not assessed, the response for non-target lesions should be "unable to assess" except where there is clear progression. Overall response would be "unable to assess" if either the target response or the non-target response is "unable to assess," except when there is clear of progression as this equates with the case being not evaluable at that timepoint.

Table 3 *Best* Overall Response When Confirmation is Required

Overall Response at First Timepoint	Overall Response at Subsequent Timepoint	Best Overall Response
CR	CR	CR
CR	PR	SD, PD, or PR [a]
CR	SD	SD, provided minimum duration for SD was met; otherwise, PD
CR	PD	SD, provided minimum duration for SD was met; otherwise, PD
CR	NE	SD, provided minimum duration for SD was met; otherwise, NE
PR	CR	PR
PR	PR	PR
PR	SD	SD
PR	PD	SD, provided minimum duration for SD was met; otherwise, PD
PR	NE	SD, provided minimum duration for SD was met; otherwise, NE
NE	NE	NE

CR = complete response; NE = Not evaluable; PD = progressive disease; PR = partial response; SD = stable disease.

[a] If a CR is truly met at the first timepoint, any disease seen at a subsequent timepoint, even disease meeting PR criteria relative to baseline, qualifies as PD at that point (since disease meeting PR criteria relative to baseline, qualifies as PD at that point (since disease must have reappeared after CR). Best response would depend on whether the minimum duration for SD was met. However, sometimes CR may be claimed when subsequent scans suggest small lesions were likely still present and in fact the *participant* had PR, not CR, at the first timepoint. Under these circumstances, the original CR should be changed to the PR and the best response is PR.

Special Notes on Response Assessment

When nodal disease is included in the sum of target lesions and the nodes decrease to "normal" size (<10 mm), they may still have a measurement reported on scans. This measurement should be recorded even though the nodes are normal in order not to overstate progression should it be based on *an* increase in *the* size of the nodes. As noted earlier, this means that *in participants* with CR*, the sum of diameters* may not *equal* "zero."

Participants with a global deterioration of health status requiring discontinuation of treatment without objective evidence of disease progression at that time should be reported as "symptomatic deterioration." Every effort should be made to document objective progression

even after discontinuation of study treatment. Symptomatic deterioration is not a descriptor of an objective response; it is a reason for stopping study therapy. The objective response status of such *participants* is to be determined by evaluation of target and non-target disease as shown in Table 1 and Table 2.

For equivocal finding of progression (e.g., very small and uncertain new lesions, cystic changes or necrosis in existing lesions), treatment may continue until the next scheduled assessment. If at the next schedule assessment progression is confirmed, the date of progression should be the earlier date when the progression was suspected.

If a *participant* undergoes an excisional biopsy or other appropriate approach (e.g., multiple passes with large core needle) of a new lesion or an existing solitary progressive lesion that following serial sectioning and pathological examination reveals no evidence of malignancy (e.g., inflammatory cells, fibrosis) then the new lesions or solitary progressive lesion will not constitute disease progression.

In studies for which patients with advance disease are eligible (i.e., Primary disease still or partially present), the primary tumor should also be captured as a target or non-target lesions, as appropriate. This is to avoid an incorrect assessment of CR if the primary tumor is still present but not evaluated as a target or non-target lesion.

9.7. Appendix 7. ECOG Performance Status

Grade	Criteria
0	Fully active, able to carry on all pre-disease performance without restriction
1	Restricted in physically strenuous activity, but ambulatory and able to carry out work in a light or sedentary nature, e.g., light housework, office work
2	Ambulatory and capable of all selfcare, but unable to carry out any work activities up and about more than 50% of waking hours
3	Capable of only limited selfcare<br>Confined to bed or chair more than 50% of waking hours
4	Completely disabled<br>Cannot carry on any selfcare<br>Totally confined to bed or chair
5	Dead

9.8. Appendix 8. Abbreviations

ACT	adoptive T-cell therapy
AI	aromatase inhibitor
ADT	androgen-deprivation therapy
AE	adverse event
AESI	adverse event of special interest
ALC	absolute lymphocyte count
ALL	*acute lymphocytic leukemia*
ANC	absolute neutrophil count
ASTCT	Cytokine Release Syndrome Consensus Grading Scale
B-ALL	*B-cell acute lymphoblastic leukemia*
BC	*brain cancer*
BID	twice daily
BiPAP	*bilevel positive airway pressure*
BML	*below measurable limits*
bpm	*beats per minute*
BUN	*blood urea nitrogen*
CAR	chimeric antigen receptor
CBC	complete blood count
CEA	*carcinoembryonic antigen*
cGCPs	current good clinical practices
CI	Confidence interval
CIOMS	Council for International Organizations of Medical Sciences
CLS	Capillary leak syndrome
CMV	cytomegolovirus
CNS	central nervous system
CONSORT	Consolidated Standards of Reporting Trials
CPAP	*continuous positive airway pressure*
CPF	Cell Processing Facility
CPS	combined positive score
CR	complete response
CRC	colorectal cancer
CRP	C-reactive protein
CRPC	castration-resistant prostate cancer
CRS	cytokine release syndrome
CSF	cerebrospinal fluid
CSR	clinical study report
CT	computed tomography
CTLA	cytotoxic T-lymphocyte-associated
DLBCL	diffuse large B-cell lymphoma

DLT	dose-limiting toxicity
dMMR	mismatch repair deficiency
DMSO	dimethyl sulfoxide
DOR	*duration of response*
EBV	*Epstein Barr virus*
ECG	electrocardiogram
ECOG	Eastern Cooperative Oncology Group
ECHO	*echocardiogram*
eCRF	electronic Case Report Form
EDC	electronic data capture
EEG	electroencephalogram
ELISPOT	*enzyme-linked immunosorbent spot*
EMA	European Medicines Agency
ER	estrogen receptor
ER+	estrogen receptor positive
FEV1	forced expiratory volume in one second
FDA	Food and Drug Administration
FNA	fine needle aspiration
FSH	follicle stimulating hormone
FVC	forced vital capacity
GC	gemcitabine and cisplatin
GCP	Good Clinical Practice
G-CSF	granulocyte colony-stimulating factor
GM-CSF	granulocyte-macrophage colony-stimulating factor
GnRH	gonadotropin release hormone
GMP	Good Manufacturing Practice
GVHD	graft-versus-host disease
HCC	hepatocellular carcinoma
HCV	*hepatitis C virus*
HER2	human epidermal growth factor receptor 2
HIPAA	Health Insurance Portability and Accountability Act
HLA	human leukocyte antigen
HLH	hemophagocytic lymphohistiocytosis
HNSCC	head and neck squamous cell cancer
HPV	human papillomavirus
HR+	hormone receptor positive
HR-	hormone receptor negative
HRT	hormonal replacement therapy
ICANS	immune effector cell-associated neurotoxicity
ICC	investigator’s choice of chemotherapy

ICE	Immune Effector Cell-Associated Encephalopathy
ICF	informed consent form
IEC	Independent Ethics Committees
IFN	interferon
IHC	immunohistochemistry
IL	interleukin
IMP	Investigational Medicinal Product
IND	Investigational New Drug (Application)
IP	Investigational Product
IRB	Investigational Review Board
IV	intravenous
LAK	lymphokine-activated killer
LDH	lactate dehydrogenase
LTFU	long-term follow up
MAD	maximum administered dose
MBC	metastatic breast cancer
MHC	major histocompatibility complex
MOA	mechanism of action
MRI	magnetic resonance imaging
MSI-H	high microsatellite instability
MSS	microsatellite-stable
MTD	maximum tolerated dose
MVAC	methotrexate, vinblastine, doxorubicin, and cisplatin
NCCN	National Comprehensive Cancer Network
NCI	National Cancer Institute
NCI CTCAE	National Cancer Institute Common Terminology Criteria for Adverse Events
NGS	next generation sequencing
NeoTCR	personalized adoptive T-cell receptor
NHL	non-Hodgkin's lymphoma
nivo	*nivolumab*
NSCLC	non-small cell lung cancer
ORR	objective response rate
OS	overall survival
P1	Product 1
PARP	poly ADP ribose polymerase
PBMC	peripheral blood mononuclear cell
PCR	polymerase chain reaction
PCWG3	Prostate Cancer Working Group 3
PD	pharmacodynamic
PD-1	programmed death 1

PD-L1	programmed death ligand 1
PET	positron emission tomography
PFS	progression-free survival
PFT	*pulmonary function test*
PICC	*peripherally inserted central catheter*
PK	pharmacokinetic(s)
PR	partial response
PR+	progesterone receptor-positive
PSA	prostate-specific antigen
Q4W	*Every 4 weeks*
RCC	renal cell cancer
RECIST	Response Evaluation Criteria in Solid Tumors
SAE	serious adverse event
sCD25	*soluble interleuken-2 receptor*
SCLC	small cell lung cancer
SD	*stable disease*
SEER	Surveillance, Epidemiology, and End Results
SERM	selective estrogen receptor modulators
SERD	selective estrogen receptor degraders
SD	stable disease
SmPC	Summary of Product Characteristics
SOA	Schedule of Activities
SUSAR	suspected unexpected serious adverse reactions
SRT	safety review team
T_{cm}	T central memory
TCR	T-cell receptor
TIL	tumor-infiltrating T cells
TLS	tumor lysis syndrome
T_{msc}	T memory stem cell
TNBC	triple-negative breast cancer
TNF	tumor necrosis factor
u3TCR	up to 3 TCR clonal populations per product
ULN	upper limit of normal
USPI	United States product insert
WBC	*white blood cell*
WES	whole exome sequencing
WHO	World Health Organization
WOCBP	women of childbearing potential

10. References

Abe Y, Choi I, Hara K, Matsushima T, Nishimura J, Inaba S, et al. Hemophagocytic syndrome: a rare complication of allogeneic nonmyeloablative hematopoietic stem cell transplantation. Bone Marrow Transpl. 2002 May;29(9):799.

Alyea EP, Kim HT, Ho V, Cutler C, Gribben J, DeAngelo DJ, et al. Comparative outcome of nonmyeloablative and myeloablative allogeneic hematopoietic cell transplantation for patients older than 50 years of age. Blood. 2005;105(4):1810-4.

American Cancer Society. Key statistics for ovarian cancer [internet]. 2019. Available from: https://www.cancer.org/cancer/ovarian-cancer/about/key-statistics.html.

Angelova M, Mlecnik B, Vasaturo A, Bindea G, Fredriksen T, Lafontaine L, et al. Evolution of metastases in space and time under immune selection. Cell. 2018;175(3):751-65.

Arber C, Abhyankar H, Heslop HE, Brenner MK, Liu H, Dotti G, et al. The immunogenicity of virus-derived 2A sequences in immunocompetent individuals. Gene Ther. 2013 Sep;20(9):958.

Atkins MB, Lotze MT, Dutcher JP, Fisher RI, Weiss G, Margolin K, et al. High-dose recombinant interleukin 2 therapy for patients with metastatic melanoma: analysis of 270 patients treated between 1985 and 1993. J Clin Oncol. 1999;17(7):2105-.

Aupérin A, Le Péchoux C, Rolland E, Curran WJ, Furuse K, Fournel P et al. Meta-analysis of concomitant versus sequential radiochemotherapy in locally advanced non-small-cell lung cancer. J Clin Oncol. 2010 May 1;28(13):2181-90.

Bai S, Jorga K, Xin Y, Jin D, Zheng Y, Damico-Beyer LA, et al. A guide to rational dosing of monoclonal antibodies. Clin Pharmacokinet. 2012;51(2):119-35.

Barrett DM, Teachey DT, Grupp SA. Toxicity management for patients receiving novel T-cell engaging therapies. Curr Opin Pediatr. 2014;26(1):43.

Bennouna J, Sastre J, Arnold D, Österlund P, Greil R, Van Cutsem E, et al. Continuation of bevacizumab after first progression in metastatic colorectal cancer (ML18147): a randomised phase 3 trial. Lancet Oncol. 2013;14(1):29-37.

Bianchini D, Lorente D, Rodriguez-Vida A, Omlin A, Pezaro C, Ferraldeschi R, et al. Antitumour activity of enzalutamide (MDV3100) in patients with metastatic castration-resistant prostate cancer (CRPC) pre-treated with docetaxel and abiraterone. Eur J Cancer. 2014;50(1):78-84.

Blair DA, Turner DL, Bose TO, Pham QM, Bouchard KR, Williams KJ, et al. Duration of antigen availability influences the expansion and memory differentiation of T cells. J Immunol. 2011;187(5):2310-21.

Blum JL, Dees EC, Vukelja SJ, Amare M, Gill DP, McMahon RT, et al. Phase II trial of capecitabine and weekly paclitaxel in patients with metastatic breast cancer previously treated with every-3-week taxane therapy. Clin Breast Cancer. 2007;7(6):465-70.

Bollard CM, Tripic T, Cruz CR, Dotti G, Gottschalk S, Torrano V, et al. Tumor-specific T-cells engineered to overcome tumor immune evasion induce clinical responses in patients with relapsed Hodgkin lymphoma. J Clin Oncol. 2018;36(11):1128-39.

Brahmer JR, Lacchetti C, Schneider BJ, Atkins MB, Brassil KJ, Caterino JM, et al. Management of immune-related adverse events in patients treated with immune checkpoint inhibitor therapy: American Society of Clinical Oncology Clinical Practice Guideline. Journal of Clinical Oncology. 2018;36(17):1714-68.

Brahmer JR, Tykodi SS, Chow LQ, Hwu WJ, Topalian SL, Hwu P, et al. Safety and activity of anti–PD-L1 antibody in patients with advanced cancer. New Engl J Med. 2012;366(26):2455-65.

Bray F, Ferlay J, Soerjomataram I, Siegel RL, Torre LA, Jemal A. Global cancer statistics 2018: GLOBOCAN estimates of incidence and mortality worldwide for 36 cancers in 185 countries. CA Cancer J Clin 2018;68:394-424.

Brudno JN, Maric I, Hartman SD, Rose JJ, Wang M, Lam N, et al. T cells genetically modified to express an anti–B-cell maturation antigen chimeric antigen receptor cause remissions of poor-prognosis relapsed multiple myeloma. J Clin Oncol. 2018;36(22):2267-80.

Burt RK, Craig RM, Milanetti F, Quigley K, Gozdziak P, Bucha J, et al. Autologous nonmyeloablative hematopoietic stem cell transplantation in patients with severe anti-TNF refractory Crohn disease: long-term follow-up. Blood. 2010;116(26):6123-32.

Busch DH, Fräßle SP, Sommermeyer D, Buchholz VR, Riddell SR. Role of memory T cell subsets for adoptive immunotherapy. Seminars in Immunology. 2018;28(1):28-34.

Burtness B, Harrington KJ, Greil R, et al. Pembrolizumab alone or with chemotherapy versus cetuximab with chemotherapy for recurrent or metastatic squamous cell carcinoma of the head and neck (KEYNOTE-048): a randomised, open-label, phase 3 study Lancet 2019;394:1915-1928.

Cairo MS, Bishop M. Tumour lysis syndrome: new therapeutic strategies and classification. Brit J Haematol. 2004;127(1):3-11.

Cairo MS, Coiffier B, Reiter A, Younes A, TLS Expert Panel. Recommendations for the evaluation of risk and prophylaxis of tumour lysis syndrome (TLS) in adults and children with malignant diseases: an expert TLS panel consensus. Br J Hematol. 2010;149(4):578-86.

Cameron BJ, Gerry AB, Dukes J, Harper JV, Kannan V, Bianchi FC, et al. Identification of a Titin-derived HLA-A1–presented peptide as a cross-reactive target for engineered MAGE A3–directed T cells. Sci Transl Med. 2013;5(197):197ra103.

Cancer.net. Lung Cancer – Non-Small Cell: Statistics [internet]. 2020. Available from: https://www.cancer.net/cancer-types/lung-cancer-non-small-cell/statistics

Chapman PB, Hauschild A, Robert C, Larkin JM, Haanen JB, Ribas A, et al. Updated overall survival (OS) results for BRIM-3, a phase III randomized, open-label, multicenter trial comparing BRAF inhibitor vemurafenib (vem) with dacarbazine (DTIC) in previously untreated patients with BRAFV600E-mutated melanoma. J Clin Oncol. 2012;30(15):8502.

Chatzkel JA, Swank J, Ludlow S, Lombardi K, Croft C, et al. Overall responses with coordinated pembrolizumab and high dose IL-2 (5-in-a-row schedule) for therapy of metastatic clear cell renal cancer: A single center, single arm trial. J Clin Oncol. 2019; 37(7)_suppl

Chen B, Feng B, Tang Y, You Y, Wang Y, Hou W, et al. Blocking GluN2B subunits reverses the enhanced seizure susceptibility after prolonged febrile seizures with a wide therapeutic time-window. Exp Neurol. 2016;283:29-38.

Chen DS, Irving BA, Hodi FS. Molecular pathways: next-generation immunotherapy—inhibiting programmed death-ligand 1 and programmed death-1. Clin Cancer Res. 2012;18(24):6580-7.

Cherkassky L, Morello A, Villena-Vargas J, Feng Y, Dimitrov DS, Jones DR, et al. Human CAR-T cells with cell-intrinsic PD-1 checkpoint blockade resist tumor-mediated inhibition. J Clin Invest. 2016;126(8):3130-44.

Chodon T, Comin-Anduix B, Chmielowski B, Koya RC, Wu Z, Auerbach M, et al. Adoptive transfer of MART-1 T-cell receptor transgenic lymphocytes and dendritic cell vaccination in patients with metastatic melanoma. Clin Cancer Res. 2014;20(9):2457-65.

Chong EA, Levine BL, Grupp SA, Davis M, Siegel DL, Maude SL, et al. CD19-Directed CAR-T-Cell (CTL019) product viability and clinical outcomes in non-Hodgkin lymphomas and B-cell acute lymphoblastic leukemia. Blood. 2018;132(Suppl 1):197.

Chow LQM. Head and Neck Cancer. N Engl J Med. 2020 Jan 2;382(1):60-72.

D'Angelo SP, Melchiori L, Merchant MS, Bernstein D, Glod J, Kaplan R, Grupp S, Tap WD, Chagin K, Binder GK, Basu S. Antitumor activity associated with prolonged persistence of adoptively transferred NY-ESO-1 c259T cells in synovial sarcoma. Cancer Discover. 2018;8(8):944-57.

Das RK, Vernau L, Grupp SA, Barrett DM. Naive T cell deficits at diagnosis and after chemotherapy impair cell therapy potential in pediatric cancers. Cancer Discover. 2019:CD-18.

de Bono JS, Logothetis CJ, Molina A, Fizazi K, North S, Chu L, et al. Abiraterone and increased survival in metastatic prostate cancer. New Engl J Med. 2011;364(21):1995-2005.

de Bono JS, Oudard S, Ozguroglu M, Hansen S, Machiels JP, Kocak I, et al. Prednisone plus cabazitaxel or mitoxantrone for metastatic castration-resistant prostate cancer progressing after docetaxel treatment: a randomised open-label trial. Lancet. 2010;376(9747):1147-54.

Doran SL, Stevanović S, Adhikary S, et al. T-Cell Receptor Gene Therapy for Human Papillomavirus–Associated Epithelial Cancers: A First-in-Human, Phase I/II Study. J Clin Oncol. 2019:37(30):2759.

Dudley ME, Wunderlich JR, Yang JC, Sherry RM, Topalian SL, Restifo NP, et al. Adoptive cell transfer therapy following non-myeloablative but lymphodepleting chemotherapy for the treatment of patients with refractory metastatic melanoma. J Clin Oncol. 2005;23(10):2346.

Dudley ME, Yang JC, Sherry R, Hughes MS, Royal R, Kammula U, Robbins PF, Huang J, Citrin DE, Leitman SF, Wunderlich J. Adoptive cell therapy for patients with metastatic melanoma: evaluation of intensive myeloablative chemoradiation preparative regimens. J Clin Oncol. 2008;26(32):5233.

Dummer W, Niethammer AG, Baccala R, Lawson BR, Wagner N, Reisfeld RA, Theofilopoulos AN. T cell homeostatic proliferation elicits effective antitumor autoimmunity. J Clin Invest. 2002;110(2):185-92.

Dutcher JP, Schwartzentruber DJ, Kaufman HL, Agarwala SS, Tarhini AA, Lowder JN, et al. High dose interleukin-2 (Aldesleukin) – expert consensus on best management practices-2014. J Immunother Cancer. 2014 2(1):26.

Elavia N, Panch SR, McManus A, Bikkani T, Szymanski J, Highfill SL, Jin P, Brudno J, Kochenderfer J, Stroncek DF. Effects of starting cellular material composition on chimeric antigen receptor T-cell expansion and characteristics. Transfusion. 2019;59(5):1755-64.

Ellebaek E, Iversen TZ, Junker N, Donia M, Engell-Noerregaard L, Met Ö, et al. Adoptive cell therapy with autologous tumor infiltrating lymphocytes and low-dose Interleukin-2 in metastatic melanoma patients. J Transl Med. 2012;10(1):169.

Ferreria RA, Vastert SJ, Abinun M, Foster HE, Modesto C, Olive T, Kuis W, Wulffraat NM. Hemophagocytosis during fludarabine-based SCT for systemic juvenile idiopathic arthritis. Bone Marrow Transpl. 2006;38(3):249.

Frey NV, Levine BL, Lacey SF, Grupp SA, Maude SL, Schuster SJ, et al. Refractory cytokine release syndrome in recipients of chimeric antigen receptor (CAR) T cells. Blood. 2014;124:2296.

Fryar CD, Gu Q, Ogden CL, Flegal KM. Anthropometric reference data for children and adults: United States, 2011–2014. National Center for Health Statistics. Vital Health Stat 3(39). 2016. Accessed: https://www.cdc.gov/nchs/data/series/sr_03/sr03_039.pdf

Fumoleau P, Largillier R, Clippe C, Dieras V, Orfeuvre H, Lesimple Tet al. Multicentre, phase II study evaluating capecitabine monotherapy in patients with anthracycline-and taxane-pretreated metastatic breast cancer. European Journal of Cancer. 2004;40(4):536-42.

Gardner RA, Finney O, Annesley C, Brakke H, Summers C, Leger K, et al. Intent-to-treat leukemia remission by CD19 CAR T cells of defined formulation and dose in children and young adults. Blood. 2017;129(25):3322-31.

Giantonio BJ, Catalano PJ, Meropol NJ, O'Dwyer PJ, Mitchell EP, Alberts SR, et al. Bevacizumab in combination with oxaliplatin, fluorouracil, and leucovorin (FOLFOX4) for previously treated metastatic colorectal cancer: results from the Eastern Cooperative Oncology Group Study E3200. J Clin Oncol. 2007;25(12):1539.

Gobbini E, Ezzalfani M, Dieras V, et al. : Time trends of overall survival among metastatic breast cancer patients in the real-life ESME cohort. Eur J Cancer. 2018;96:17–24.

Gandhi L, Rodríguez-Abreu D, Gadgeel S, et al. Pembrolizumab plus Chemotherapy in Metastatic Non-Small-Cell Lung Cancer. N Engl J Med 2018; 378:2078.

Gilbert MJ. Severe neurotoxicity in the phase 2 trial of JCAR015 in adult B-ALL (ROCKET study): analyses of patient, protocol and product attributes. J Immunother Cancer. 2017;8:12.

Goldrath AW, Bevan MJ. Selecting and maintaining a diverse T-cell repertoire. Nature1999;402(6759):255.

Gordon AN, Fleagle JT, Guthrie D, Parkin DE, Gore ME, Lacave AJ. Recurrent epithelial ovarian carcinoma: a randomized phase III study of pegylated liposomal doxorubicin versus topotecan. J Clin Oncol. 2001;19(14):3312-22.

Gros A, Parkhurst MR, Tran E, Pasetto A, Robbins PF, Ilyas S, et al. Prospective identification of neoantigen-specific lymphocytes in the peripheral blood of melanoma patients. Nat Med. 2016;22(4):433.

Hacohen N, Fritsch EF, Carter TA, Lander ES, Wu CJ. Getting personal with neoantigen-based therapeutic cancer vaccines. Cancer Immunol Res. 2013;1(1):11-5.

Hales RK, Banchereau J, Ribas A, Tarhini AA, Weber JS, Fox BA, et al. Assessing oncologic benefit in clinical trials of immunotherapy agents. Ann Oncol. 2010;21(10):1944-51.

Hamid O, Robert C, Daud A, Hodi FS, Hwu WJ, Kefford R, et al. Safety and tumor responses with lambrolizumab (anti–PD-1) in melanoma. New Engl J Med. 2013;369(2):134-44.

Herbst RS, Soria JC, Kowanetz M, Fine GD, Hamid O, Gordon MS, et al. Predictive correlates of response to the anti-PD-L1 antibody MPDL3280A in cancer patients. Nature. 2014;515(7528):563.

Hirayama AV, Gauthier J, Hay KA, Sheih A, Cherian S, Chen X, et al. Efficacy and toxicity of JCAR014 in combination with durvalumab for the treatment of patients with relapsed/refractory aggressive B-cell non-Hodgkin lymphoma. Blood. 2018; 132 (Supp 1):1680.

Hodi FS, O'day SJ, McDermott DF, Weber RW, Sosman JA, Haanen JB, et al. Improved survival with ipilimumab in patients with metastatic melanoma. New Engl J Med. 2010;363(8):711-23.

Holtzman NG, El Chaer F, Mohindra P, Badros A, Dahiya S, Hardy NM, et al. Relapsed diffuse large B-cell lymphoma after allogeneic CAR T-cell therapy successfully treated with PD1 inhibition. Biol Blood Marrow Tr. 2019;25(3):S182-3.

Hong DS, Clarke J, Johanns T, Kabriaei P, Heymach JV, Galal A et al. Initial safety, efficacy, and product attributes from the SURPASS trial with ADP-A2M4CD8, a SPEAR T-cell therapy incorporating an affinity optimized TCR targeting MAGE-A4 and a CD8a co-receptor. Society for Immunotherapy of Cancer (SITC), Virtual Congress, November 9-14, 2020.

Hont AB, Cruz CR, Ulrey R, O'Brien B, Stanojevic M, Datar A, et al. Immunotherapy of Relapsed and Refractory Solid Tumors with Ex Vivo Expanded Multi-Tumor Associated Antigen Specific Cytotoxic T Lymphocytes: A Phase I Study. J Clin Oncol. 2019:JCO-19.

Heczey A, Louis CU, Savoldo B, Dakhova O, Durett A, Grilley B, et al. CAR T Cells Administered in Combination with Lymphodepletion and PD-1 Inhibition to Patients with Neuroblastoma. Mol Ther. 2017 Sep 6; 25(9): 2214–2224.

Jacobson CA, Westin JR, Miklos DB, Herrera AF, Lee J, Seng J, et al. Phase 1/2 primary analysis of ZUMA-6: Axicabtagene ciloleucel (Axi-Cel) in combination with atezolizumab (Atezo) for the treatment of patients (Pts) with refractory diffuse large B cell lymphoma (DLBCL). In: Proceedings of the Annual Meeting of the American Association for Cancer Research 2020; 2020 Apr 27-28 and Jun 22-24. Philadelphia (PA): AACR; Cancer Res 2020;80(16 Suppl):Abstract nr CT055.

Jazaeri AA, Zsiros E, Amaria RN, Artz AS, Edwards RP, Wenham RM, et al. Safety and efficacy of adoptive cell transfer using autologous tumor infiltrating lymphocytes (LN-145) for treatment of recurrent, metastatic, or persistent cervical carcinoma. J Clin Oncol. 2019;37(15 suppl): 2538-2538.

Johnson LA, Morgan RA, Dudley ME, Cassard L, Yang JC, Hughes MS, et al. Gene therapy with human and mouse T-cell receptors mediates cancer regression and targets normal tissues expressing cognate antigen. Blood. 2009;114(3):535-46.

Jordan MB, Allen CE, Weitzman S, Filipovich AH, McClain KL. How I treat hemophagocytic lymphohistiocytosis. Blood. 2011;118(15):4041-52.

Kantoff PW, Higano CS, Shore ND, Berger ER, Small EJ, Penson DF, et al. Sipuleucel-T immunotherapy for castration-resistant prostate cancer. New Engl J Med. 2010;363(5):411-22.

Kaufman B, Shapira-Frommer R, Schmutzler RK, Audeh MW, Friedlander M, Balmaña J, et al. Olaparib monotherapy in patients with advanced cancer and a germline BRCA1/2 mutation. J Clin Oncol. 2015;33:244-50.

Khalil DN, Smith EL, Brentjens RJ, Wolchok JD. The future of cancer treatment: immunomodulation, CARs and combination immunotherapy. Nat Rev Clin Oncol. 2016;13(5):273.

Klebanoff CA, Gattinoni L, Restifo NP. Sorting through subsets: which T cell populations mediate highly effective adoptive immunotherapy? J Immunother. 2012;35(9):651.

Klebanoff CA, Khong HT, Antony PA, Palmer DC, Restifo NP. Sinks, suppressors and antigen presenters: how lymphodepletion enhances T cell-mediated tumor immunotherapy. Trends Immunol. 2005;26(2):111-7.

Kochenderfer JN, Dudley ME, Kassim SH, Somerville RP, Carpenter RO, Stetler-Stevenson M, et al. Chemotherapy-refractory diffuse large B-cell lymphoma and indolent B-cell malignancies can be effectively treated with autologous T cells expressing an anti-CD19 chimeric antigen receptor. J Clin Oncol. 2015;33(6):540.

Kontos S, Grimm AJ, Hubbell JA. Engineering antigen-specific immunological tolerance. Curr Opin Immunol. 2015;35:80-8.

La Rosée P. Treatment of hemophagocytic lymphohistiocytosis in adults. ASH Education Program Book. 2015;2015(1):190-6.

Lackner H, Urban C, Sovinz P, Benesch M, Moser A, Schwinger W. Hemophagocytic lymphohistiocytosis as severe adverse event of antineoplastic treatment in children. Haematologica. 2008;93(2):291-4.

Lamers CH, Sleijfer S, Vulto AG, Kruit WH, Kliffen M, Debets R, et al. Treatment of metastatic renal cell carcinoma with autologous T-lymphocytes genetically retargeted against carbonic anhydrase IX: first clinical experience. J Clin Oncol. 2006;24(13):e20-e22.

Larkin J, Minor D, D'Angelo S, Neyns B, Smylie M, Miller W, et al. Overall survival in patients with advanced melanoma who received Nivolumab versus investigator's choice chemotherapy in checkmate 037: a randomized, controlled, open-label Phase III Trial. J Clin Oncol. 2018;36(4):383-390.

Lawrence MS, Stojanov P, Polak P, Kryukov GV, Cibulskis K, Sivachenko A, Carter SL, Stewart C, Mermel CH, Roberts SA, Kiezun A. Mutational heterogeneity in cancer and the search for new cancer-associated genes. Nature. 2013;499(7457):214.

Le DT, Uram JN, Wang H, Bartlett BR, Kemberling H, Eyring AD, et al. PD-1 blockade in tumors with mismatch-repair deficiency. New Engl J Med. 2015;372(26):2509-20.

Lee DW, Gardner R, Porter DL, Louis CU, Ahmed N, Jensen M, et al. Current concepts in the diagnosis and management of cytokine release syndrome. Blood. 2014;124(2):188-95.

Lee DW, Kochenderfer JN, Stetler-Stevenson M, Cui YK, Delbrook C, Feldman SA, et al. T cells expressing CD19 chimeric antigen receptors for acute lymphoblastic leukaemia in children and young adults: a phase 1 dose-escalation trial. Lancet. 2015;385(9967):517-28.

Lee DW, Santomasso BD, Locke FL, Ghobadi A, Turtle CJ, Brudno JN, et al. ASBMT consensus grading for cytokine release syndrome and neurological toxicity associated with immune effector cells. Biol Blood Marrow Transplant. 2018.

Lee DW, Stetler-Stevenson M, Yuan CM, Shah NN, Delbrook C, Yates B, et al. Long-term outcomes following CD19 CAR T cell therapy for B-ALL are superior in patients receiving a fludarabine/cyclophosphamide preparative regimen and post-CAR hematopoietic stem cell transplantation. Blood. 2016;218.

Lee JJ, Sun W. Options for second-line treatment in metastatic colorectal cancer. Clin Adv Hematol Oncol. 2016;14(1):46-54.

Lee V, Murphy A, Le DT, Diaz LA. Mismatch repair deficiency and response to immune checkpoint blockade. Oncologist. 2016;21(10):1200-11.

Leisegang M, Engels B, Schreiber K, Yew PY, Kiyotani K, Idel C, et al. Eradication of large solid tumors by gene therapy with a T-cell receptor targeting a single cancer-specific point mutation. Clin Cancer Res. 2016;22(11):2734-43.

Leon LF, Golsorkhi A, Liu S, Drozdowskyj A, Rosell R. Overall survival analyses of first-line erlotinib versus chemotherapy in the EURTAC study population controlling for the use of post-study therapy. Ann Oncol. 2014; 25 (abstract 1273P): iv426-iv470.

Li AM, Hucks GE, Dinofia AM, Seif AE, Teachey DT, Baniewicz D, et al. Checkpoint Inhibitors Augment CD19-Directed Chimeric Antigen Receptor (CAR) T Cell Therapy in Relapsed B-Cell Acute Lymphoblastic Leukemia. Blood. 2018;132 Suppl 1:556.

Liang X, Potter J, Kumar S, Zou Y, Quintanilla R, Sridharan M, et al. Rapid and highly efficient mammalian cell engineering via Cas9 protein transfection. Journal Biotechnol. 2015;208:44-53.

Linette GP, Stadtmauer EA, Maus MV, Rapoport AP, Levine BL, Emery L, et al. Cardiovascular toxicity and titin cross-reactivity of affinity-enhanced T cells in myeloma and melanoma. Blood. 2013;122(6):863-71.

Long GV, Tykodi SS, Schneider JG, Garbe C, Gravis G, Rashford M, et al. Assessment of nivolumab exposure and clinical safety of 480 mg every 4 weeks flat-dosing schedule in patients with cancer. Ann Oncol. 2018;29(11):2208-13.

Loriot Y, Bianchini D, Ileana E, Sandhu S, Patrikidou A, Pezaro C, et al. Antitumour activity of abiraterone acetate against metastatic castration-resistant prostate cancer progressing after docetaxel and enzalutamide (MDV3100). Ann Oncol. 2013;24(7):1807-12.

Mackensen A, Meidenbauer N, Vogl S, Launer M, Berger J, Andreesen R. Phase I Study of Adoptive T-Cell Therapy Using Antigen-Specific CD8+ T Cells for the Treatment of Patients with Metastatic Melanoma. J Clin Oncol. 2006;24(31):5060-9.

Mathijssen RH, De Jong FA, Loos WJ, Van Der Bol JM, Verweij J, Sparreboom A. Flat-fixed dosing versus body surface area–based dosing of anticancer drugs in adults: does it make a difference? Oncologist. 2007;12(8):913-23.

Maude SL, Frey N, Shaw PA, Aplenc R, Barrett DM, Bunin NJ, et al. Chimeric antigen receptor T cells for sustained remissions in leukemia. New Engl J Med. 2014;371(16):1507-17.

Maude SL, Laetsch TW, Buechner J, Rives S, Boyer M, Bittencourt H, et al. Tisagenlecleucel in children and young adults with B-cell lymphoblastic leukemia. New Engl J Med. 2018;378(5):439-48.

Maude SL, Teachey DT, Porter DL, Grupp SA. CD19-targeted chimeric antigen receptor T-cell therapy for acute lymphoblastic leukemia. Blood. 2015;125(26):4017-23.

Martin PJ, Rizzo JD, Wingard JR, Ballen K, Curtin PT, Cutler C, et al. First-and second-line systemic treatment of acute graft-versus-host disease: recommendations of the American Society of Blood and Marrow Transplantation. Biol Blood Marrow Tr. 2012;18(8):1150-63.

McGranahan N, Rosenthal R, Hiley CT, Rowan AJ, Watkins TB, Wilson GA, et al. Allele-specific HLA loss and immune escape in lung cancer evolution. Cell. 2017;171(6):1259-71.

Mezynski J, Pezaro C, Bianchini D, Zivi A, Sandhu S, Thompson E, et al. Antitumour activity of docetaxel following treatment with the CYP17A1 inhibitor abiraterone: clinical evidence for cross-resistance? Ann Oncol. 2012;23(11):2943-7.

Miles DW, Diéras V, Cortés J, et al. : First-line bevacizumab in combination with chemotherapy for HER2-negative metastatic breast cancer: pooled and subgroup analyses of data from 2447 patients. Ann Oncol. 2013;24(11):2773–80.

Morgan RA, Dudley ME, Wunderlich JR, Hughes MS, Yang JC, Sherry RM, et al. Cancer regression in patients after transfer of genetically engineered lymphocytes. Science. 2006;314(5796):126-9.

Mueller KT, Maude SL, Porter DL, Frey N, Wood P, Han X, et al. Cellular kinetics of CTL019 in relapsed/refractory B-cell acute lymphoblastic leukemia and chronic lymphocytic leukemia. Blood. 2017;130(21):2317-25.

Mueller KT, Waldron E, Grupp SA, Levine JE, Laetsch TW, Pulsipher MA, et al. Clinical pharmacology of tisagenlecleucel in B-cell acute lymphoblastic leukemia. Clin Cancer Res. 2018;24(24):6175-84.

Mutch DG, Orlando M, Goss T, Teneriello MG, Gordon AN, McMeekin SD, et al. Randomized phase III trial of gemcitabine compared with pegylated liposomal doxorubicin in patients with platinum-resistant ovarian cancer. J Clin Oncol. 2007;25(19):2811-8.

Neelapu SS, Locke FL, Bartlett NL, Lekakis LJ, Miklos DB, Jacobson CA, et al. Axicabtagene ciloleucel CAR-T-cell therapy in refractory large B-cell lymphoma. New Eng J Med. 2017;377(26):2531-44.

Nguyen LT, Saibil SD, Sotov V, Le MX, Khoja L, Ghazarian D, et al. Phase II clinical trial of adoptive cell therapy for patients with metastatic melanoma with autologous tumor-infiltrating lymphocytes and low-dose interleukin-2. Cancer Immunol Immunother. 2019;68(5):773-85.

Ninomiya S, Narala N, Huye L, Yagyu S, Savoldo B, Dotti G, et al. Tumor indoleamine 2, 3-dioxygenase (IDO) inhibits CD19-CAR T cells and is downregulated by lymphodepleting drugs. Blood. 2015;125(25):3905-16.

Noonan KL, North S, Bitting RL, Armstrong AJ, Ellard SL, Chi KN. Clinical activity of abiraterone acetate in patients with metastatic castration-resistant prostate cancer progressing after enzalutamide. Ann Oncol. 2013;24(7):1802-7.

Norberg SM, Nagarsheth N, Doran S, Kanakry JA, Adhikary S, Schweitzer C, et al. Regression of Epithelial Cancers Following T Cell Receptor Gene Therapy Targeting Human Papillomavirus. ASH Annual Meeting 2018.

Nowicki TS, Berent-Maoz B, Huang RR, Wang X, Cheung-Lau G, Kaplan-Lefko P, et al. Abstract CT008: A pilot trial of the combination of transgenic NY-ESO-1-reactive adoptive cellular therapy with dendritic cell vaccination with or without ipilimumab in patients with sarcoma and melanoma. Cancer Res. 2018;78(13 Suppl): CT008.

Oncologic Drugs Advisory Committee Meeting. Tisagenlecleucel: Novartis Pharmaceuticals Corporation. FDA Briefing Document. https://www.fda.gov/downloads/AdvisoryCommittees/CommitteesMeetingMaterials/Drugs/OncologicDrugsAdvisoryCommittee/UCM566166.pdf

O'Shaughnessy J, Miles D, Vukelja S, Moiseyenko V, Ayoub JP, Cervantes G, et al. Superior survival with capecitabine plus docetaxel combination therapy in anthracycline-pretreated patients with advanced breast cancer: phase III trial results. J Clin Onc. 2002;20(12):2812-23.

Ott PA, Hu Z, Keskin DB, Shukla SA, Sun J, Bozym DJ, et al. An immunogenic personal neoantigen vaccine for patients with melanoma. Nature. 2017;547(7662):217.

Ou SI, Gadgeel SM, Barlesi F, Yang JC, De Petris L, Kim DW. Pooled overall survival and safety data from the pivotal phase II studies (NP28673 and NP28761) of alectinib in ALK-positive non-small-cell lung cancer. Lung Cancer. 2020;139:22-27.

Park JH, Rivière I, Gonen M, Wang X, Sénéchal B, Curran KJ, et al. Long-term follow-up of CD19 CAR-Therapy in acute lymphoblastic leukemia. New Engl J Med. 2018;378(5):449-59.

Park S, Keam B, Kim SH, Kim KH, Kim YJ, Kim JS, et al. Pemetrexed singlet versus nonpemetrexed-based platinum doublet as second-line chemotherapy after first-line epidermal growth factor receptor (EGFR) tyrosine kinase inhibitor failure in non-small cell lung cancer patients with EGFR mutations. Cancer Res Treat. 2015;47(4):630.

Paulson KG, Voillet V, McAfee MS, Hunter DS, Wagener FD, Perdicchio M, et al. Acquired cancer resistance to combination immunotherapy from transcriptional loss of class I HLA. Nature communications. 2018;9(1):3868.

Porter DL, Hwang WT, Frey NV, Lacey SF, Shaw PA, Loren AW, et al. Chimeric antigen receptor T cells persist and induce sustained remissions in relapsed refractory chronic lymphocytic leukemia. Sci Transl Med. 2015;7(303):303ra139.

Pujade-Lauraine E, Hilpert F, Weber B, Reuss A, Poveda A, Kristensen G, et al. Bevacizumab combined with chemotherapy for platinum-resistant recurrent ovarian cancer: The AURELIA open-label randomized phase III trial. J Clin Oncol. 2014;32(13):1302-08.

Rabinstein AA. Treatment of *cerebral* edema. Neurologist. 2006;12(2):59-73.

Radvanyi LG, Bernatchez C, Zhang M, Fox PS, Miller P, Chacon J, et al. Specific lymphocyte subsets predict response to adoptive cell therapy using expanded autologous tumor-infiltrating lymphocytes in metastatic melanoma patients. Clin Cancer Res. 2012;18(24):6758-70.

Ramalingam SS, Vansteenkiste J, Planchard D, Cho BC, Gray JE, Ohe Y. Overall Survival with Osimertinib in Untreated, EGFR-Mutated Advanced NSCLC. N Engl J Med. 2020;382(1):41-50.

Ramos CA, Bilgi M, Gerken C, Dakhova O, Mei Z, Wu MF, et al. CD30-Chimeric Antigen Receptor (CAR) T Cells for Therapy of Hodgkin Lymphoma (HL) [abstract]. Translplantation & Cellular Therapy Meetings. February 20-24, 2019. Houston, Texas. Abstract 79.

Rapoport AP, Stadtmauer EA, Binder-Scholl GK, Goloubeva O, Vogl DT, Lacey SF, et al. NY-ESO-1–specific TCR–engineered T cells mediate sustained antigen-specific antitumor effects in myeloma. Nat Med. 2015;21(8):914.

Reck M, Rodríguez-Abreu D, Robinson AG, et al. Pembrolizumab versus Chemotherapy for PD-L1-Positive Non-Small-Cell Lung Cancer. N Engl J Med 2016; 375:1823.

Riaz N, Havel JJ, Makarov V, Desrichard A, Urba WJ, Sims JS, et al. Tumor and microenvironment evolution during immunotherapy with nivolumab. Cell. 2017;171(4):934-49.

Ribas A. Tumor immunotherapy directed at PD-1. N Engl J Med. 2012;366(26):2517-9.

Rizvi NA, Hellmann MD, Snyder A, Kvistborg P, Makarov V, Havel JJ, et al. Mutational landscape determines sensitivity to PD-1 blockade in non–small cell lung cancer. Science. 2015;348(6230):124-8.

Robbins PF, Lu YC, El-Gamil M, Li YF, Gross C, Gartner J, et al. Mining exomic sequencing data to identify mutated antigens recognized by adoptively transferred tumor-reactive T cells. Nat Med. 2013;19(6):747.

Robbins PF, Morgan RA, Feldman SA, Yang JC, Sherry RM, Dudley ME, Wunderlich JR, Nahvi AV, Helman LJ, Mackall CL, Kammula US. Tumor regression in patients with metastatic synovial cell sarcoma and melanoma using genetically engineered lymphocytes reactive with NY-ESO-1. J Clin Oncol. 2011;29(7):917.

Robert C, Schachter J, Long GV, Arance A, Grob JJ, Mortier L, et al. Pembrolizumab versus ipilimumab in advanced melanoma. New Engl J Med 2015;372(26):2521-32.

Roché H, Vahdat LT. Treatment of metastatic breast cancer: second line and beyond. Ann Oncol. 2011;22(5):1000-10.

Rodler E, Korde L, Gralow J. Current treatment options in triple negative breast cancer. Breast Dis. 2010;31(1-2):99-12.

Roh W, Chen PL, Reuben A, Spencer CN, Prieto PA, Miller JP, et al. Integrated molecular analysis of tumor biopsies on sequential CTLA-4 and PD-1 blockade reveals markers of response and resistance. Sci Transl Med. 2017;9(379):3560.

Rosell R, Carcereny E, Gervais R, et al. Erlotinib versus standard chemotherapy as first-line treatment for European patients with advanced EGFR mutation-positive non-small-cell lung cancer (EURTAC): a multicentre, open-label, randomised phase 3 trial. Lancet Oncol. 2012; 13: 239-246

Rosenberg SA, Dudley ME. Adoptive cell therapy for the treatment of patients with metastatic melanoma. Curr Opin Immunol. 2009;21(2):233-40.

Rosenberg SA, Lotze MT, Muul LM, Leitman S, Chang AE, Ettinghausen SE, et al. Observations on the systemic administration of autologous lymphokine-activated killer cells and recombinant interleukin-2 to patients with metastatic cancer. N Engl J Med. 1985;313(23):1485-92.

Rosenberg SA, Restifo NP. Adoptive cell transfer as personalized immunotherapy for human cancer. Science. 2015;348(6230):62-8.

Rosenberg SA, Yang JC, Topalian SL, Schwartzentruber DJ, Weber JS, Parkinson DR, et al. Treatment of 283 consecutive patients with metastatic melanoma or renal cell cancer using high-dose bolus interleukin 2. JAMA. 1994;271(12):907-13.

Rosenberg SA, Yannelli JR, Yang JC, Topalian SL, Schwartzentruber DJ, Weber JS, et al. Treatment of patients with metastatic melanoma with autologous tumor-infiltrating lymphocytes and interleukin 2. J Natl Cancer Inst. 1994;86(15):1159-66.

Rosenberg SA. IL-2: the first effective immunotherapy for human cancer. J Immunol. 2014;192(12):5451-8.

Rossi J, Paczkowski P, Shen YW, Morse K, Flynn B, Kaiser A, et al. Preinfusion polyfunctional anti-CD19 chimeric antigen receptor T cells are associated with clinical outcomes in NHL. Blood. 2018;132(8):804-14.

Rupp LJ, Schumann K, Roybal KT, Gate RE, Chun JY, Lim WA, et al. CRISPR/Cas9-mediated PD-1 disruption enhances anti-tumor efficacy of human chimeric antigen receptor T cells. Sci Rep. 2017;7(1):737.

Sarnaik AA, Khushalani NI, Chesney JA, Kluger HM, Curti BD, Lewis KD, et al. Safety and efficacy of cryopreserved autologous tumor infiltrating lymphocyte therapy (LN-144, lifileucel) in advanced metastatic melanoma patients following progression on checkpoint inhibitors. In 33rd annual meeting & pre-conference programs of the Society for Immunotherapy of Cancer (SITC 2018) 2018 Nov 7.

Sabatino M, Hu J, Sommariva M, Gautam S, Fellowes V, Hocker JD, et al. Generation of clinical-grade CD19-specific CAR-modified CD8+ memory stem cells for the treatment of human B-cell malignancies. Blood. 2016;128(4):519-28.

Sanghavi K, Wiseman A, Kirstein MN, Cao Q, Brundage R, Jensen K, et al. Personalized fludarabine dosing to reduce nonrelapse mortality in hematopoietic stem-cell transplant recipients receiving reduced intensity conditioning. Transl Res. 2016 Sep;175:103-115.

Sahin U, Türeci Ö. Personalized vaccines for cancer immunotherapy. Science. 2018;359(6382):1355-60.

Schadendorf D, Hodi FS, Robert C, Weber JS, Margolin K, Hamid O, et al. Pooled analysis of long-term survival data from phase II and phase III trials of ipilimumab in unresectable or metastatic melanoma. J Clin Oncol. 2015;33(17):1889.

Scher HI, Fizazi K, Saad F, Taplin ME, Sternberg CN, Miller K, et al. Increased survival with enzalutamide in prostate cancer after chemotherapy. New Engl J Med. 2012;367(13):1187-97.

Schmid P, Adams S, Rugo HS, et al. : Atezolizumab and Nab-Paclitaxel in Advanced Triple-Negative Breast Cancer. N Engl J Med. 2018;379:2108–21.

Schrader AJ, Boegemann M, Ohlmann CH, Schnoeller TJ, Krabbe LM, Hajili T, et al. Enzalutamide in castration-resistant prostate cancer patients progressing after docetaxel and abiraterone. Eur Urol. 2014;65(1):30-6.

Schreier DJ. Engineering an Immunity to Cancer: A New Era of Adoptive Cellular Therapy with Tisagenlecleucel in Pediatric ALL. Pharmacy Grand Rounds; October 17, 2017; Department of Pharmacy, Mayo Clinic.

Schumacher TN, Schreiber RD. Neoantigens in cancer immunotherapy. Science. 2015;348(6230):69-74.

Schuster SJ, Bishop MR, Tam CS, Waller EK, Borchmann P, McGuirk JP, et al. Tisagenlecleucel in adult relapsed or refractory diffuse large B-cell lymphoma. New Engl J Med. 2019;380(1):45-56.

Schuster SJ, Svoboda J, Chong EA, Nasta SD, Mato AR, Anak Ö, et al. Chimeric antigen receptor T cells in refractory B-cell lymphomas. New Engl J Med. 2017;377(26):2545-54.

Schwartz RN, Stover L, Dutcher JP. Managing toxicities of high-dose interleukin-2. Oncology (Williston Park). 2002;16(11 Suppl 13):11-20.

Schwartzentruber DJ. Guidelines for the safe administration of high-dose interleukin-2. J Immunother. 2001;24(4):287-93.

Shimabukuro-Vornhagen A, Gödel P, Subklewe M, Stemmler HJ, Schlößer HA, Schlaak M, et al. Cytokine release syndrome. J Immunother Cancer. 2018;6(1):56.

Siegel RL, Miller KD, Jemal A. Cancer statistics, 2018. CA Cancer J Clin. 2018;68(1):7-30.

Smaglo BG, Hwang J. Continuum of care with anti-angiogenic therapies in metastatic colorectal cancer. J Gastrointest Oncol. 2013;4(3):299.

Snyder A, Makarov V, Merghoub T, Yuan J, Zaretsky JM, Desrichard A, et al. Genetic basis for clinical response to CTLA-4 blockade in melanoma. New Engl J Med. 2014;371(23):2189-99.

Socinski MA, Jotte RM, Cappuzzo F, et al. Atezolizumab for First-Line Treatment of Metastatic Nonsquamous NSCLC. N Engl J Med 2018; 378:2288.

Sollid LM, Iversen R, Steinsbø Ø, Qiao SW, Bergseng E, Dørum S, et al. Small bowel, celiac disease and adaptive immunity. Digestive Dis. 2015;33(2):115-21.

Solomon BJ, Kim DW, Wu YL, Nakagawa K, Mekhail T, Felip E et al. Final Overall Survival Analysis from a Study Comparing First-Line Crizotinib Versus Chemotherapy in ALK-Mutation-Positive Non-Small-Cell Lung Cancer. J Clin Oncol. 2018;36(22):2251-2258.

Stadtmauer EA, Fraietta JA, Davis MM, Cohen AD, Weber KL, Lancaster E et al. CRISPR-engineered T cells in patients with refractory cancer. Science. 2020 Feb 28;367(6481)

Stemberger C, Graef P, Odendahl M, Albrecht J, Dössinger G, Anderl F, et al. Lowest numbers of primary CD8+ T cells can reconstitute protective immunity upon adoptive immunotherapy. Blood. 2014;124(4):628-37.

Stevanović S, Pasetto A, Helman SR, Gartner JJ, Prickett TD, Howie B, Robins HS, Robbins PF, Klebanoff CA, Rosenberg SA, Hinrichs CS. Landscape of immunogenic tumor antigens in successful immunotherapy of virally induced epithelial cancer. Science. 2017 Apr 14;356(6334):200-5.

Stone JD, Aggen DH, Schietinger A, Schreiber H, Kranz DM. A sensitivity scale for targeting T cells with chimeric antigen receptors (CARs) and bispecific T-cell Engagers (BiTEs). Oncoimmunology. 2012;1(6):863-73.

Surh CD, Sprent J. Homeostatic T cell proliferation: how far can T cells be activated to self-ligands? J Exp Med. 2000;192(4):F9-14.

Surveillance, Epidemiology, and End Results (SEER) Program. 2008–2014.

Teachey DT, Bishop MR, Maloney DG, Grupp SA. Toxicity management after chimeric antigen receptor T-cell therapy: one size does not fit 'ALL'. Nat Rev Clin Oncol. 2018;15(4):218.

Teachey DT, Rheingold SR, Maude SL, Zugmaier G, Barrett DM, Seif AE, et al. Cytokine release syndrome after blinatumomab treatment related to abnormal macrophage activation and ameliorated with cytokine-directed therapy. Blood. 2013;121(26):5154-7.

Thomas ES, Gomez HL, Li RK, Chung HC, Fein LE, Chan VF, et al. Ixabepilone plus capecitabine for metastatic breast cancer progressing after anthracycline and taxane treatment. J Clin Oncol. 2008;26(13):2223.

Topalian SL, Hodi FS, Brahmer JR, Gettinger SN, Smith DC, McDermott DFet al. Safety, activity, and immune correlates of anti–PD-1 antibody in cancer. New Engl J Med. 2012;366(26):2443-54.

Topalian SL, Solomon D, Avis FP, Chang AE, Freerksen DL, Linehan WM, et al. Immunotherapy of patients with advanced cancer using tumor-infiltrating lymphocytes and recombinant interleukin-2: a pilot study. J Clin Oncol. 1988;6(5):839-53.

Tran E, Ahmadzadeh M, Lu YC, Gros A, Turcotte S, Robbins PF, et al. Immunogenicity of somatic mutations in human gastrointestinal cancers. Science. 2015;350(6266):1387-90.

Tran E, Robbins PF, Lu YC, Prickett TD, Gartner JJ, Jia L, et al. T-cell transfer therapy targeting mutant KRAS in cancer. New Engl J Med. 2016;375(23):2255-62.

Tran E, Robbins PF, Rosenberg SA. 'Final common pathway' of human cancer immunotherapy: targeting random somatic mutations. Nat Immunol. 2017;18(3):255.

Tran E, Turcotte S, Gros A, Robbins PF, Lu YC, Dudley ME, et al. Cancer immunotherapy based on mutation-specific CD4+ T cells in a patient with epithelial cancer. Science. 2014;344(6184):641-5.

Tumeh PC, Harview CL, Yearley JH, Shintaku IP, Taylor EJ, Robert L, et al. PD-1 blockade induces responses by inhibiting adaptive immune resistance. Nature. 2014;515(7528):568.

Turtle CJ, Hanafi LA, Berger C, Hudecek M, Pender B, Robinson E, et al. Immunotherapy of non-Hodgkin's lymphoma with a defined ratio of CD8+ and CD4+ CD19-specific chimeric antigen receptor–modified T cells. Science translational medicine. 2016;8(355):355ra116.

Turtle CJ, Hay KA, Hanafi LA, Li D, Cherian S, Chen X, et al. Durable molecular remissions in chronic lymphocytic leukemia treated with CD19-specific chimeric antigen receptor-modified T cells after failure of ibrutinib. J Clin Oncol. 2017;35(26):3010-20.

Van Allen EM, Miao D, Schilling B, Shukla SA, Blank C, Zimmer L, et al. Genomic correlates of response to CTLA-4 blockade in metastatic melanoma. Science. 2015;350(6257):207-11.

Van Cutsem E, Tabernero J, Lakomy R, Prenen H, Prausová J, Macarulla T, et al. Addition of aflibercept to fluorouracil, leucovorin, and irinotecan improves survival in a phase III randomized trial in patients with metastatic colorectal cancer previously treated with an oxaliplatin-based regimen. J Clin Oncol. 2012;30(28):3499-506.

Verdegaal EM, De Miranda NF, Visser M, Harryvan T, Van Buuren MM, Andersen RS, et al. Neoantigen landscape dynamics during human melanoma–T cell interactions. Nature. 2016;536(7614):91.

Watanabe K, Kuramitsu S, Posey Jr AD, June CH. Expanding the Therapeutic Window for CAR T-cell therapy in Solid Tumors: The Knowns and Unknowns of CAR T Cell Biology. Front Immunol. 2018;9.

Weber JS, D'Angelo SP, Minor D, Hodi FS, Gutzmer R, Neyns B, et al. Nivolumab versus chemotherapy in patients with advanced melanoma who progressed after anti-CTLA-4 treatment (CheckMate 037): a randomised, controlled, open-label, phase 3 trial. Lancet Oncol. 2015;16(4):375-84.

Wherry EJ, Ha SJ, Kaech SM, Haining WN, Sarkar S, Kalia V, et al. Molecular signature of CD8+ T cell exhaustion during chronic viral infection. Immunity. 2007;27(4):670-84.

Wolchok JD, Kluger H, Callahan MK, Postow MA, Rizvi NA, Lesokhin AM, Segal NH, Ariyan CE, Gordon RA, Reed K, Burke MM. Nivolumab plus ipilimumab in advanced melanoma. New England Journal of Medicine. 2013;369(2):122-33.

Yardley DA, Coleman R, Conte P, et al. : nab-Paclitaxel plus carboplatin or gemcitabine versus gemcitabine plus carboplatin as first-line treatment of patients with triple-negative metastatic breast cancer: results from the tnAcity trial. Ann Oncol. 2018;29(8):1763–70.

Yee C, Thompson JA, Byrd D, Riddell SR, Roche P, Celis E, et al. Adoptive T-cell therapy using antigen-specific CD8+ T cell clones for the treatment of patients with metastatic melanoma: *in vivo* persistence, migration, and antitumor effect of transferred T cells. Proceedings of the National Academy of Sciences. 2002;99(25):16168-73.

Yoon D, Osborn M, Tolar J, Kim C. Incorporation of immune checkpoint blockade into chimeric antigen receptor T cells (CAR-Ts): combination or built-in CAR-T. Int J Mol Sci. 2018;19(2):340.

Zacharakis N, Chinnasamy H, Black M, Xu H, Lu YC, Zheng Z, et al. Immune recognition of somatic mutations leading to complete durable regression in metastatic breast cancer. Nature medicine. 2018;24(6):724.

Zhou X, Wu J, Duan C, Liu Y. Retrospective Analysis of Adoptive TIL Therapy plus Anti-PD1 Therapy in Patients with Chemotherapy-Resistant Metastatic Osteosarcoma. J Immunol Res. 2020 Oct 1;2020:7890985.
