# Supplementary material for: Non-viral precision T cell receptor replacement for personalized cell therapy
Source: Nature. 2022 Nov 10;615(7953):687–96. doi: 10.1038/s41586-022-05531-1 (PMC9768791; doi:10.1038/s41586-022-05531-1)
Supplement: Supplementary file 2 — Reporting Summary [file 41586_2022_5531_MOESM2_ESM.pdf]

nature portfolio

Corresponding author(s): Antoni Ribas

Last updated by author(s): Dec 15, 2022

Reporting Summary

Nature Portfolio wishes to improve the reproducibility of the work that we publish. This form provides structure for consistency and transparency in reporting. For further information on Nature Portfolio policies, see our Editorial Policies and the Editorial Policy Checklist.

Statistics

For all statistical analyses, confirm that the following items are present in the figure legend, table legend, main text, or Methods section.

n/a	Confirmed	
☐	☒	The exact sample size (*n*) for each experimental group/condition, given as a discrete number and unit of measurement
☐	☒	A statement on whether measurements were taken from distinct samples or whether the same sample was measured repeatedly
☐	☒	The statistical test(s) used AND whether they are one- or two-sided<br>*Only common tests should be described solely by name; describe more complex techniques in the Methods section.*
☒	☐	A description of all covariates tested
☐	☒	A description of any assumptions or corrections, such as tests of normality and adjustment for multiple comparisons
☐	☒	A full description of the statistical parameters including central tendency (e.g. means) or other basic estimates (e.g. regression coefficient) AND variation (e.g. standard deviation) or associated estimates of uncertainty (e.g. confidence intervals)
☐	☒	For null hypothesis testing, the test statistic (e.g. *F*, *t*, *r*) with confidence intervals, effect sizes, degrees of freedom and *P* value noted<br>*Give P values as exact values whenever suitable.*
☒	☐	For Bayesian analysis, information on the choice of priors and Markov chain Monte Carlo settings
☒	☐	For hierarchical and complex designs, identification of the appropriate level for tests and full reporting of outcomes
☐	☒	Estimates of effect sizes (e.g. Cohen's *d*, Pearson's *r*), indicating how they were calculated

Our web collection on statistics for biologists contains articles on many of the points above.

Software and code

Policy information about availability of computer code

Data collection	Flow cytometry data was collected using BD FACSDiva (V8.0.3) and analysed with FlowJo (V10.7.1 or V10.8.1), or FCS Express (V6.6.21.0). Serum cytokine analysis was performed using a MESO QuickPlex SQ 120 instrument and Discovery Workbench 4.0 software.
Data analysis	The following software was used for analysis (Version and or location listed in parenthesis): OptiType (1.3.4), netMHCpan (3.0, 4.0, or 4.1 as indicated in the methods), RSEM (1.3.3), STAR (2.7.6a), MiXCR (2.1.3), VarDictJava (1.8.2), VarScan (2.4.4), Sentieon (BWA, 201911.01), Sequenza (3.0), Strelka2 (2.9.10), Mutect (3.1-0-g72492bb), MuTect2 (4.1.8.1), pyClone (0.13.1), Ensembl (release 101, http://ensembl.org/), GraphPad Prism (9.4.1).

For manuscripts utilizing custom algorithms or software that are central to the research but not yet described in published literature, software must be made available to editors and reviewers. We strongly encourage code deposition in a community repository (e.g. GitHub). See the Nature Portfolio guidelines for submitting code & software for further information.

Data

Policy information about availability of data

All manuscripts must include a data availability statement. This statement should provide the following information, where applicable:

- Accession codes, unique identifiers, or web links for publicly available datasets
- A description of any restrictions on data availability
- For clinical datasets or third party data, please ensure that the statement adheres to our policy

The following publicly available data sets were utilised: ExAc (3.1, https://gnomad.broadinstitute.org/downloads#exac-variants), dbSNP (v146, ftp://ftp.broadinstitute.org/bundle), GATK Resource Bundle (hg19/Grch37, ftp://ftp.broadinstitute.org/bundle), Human Proteome (Homo_sapiens.GRCh37.75.pep.all.fa, http://ensembl.org/), IMGT (TCR/HLA, 3.1.17, http://www.imgt.org/), RefSeq (1052019, ftp://hgdownload.cse.ucsc.edu/goldenPath), TCGA (Version 1.0, https://portal.gdc.cancer.gov/), Broad Institute (hg19, ftp://ftp.broadinstitute.org/bundle). The TCR sequences from the present study are available in the article supplemental files, and the genomics data is available on reasonable request from the European Genome-Phenome Archive (EGA) repository.

Human research participants

Policy information about studies involving human research participants and Sex and Gender in Research.

Reporting on sex and gender	Patients were screened and enrolled on this study irrespective of their sex/gender. Any data regarding a patient's sex and gender was collected and provided to the sponsor by the treating physician and PI for the study at each site. No sex- or gender-based analysis has been conducted in this small dataset.
Population characteristics	Provided in extended data table 3.
Recruitment	Patients were recruited across 9 clinical investigational sites. Given the phase 1 nature and complexity of the study, the sites were limited to the United States of America. There were no biases introduced and patients were screened on a first-come first-serve basis based on meeting the protocol inclusion-exclusion criteria. The principal investigators identified patients based on the inclusion exclusion criteria and contacted the PACT medical monitor to confirm if an informed consent form could be signed. No protocol waivers were allowed on this study.
Ethics oversight	The trial was conducted in accordance with the principles of the Declaration of Helsinki. The trial protocol and statistical analysis plan were designed in a collaboration between the sponsor (PACT Pharma Inc.) and the authors. The protocol was approved by the institutional review board from each clinical site enrolling patients: City of Hope, Duarte California; University of California Los Angeles, Los Angeles California; University of California, Irvine Medical Center, Orange, California; University of California, Davis, Sacramento California; University of California, San Francisco, San Francisco California; Northwestern University Medical Center, Chicago Illinois; Memorial Sloan Kettering Cancer Center, New York, New York; Tennessee Oncology, Nashville, Tennessee; and Fred Hutchinson Cancer Research Center, Seattle, Washington.

Note that full information on the approval of the study protocol must also be provided in the manuscript.

Field-specific reporting

Please select the one below that is the best fit for your research. If you are not sure, read the appropriate sections before making your selection.

☒ Life sciences ☐ Behavioural & social sciences ☐ Ecological, evolutionary & environmental sciences

For a reference copy of the document with all sections, see nature.com/documents/nr-reporting-summary-flat.pdf

Life sciences study design

All studies must disclose on these points even when the disclosure is negative.

Sample size	Up to approximately 76 evaluable participants will be enrolled into the Initial Phase. The planned enrollment for the Expansion Phase study is potentially up to 112 participants, depending on the number and size of the cohorts. The total anticipated enrollment in this study is approximately 9–188 participants.<br>Three to 12 participants will be enrolled into each dose level cohort in the Phase 1a portion of the study. If the study proceeds to the dose-expansion basket cohorts in the Phase 1a, up to 40 additional participants may be enrolled (up to 20 each in the TCR alone and TCR + IL-2 baskets).<br>The dose-escalation stage sample size was based on the probability of not observing any DLTs in 3 participants, and the probability of observing fewer than 2 DLTs in 6 participants for underlying DLT rates during the dose-escalation stage.
Data exclusions	Patients went through a screening process for TCR selection and cell therapy manufacture. Patients were excluded from dosing if they failed eligibility criteria or if a product could not be manufactured. Only products manufactured using version 2.0 or 2.1 were included in the analysis. No primary or secondary endpoint data were excluded from the analysis. All available manufactured products and PBMCs were analysed. Two post-infusion biopsies were excluded from analysis due to insufficient tumour content.

Replication	The NeoTCR-P1 is an autologous TCR therapy manufactured with the patients' own PBMCs and their own unique TCRs. The cell therapies were manufactured with up to three independent lots consisting of three unique TCRs or, in some cases, a single TCR, giving technical replicates of the manufacturing for an individual patient. The study included treatment of human participants with a personalized NeoTCR-P1 cell therapy product. Due to the disease characteristics and personalized nature of the cell therapy product, replication of the findings may vary depending on the disease state and the neoTCR selected for infusion. Flow cytometry experiments to analyse the final cell product or post-infusion PBMC samples were performed in duplicate, if there were enough cells available, and all attempts at replication were successful.
Randomization	This was a Phase 1a, open label, 3+3 dose escalation trial design trial design to evaluate NeoTCR-P1 infused as a single agent without or with IL-2, or in combination with nivolumab. Patients were not randomized and were enrolled at the maximum open dose level during the trial, if enough cells were manufactured to meet a given dose level. Once a dose level was cleared, patients and their treating physician had the option to administer the NeoTCR-P1 cell therapy in combination with IL-2. No patients were treated in combination with nivolumab.
Blinding	This was a single arm, open label trial design, thus blinding is not relevant to the study.

Reporting for specific materials, systems and methods

We require information from authors about some types of materials, experimental systems and methods used in many studies. Here, indicate whether each material, system or method listed is relevant to your study. If you are not sure if a list item applies to your research, read the appropriate section before selecting a response.

Materials & experimental systems

n/a	Involved in the study
☐	☒ Antibodies
☐	☒ Eukaryotic cell lines
☒	☐ Palaeontology and archaeology
☒	☐ Animals and other organisms
☐	☒ Clinical data
☒	☐ Dual use research of concern

Methods

n/a	Involved in the study
☒	☐ ChIP-seq
☐	☒ Flow cytometry
☒	☐ MRI-based neuroimaging

Antibodies

Antibodies used	The antibodies used for flow cytometry are detailed in Supplementary Information Table 4. Tumour tissue sections were stained with anti-CD3 (clone EP4426, Abcam; anti-rabbit AF647, ThermoFisher), Vector2A RNAScope Probe to identify neoTCR edited cells (Advanced Cell Diagnostics; Opal 570, Akoya Biosciences, Marlborough, MA), and DAPI (ACD).
Validation	Antibodies used for flow cytometry were validated using human PBMCs, isolated T cells, or activated T cells (activated with TransACT for 48-72 h). Antibodies were titrated and fluorescence minus one (FMO) controls were created to set gates for positive events. For tumour tissue staining, anti-CD3 was protein A purified and validated for IHC on Jurkat (Human T cell leukemia T lymphocytes) cells by the manufacturer (Abcam). The Vector2A RNAScope Probe was validated on FFPE neoTCR edited cell pellets, with un-edited cells used as a negative control.

Eukaryotic cell lines

Policy information about cell lines and Sex and Gender in Research

Cell line source(s)	The SW620 colorectal cancer cell line was purchased from ATCC, and a master cell bank was generated. Cells were transduced to express nucLight red, and further edited to insert an R20Q point mutation in COX6C. Cells were again expanded to generate additional working cell banks.
Authentication	Genotyping confirmed editing but cell lines were not further authenticated.
Mycoplasma contamination	All cell banks tested negative for mycoplasma.
Commonly misidentified lines (See ICLAC register)	*Name any commonly misidentified cell lines used in the study and provide a rationale for their use.*

Clinical data

Policy information about clinical studies

All manuscripts should comply with the ICMJE guidelines for publication of clinical research and a completed CONSORT checklist must be included with all submissions.

Clinical trial registration	NCT03970382
Study protocol	The study protocol, "A Phase 1a/1b, Open-label First-in-human Study of the Safety, Tolerability and Feasibility of Gene-edited Autologous NeoTCR-T Cells (NeoTCR-P1) Administered as a Single Agent or in Combination With Anti-PD-1 to Patients With Locally Advanced or Metastatic Solid Tumors" is provided in the Supplemental Information Files.

Data collection	From December 2019 to February 2022, the study was active at 9 investigational sites: City of Hope, Duarte California; University of California Los Angeles, Los Angeles California; University of California, Irvine Medical Center, Orange, California; University of California, Davis, Sacramento California; University of California, San Francisco, San Francisco California; Northwestern University Medical Center, Chicago Illinois; Memorial Sloan Kettering Cancer Center, New York, New York; Tennessee Oncology, Nashville, Tennessee; and Fred Hutchinson Cancer Research Center, Seattle, Washington. All samples were collected at the patients' investigational site. Data was analysed at PACT Pharma.
Outcomes	Primary Outcomes:<br>1. Incidence of adverse events as defined as Dose limiting toxicity (DLT): DLT was defined as protocol-defined adverse events that occur within 28 days following infusion of Neo-TCR-P1 administered as a single agent or in combination with nivolumab.<br>2. Number of participants with adverse events as a measure of safety and tolerability of NeoTCR-P1 or NeoTCR-P1 in combination with nivolumab: Toxicity was classified and graded according to the National Cancer Institute's Common Terminology Criteria for Adverse Events (CTCAE, version 5.0). Cytokine release syndrome (CRS) and neurotoxicity associated with NeoTCR-P1 will be graded according to ASBMT consensus grading.<br>3. Maximum Tolerated Dose (MTD) of NeoTCR-P1: The MTD was defined as the highest dose with an observed incidence of DLT in no more than one out of six patients treated at a particular dose level.<br>4. Feasibility of manufacturing NeoTCR-P1: Percent of screened patients that enrolled on study and receive NeoTCR-P1<br>Secondary Outcomes:<br>1. Maximum concentration of NeoTCR-P1 (Cmax) in the peripheral blood<br>2. Area-under-the-concentration-vs-time-curve (AUC) in the peripheral blood<br>3. Persistence of NeoTCR-P1 in samples of peripheral blood<br>4. Objective Response Rate (ORR) in participants with solid tumors following infusion of NeoTCR-P1 as a single agent or in combination with nivolumab: ORR was defined as Complete Response (CR) or Partial Response (PR) per RECIST v1.1, as determined by the investigator<br>5. Duration of Response mediated by NeoTCR-P1 administered as a single agent or in combination with nivolumab to participants with solid tumors: Duration of response, defined as time from the first occurrence of a documented objective response to the time of relapse or death from any cause<br>6. Progression free survival (PFS) in participants with solid tumors following infusion of NeoTCR-P1 as a single agent or in combination with nivolumab: PFS will be defined from date of administration of NeoTCR-P1 cell infusion to the date of disease progression per the RECIST v1.1 or death as a result of any cause. Subjects who do not meet criteria for progression by the analysis data cut-off date will be censored at their last evaluable disease assessment date<br>7. Overall survival (OS) in participants with solid tumors following infusion of NeoTCR-P1 as a single agent or in combination with nivolumab: OS will be measured from the date of administration of NeoTCR-P1 to the date of death. Subjects who have not died by the analysis data cut-off date will be censored at their last date of contact.

Flow Cytometry

Plots

Confirm that:

☒ The axis labels state the marker and fluorochrome used (e.g. CD4-FITC).

☒ The axis scales are clearly visible. Include numbers along axes only for bottom left plot of group (a 'group' is an analysis of identical markers).

☒ All plots are contour plots with outliers or pseudocolor plots.

☒ A numerical value for number of cells or percentage (with statistics) is provided.

Methodology

Sample preparation	Peripheral Blood Mononuclear Cells (PBMCs) were collected in ACD or CPT tubes and shipped to Precision for Medicine for PBMC isolation and cryopreservation prior to analysis. Apheresis products were obtained from the patient at the study site and shipped overnight to the study sponsor. After cell manufacture, aliquots of T cells were cryopreserved prior to analysis.
Instrument	Flow cytometry data was collected on an Attune NxT, or cell sorted using a FACS Aria III.
Software	Flow cytometry data was collected using BD FACSDiva (V8.0.8) and analysed with FlowJo (V10.7.1 or V10.8.1), or FCS Express (V6.6.21.0).
Cell population abundance	NeoTCR T cells were single-cell sorted from patient PBMCs using two color staining for neoantigen-HLA multimer CD8+ cells, and were detected at a frequency of >1 in 300,000 CD8 T cells. In the incoming cell product, enriched T cells were greater than 90% pure (determined by CD4 and CD8 staining). NeoTCR+ T cell abundance varied with the starting sample but ranged from 1.9-46.8% of live cells in the final cell product and 0.04-37.3% of live cells in post-infusion PBMCs.
Gating strategy	Gating strategies are shown in Supplementary Information Section. Fluorescence minus one (FMO) controls were created to set gates for positive events.

☒ Tick this box to confirm that a figure exemplifying the gating strategy is provided in the Supplementary Information.
